# Supplementary material for: PM2.5 leads to adverse pregnancy outcomes by inducing trophoblast oxidative stress and mitochondrial apoptosis via KLF9/CYP1A1 transcriptional axis
Source: eLife. 2023 Sep 22;12:e85944. doi: 10.7554/eLife.85944 (PMC10584374; doi:10.7554/eLife.85944)
Supplement: Figure 2—source data 1. — (Figure 2F-BCL-2) The expression of BCL-2 expression in the placental tissue of Wild Type (The first three columns) and PM2.5-treated mice (the last three columns). (Figure 2F-BAX) The expression of BAX expression in the placental tissue of Wild Type (The first three columns) and PM2.5-treated mice (the last three columns). (Figure 2F-CC3) The expression of Cleaved-Caspase 3 expression in the placental tissue of Wild Type (The first three columns) and PM2.5-treated mice (the last three columns). (Figure 2F-β-actin) The expression of β-actin expression in the placental tissue of Wild Type (The first three columns) and PM2.5-treated mice (the last three columns). [file elife-85944-fig2-data1.zip › Figure 2-source data 1/Figure2-source data1-Figure legends.docx]

**Figure2F-BCL-2** The expression of BCL-2 expression in the placental tissue of Wild Type (The first three columns) and PM2.5-treated mice (the last three columns).

**Figure2F-BAX** The expression of BAX expression in the placental tissue of Wild Type (The first three columns) and PM2.5-treated mice (the last three columns).

**Figure2F-CC3** The expression of Cleaved-Caspase 3 expression in the placental tissue of Wild Type (The first three columns) and PM2.5-treated mice (the last three columns).

**Figure2F-****β-actin** The expression of β-actin expression in the placental tissue of Wild Type (The first three columns) and PM2.5-treated mice (the last three columns).
