# Supplementary figures and images for: PM2.5 leads to adverse pregnancy outcomes by inducing trophoblast oxidative stress and mitochondrial apoptosis via KLF9/CYP1A1 transcriptional axis (part 1 of 2)
Source: eLife. 2023 Sep 22;12:e85944. doi: 10.7554/eLife.85944 (PMC10584374; doi:10.7554/eLife.85944)

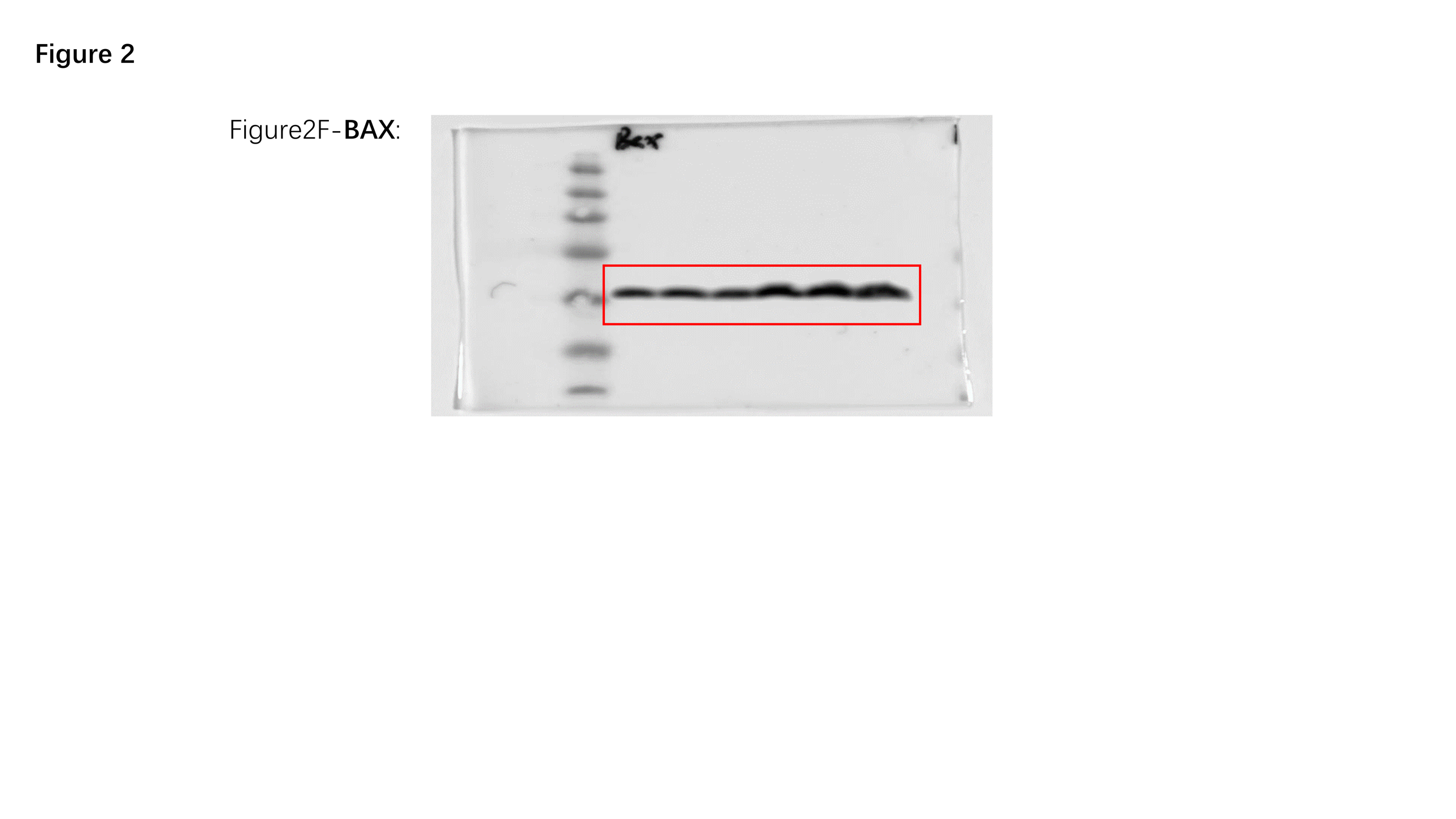

Supplement: Figure 2—source data 1. — (Figure 2F-BCL-2) The expression of BCL-2 expression in the placental tissue of Wild Type (The first three columns) and PM2.5-treated mice (the last three columns). (Figure 2F-BAX) The expression of BAX expression in the placental tissue of Wild Type (The first three columns) and PM2.5-treated mice (the last three columns). (Figure 2F-CC3) The expression of Cleaved-Caspase 3 expression in the placental tissue of Wild Type (The first three columns) and PM2.5-treated mice (the last three columns). (Figure 2F-β-actin) The expression of β-actin expression in the placental tissue of Wild Type (The first three columns) and PM2.5-treated mice (the last three columns). [file elife-85944-fig2-data1.zip › Figure 2-source data 1/Figure2F-BAX.TIF]

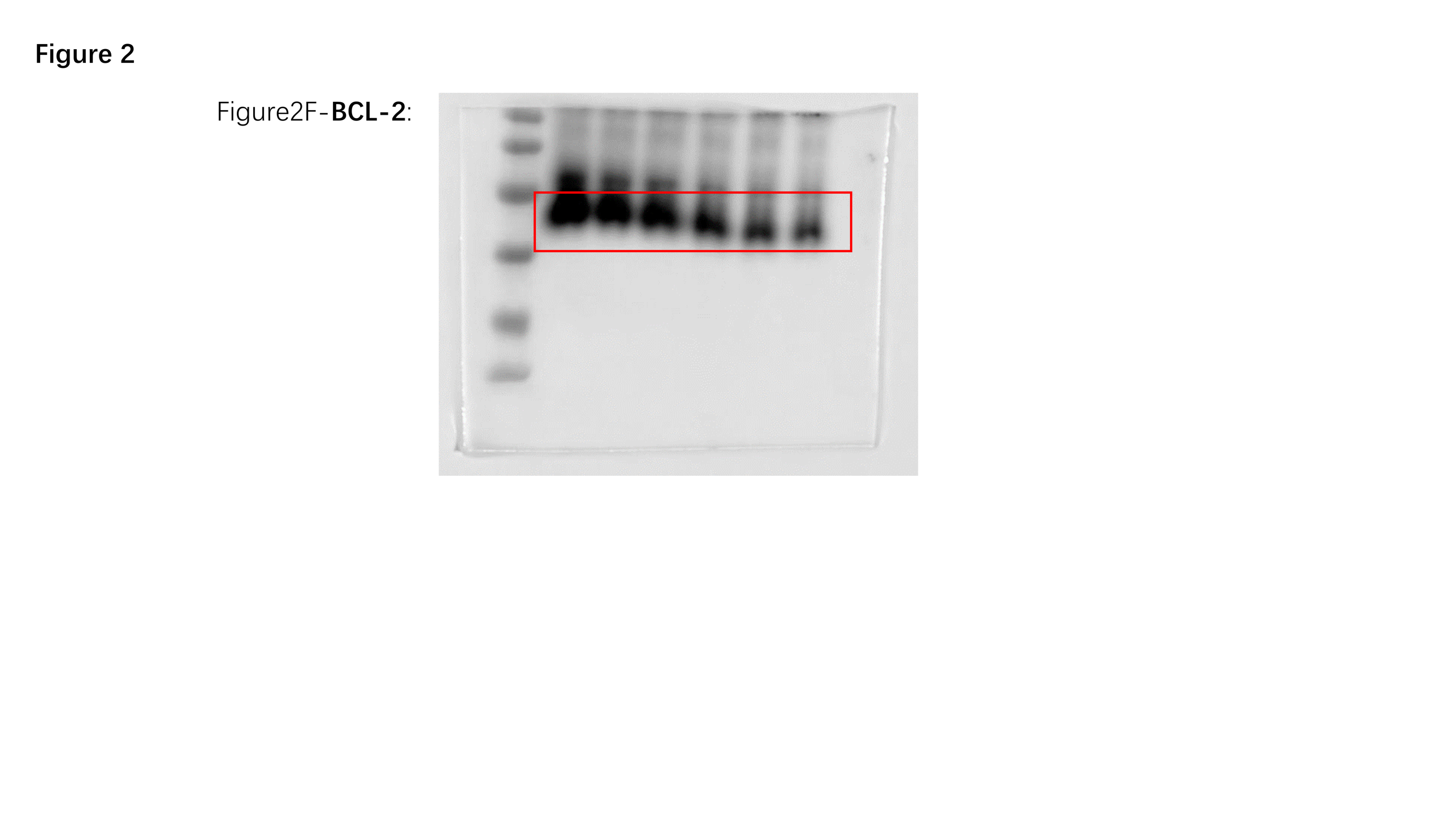

Supplement: Figure 2—source data 1. — (Figure 2F-BCL-2) The expression of BCL-2 expression in the placental tissue of Wild Type (The first three columns) and PM2.5-treated mice (the last three columns). (Figure 2F-BAX) The expression of BAX expression in the placental tissue of Wild Type (The first three columns) and PM2.5-treated mice (the last three columns). (Figure 2F-CC3) The expression of Cleaved-Caspase 3 expression in the placental tissue of Wild Type (The first three columns) and PM2.5-treated mice (the last three columns). (Figure 2F-β-actin) The expression of β-actin expression in the placental tissue of Wild Type (The first three columns) and PM2.5-treated mice (the last three columns). [file elife-85944-fig2-data1.zip › Figure 2-source data 1/Figure2F-BCL2.TIF]

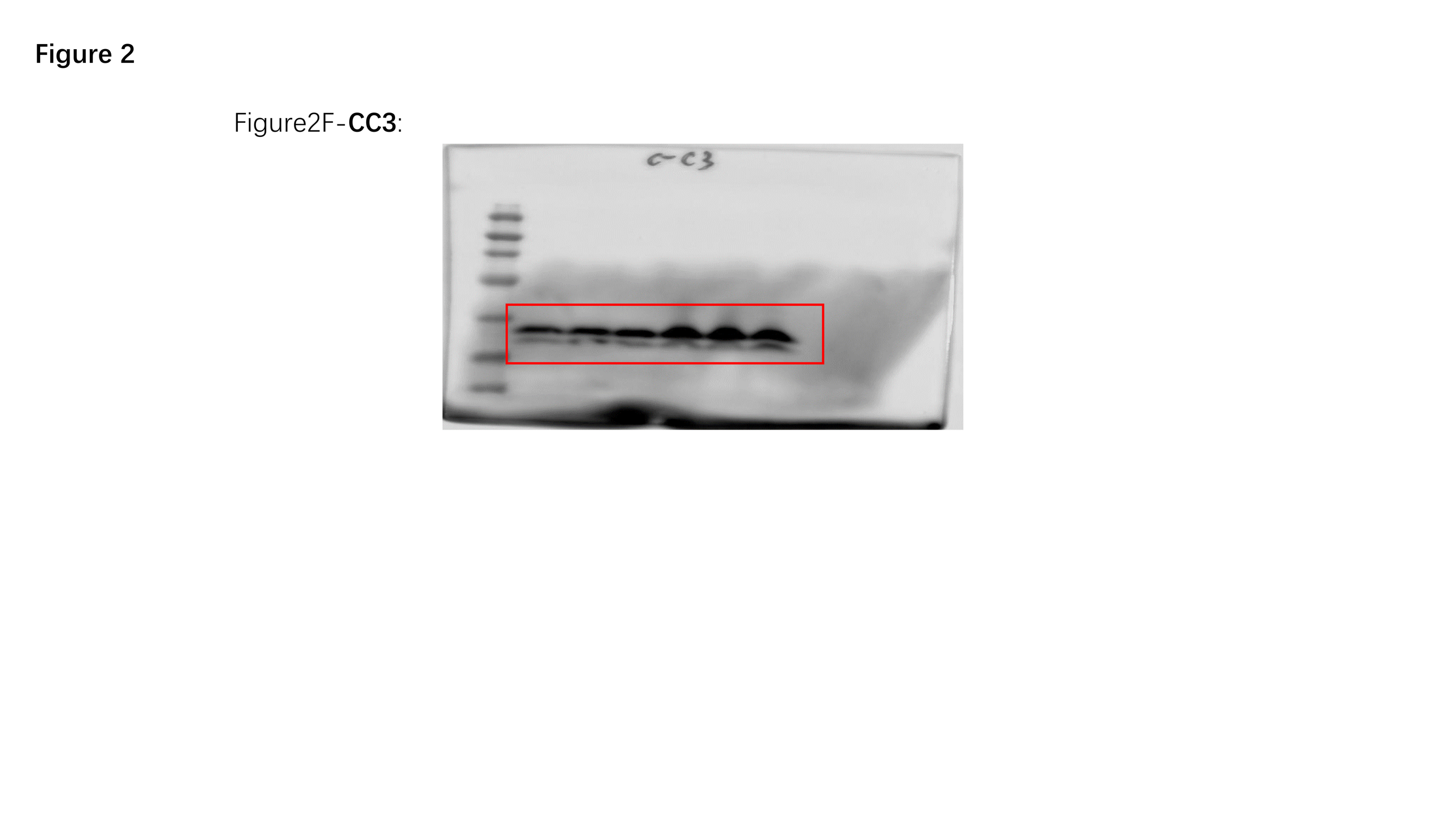

Supplement: Figure 2—source data 1. — (Figure 2F-BCL-2) The expression of BCL-2 expression in the placental tissue of Wild Type (The first three columns) and PM2.5-treated mice (the last three columns). (Figure 2F-BAX) The expression of BAX expression in the placental tissue of Wild Type (The first three columns) and PM2.5-treated mice (the last three columns). (Figure 2F-CC3) The expression of Cleaved-Caspase 3 expression in the placental tissue of Wild Type (The first three columns) and PM2.5-treated mice (the last three columns). (Figure 2F-β-actin) The expression of β-actin expression in the placental tissue of Wild Type (The first three columns) and PM2.5-treated mice (the last three columns). [file elife-85944-fig2-data1.zip › Figure 2-source data 1/Figure2F-CC3.TIF]

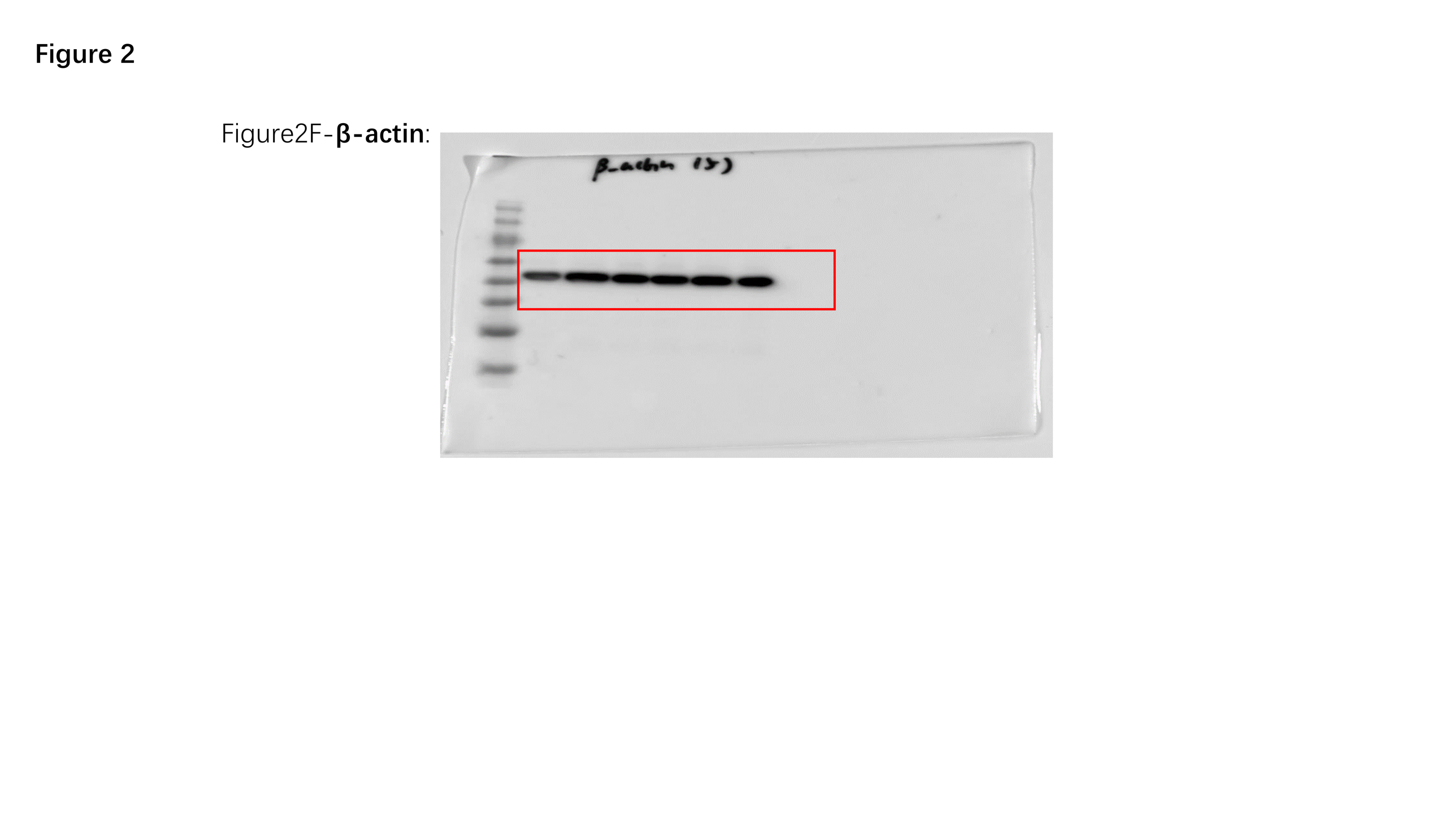

Supplement: Figure 2—source data 1. — (Figure 2F-BCL-2) The expression of BCL-2 expression in the placental tissue of Wild Type (The first three columns) and PM2.5-treated mice (the last three columns). (Figure 2F-BAX) The expression of BAX expression in the placental tissue of Wild Type (The first three columns) and PM2.5-treated mice (the last three columns). (Figure 2F-CC3) The expression of Cleaved-Caspase 3 expression in the placental tissue of Wild Type (The first three columns) and PM2.5-treated mice (the last three columns). (Figure 2F-β-actin) The expression of β-actin expression in the placental tissue of Wild Type (The first three columns) and PM2.5-treated mice (the last three columns). [file elife-85944-fig2-data1.zip › Figure 2-source data 1/Figure2F-a┬-actin.TIF]

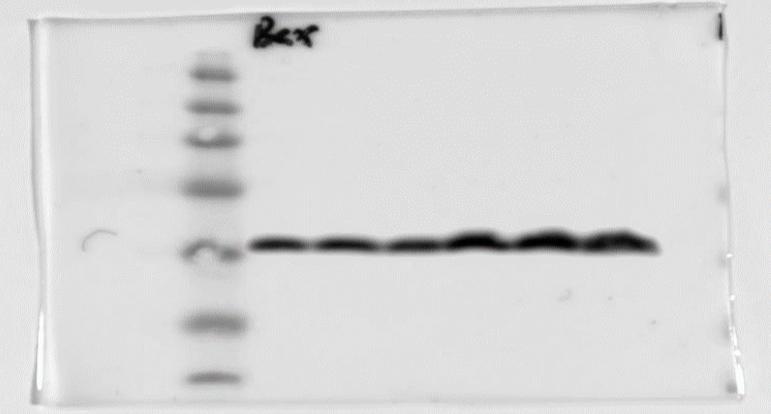

Supplement: Figure 2—source data 2. — (Figure 2F-BCL-2) The expression of BCL-2 expression in the placental tissue of Wild Type (The first three columns) and PM2.5-treated mice (the last three columns). (Figure 2F-BAX) The expression of BAX expression in the placental tissue of Wild Type (The first three columns) and PM2.5-treated mice (the last three columns). (Figure 2F-CC3) The expression of Cleaved-Caspase 3 expression in the placental tissue of Wild Type (The first three columns) and PM2.5-treated mice (the last three columns). (Figure 2F-β-actin) The expression of β-actin expression in the placental tissue of Wild Type (The first three columns) and PM2.5-treated mice (the last three columns). [file elife-85944-fig2-data2.zip › Figure 2-source data 2/Figure2F-BAX.tif]

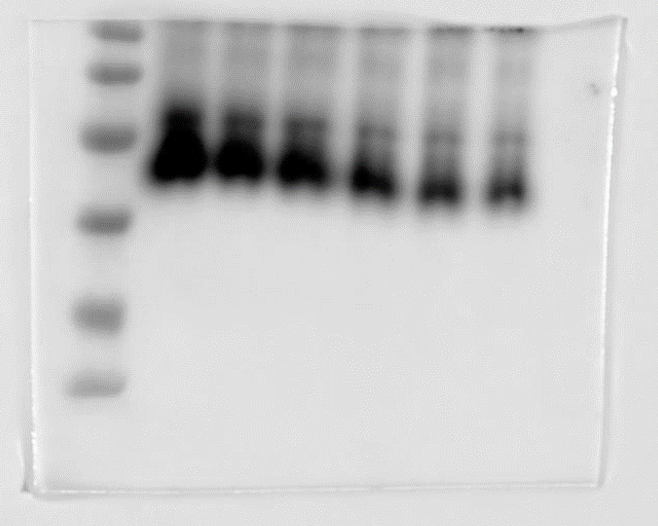

Supplement: Figure 2—source data 2. — (Figure 2F-BCL-2) The expression of BCL-2 expression in the placental tissue of Wild Type (The first three columns) and PM2.5-treated mice (the last three columns). (Figure 2F-BAX) The expression of BAX expression in the placental tissue of Wild Type (The first three columns) and PM2.5-treated mice (the last three columns). (Figure 2F-CC3) The expression of Cleaved-Caspase 3 expression in the placental tissue of Wild Type (The first three columns) and PM2.5-treated mice (the last three columns). (Figure 2F-β-actin) The expression of β-actin expression in the placental tissue of Wild Type (The first three columns) and PM2.5-treated mice (the last three columns). [file elife-85944-fig2-data2.zip › Figure 2-source data 2/Figure2F-BCL-2.tif]

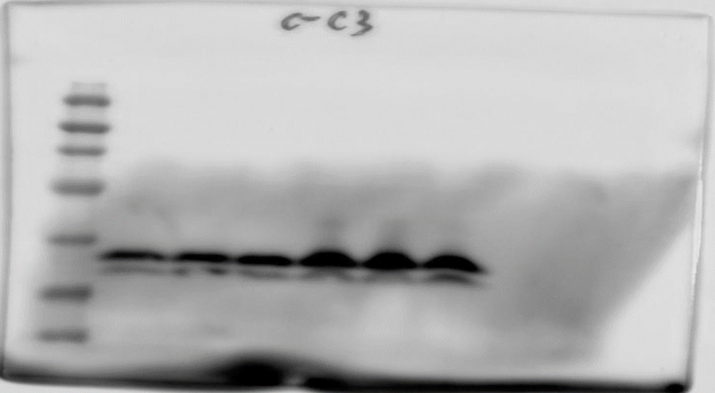

Supplement: Figure 2—source data 2. — (Figure 2F-BCL-2) The expression of BCL-2 expression in the placental tissue of Wild Type (The first three columns) and PM2.5-treated mice (the last three columns). (Figure 2F-BAX) The expression of BAX expression in the placental tissue of Wild Type (The first three columns) and PM2.5-treated mice (the last three columns). (Figure 2F-CC3) The expression of Cleaved-Caspase 3 expression in the placental tissue of Wild Type (The first three columns) and PM2.5-treated mice (the last three columns). (Figure 2F-β-actin) The expression of β-actin expression in the placental tissue of Wild Type (The first three columns) and PM2.5-treated mice (the last three columns). [file elife-85944-fig2-data2.zip › Figure 2-source data 2/Figure2F-CC3.tif]

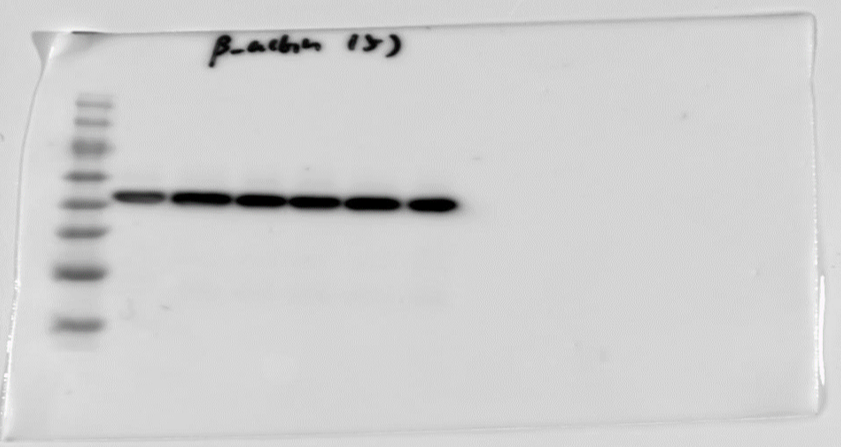

Supplement: Figure 2—source data 2. — (Figure 2F-BCL-2) The expression of BCL-2 expression in the placental tissue of Wild Type (The first three columns) and PM2.5-treated mice (the last three columns). (Figure 2F-BAX) The expression of BAX expression in the placental tissue of Wild Type (The first three columns) and PM2.5-treated mice (the last three columns). (Figure 2F-CC3) The expression of Cleaved-Caspase 3 expression in the placental tissue of Wild Type (The first three columns) and PM2.5-treated mice (the last three columns). (Figure 2F-β-actin) The expression of β-actin expression in the placental tissue of Wild Type (The first three columns) and PM2.5-treated mice (the last three columns). [file elife-85944-fig2-data2.zip › Figure 2-source data 2/Figure2F-a┬-actin.tif]

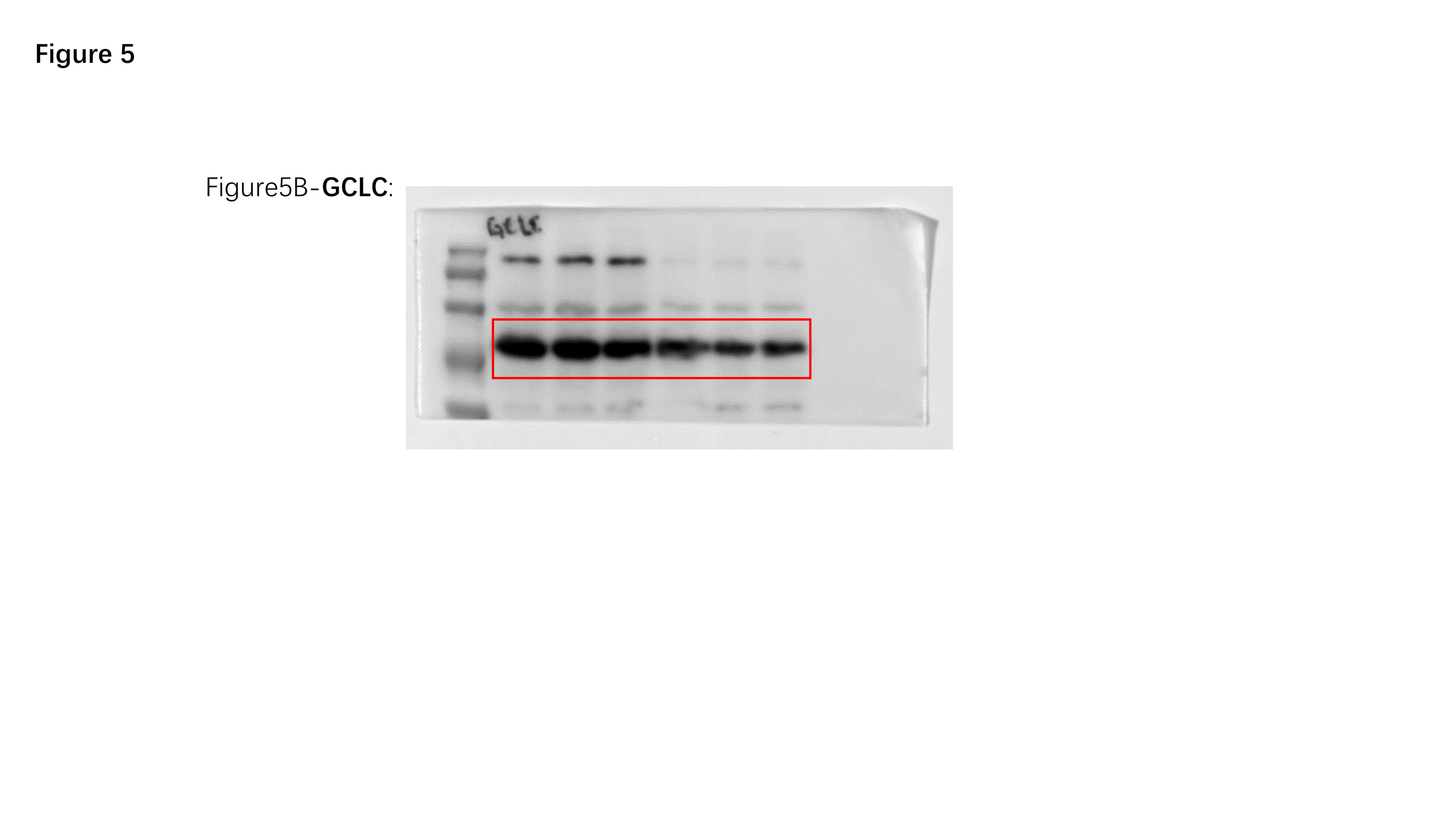

Supplement: Figure 5—source data 1. — (Figure 5B-HO-1) The expression of HO-1 expression in the placental tissue of Wild Type (The first three columns) and PM2.5-treated mice (the last three columns) (Figure 5B-NQO-1). The expression of NQO-1 expression in the placental tissue of Wild Type (The first three columns) and PM2.5-treated mice (the last three columns) (Figure 5B-GCLC). The expression of GCLC expression in the placental tissue of Wild Type (The first three columns) and PM2.5-treated mice (the last three columns) (Figure 5B-SOD-1). The expression of SOD-1 expression in the placental tissue of Wild Type (The first three columns) and PM2.5-treated mice (the last three columns) (Figure 5B-β-actin). The expression of β-actin expression in the placental tissue of Wild Type (The first three columns) and PM2.5-treated mice (the last three columns) (Figure 5C-HO-1). The expression of HO-1 expression in the PM2.5-treated HTR8/SVneo cells (PM2.5 concentration: 0. 50 μg/mL, 100 μg/mL, 200 μg/mL) (Figure 5C-NQO-1). The expression of NQO-1 expression in the PM2.5-treated HTR8/SVneo cells (PM2.5 concentration: 0. 50 μg/mL, 100 μg/mL, 200 μg/mL) (Figure 5C-GCLC). The expression of GCLC expression in the PM2.5-treated HTR8/SVneo cells (PM2.5 concentration: 0. 50 μg/mL, 100 μg/mL, 200 μg/mL) (Figure 5C-SOD-1). The expression of SOD-1 expression in the PM2.5-treated HTR8/SVneo cells (PM2.5 concentration: 0. 50 μg/mL, 100 μg/mL, 200 μg/mL) (Figure 5C-β-actin). The expression of β-actin expression in the PM2.5-treated HTR8/SVneo cells (PM2.5 concentration: 0. 50 μg/mL, 100 μg/mL, 200 μg/mL) (Figure 5K-Mito-Cyt-c). The expression of Cyt-c expression in mitochondria after treated with different concentration of PM2.5 (0, 50 μg/mL, 100 μg/mL, 200 μg/mL) (Figure 5K-Mito-VDAC-1). The expression of VDAC-1 expression in mitochondria after treated with different concentration of PM2.5 (0, 50 μg/mL, 100 μg/mL, 200 μg/mL) (Figure 5K-Cyto-Cyt-c). The expression of Cyt-c expression in cytoplasm after treated with different co [file elife-85944-fig5-data1.zip › Figure 5-source data 1/Figure5B-GCLC.TIF]

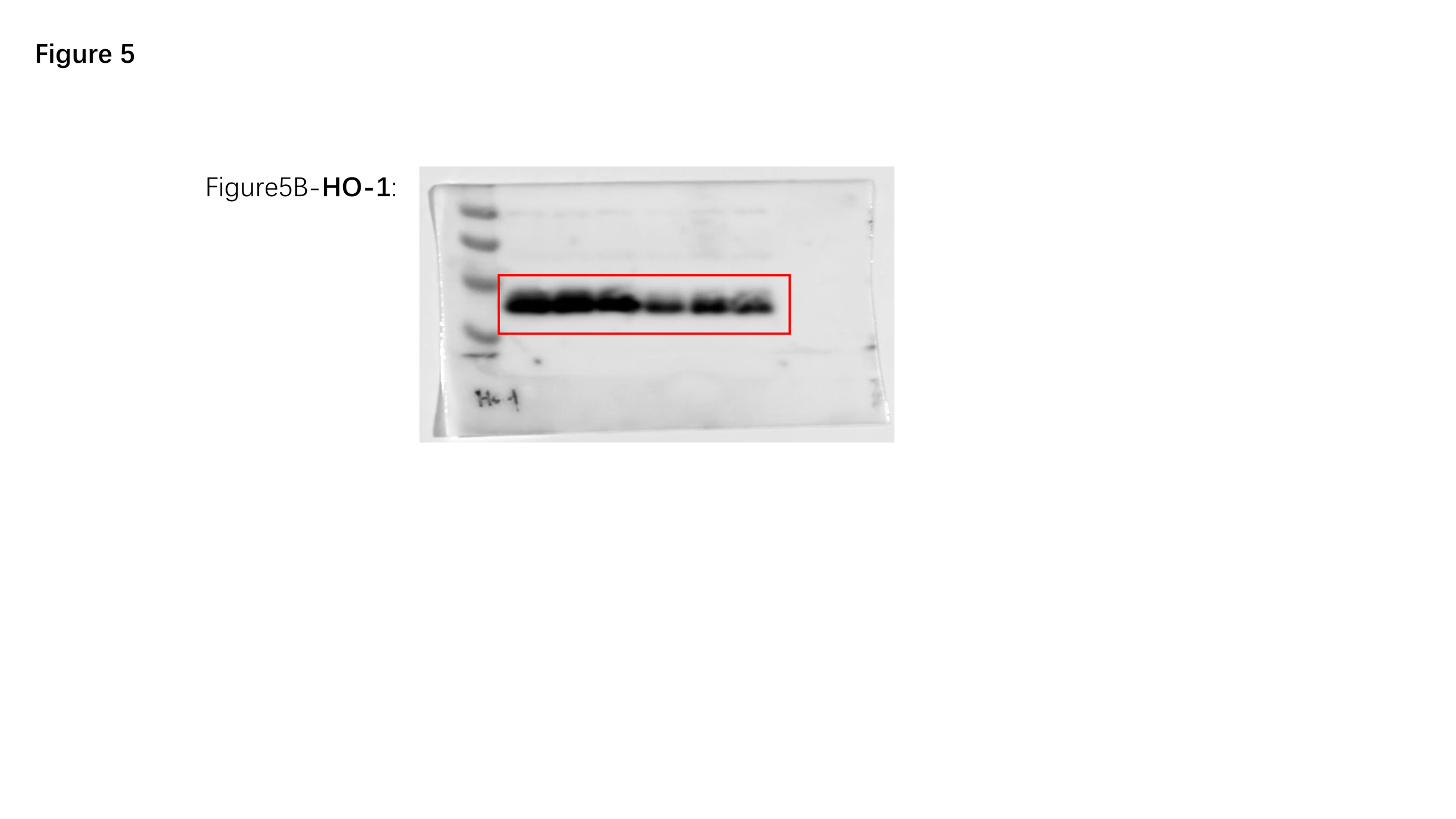

Supplement: Figure 5—source data 1. — (Figure 5B-HO-1) The expression of HO-1 expression in the placental tissue of Wild Type (The first three columns) and PM2.5-treated mice (the last three columns) (Figure 5B-NQO-1). The expression of NQO-1 expression in the placental tissue of Wild Type (The first three columns) and PM2.5-treated mice (the last three columns) (Figure 5B-GCLC). The expression of GCLC expression in the placental tissue of Wild Type (The first three columns) and PM2.5-treated mice (the last three columns) (Figure 5B-SOD-1). The expression of SOD-1 expression in the placental tissue of Wild Type (The first three columns) and PM2.5-treated mice (the last three columns) (Figure 5B-β-actin). The expression of β-actin expression in the placental tissue of Wild Type (The first three columns) and PM2.5-treated mice (the last three columns) (Figure 5C-HO-1). The expression of HO-1 expression in the PM2.5-treated HTR8/SVneo cells (PM2.5 concentration: 0. 50 μg/mL, 100 μg/mL, 200 μg/mL) (Figure 5C-NQO-1). The expression of NQO-1 expression in the PM2.5-treated HTR8/SVneo cells (PM2.5 concentration: 0. 50 μg/mL, 100 μg/mL, 200 μg/mL) (Figure 5C-GCLC). The expression of GCLC expression in the PM2.5-treated HTR8/SVneo cells (PM2.5 concentration: 0. 50 μg/mL, 100 μg/mL, 200 μg/mL) (Figure 5C-SOD-1). The expression of SOD-1 expression in the PM2.5-treated HTR8/SVneo cells (PM2.5 concentration: 0. 50 μg/mL, 100 μg/mL, 200 μg/mL) (Figure 5C-β-actin). The expression of β-actin expression in the PM2.5-treated HTR8/SVneo cells (PM2.5 concentration: 0. 50 μg/mL, 100 μg/mL, 200 μg/mL) (Figure 5K-Mito-Cyt-c). The expression of Cyt-c expression in mitochondria after treated with different concentration of PM2.5 (0, 50 μg/mL, 100 μg/mL, 200 μg/mL) (Figure 5K-Mito-VDAC-1). The expression of VDAC-1 expression in mitochondria after treated with different concentration of PM2.5 (0, 50 μg/mL, 100 μg/mL, 200 μg/mL) (Figure 5K-Cyto-Cyt-c). The expression of Cyt-c expression in cytoplasm after treated with different co [file elife-85944-fig5-data1.zip › Figure 5-source data 1/Figure5B-HO-1.TIF]

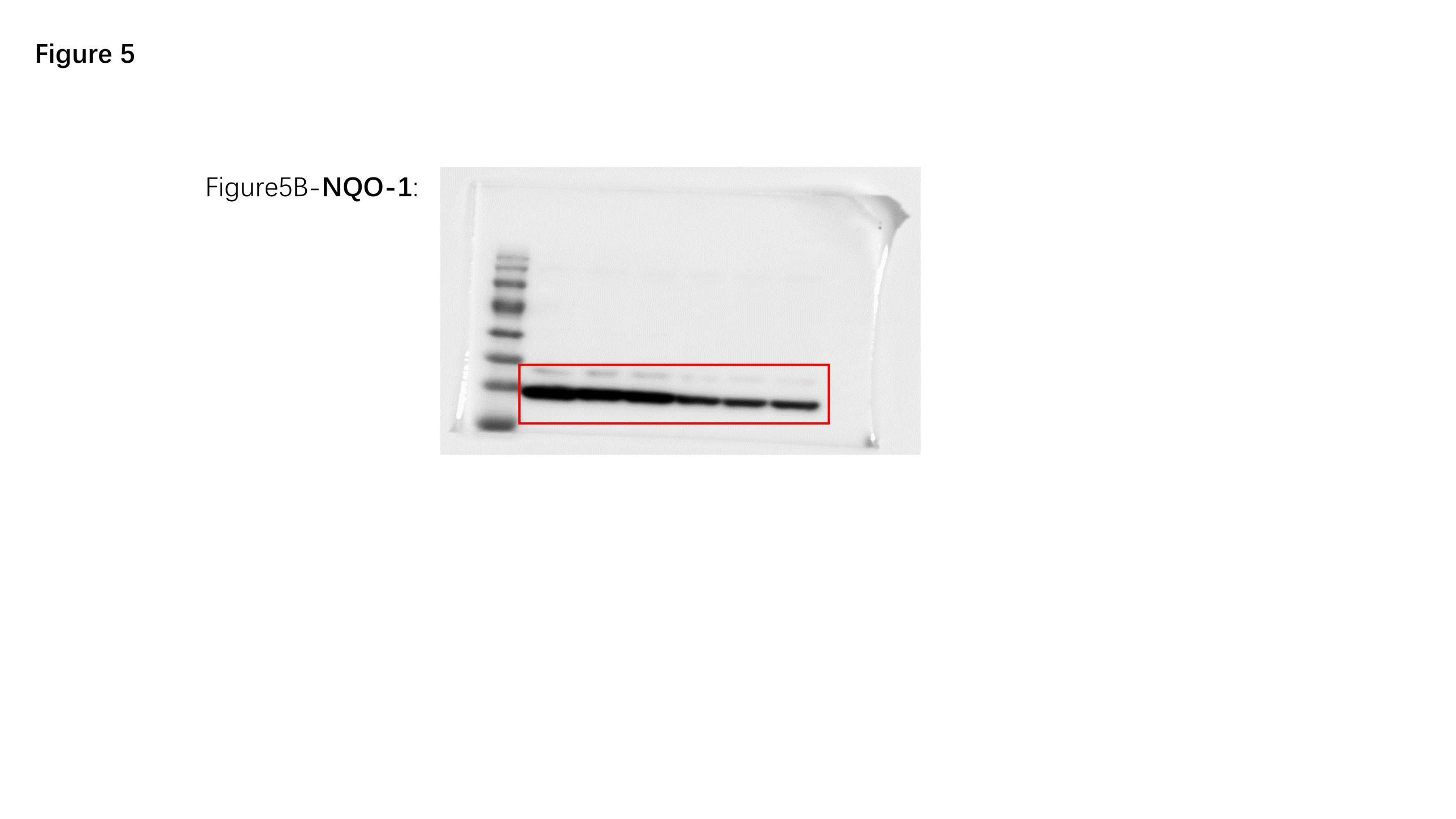

Supplement: Figure 5—source data 1. — (Figure 5B-HO-1) The expression of HO-1 expression in the placental tissue of Wild Type (The first three columns) and PM2.5-treated mice (the last three columns) (Figure 5B-NQO-1). The expression of NQO-1 expression in the placental tissue of Wild Type (The first three columns) and PM2.5-treated mice (the last three columns) (Figure 5B-GCLC). The expression of GCLC expression in the placental tissue of Wild Type (The first three columns) and PM2.5-treated mice (the last three columns) (Figure 5B-SOD-1). The expression of SOD-1 expression in the placental tissue of Wild Type (The first three columns) and PM2.5-treated mice (the last three columns) (Figure 5B-β-actin). The expression of β-actin expression in the placental tissue of Wild Type (The first three columns) and PM2.5-treated mice (the last three columns) (Figure 5C-HO-1). The expression of HO-1 expression in the PM2.5-treated HTR8/SVneo cells (PM2.5 concentration: 0. 50 μg/mL, 100 μg/mL, 200 μg/mL) (Figure 5C-NQO-1). The expression of NQO-1 expression in the PM2.5-treated HTR8/SVneo cells (PM2.5 concentration: 0. 50 μg/mL, 100 μg/mL, 200 μg/mL) (Figure 5C-GCLC). The expression of GCLC expression in the PM2.5-treated HTR8/SVneo cells (PM2.5 concentration: 0. 50 μg/mL, 100 μg/mL, 200 μg/mL) (Figure 5C-SOD-1). The expression of SOD-1 expression in the PM2.5-treated HTR8/SVneo cells (PM2.5 concentration: 0. 50 μg/mL, 100 μg/mL, 200 μg/mL) (Figure 5C-β-actin). The expression of β-actin expression in the PM2.5-treated HTR8/SVneo cells (PM2.5 concentration: 0. 50 μg/mL, 100 μg/mL, 200 μg/mL) (Figure 5K-Mito-Cyt-c). The expression of Cyt-c expression in mitochondria after treated with different concentration of PM2.5 (0, 50 μg/mL, 100 μg/mL, 200 μg/mL) (Figure 5K-Mito-VDAC-1). The expression of VDAC-1 expression in mitochondria after treated with different concentration of PM2.5 (0, 50 μg/mL, 100 μg/mL, 200 μg/mL) (Figure 5K-Cyto-Cyt-c). The expression of Cyt-c expression in cytoplasm after treated with different co [file elife-85944-fig5-data1.zip › Figure 5-source data 1/Figure5B-NQO-1.TIF]

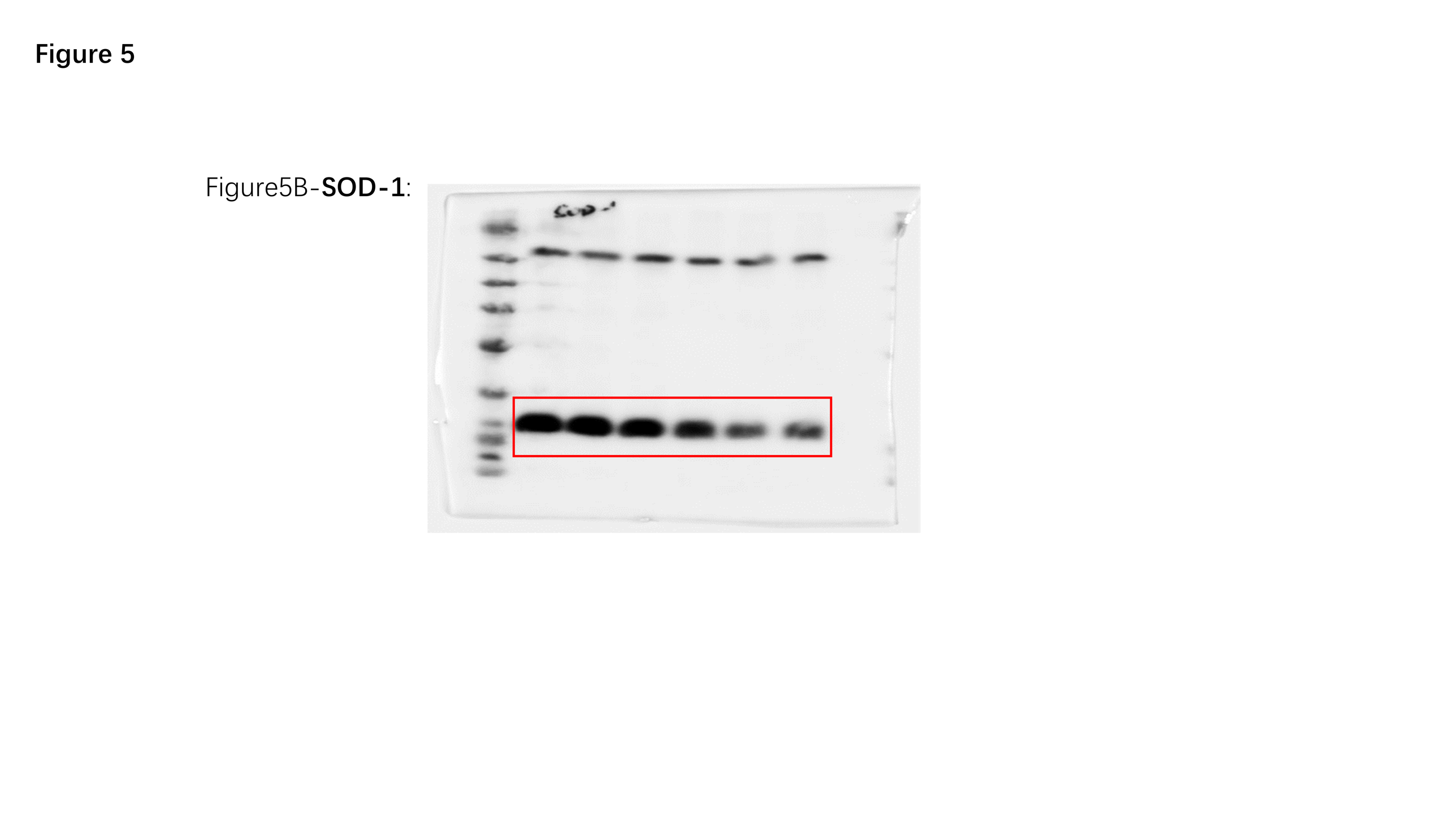

Supplement: Figure 5—source data 1. — (Figure 5B-HO-1) The expression of HO-1 expression in the placental tissue of Wild Type (The first three columns) and PM2.5-treated mice (the last three columns) (Figure 5B-NQO-1). The expression of NQO-1 expression in the placental tissue of Wild Type (The first three columns) and PM2.5-treated mice (the last three columns) (Figure 5B-GCLC). The expression of GCLC expression in the placental tissue of Wild Type (The first three columns) and PM2.5-treated mice (the last three columns) (Figure 5B-SOD-1). The expression of SOD-1 expression in the placental tissue of Wild Type (The first three columns) and PM2.5-treated mice (the last three columns) (Figure 5B-β-actin). The expression of β-actin expression in the placental tissue of Wild Type (The first three columns) and PM2.5-treated mice (the last three columns) (Figure 5C-HO-1). The expression of HO-1 expression in the PM2.5-treated HTR8/SVneo cells (PM2.5 concentration: 0. 50 μg/mL, 100 μg/mL, 200 μg/mL) (Figure 5C-NQO-1). The expression of NQO-1 expression in the PM2.5-treated HTR8/SVneo cells (PM2.5 concentration: 0. 50 μg/mL, 100 μg/mL, 200 μg/mL) (Figure 5C-GCLC). The expression of GCLC expression in the PM2.5-treated HTR8/SVneo cells (PM2.5 concentration: 0. 50 μg/mL, 100 μg/mL, 200 μg/mL) (Figure 5C-SOD-1). The expression of SOD-1 expression in the PM2.5-treated HTR8/SVneo cells (PM2.5 concentration: 0. 50 μg/mL, 100 μg/mL, 200 μg/mL) (Figure 5C-β-actin). The expression of β-actin expression in the PM2.5-treated HTR8/SVneo cells (PM2.5 concentration: 0. 50 μg/mL, 100 μg/mL, 200 μg/mL) (Figure 5K-Mito-Cyt-c). The expression of Cyt-c expression in mitochondria after treated with different concentration of PM2.5 (0, 50 μg/mL, 100 μg/mL, 200 μg/mL) (Figure 5K-Mito-VDAC-1). The expression of VDAC-1 expression in mitochondria after treated with different concentration of PM2.5 (0, 50 μg/mL, 100 μg/mL, 200 μg/mL) (Figure 5K-Cyto-Cyt-c). The expression of Cyt-c expression in cytoplasm after treated with different co [file elife-85944-fig5-data1.zip › Figure 5-source data 1/Figure5B-SOD-1.TIF]

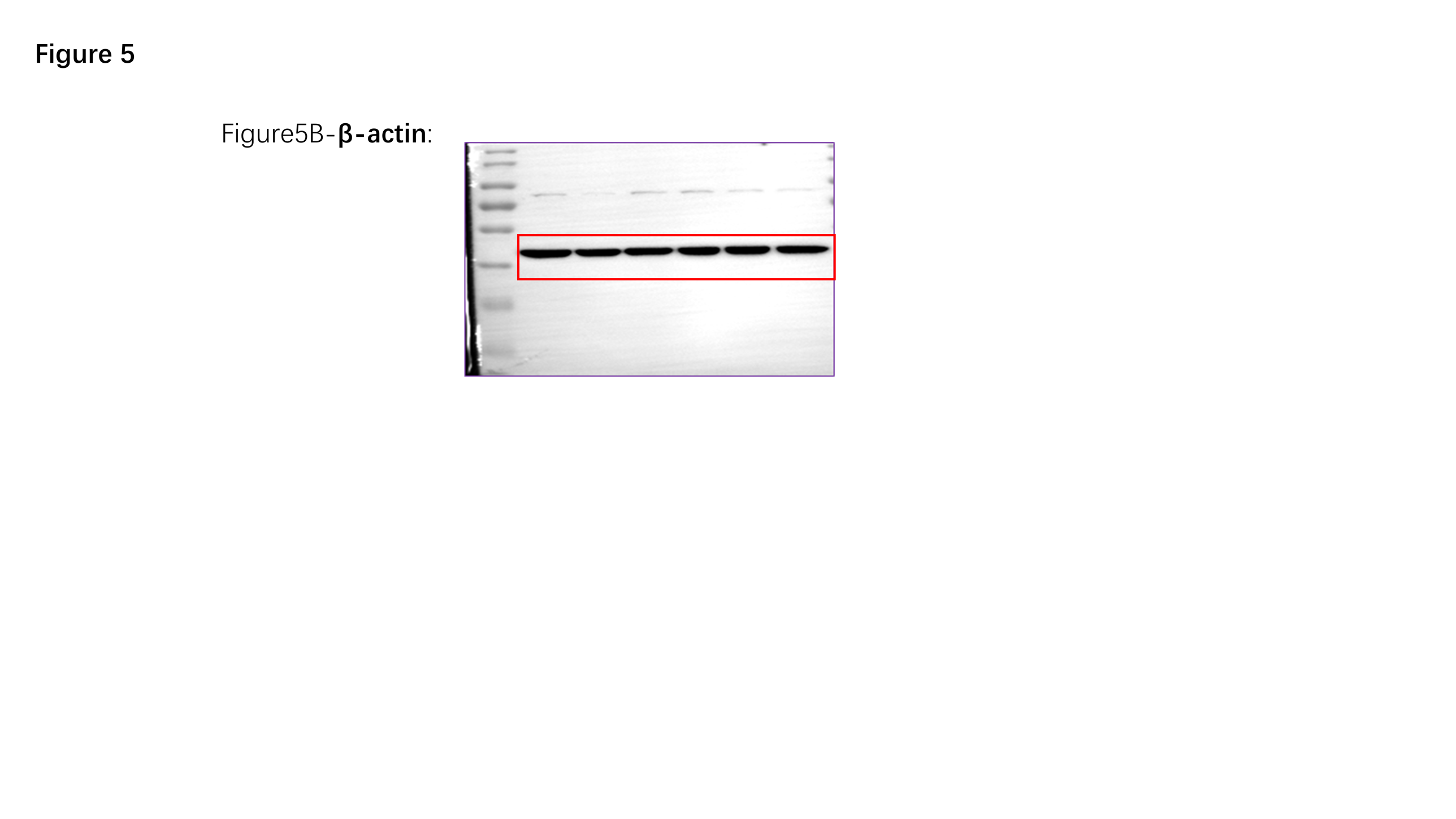

Supplement: Figure 5—source data 1. — (Figure 5B-HO-1) The expression of HO-1 expression in the placental tissue of Wild Type (The first three columns) and PM2.5-treated mice (the last three columns) (Figure 5B-NQO-1). The expression of NQO-1 expression in the placental tissue of Wild Type (The first three columns) and PM2.5-treated mice (the last three columns) (Figure 5B-GCLC). The expression of GCLC expression in the placental tissue of Wild Type (The first three columns) and PM2.5-treated mice (the last three columns) (Figure 5B-SOD-1). The expression of SOD-1 expression in the placental tissue of Wild Type (The first three columns) and PM2.5-treated mice (the last three columns) (Figure 5B-β-actin). The expression of β-actin expression in the placental tissue of Wild Type (The first three columns) and PM2.5-treated mice (the last three columns) (Figure 5C-HO-1). The expression of HO-1 expression in the PM2.5-treated HTR8/SVneo cells (PM2.5 concentration: 0. 50 μg/mL, 100 μg/mL, 200 μg/mL) (Figure 5C-NQO-1). The expression of NQO-1 expression in the PM2.5-treated HTR8/SVneo cells (PM2.5 concentration: 0. 50 μg/mL, 100 μg/mL, 200 μg/mL) (Figure 5C-GCLC). The expression of GCLC expression in the PM2.5-treated HTR8/SVneo cells (PM2.5 concentration: 0. 50 μg/mL, 100 μg/mL, 200 μg/mL) (Figure 5C-SOD-1). The expression of SOD-1 expression in the PM2.5-treated HTR8/SVneo cells (PM2.5 concentration: 0. 50 μg/mL, 100 μg/mL, 200 μg/mL) (Figure 5C-β-actin). The expression of β-actin expression in the PM2.5-treated HTR8/SVneo cells (PM2.5 concentration: 0. 50 μg/mL, 100 μg/mL, 200 μg/mL) (Figure 5K-Mito-Cyt-c). The expression of Cyt-c expression in mitochondria after treated with different concentration of PM2.5 (0, 50 μg/mL, 100 μg/mL, 200 μg/mL) (Figure 5K-Mito-VDAC-1). The expression of VDAC-1 expression in mitochondria after treated with different concentration of PM2.5 (0, 50 μg/mL, 100 μg/mL, 200 μg/mL) (Figure 5K-Cyto-Cyt-c). The expression of Cyt-c expression in cytoplasm after treated with different co [file elife-85944-fig5-data1.zip › Figure 5-source data 1/Figure5B-a┬-actin.TIF]

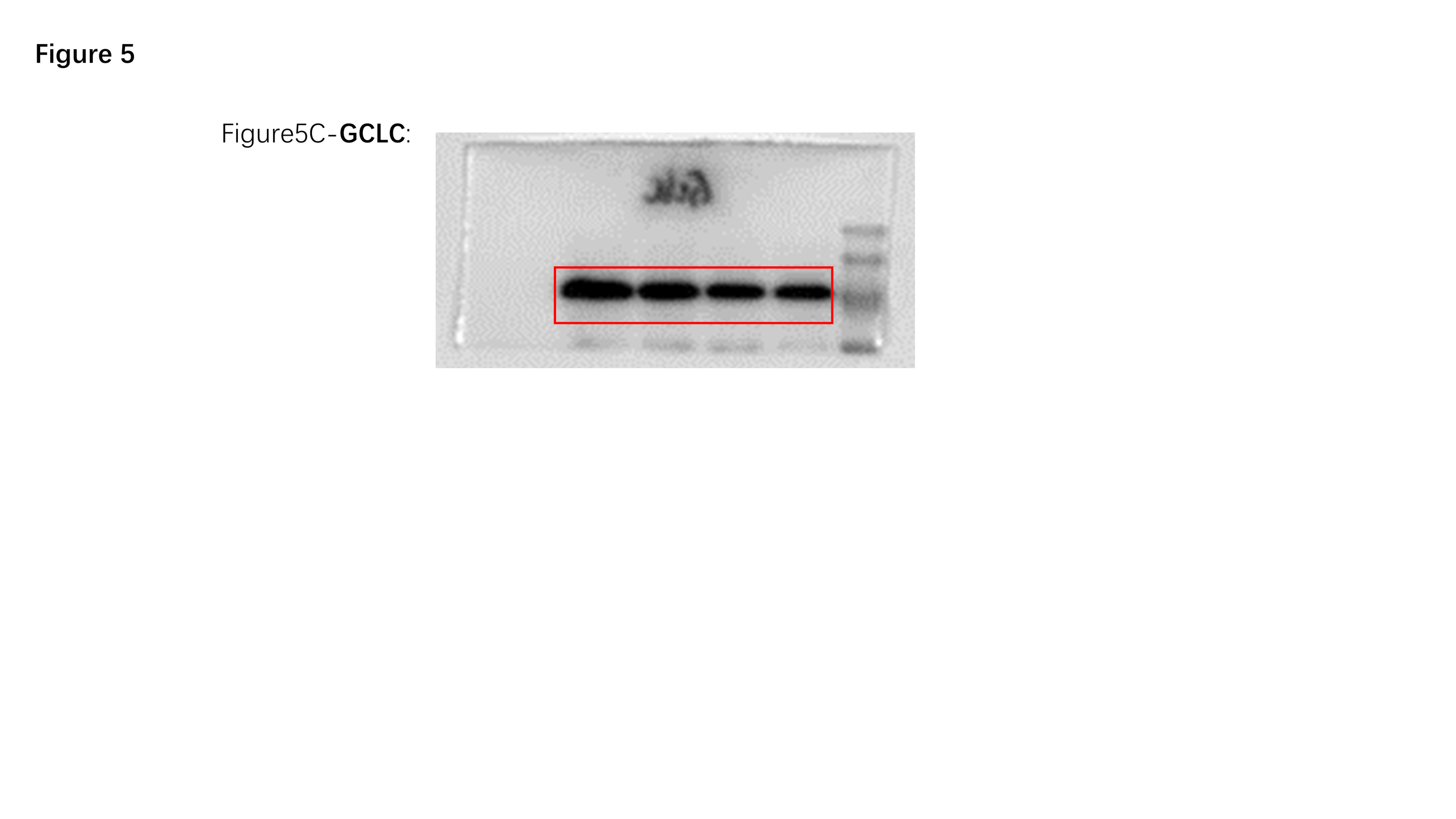

Supplement: Figure 5—source data 1. — (Figure 5B-HO-1) The expression of HO-1 expression in the placental tissue of Wild Type (The first three columns) and PM2.5-treated mice (the last three columns) (Figure 5B-NQO-1). The expression of NQO-1 expression in the placental tissue of Wild Type (The first three columns) and PM2.5-treated mice (the last three columns) (Figure 5B-GCLC). The expression of GCLC expression in the placental tissue of Wild Type (The first three columns) and PM2.5-treated mice (the last three columns) (Figure 5B-SOD-1). The expression of SOD-1 expression in the placental tissue of Wild Type (The first three columns) and PM2.5-treated mice (the last three columns) (Figure 5B-β-actin). The expression of β-actin expression in the placental tissue of Wild Type (The first three columns) and PM2.5-treated mice (the last three columns) (Figure 5C-HO-1). The expression of HO-1 expression in the PM2.5-treated HTR8/SVneo cells (PM2.5 concentration: 0. 50 μg/mL, 100 μg/mL, 200 μg/mL) (Figure 5C-NQO-1). The expression of NQO-1 expression in the PM2.5-treated HTR8/SVneo cells (PM2.5 concentration: 0. 50 μg/mL, 100 μg/mL, 200 μg/mL) (Figure 5C-GCLC). The expression of GCLC expression in the PM2.5-treated HTR8/SVneo cells (PM2.5 concentration: 0. 50 μg/mL, 100 μg/mL, 200 μg/mL) (Figure 5C-SOD-1). The expression of SOD-1 expression in the PM2.5-treated HTR8/SVneo cells (PM2.5 concentration: 0. 50 μg/mL, 100 μg/mL, 200 μg/mL) (Figure 5C-β-actin). The expression of β-actin expression in the PM2.5-treated HTR8/SVneo cells (PM2.5 concentration: 0. 50 μg/mL, 100 μg/mL, 200 μg/mL) (Figure 5K-Mito-Cyt-c). The expression of Cyt-c expression in mitochondria after treated with different concentration of PM2.5 (0, 50 μg/mL, 100 μg/mL, 200 μg/mL) (Figure 5K-Mito-VDAC-1). The expression of VDAC-1 expression in mitochondria after treated with different concentration of PM2.5 (0, 50 μg/mL, 100 μg/mL, 200 μg/mL) (Figure 5K-Cyto-Cyt-c). The expression of Cyt-c expression in cytoplasm after treated with different co [file elife-85944-fig5-data1.zip › Figure 5-source data 1/Figure5C-GCLC.TIF]

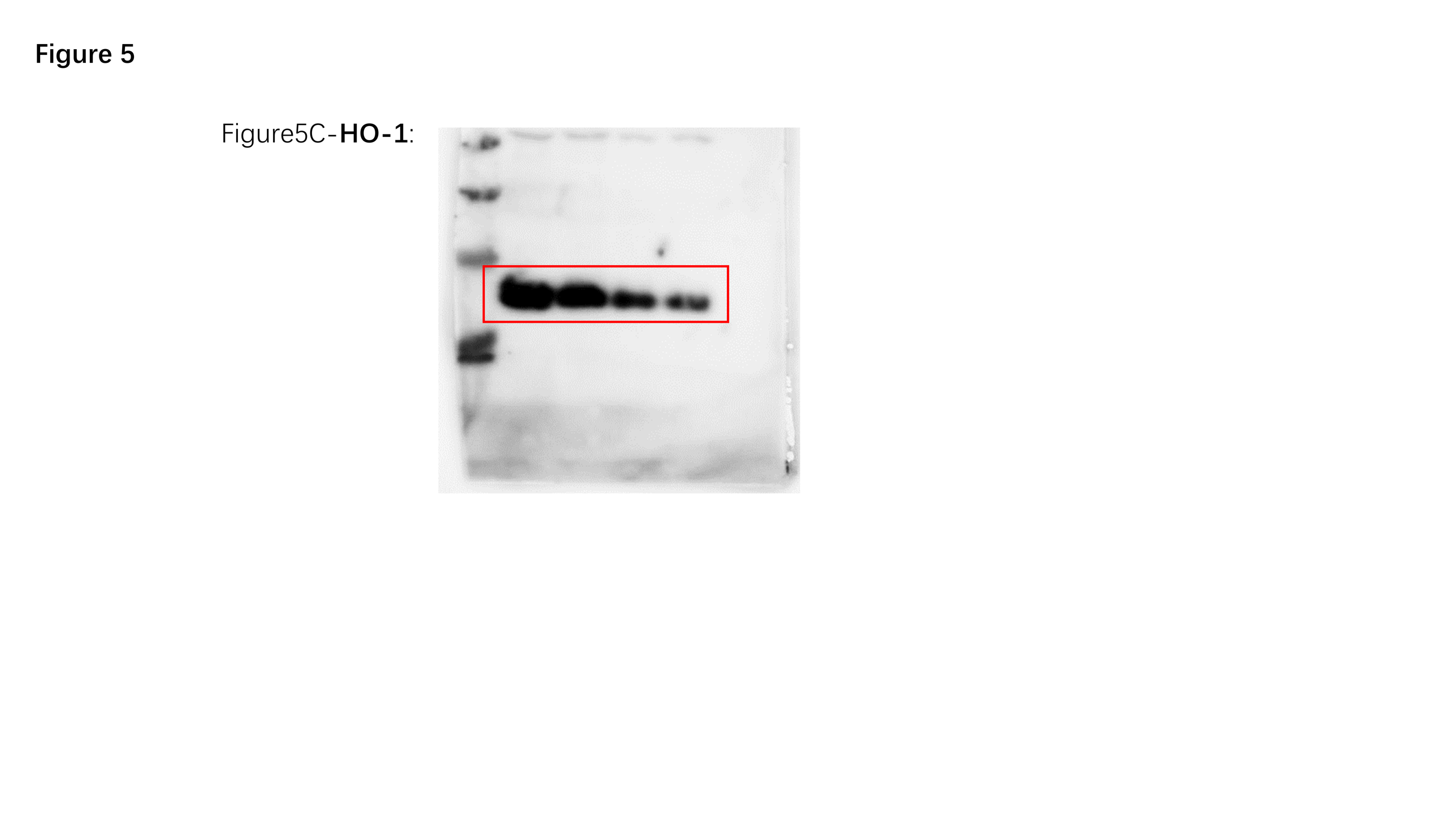

Supplement: Figure 5—source data 1. — (Figure 5B-HO-1) The expression of HO-1 expression in the placental tissue of Wild Type (The first three columns) and PM2.5-treated mice (the last three columns) (Figure 5B-NQO-1). The expression of NQO-1 expression in the placental tissue of Wild Type (The first three columns) and PM2.5-treated mice (the last three columns) (Figure 5B-GCLC). The expression of GCLC expression in the placental tissue of Wild Type (The first three columns) and PM2.5-treated mice (the last three columns) (Figure 5B-SOD-1). The expression of SOD-1 expression in the placental tissue of Wild Type (The first three columns) and PM2.5-treated mice (the last three columns) (Figure 5B-β-actin). The expression of β-actin expression in the placental tissue of Wild Type (The first three columns) and PM2.5-treated mice (the last three columns) (Figure 5C-HO-1). The expression of HO-1 expression in the PM2.5-treated HTR8/SVneo cells (PM2.5 concentration: 0. 50 μg/mL, 100 μg/mL, 200 μg/mL) (Figure 5C-NQO-1). The expression of NQO-1 expression in the PM2.5-treated HTR8/SVneo cells (PM2.5 concentration: 0. 50 μg/mL, 100 μg/mL, 200 μg/mL) (Figure 5C-GCLC). The expression of GCLC expression in the PM2.5-treated HTR8/SVneo cells (PM2.5 concentration: 0. 50 μg/mL, 100 μg/mL, 200 μg/mL) (Figure 5C-SOD-1). The expression of SOD-1 expression in the PM2.5-treated HTR8/SVneo cells (PM2.5 concentration: 0. 50 μg/mL, 100 μg/mL, 200 μg/mL) (Figure 5C-β-actin). The expression of β-actin expression in the PM2.5-treated HTR8/SVneo cells (PM2.5 concentration: 0. 50 μg/mL, 100 μg/mL, 200 μg/mL) (Figure 5K-Mito-Cyt-c). The expression of Cyt-c expression in mitochondria after treated with different concentration of PM2.5 (0, 50 μg/mL, 100 μg/mL, 200 μg/mL) (Figure 5K-Mito-VDAC-1). The expression of VDAC-1 expression in mitochondria after treated with different concentration of PM2.5 (0, 50 μg/mL, 100 μg/mL, 200 μg/mL) (Figure 5K-Cyto-Cyt-c). The expression of Cyt-c expression in cytoplasm after treated with different co [file elife-85944-fig5-data1.zip › Figure 5-source data 1/Figure5C-HO-1.TIF]

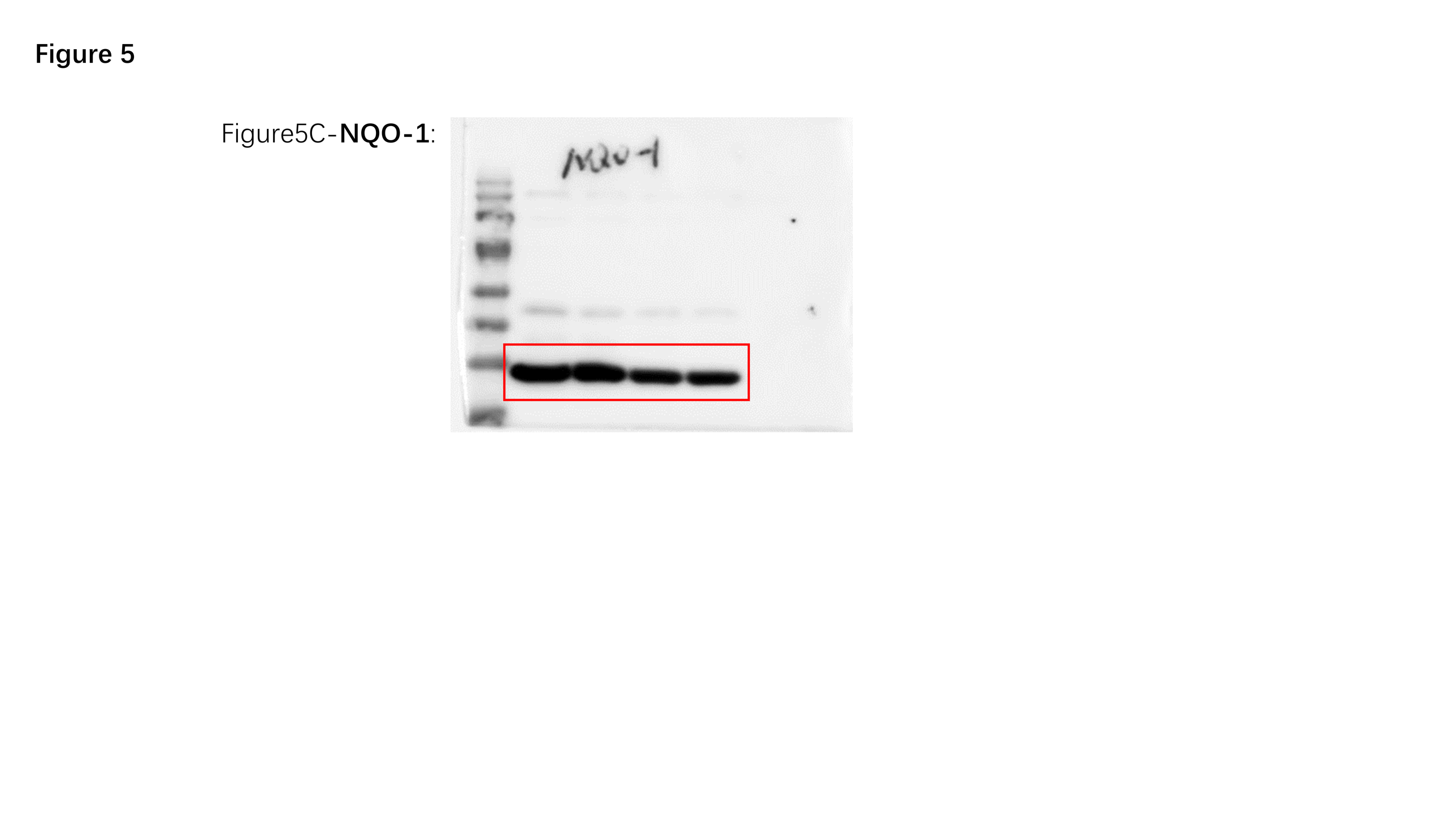

Supplement: Figure 5—source data 1. — (Figure 5B-HO-1) The expression of HO-1 expression in the placental tissue of Wild Type (The first three columns) and PM2.5-treated mice (the last three columns) (Figure 5B-NQO-1). The expression of NQO-1 expression in the placental tissue of Wild Type (The first three columns) and PM2.5-treated mice (the last three columns) (Figure 5B-GCLC). The expression of GCLC expression in the placental tissue of Wild Type (The first three columns) and PM2.5-treated mice (the last three columns) (Figure 5B-SOD-1). The expression of SOD-1 expression in the placental tissue of Wild Type (The first three columns) and PM2.5-treated mice (the last three columns) (Figure 5B-β-actin). The expression of β-actin expression in the placental tissue of Wild Type (The first three columns) and PM2.5-treated mice (the last three columns) (Figure 5C-HO-1). The expression of HO-1 expression in the PM2.5-treated HTR8/SVneo cells (PM2.5 concentration: 0. 50 μg/mL, 100 μg/mL, 200 μg/mL) (Figure 5C-NQO-1). The expression of NQO-1 expression in the PM2.5-treated HTR8/SVneo cells (PM2.5 concentration: 0. 50 μg/mL, 100 μg/mL, 200 μg/mL) (Figure 5C-GCLC). The expression of GCLC expression in the PM2.5-treated HTR8/SVneo cells (PM2.5 concentration: 0. 50 μg/mL, 100 μg/mL, 200 μg/mL) (Figure 5C-SOD-1). The expression of SOD-1 expression in the PM2.5-treated HTR8/SVneo cells (PM2.5 concentration: 0. 50 μg/mL, 100 μg/mL, 200 μg/mL) (Figure 5C-β-actin). The expression of β-actin expression in the PM2.5-treated HTR8/SVneo cells (PM2.5 concentration: 0. 50 μg/mL, 100 μg/mL, 200 μg/mL) (Figure 5K-Mito-Cyt-c). The expression of Cyt-c expression in mitochondria after treated with different concentration of PM2.5 (0, 50 μg/mL, 100 μg/mL, 200 μg/mL) (Figure 5K-Mito-VDAC-1). The expression of VDAC-1 expression in mitochondria after treated with different concentration of PM2.5 (0, 50 μg/mL, 100 μg/mL, 200 μg/mL) (Figure 5K-Cyto-Cyt-c). The expression of Cyt-c expression in cytoplasm after treated with different co [file elife-85944-fig5-data1.zip › Figure 5-source data 1/Figure5C-NQO-1.TIF]

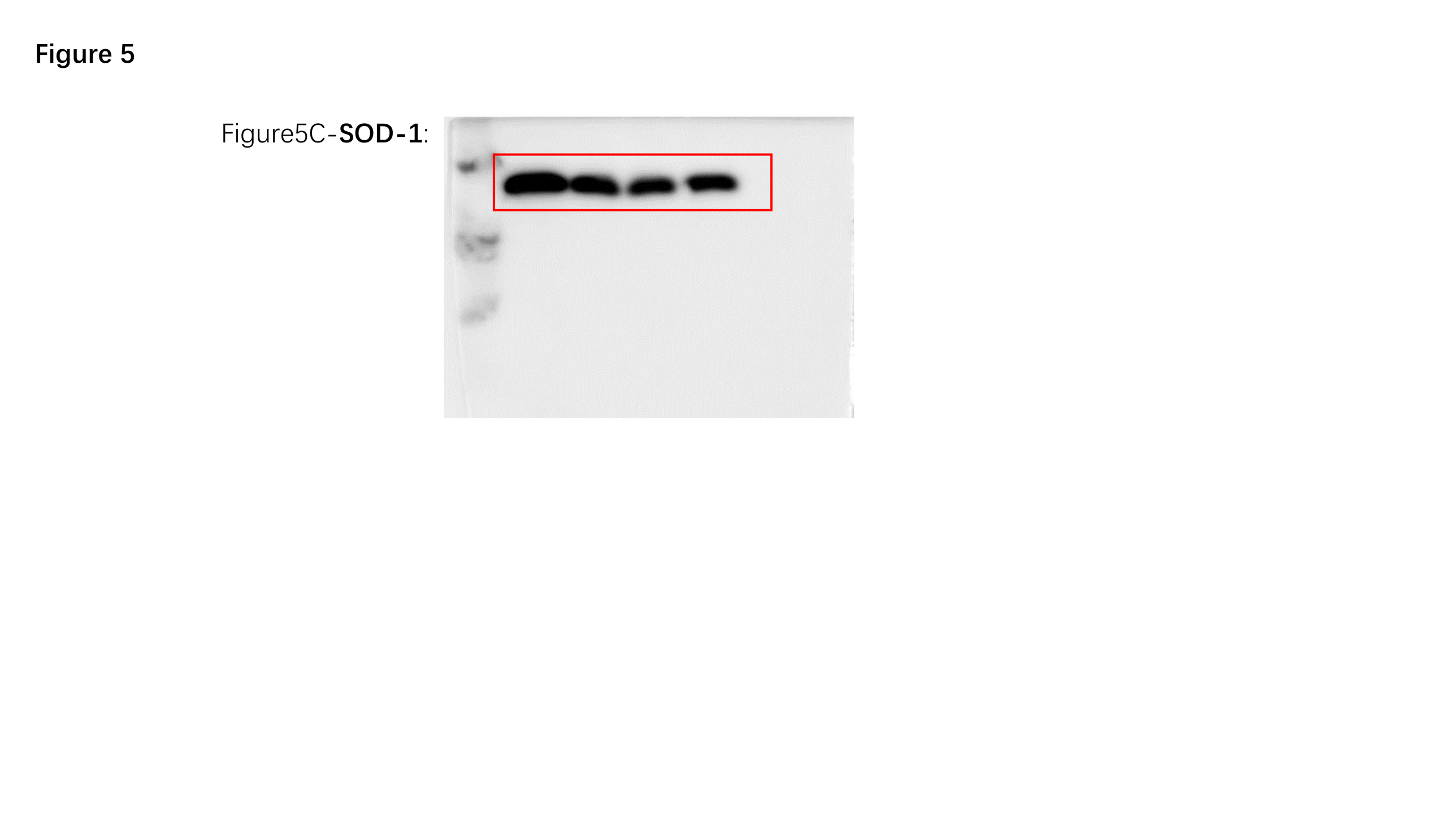

Supplement: Figure 5—source data 1. — (Figure 5B-HO-1) The expression of HO-1 expression in the placental tissue of Wild Type (The first three columns) and PM2.5-treated mice (the last three columns) (Figure 5B-NQO-1). The expression of NQO-1 expression in the placental tissue of Wild Type (The first three columns) and PM2.5-treated mice (the last three columns) (Figure 5B-GCLC). The expression of GCLC expression in the placental tissue of Wild Type (The first three columns) and PM2.5-treated mice (the last three columns) (Figure 5B-SOD-1). The expression of SOD-1 expression in the placental tissue of Wild Type (The first three columns) and PM2.5-treated mice (the last three columns) (Figure 5B-β-actin). The expression of β-actin expression in the placental tissue of Wild Type (The first three columns) and PM2.5-treated mice (the last three columns) (Figure 5C-HO-1). The expression of HO-1 expression in the PM2.5-treated HTR8/SVneo cells (PM2.5 concentration: 0. 50 μg/mL, 100 μg/mL, 200 μg/mL) (Figure 5C-NQO-1). The expression of NQO-1 expression in the PM2.5-treated HTR8/SVneo cells (PM2.5 concentration: 0. 50 μg/mL, 100 μg/mL, 200 μg/mL) (Figure 5C-GCLC). The expression of GCLC expression in the PM2.5-treated HTR8/SVneo cells (PM2.5 concentration: 0. 50 μg/mL, 100 μg/mL, 200 μg/mL) (Figure 5C-SOD-1). The expression of SOD-1 expression in the PM2.5-treated HTR8/SVneo cells (PM2.5 concentration: 0. 50 μg/mL, 100 μg/mL, 200 μg/mL) (Figure 5C-β-actin). The expression of β-actin expression in the PM2.5-treated HTR8/SVneo cells (PM2.5 concentration: 0. 50 μg/mL, 100 μg/mL, 200 μg/mL) (Figure 5K-Mito-Cyt-c). The expression of Cyt-c expression in mitochondria after treated with different concentration of PM2.5 (0, 50 μg/mL, 100 μg/mL, 200 μg/mL) (Figure 5K-Mito-VDAC-1). The expression of VDAC-1 expression in mitochondria after treated with different concentration of PM2.5 (0, 50 μg/mL, 100 μg/mL, 200 μg/mL) (Figure 5K-Cyto-Cyt-c). The expression of Cyt-c expression in cytoplasm after treated with different co [file elife-85944-fig5-data1.zip › Figure 5-source data 1/Figure5C-SOD-1.TIF]

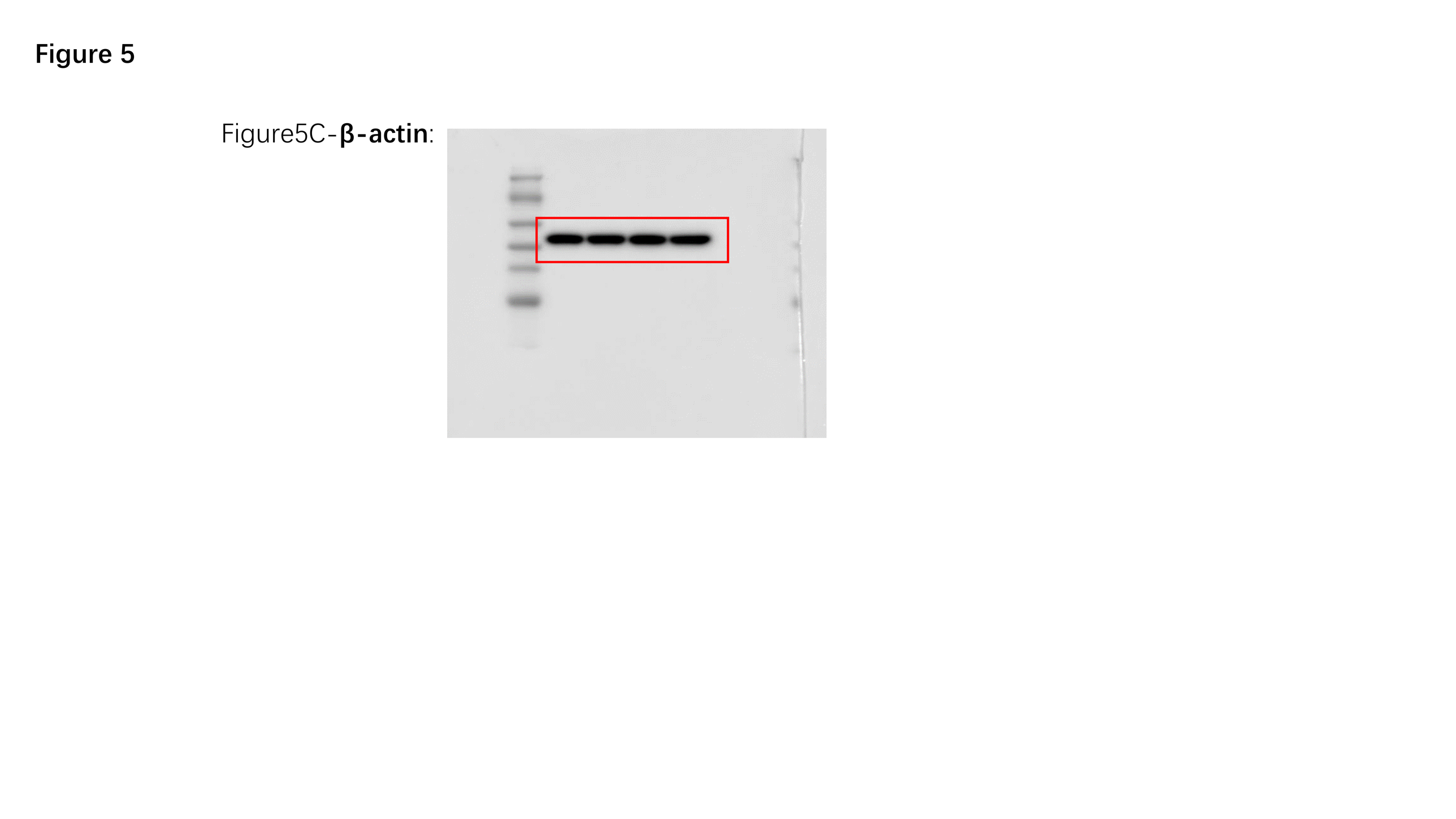

Supplement: Figure 5—source data 1. — (Figure 5B-HO-1) The expression of HO-1 expression in the placental tissue of Wild Type (The first three columns) and PM2.5-treated mice (the last three columns) (Figure 5B-NQO-1). The expression of NQO-1 expression in the placental tissue of Wild Type (The first three columns) and PM2.5-treated mice (the last three columns) (Figure 5B-GCLC). The expression of GCLC expression in the placental tissue of Wild Type (The first three columns) and PM2.5-treated mice (the last three columns) (Figure 5B-SOD-1). The expression of SOD-1 expression in the placental tissue of Wild Type (The first three columns) and PM2.5-treated mice (the last three columns) (Figure 5B-β-actin). The expression of β-actin expression in the placental tissue of Wild Type (The first three columns) and PM2.5-treated mice (the last three columns) (Figure 5C-HO-1). The expression of HO-1 expression in the PM2.5-treated HTR8/SVneo cells (PM2.5 concentration: 0. 50 μg/mL, 100 μg/mL, 200 μg/mL) (Figure 5C-NQO-1). The expression of NQO-1 expression in the PM2.5-treated HTR8/SVneo cells (PM2.5 concentration: 0. 50 μg/mL, 100 μg/mL, 200 μg/mL) (Figure 5C-GCLC). The expression of GCLC expression in the PM2.5-treated HTR8/SVneo cells (PM2.5 concentration: 0. 50 μg/mL, 100 μg/mL, 200 μg/mL) (Figure 5C-SOD-1). The expression of SOD-1 expression in the PM2.5-treated HTR8/SVneo cells (PM2.5 concentration: 0. 50 μg/mL, 100 μg/mL, 200 μg/mL) (Figure 5C-β-actin). The expression of β-actin expression in the PM2.5-treated HTR8/SVneo cells (PM2.5 concentration: 0. 50 μg/mL, 100 μg/mL, 200 μg/mL) (Figure 5K-Mito-Cyt-c). The expression of Cyt-c expression in mitochondria after treated with different concentration of PM2.5 (0, 50 μg/mL, 100 μg/mL, 200 μg/mL) (Figure 5K-Mito-VDAC-1). The expression of VDAC-1 expression in mitochondria after treated with different concentration of PM2.5 (0, 50 μg/mL, 100 μg/mL, 200 μg/mL) (Figure 5K-Cyto-Cyt-c). The expression of Cyt-c expression in cytoplasm after treated with different co [file elife-85944-fig5-data1.zip › Figure 5-source data 1/Figure5C-a┬-actin.TIF]

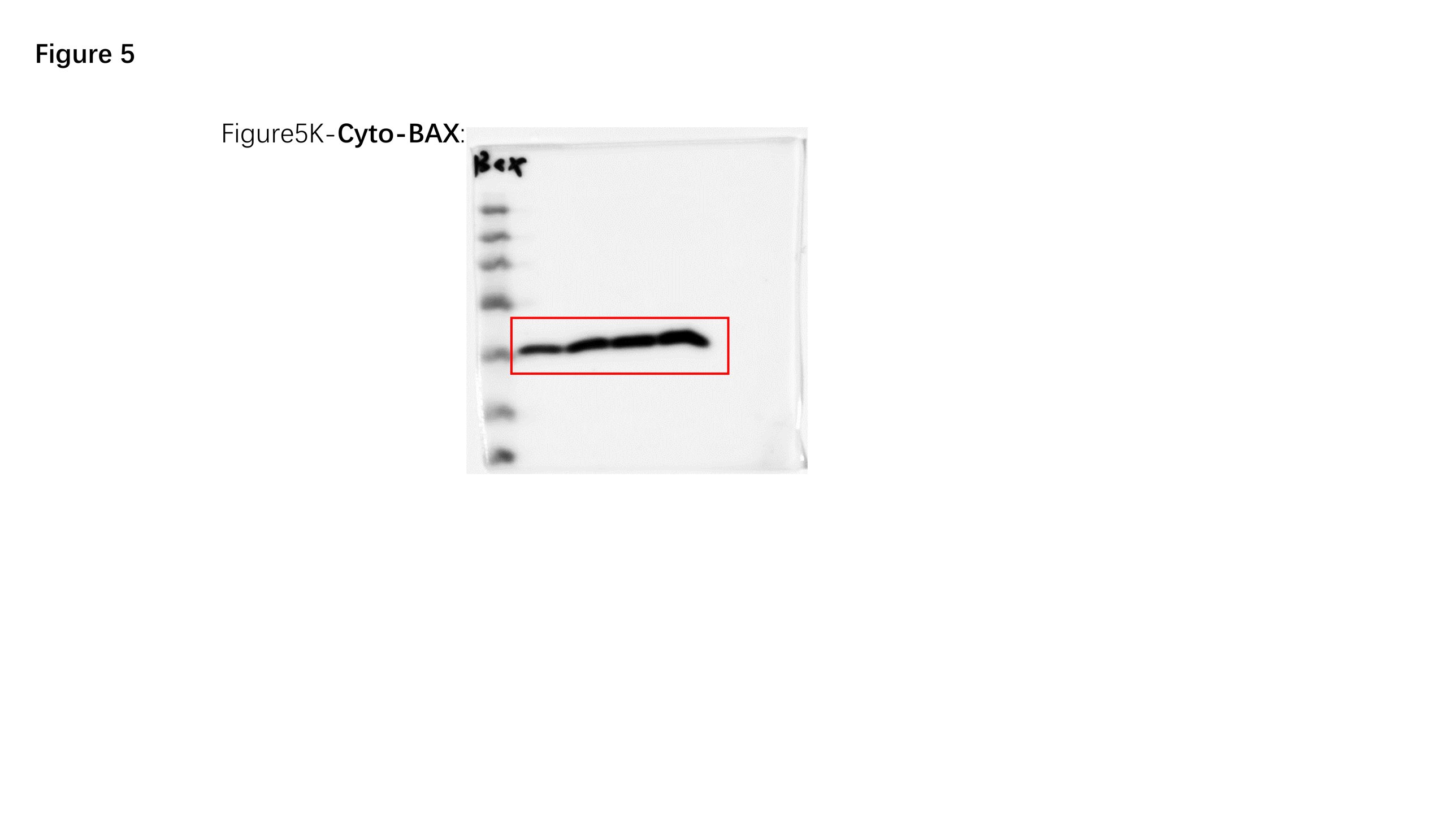

Supplement: Figure 5—source data 1. — (Figure 5B-HO-1) The expression of HO-1 expression in the placental tissue of Wild Type (The first three columns) and PM2.5-treated mice (the last three columns) (Figure 5B-NQO-1). The expression of NQO-1 expression in the placental tissue of Wild Type (The first three columns) and PM2.5-treated mice (the last three columns) (Figure 5B-GCLC). The expression of GCLC expression in the placental tissue of Wild Type (The first three columns) and PM2.5-treated mice (the last three columns) (Figure 5B-SOD-1). The expression of SOD-1 expression in the placental tissue of Wild Type (The first three columns) and PM2.5-treated mice (the last three columns) (Figure 5B-β-actin). The expression of β-actin expression in the placental tissue of Wild Type (The first three columns) and PM2.5-treated mice (the last three columns) (Figure 5C-HO-1). The expression of HO-1 expression in the PM2.5-treated HTR8/SVneo cells (PM2.5 concentration: 0. 50 μg/mL, 100 μg/mL, 200 μg/mL) (Figure 5C-NQO-1). The expression of NQO-1 expression in the PM2.5-treated HTR8/SVneo cells (PM2.5 concentration: 0. 50 μg/mL, 100 μg/mL, 200 μg/mL) (Figure 5C-GCLC). The expression of GCLC expression in the PM2.5-treated HTR8/SVneo cells (PM2.5 concentration: 0. 50 μg/mL, 100 μg/mL, 200 μg/mL) (Figure 5C-SOD-1). The expression of SOD-1 expression in the PM2.5-treated HTR8/SVneo cells (PM2.5 concentration: 0. 50 μg/mL, 100 μg/mL, 200 μg/mL) (Figure 5C-β-actin). The expression of β-actin expression in the PM2.5-treated HTR8/SVneo cells (PM2.5 concentration: 0. 50 μg/mL, 100 μg/mL, 200 μg/mL) (Figure 5K-Mito-Cyt-c). The expression of Cyt-c expression in mitochondria after treated with different concentration of PM2.5 (0, 50 μg/mL, 100 μg/mL, 200 μg/mL) (Figure 5K-Mito-VDAC-1). The expression of VDAC-1 expression in mitochondria after treated with different concentration of PM2.5 (0, 50 μg/mL, 100 μg/mL, 200 μg/mL) (Figure 5K-Cyto-Cyt-c). The expression of Cyt-c expression in cytoplasm after treated with different co [file elife-85944-fig5-data1.zip › Figure 5-source data 1/Figure5K-Cyto-BAX.TIF]

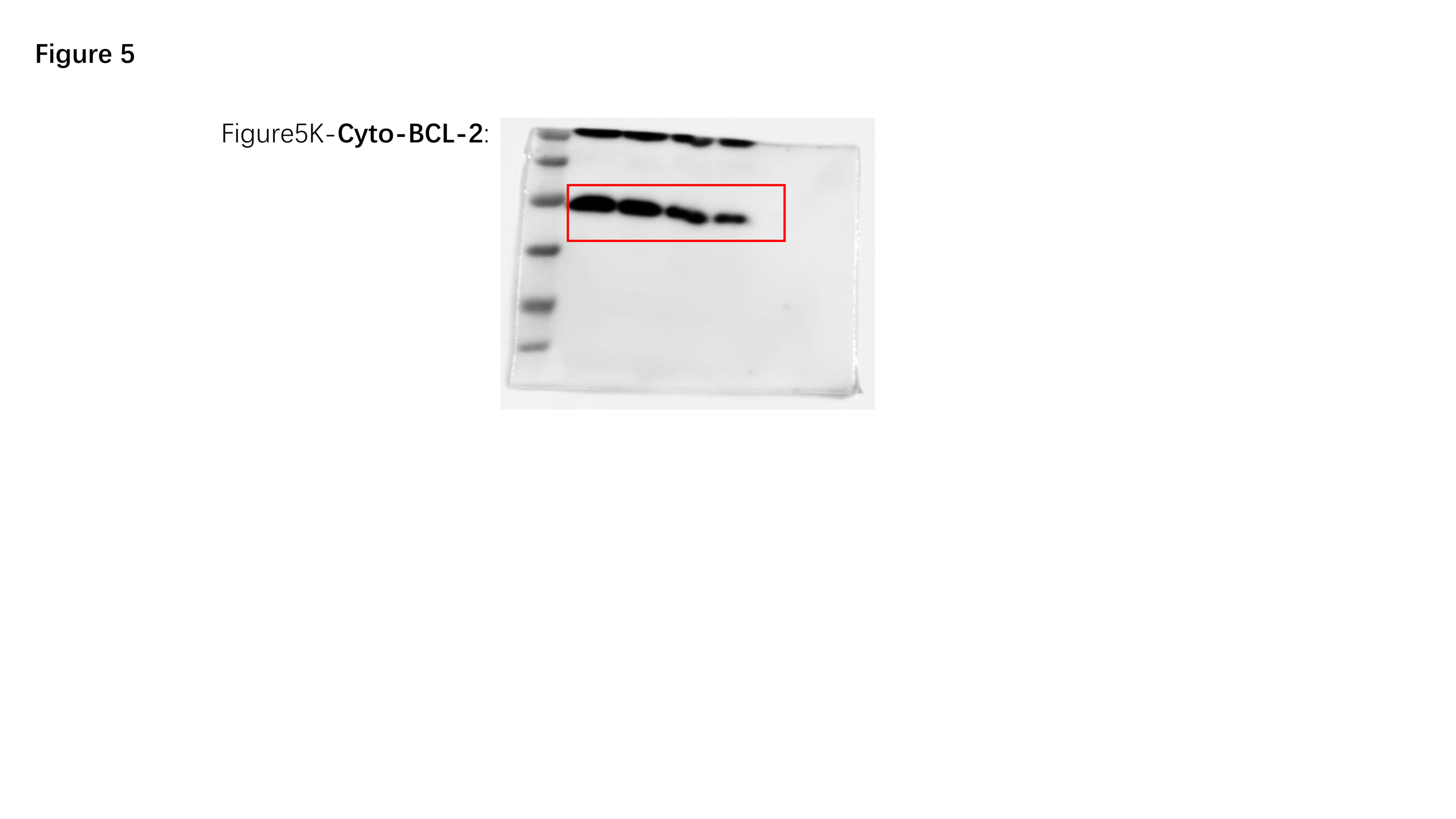

Supplement: Figure 5—source data 1. — (Figure 5B-HO-1) The expression of HO-1 expression in the placental tissue of Wild Type (The first three columns) and PM2.5-treated mice (the last three columns) (Figure 5B-NQO-1). The expression of NQO-1 expression in the placental tissue of Wild Type (The first three columns) and PM2.5-treated mice (the last three columns) (Figure 5B-GCLC). The expression of GCLC expression in the placental tissue of Wild Type (The first three columns) and PM2.5-treated mice (the last three columns) (Figure 5B-SOD-1). The expression of SOD-1 expression in the placental tissue of Wild Type (The first three columns) and PM2.5-treated mice (the last three columns) (Figure 5B-β-actin). The expression of β-actin expression in the placental tissue of Wild Type (The first three columns) and PM2.5-treated mice (the last three columns) (Figure 5C-HO-1). The expression of HO-1 expression in the PM2.5-treated HTR8/SVneo cells (PM2.5 concentration: 0. 50 μg/mL, 100 μg/mL, 200 μg/mL) (Figure 5C-NQO-1). The expression of NQO-1 expression in the PM2.5-treated HTR8/SVneo cells (PM2.5 concentration: 0. 50 μg/mL, 100 μg/mL, 200 μg/mL) (Figure 5C-GCLC). The expression of GCLC expression in the PM2.5-treated HTR8/SVneo cells (PM2.5 concentration: 0. 50 μg/mL, 100 μg/mL, 200 μg/mL) (Figure 5C-SOD-1). The expression of SOD-1 expression in the PM2.5-treated HTR8/SVneo cells (PM2.5 concentration: 0. 50 μg/mL, 100 μg/mL, 200 μg/mL) (Figure 5C-β-actin). The expression of β-actin expression in the PM2.5-treated HTR8/SVneo cells (PM2.5 concentration: 0. 50 μg/mL, 100 μg/mL, 200 μg/mL) (Figure 5K-Mito-Cyt-c). The expression of Cyt-c expression in mitochondria after treated with different concentration of PM2.5 (0, 50 μg/mL, 100 μg/mL, 200 μg/mL) (Figure 5K-Mito-VDAC-1). The expression of VDAC-1 expression in mitochondria after treated with different concentration of PM2.5 (0, 50 μg/mL, 100 μg/mL, 200 μg/mL) (Figure 5K-Cyto-Cyt-c). The expression of Cyt-c expression in cytoplasm after treated with different co [file elife-85944-fig5-data1.zip › Figure 5-source data 1/Figure5K-Cyto-BCL-2.TIF]

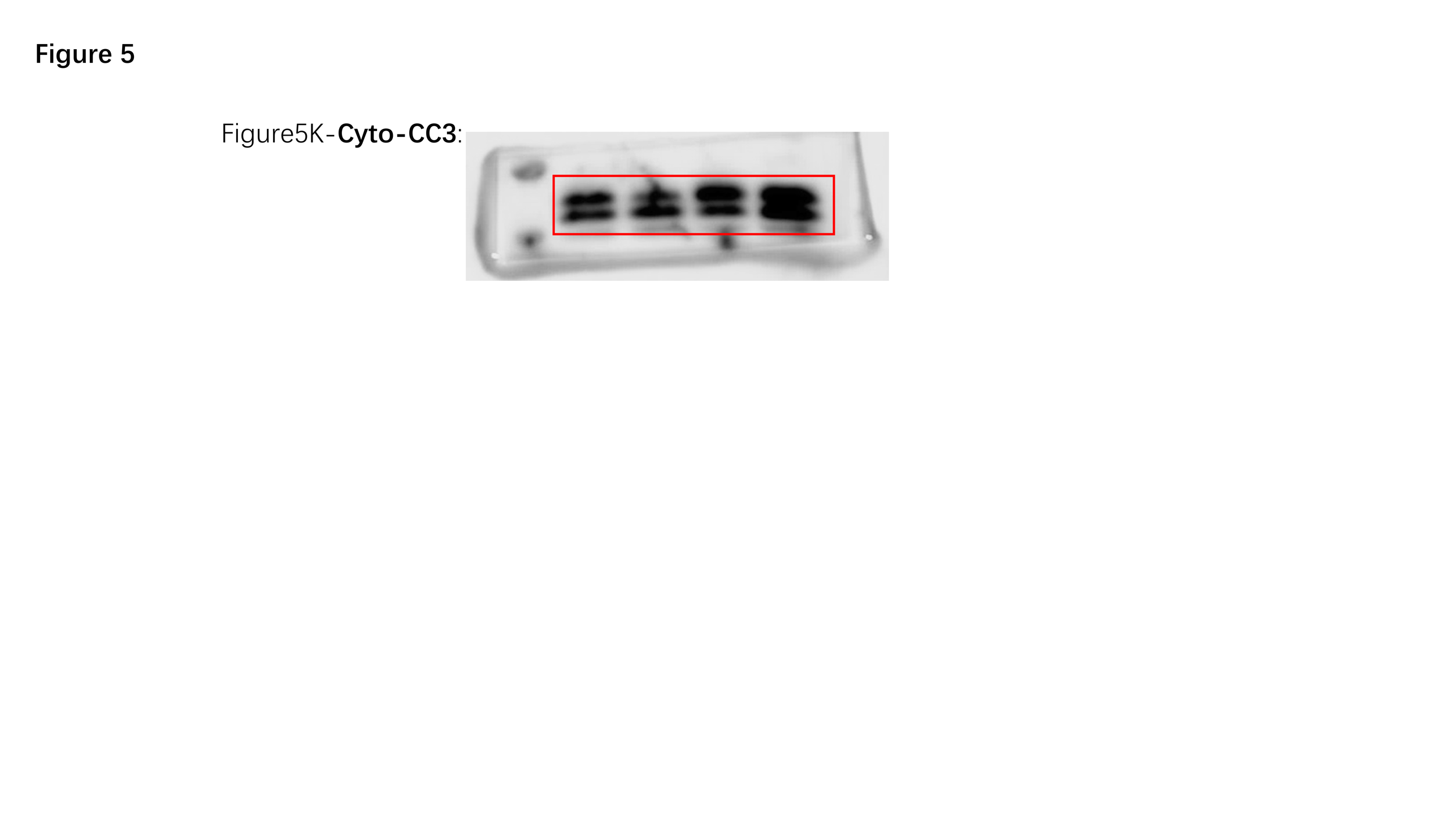

Supplement: Figure 5—source data 1. — (Figure 5B-HO-1) The expression of HO-1 expression in the placental tissue of Wild Type (The first three columns) and PM2.5-treated mice (the last three columns) (Figure 5B-NQO-1). The expression of NQO-1 expression in the placental tissue of Wild Type (The first three columns) and PM2.5-treated mice (the last three columns) (Figure 5B-GCLC). The expression of GCLC expression in the placental tissue of Wild Type (The first three columns) and PM2.5-treated mice (the last three columns) (Figure 5B-SOD-1). The expression of SOD-1 expression in the placental tissue of Wild Type (The first three columns) and PM2.5-treated mice (the last three columns) (Figure 5B-β-actin). The expression of β-actin expression in the placental tissue of Wild Type (The first three columns) and PM2.5-treated mice (the last three columns) (Figure 5C-HO-1). The expression of HO-1 expression in the PM2.5-treated HTR8/SVneo cells (PM2.5 concentration: 0. 50 μg/mL, 100 μg/mL, 200 μg/mL) (Figure 5C-NQO-1). The expression of NQO-1 expression in the PM2.5-treated HTR8/SVneo cells (PM2.5 concentration: 0. 50 μg/mL, 100 μg/mL, 200 μg/mL) (Figure 5C-GCLC). The expression of GCLC expression in the PM2.5-treated HTR8/SVneo cells (PM2.5 concentration: 0. 50 μg/mL, 100 μg/mL, 200 μg/mL) (Figure 5C-SOD-1). The expression of SOD-1 expression in the PM2.5-treated HTR8/SVneo cells (PM2.5 concentration: 0. 50 μg/mL, 100 μg/mL, 200 μg/mL) (Figure 5C-β-actin). The expression of β-actin expression in the PM2.5-treated HTR8/SVneo cells (PM2.5 concentration: 0. 50 μg/mL, 100 μg/mL, 200 μg/mL) (Figure 5K-Mito-Cyt-c). The expression of Cyt-c expression in mitochondria after treated with different concentration of PM2.5 (0, 50 μg/mL, 100 μg/mL, 200 μg/mL) (Figure 5K-Mito-VDAC-1). The expression of VDAC-1 expression in mitochondria after treated with different concentration of PM2.5 (0, 50 μg/mL, 100 μg/mL, 200 μg/mL) (Figure 5K-Cyto-Cyt-c). The expression of Cyt-c expression in cytoplasm after treated with different co [file elife-85944-fig5-data1.zip › Figure 5-source data 1/Figure5K-Cyto-CC3.TIF]

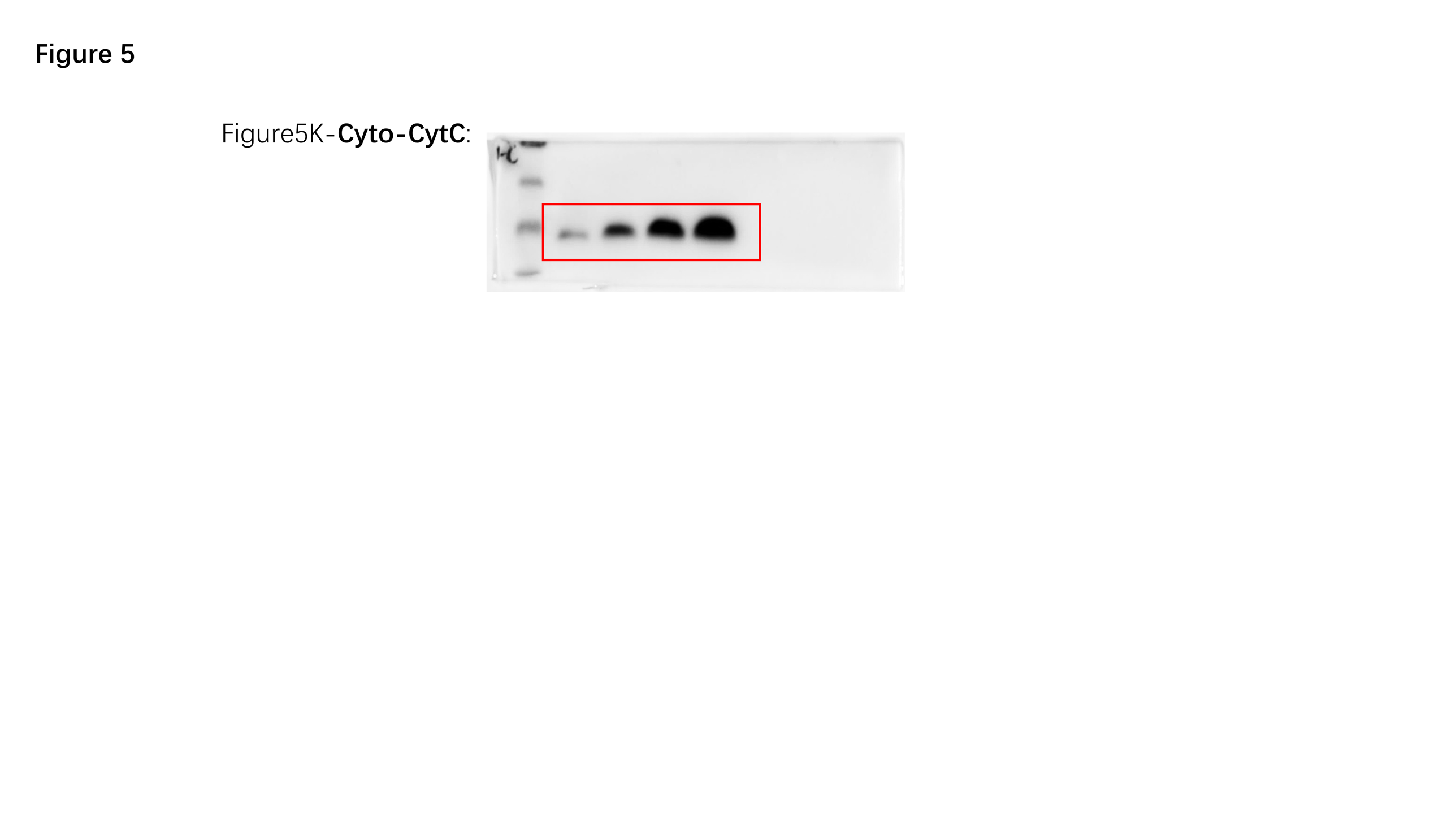

Supplement: Figure 5—source data 1. — (Figure 5B-HO-1) The expression of HO-1 expression in the placental tissue of Wild Type (The first three columns) and PM2.5-treated mice (the last three columns) (Figure 5B-NQO-1). The expression of NQO-1 expression in the placental tissue of Wild Type (The first three columns) and PM2.5-treated mice (the last three columns) (Figure 5B-GCLC). The expression of GCLC expression in the placental tissue of Wild Type (The first three columns) and PM2.5-treated mice (the last three columns) (Figure 5B-SOD-1). The expression of SOD-1 expression in the placental tissue of Wild Type (The first three columns) and PM2.5-treated mice (the last three columns) (Figure 5B-β-actin). The expression of β-actin expression in the placental tissue of Wild Type (The first three columns) and PM2.5-treated mice (the last three columns) (Figure 5C-HO-1). The expression of HO-1 expression in the PM2.5-treated HTR8/SVneo cells (PM2.5 concentration: 0. 50 μg/mL, 100 μg/mL, 200 μg/mL) (Figure 5C-NQO-1). The expression of NQO-1 expression in the PM2.5-treated HTR8/SVneo cells (PM2.5 concentration: 0. 50 μg/mL, 100 μg/mL, 200 μg/mL) (Figure 5C-GCLC). The expression of GCLC expression in the PM2.5-treated HTR8/SVneo cells (PM2.5 concentration: 0. 50 μg/mL, 100 μg/mL, 200 μg/mL) (Figure 5C-SOD-1). The expression of SOD-1 expression in the PM2.5-treated HTR8/SVneo cells (PM2.5 concentration: 0. 50 μg/mL, 100 μg/mL, 200 μg/mL) (Figure 5C-β-actin). The expression of β-actin expression in the PM2.5-treated HTR8/SVneo cells (PM2.5 concentration: 0. 50 μg/mL, 100 μg/mL, 200 μg/mL) (Figure 5K-Mito-Cyt-c). The expression of Cyt-c expression in mitochondria after treated with different concentration of PM2.5 (0, 50 μg/mL, 100 μg/mL, 200 μg/mL) (Figure 5K-Mito-VDAC-1). The expression of VDAC-1 expression in mitochondria after treated with different concentration of PM2.5 (0, 50 μg/mL, 100 μg/mL, 200 μg/mL) (Figure 5K-Cyto-Cyt-c). The expression of Cyt-c expression in cytoplasm after treated with different co [file elife-85944-fig5-data1.zip › Figure 5-source data 1/Figure5K-Cyto-CytC.TIF]

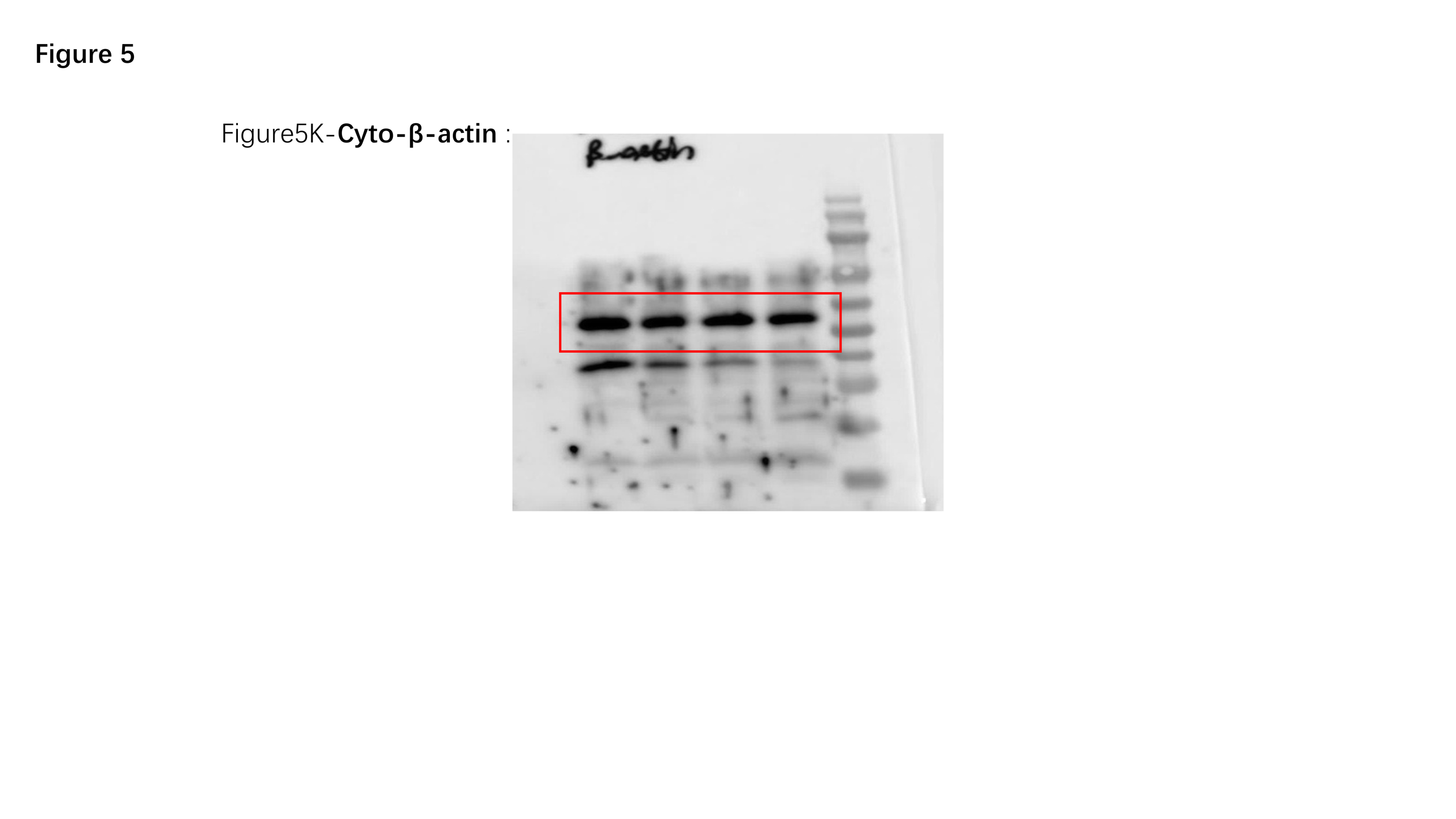

Supplement: Figure 5—source data 1. — (Figure 5B-HO-1) The expression of HO-1 expression in the placental tissue of Wild Type (The first three columns) and PM2.5-treated mice (the last three columns) (Figure 5B-NQO-1). The expression of NQO-1 expression in the placental tissue of Wild Type (The first three columns) and PM2.5-treated mice (the last three columns) (Figure 5B-GCLC). The expression of GCLC expression in the placental tissue of Wild Type (The first three columns) and PM2.5-treated mice (the last three columns) (Figure 5B-SOD-1). The expression of SOD-1 expression in the placental tissue of Wild Type (The first three columns) and PM2.5-treated mice (the last three columns) (Figure 5B-β-actin). The expression of β-actin expression in the placental tissue of Wild Type (The first three columns) and PM2.5-treated mice (the last three columns) (Figure 5C-HO-1). The expression of HO-1 expression in the PM2.5-treated HTR8/SVneo cells (PM2.5 concentration: 0. 50 μg/mL, 100 μg/mL, 200 μg/mL) (Figure 5C-NQO-1). The expression of NQO-1 expression in the PM2.5-treated HTR8/SVneo cells (PM2.5 concentration: 0. 50 μg/mL, 100 μg/mL, 200 μg/mL) (Figure 5C-GCLC). The expression of GCLC expression in the PM2.5-treated HTR8/SVneo cells (PM2.5 concentration: 0. 50 μg/mL, 100 μg/mL, 200 μg/mL) (Figure 5C-SOD-1). The expression of SOD-1 expression in the PM2.5-treated HTR8/SVneo cells (PM2.5 concentration: 0. 50 μg/mL, 100 μg/mL, 200 μg/mL) (Figure 5C-β-actin). The expression of β-actin expression in the PM2.5-treated HTR8/SVneo cells (PM2.5 concentration: 0. 50 μg/mL, 100 μg/mL, 200 μg/mL) (Figure 5K-Mito-Cyt-c). The expression of Cyt-c expression in mitochondria after treated with different concentration of PM2.5 (0, 50 μg/mL, 100 μg/mL, 200 μg/mL) (Figure 5K-Mito-VDAC-1). The expression of VDAC-1 expression in mitochondria after treated with different concentration of PM2.5 (0, 50 μg/mL, 100 μg/mL, 200 μg/mL) (Figure 5K-Cyto-Cyt-c). The expression of Cyt-c expression in cytoplasm after treated with different co [file elife-85944-fig5-data1.zip › Figure 5-source data 1/Figure5K-Cyto-a┬-actin .TIF]

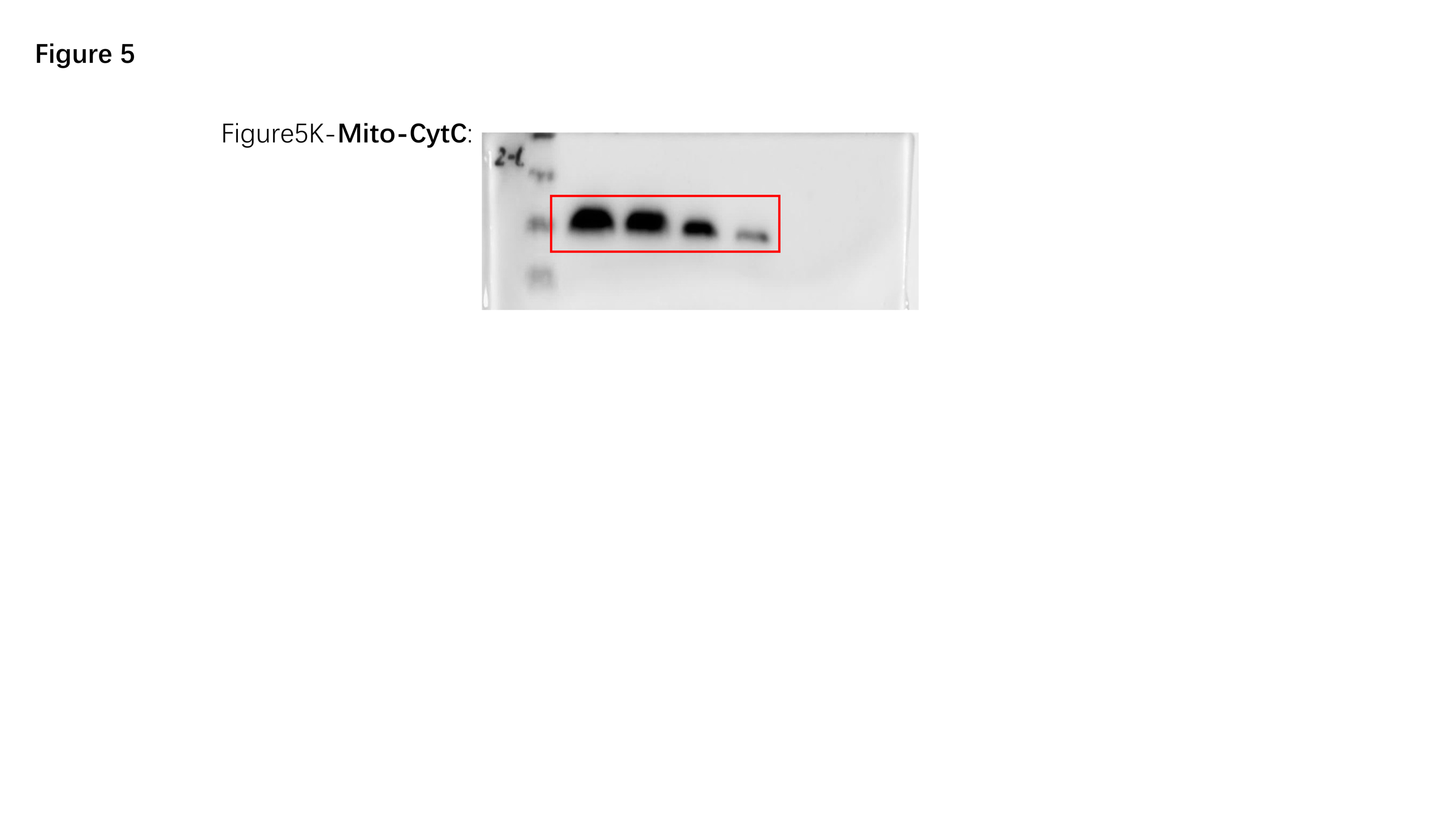

Supplement: Figure 5—source data 1. — (Figure 5B-HO-1) The expression of HO-1 expression in the placental tissue of Wild Type (The first three columns) and PM2.5-treated mice (the last three columns) (Figure 5B-NQO-1). The expression of NQO-1 expression in the placental tissue of Wild Type (The first three columns) and PM2.5-treated mice (the last three columns) (Figure 5B-GCLC). The expression of GCLC expression in the placental tissue of Wild Type (The first three columns) and PM2.5-treated mice (the last three columns) (Figure 5B-SOD-1). The expression of SOD-1 expression in the placental tissue of Wild Type (The first three columns) and PM2.5-treated mice (the last three columns) (Figure 5B-β-actin). The expression of β-actin expression in the placental tissue of Wild Type (The first three columns) and PM2.5-treated mice (the last three columns) (Figure 5C-HO-1). The expression of HO-1 expression in the PM2.5-treated HTR8/SVneo cells (PM2.5 concentration: 0. 50 μg/mL, 100 μg/mL, 200 μg/mL) (Figure 5C-NQO-1). The expression of NQO-1 expression in the PM2.5-treated HTR8/SVneo cells (PM2.5 concentration: 0. 50 μg/mL, 100 μg/mL, 200 μg/mL) (Figure 5C-GCLC). The expression of GCLC expression in the PM2.5-treated HTR8/SVneo cells (PM2.5 concentration: 0. 50 μg/mL, 100 μg/mL, 200 μg/mL) (Figure 5C-SOD-1). The expression of SOD-1 expression in the PM2.5-treated HTR8/SVneo cells (PM2.5 concentration: 0. 50 μg/mL, 100 μg/mL, 200 μg/mL) (Figure 5C-β-actin). The expression of β-actin expression in the PM2.5-treated HTR8/SVneo cells (PM2.5 concentration: 0. 50 μg/mL, 100 μg/mL, 200 μg/mL) (Figure 5K-Mito-Cyt-c). The expression of Cyt-c expression in mitochondria after treated with different concentration of PM2.5 (0, 50 μg/mL, 100 μg/mL, 200 μg/mL) (Figure 5K-Mito-VDAC-1). The expression of VDAC-1 expression in mitochondria after treated with different concentration of PM2.5 (0, 50 μg/mL, 100 μg/mL, 200 μg/mL) (Figure 5K-Cyto-Cyt-c). The expression of Cyt-c expression in cytoplasm after treated with different co [file elife-85944-fig5-data1.zip › Figure 5-source data 1/Figure5K-Mito-CytC.TIF]

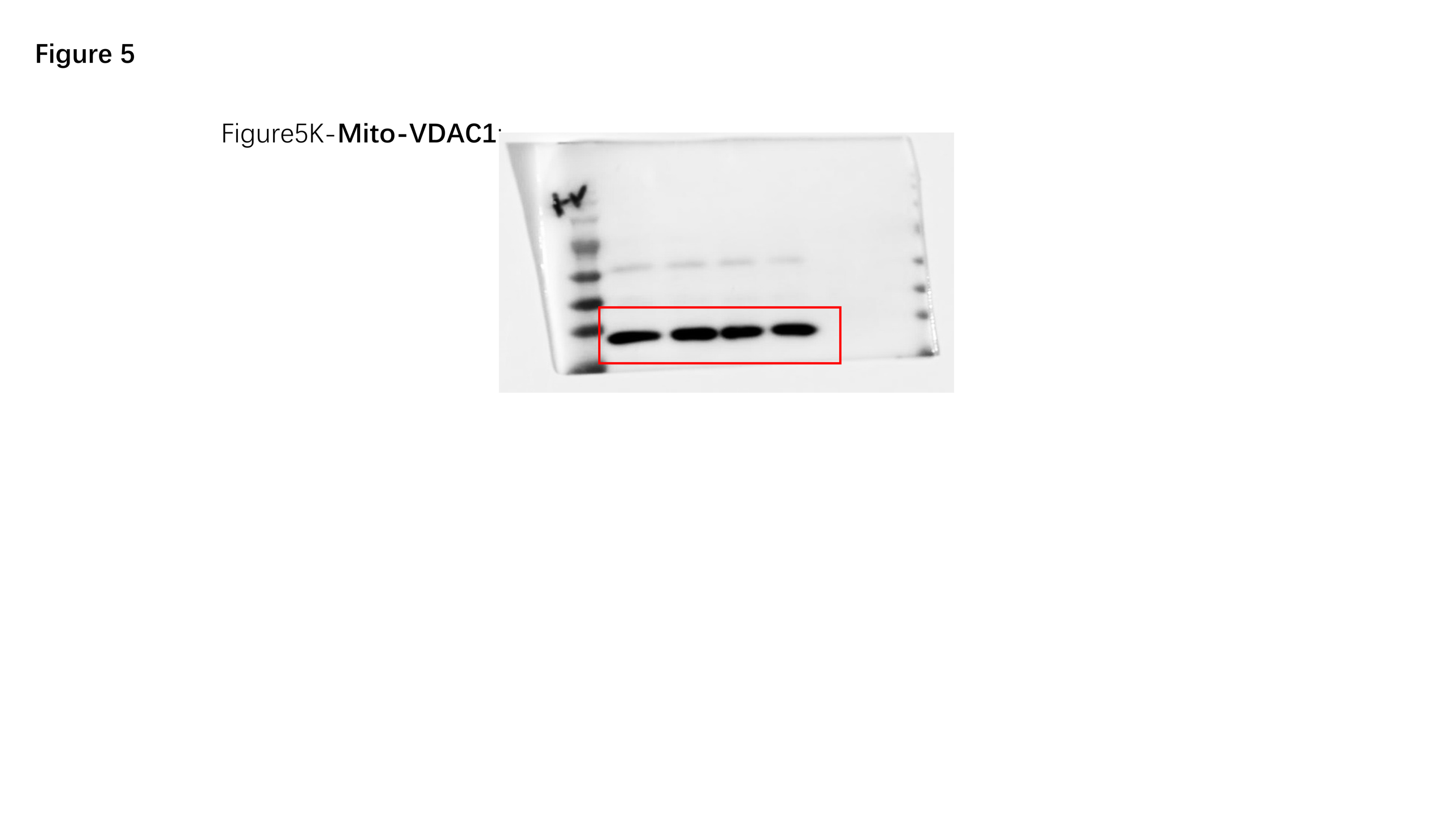

Supplement: Figure 5—source data 1. — (Figure 5B-HO-1) The expression of HO-1 expression in the placental tissue of Wild Type (The first three columns) and PM2.5-treated mice (the last three columns) (Figure 5B-NQO-1). The expression of NQO-1 expression in the placental tissue of Wild Type (The first three columns) and PM2.5-treated mice (the last three columns) (Figure 5B-GCLC). The expression of GCLC expression in the placental tissue of Wild Type (The first three columns) and PM2.5-treated mice (the last three columns) (Figure 5B-SOD-1). The expression of SOD-1 expression in the placental tissue of Wild Type (The first three columns) and PM2.5-treated mice (the last three columns) (Figure 5B-β-actin). The expression of β-actin expression in the placental tissue of Wild Type (The first three columns) and PM2.5-treated mice (the last three columns) (Figure 5C-HO-1). The expression of HO-1 expression in the PM2.5-treated HTR8/SVneo cells (PM2.5 concentration: 0. 50 μg/mL, 100 μg/mL, 200 μg/mL) (Figure 5C-NQO-1). The expression of NQO-1 expression in the PM2.5-treated HTR8/SVneo cells (PM2.5 concentration: 0. 50 μg/mL, 100 μg/mL, 200 μg/mL) (Figure 5C-GCLC). The expression of GCLC expression in the PM2.5-treated HTR8/SVneo cells (PM2.5 concentration: 0. 50 μg/mL, 100 μg/mL, 200 μg/mL) (Figure 5C-SOD-1). The expression of SOD-1 expression in the PM2.5-treated HTR8/SVneo cells (PM2.5 concentration: 0. 50 μg/mL, 100 μg/mL, 200 μg/mL) (Figure 5C-β-actin). The expression of β-actin expression in the PM2.5-treated HTR8/SVneo cells (PM2.5 concentration: 0. 50 μg/mL, 100 μg/mL, 200 μg/mL) (Figure 5K-Mito-Cyt-c). The expression of Cyt-c expression in mitochondria after treated with different concentration of PM2.5 (0, 50 μg/mL, 100 μg/mL, 200 μg/mL) (Figure 5K-Mito-VDAC-1). The expression of VDAC-1 expression in mitochondria after treated with different concentration of PM2.5 (0, 50 μg/mL, 100 μg/mL, 200 μg/mL) (Figure 5K-Cyto-Cyt-c). The expression of Cyt-c expression in cytoplasm after treated with different co [file elife-85944-fig5-data1.zip › Figure 5-source data 1/Figure5K-Mito-VDAC1.TIF]

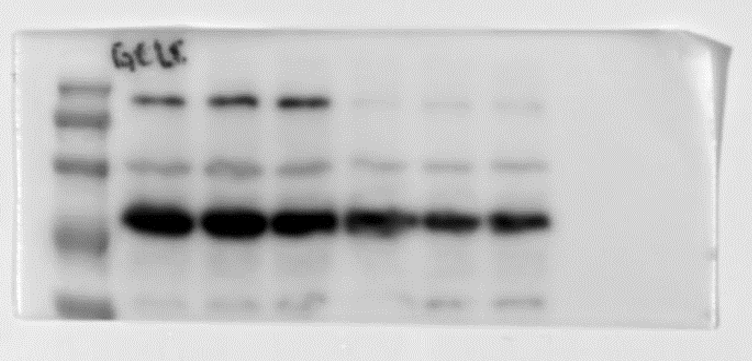

Supplement: Figure 5—source data 2. — (Figure 5B-HO-1) The expression of HO-1 expression in the placental tissue of Wild Type (The first three columns) and PM2.5-treated mice (the last three columns). (Figure 5B-NQO-1) The expression of NQO-1 expression in the placental tissue of Wild Type (The first three columns) and PM2.5-treated mice (the last three columns). (Figure 5B-GCLC) The expression of GCLC expression in the placental tissue of Wild Type (The first three columns) and PM2.5-treated mice (the last three columns). (Figure 5B-SOD-1) The expression of SOD-1 expression in the placental tissue of Wild Type (The first three columns) and PM2.5-treated mice (the last three columns). (Figure 5B-β-actin) The expression of β-actin expression in the placental tissue of Wild Type (The first three columns) and PM2.5-treated mice (the last three columns). (Figure 5C-HO-1) The expression of HO-1 expression in the PM2.5-treated HTR8/SVneo cells (PM2.5 concentration: 0. 50 μg/mL, 100 μg/mL, 200 μg/mL). (Figure 5C-NQO-1) The expression of NQO-1 expression in the PM2.5-treated HTR8/SVneo cells (PM2.5 concentration: 0. 50 μg/mL, 100 μg/mL, 200 μg/mL). (Figure 5C-GCLC) The expression of GCLC expression in the PM2.5-treated HTR8/SVneo cells (PM2.5 concentration: 0. 50 μg/mL, 100 μg/mL, 200 μg/mL). (Figure 5C-SOD-1) The expression of SOD-1 expression in the PM2.5-treated HTR8/SVneo cells (PM2.5 concentration: 0. 50 μg/mL, 100 μg/mL, 200 μg/mL). (Figure 5C-β-actin) The expression of β-actin expression in the PM2.5-treated HTR8/SVneo cells (PM2.5 concentration: 0. 50 μg/mL, 100 μg/mL, 200 μg/mL). (Figure 5K-Mito-Cyt-c) The expression of Cyt-c expression in mitochondria after treated with different concentration of PM2.5 (0, 50 μg/mL, 100 μg/mL, 200 μg/mL). (Figure 5K-Mito-VDAC-1) The expression of VDAC-1 expression in mitochondria after treated with different concentration of PM2.5 (0, 50 μg/mL, 100 μg/mL, 200 μg/mL). (Figure 5K-Cyto-Cyt-c) The expression of Cyt-c expression in cytoplasm after treated with different co [file elife-85944-fig5-data2.zip › Figure 5-source data 2/Figure5B-GCLC.tif]

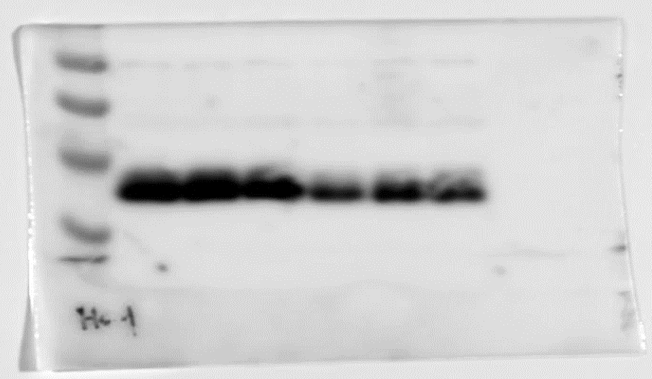

Supplement: Figure 5—source data 2. — (Figure 5B-HO-1) The expression of HO-1 expression in the placental tissue of Wild Type (The first three columns) and PM2.5-treated mice (the last three columns). (Figure 5B-NQO-1) The expression of NQO-1 expression in the placental tissue of Wild Type (The first three columns) and PM2.5-treated mice (the last three columns). (Figure 5B-GCLC) The expression of GCLC expression in the placental tissue of Wild Type (The first three columns) and PM2.5-treated mice (the last three columns). (Figure 5B-SOD-1) The expression of SOD-1 expression in the placental tissue of Wild Type (The first three columns) and PM2.5-treated mice (the last three columns). (Figure 5B-β-actin) The expression of β-actin expression in the placental tissue of Wild Type (The first three columns) and PM2.5-treated mice (the last three columns). (Figure 5C-HO-1) The expression of HO-1 expression in the PM2.5-treated HTR8/SVneo cells (PM2.5 concentration: 0. 50 μg/mL, 100 μg/mL, 200 μg/mL). (Figure 5C-NQO-1) The expression of NQO-1 expression in the PM2.5-treated HTR8/SVneo cells (PM2.5 concentration: 0. 50 μg/mL, 100 μg/mL, 200 μg/mL). (Figure 5C-GCLC) The expression of GCLC expression in the PM2.5-treated HTR8/SVneo cells (PM2.5 concentration: 0. 50 μg/mL, 100 μg/mL, 200 μg/mL). (Figure 5C-SOD-1) The expression of SOD-1 expression in the PM2.5-treated HTR8/SVneo cells (PM2.5 concentration: 0. 50 μg/mL, 100 μg/mL, 200 μg/mL). (Figure 5C-β-actin) The expression of β-actin expression in the PM2.5-treated HTR8/SVneo cells (PM2.5 concentration: 0. 50 μg/mL, 100 μg/mL, 200 μg/mL). (Figure 5K-Mito-Cyt-c) The expression of Cyt-c expression in mitochondria after treated with different concentration of PM2.5 (0, 50 μg/mL, 100 μg/mL, 200 μg/mL). (Figure 5K-Mito-VDAC-1) The expression of VDAC-1 expression in mitochondria after treated with different concentration of PM2.5 (0, 50 μg/mL, 100 μg/mL, 200 μg/mL). (Figure 5K-Cyto-Cyt-c) The expression of Cyt-c expression in cytoplasm after treated with different co [file elife-85944-fig5-data2.zip › Figure 5-source data 2/Figure5B-HO-1.tif]

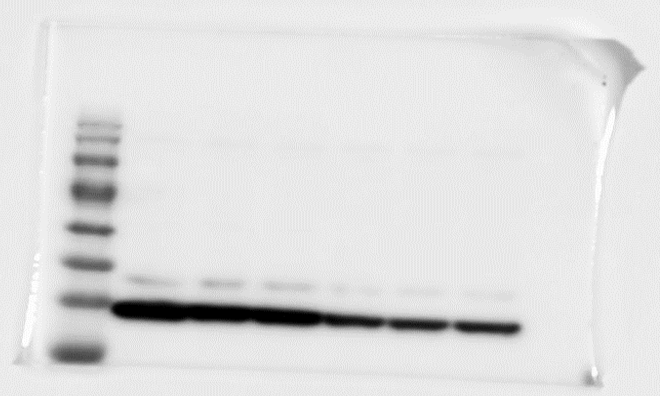

Supplement: Figure 5—source data 2. — (Figure 5B-HO-1) The expression of HO-1 expression in the placental tissue of Wild Type (The first three columns) and PM2.5-treated mice (the last three columns). (Figure 5B-NQO-1) The expression of NQO-1 expression in the placental tissue of Wild Type (The first three columns) and PM2.5-treated mice (the last three columns). (Figure 5B-GCLC) The expression of GCLC expression in the placental tissue of Wild Type (The first three columns) and PM2.5-treated mice (the last three columns). (Figure 5B-SOD-1) The expression of SOD-1 expression in the placental tissue of Wild Type (The first three columns) and PM2.5-treated mice (the last three columns). (Figure 5B-β-actin) The expression of β-actin expression in the placental tissue of Wild Type (The first three columns) and PM2.5-treated mice (the last three columns). (Figure 5C-HO-1) The expression of HO-1 expression in the PM2.5-treated HTR8/SVneo cells (PM2.5 concentration: 0. 50 μg/mL, 100 μg/mL, 200 μg/mL). (Figure 5C-NQO-1) The expression of NQO-1 expression in the PM2.5-treated HTR8/SVneo cells (PM2.5 concentration: 0. 50 μg/mL, 100 μg/mL, 200 μg/mL). (Figure 5C-GCLC) The expression of GCLC expression in the PM2.5-treated HTR8/SVneo cells (PM2.5 concentration: 0. 50 μg/mL, 100 μg/mL, 200 μg/mL). (Figure 5C-SOD-1) The expression of SOD-1 expression in the PM2.5-treated HTR8/SVneo cells (PM2.5 concentration: 0. 50 μg/mL, 100 μg/mL, 200 μg/mL). (Figure 5C-β-actin) The expression of β-actin expression in the PM2.5-treated HTR8/SVneo cells (PM2.5 concentration: 0. 50 μg/mL, 100 μg/mL, 200 μg/mL). (Figure 5K-Mito-Cyt-c) The expression of Cyt-c expression in mitochondria after treated with different concentration of PM2.5 (0, 50 μg/mL, 100 μg/mL, 200 μg/mL). (Figure 5K-Mito-VDAC-1) The expression of VDAC-1 expression in mitochondria after treated with different concentration of PM2.5 (0, 50 μg/mL, 100 μg/mL, 200 μg/mL). (Figure 5K-Cyto-Cyt-c) The expression of Cyt-c expression in cytoplasm after treated with different co [file elife-85944-fig5-data2.zip › Figure 5-source data 2/Figure5B-NQO-1.tif]

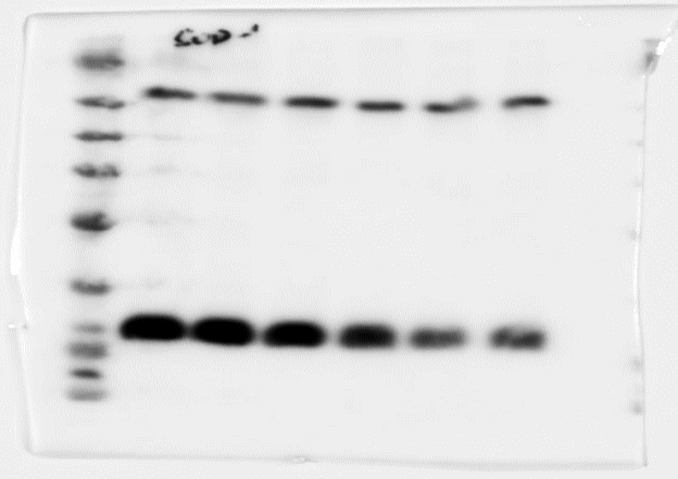

Supplement: Figure 5—source data 2. — (Figure 5B-HO-1) The expression of HO-1 expression in the placental tissue of Wild Type (The first three columns) and PM2.5-treated mice (the last three columns). (Figure 5B-NQO-1) The expression of NQO-1 expression in the placental tissue of Wild Type (The first three columns) and PM2.5-treated mice (the last three columns). (Figure 5B-GCLC) The expression of GCLC expression in the placental tissue of Wild Type (The first three columns) and PM2.5-treated mice (the last three columns). (Figure 5B-SOD-1) The expression of SOD-1 expression in the placental tissue of Wild Type (The first three columns) and PM2.5-treated mice (the last three columns). (Figure 5B-β-actin) The expression of β-actin expression in the placental tissue of Wild Type (The first three columns) and PM2.5-treated mice (the last three columns). (Figure 5C-HO-1) The expression of HO-1 expression in the PM2.5-treated HTR8/SVneo cells (PM2.5 concentration: 0. 50 μg/mL, 100 μg/mL, 200 μg/mL). (Figure 5C-NQO-1) The expression of NQO-1 expression in the PM2.5-treated HTR8/SVneo cells (PM2.5 concentration: 0. 50 μg/mL, 100 μg/mL, 200 μg/mL). (Figure 5C-GCLC) The expression of GCLC expression in the PM2.5-treated HTR8/SVneo cells (PM2.5 concentration: 0. 50 μg/mL, 100 μg/mL, 200 μg/mL). (Figure 5C-SOD-1) The expression of SOD-1 expression in the PM2.5-treated HTR8/SVneo cells (PM2.5 concentration: 0. 50 μg/mL, 100 μg/mL, 200 μg/mL). (Figure 5C-β-actin) The expression of β-actin expression in the PM2.5-treated HTR8/SVneo cells (PM2.5 concentration: 0. 50 μg/mL, 100 μg/mL, 200 μg/mL). (Figure 5K-Mito-Cyt-c) The expression of Cyt-c expression in mitochondria after treated with different concentration of PM2.5 (0, 50 μg/mL, 100 μg/mL, 200 μg/mL). (Figure 5K-Mito-VDAC-1) The expression of VDAC-1 expression in mitochondria after treated with different concentration of PM2.5 (0, 50 μg/mL, 100 μg/mL, 200 μg/mL). (Figure 5K-Cyto-Cyt-c) The expression of Cyt-c expression in cytoplasm after treated with different co [file elife-85944-fig5-data2.zip › Figure 5-source data 2/Figure5B-SOD-1.tif]

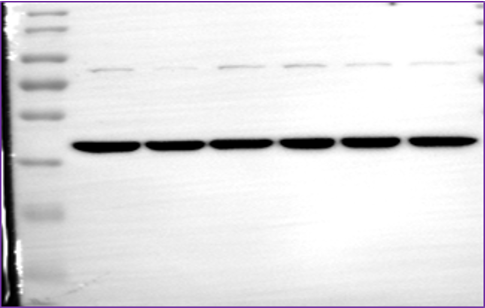

Supplement: Figure 5—source data 2. — (Figure 5B-HO-1) The expression of HO-1 expression in the placental tissue of Wild Type (The first three columns) and PM2.5-treated mice (the last three columns). (Figure 5B-NQO-1) The expression of NQO-1 expression in the placental tissue of Wild Type (The first three columns) and PM2.5-treated mice (the last three columns). (Figure 5B-GCLC) The expression of GCLC expression in the placental tissue of Wild Type (The first three columns) and PM2.5-treated mice (the last three columns). (Figure 5B-SOD-1) The expression of SOD-1 expression in the placental tissue of Wild Type (The first three columns) and PM2.5-treated mice (the last three columns). (Figure 5B-β-actin) The expression of β-actin expression in the placental tissue of Wild Type (The first three columns) and PM2.5-treated mice (the last three columns). (Figure 5C-HO-1) The expression of HO-1 expression in the PM2.5-treated HTR8/SVneo cells (PM2.5 concentration: 0. 50 μg/mL, 100 μg/mL, 200 μg/mL). (Figure 5C-NQO-1) The expression of NQO-1 expression in the PM2.5-treated HTR8/SVneo cells (PM2.5 concentration: 0. 50 μg/mL, 100 μg/mL, 200 μg/mL). (Figure 5C-GCLC) The expression of GCLC expression in the PM2.5-treated HTR8/SVneo cells (PM2.5 concentration: 0. 50 μg/mL, 100 μg/mL, 200 μg/mL). (Figure 5C-SOD-1) The expression of SOD-1 expression in the PM2.5-treated HTR8/SVneo cells (PM2.5 concentration: 0. 50 μg/mL, 100 μg/mL, 200 μg/mL). (Figure 5C-β-actin) The expression of β-actin expression in the PM2.5-treated HTR8/SVneo cells (PM2.5 concentration: 0. 50 μg/mL, 100 μg/mL, 200 μg/mL). (Figure 5K-Mito-Cyt-c) The expression of Cyt-c expression in mitochondria after treated with different concentration of PM2.5 (0, 50 μg/mL, 100 μg/mL, 200 μg/mL). (Figure 5K-Mito-VDAC-1) The expression of VDAC-1 expression in mitochondria after treated with different concentration of PM2.5 (0, 50 μg/mL, 100 μg/mL, 200 μg/mL). (Figure 5K-Cyto-Cyt-c) The expression of Cyt-c expression in cytoplasm after treated with different co [file elife-85944-fig5-data2.zip › Figure 5-source data 2/Figure5B-a┬-actin.tif]

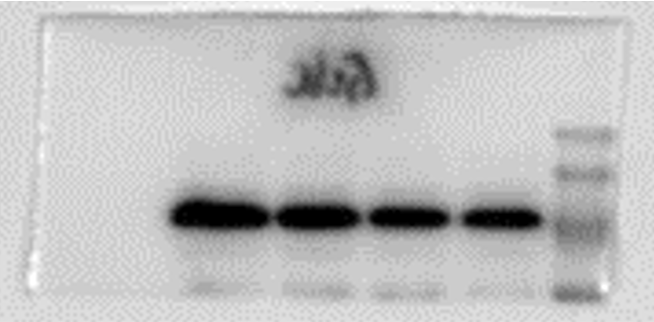

Supplement: Figure 5—source data 2. — (Figure 5B-HO-1) The expression of HO-1 expression in the placental tissue of Wild Type (The first three columns) and PM2.5-treated mice (the last three columns). (Figure 5B-NQO-1) The expression of NQO-1 expression in the placental tissue of Wild Type (The first three columns) and PM2.5-treated mice (the last three columns). (Figure 5B-GCLC) The expression of GCLC expression in the placental tissue of Wild Type (The first three columns) and PM2.5-treated mice (the last three columns). (Figure 5B-SOD-1) The expression of SOD-1 expression in the placental tissue of Wild Type (The first three columns) and PM2.5-treated mice (the last three columns). (Figure 5B-β-actin) The expression of β-actin expression in the placental tissue of Wild Type (The first three columns) and PM2.5-treated mice (the last three columns). (Figure 5C-HO-1) The expression of HO-1 expression in the PM2.5-treated HTR8/SVneo cells (PM2.5 concentration: 0. 50 μg/mL, 100 μg/mL, 200 μg/mL). (Figure 5C-NQO-1) The expression of NQO-1 expression in the PM2.5-treated HTR8/SVneo cells (PM2.5 concentration: 0. 50 μg/mL, 100 μg/mL, 200 μg/mL). (Figure 5C-GCLC) The expression of GCLC expression in the PM2.5-treated HTR8/SVneo cells (PM2.5 concentration: 0. 50 μg/mL, 100 μg/mL, 200 μg/mL). (Figure 5C-SOD-1) The expression of SOD-1 expression in the PM2.5-treated HTR8/SVneo cells (PM2.5 concentration: 0. 50 μg/mL, 100 μg/mL, 200 μg/mL). (Figure 5C-β-actin) The expression of β-actin expression in the PM2.5-treated HTR8/SVneo cells (PM2.5 concentration: 0. 50 μg/mL, 100 μg/mL, 200 μg/mL). (Figure 5K-Mito-Cyt-c) The expression of Cyt-c expression in mitochondria after treated with different concentration of PM2.5 (0, 50 μg/mL, 100 μg/mL, 200 μg/mL). (Figure 5K-Mito-VDAC-1) The expression of VDAC-1 expression in mitochondria after treated with different concentration of PM2.5 (0, 50 μg/mL, 100 μg/mL, 200 μg/mL). (Figure 5K-Cyto-Cyt-c) The expression of Cyt-c expression in cytoplasm after treated with different co [file elife-85944-fig5-data2.zip › Figure 5-source data 2/Figure5C-GCLC.tif]

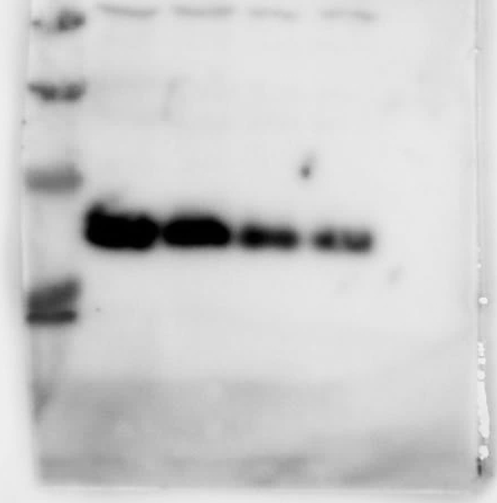

Supplement: Figure 5—source data 2. — (Figure 5B-HO-1) The expression of HO-1 expression in the placental tissue of Wild Type (The first three columns) and PM2.5-treated mice (the last three columns). (Figure 5B-NQO-1) The expression of NQO-1 expression in the placental tissue of Wild Type (The first three columns) and PM2.5-treated mice (the last three columns). (Figure 5B-GCLC) The expression of GCLC expression in the placental tissue of Wild Type (The first three columns) and PM2.5-treated mice (the last three columns). (Figure 5B-SOD-1) The expression of SOD-1 expression in the placental tissue of Wild Type (The first three columns) and PM2.5-treated mice (the last three columns). (Figure 5B-β-actin) The expression of β-actin expression in the placental tissue of Wild Type (The first three columns) and PM2.5-treated mice (the last three columns). (Figure 5C-HO-1) The expression of HO-1 expression in the PM2.5-treated HTR8/SVneo cells (PM2.5 concentration: 0. 50 μg/mL, 100 μg/mL, 200 μg/mL). (Figure 5C-NQO-1) The expression of NQO-1 expression in the PM2.5-treated HTR8/SVneo cells (PM2.5 concentration: 0. 50 μg/mL, 100 μg/mL, 200 μg/mL). (Figure 5C-GCLC) The expression of GCLC expression in the PM2.5-treated HTR8/SVneo cells (PM2.5 concentration: 0. 50 μg/mL, 100 μg/mL, 200 μg/mL). (Figure 5C-SOD-1) The expression of SOD-1 expression in the PM2.5-treated HTR8/SVneo cells (PM2.5 concentration: 0. 50 μg/mL, 100 μg/mL, 200 μg/mL). (Figure 5C-β-actin) The expression of β-actin expression in the PM2.5-treated HTR8/SVneo cells (PM2.5 concentration: 0. 50 μg/mL, 100 μg/mL, 200 μg/mL). (Figure 5K-Mito-Cyt-c) The expression of Cyt-c expression in mitochondria after treated with different concentration of PM2.5 (0, 50 μg/mL, 100 μg/mL, 200 μg/mL). (Figure 5K-Mito-VDAC-1) The expression of VDAC-1 expression in mitochondria after treated with different concentration of PM2.5 (0, 50 μg/mL, 100 μg/mL, 200 μg/mL). (Figure 5K-Cyto-Cyt-c) The expression of Cyt-c expression in cytoplasm after treated with different co [file elife-85944-fig5-data2.zip › Figure 5-source data 2/Figure5C-HO-1.tif]

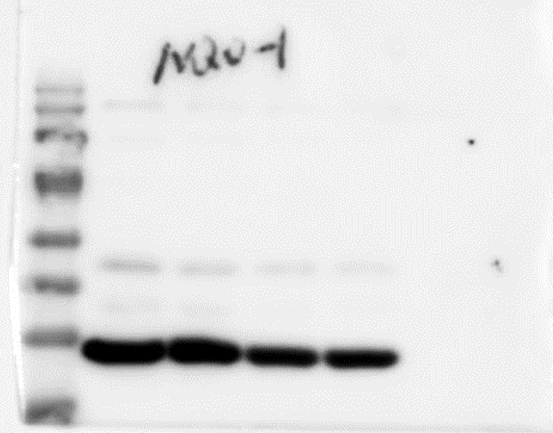

Supplement: Figure 5—source data 2. — (Figure 5B-HO-1) The expression of HO-1 expression in the placental tissue of Wild Type (The first three columns) and PM2.5-treated mice (the last three columns). (Figure 5B-NQO-1) The expression of NQO-1 expression in the placental tissue of Wild Type (The first three columns) and PM2.5-treated mice (the last three columns). (Figure 5B-GCLC) The expression of GCLC expression in the placental tissue of Wild Type (The first three columns) and PM2.5-treated mice (the last three columns). (Figure 5B-SOD-1) The expression of SOD-1 expression in the placental tissue of Wild Type (The first three columns) and PM2.5-treated mice (the last three columns). (Figure 5B-β-actin) The expression of β-actin expression in the placental tissue of Wild Type (The first three columns) and PM2.5-treated mice (the last three columns). (Figure 5C-HO-1) The expression of HO-1 expression in the PM2.5-treated HTR8/SVneo cells (PM2.5 concentration: 0. 50 μg/mL, 100 μg/mL, 200 μg/mL). (Figure 5C-NQO-1) The expression of NQO-1 expression in the PM2.5-treated HTR8/SVneo cells (PM2.5 concentration: 0. 50 μg/mL, 100 μg/mL, 200 μg/mL). (Figure 5C-GCLC) The expression of GCLC expression in the PM2.5-treated HTR8/SVneo cells (PM2.5 concentration: 0. 50 μg/mL, 100 μg/mL, 200 μg/mL). (Figure 5C-SOD-1) The expression of SOD-1 expression in the PM2.5-treated HTR8/SVneo cells (PM2.5 concentration: 0. 50 μg/mL, 100 μg/mL, 200 μg/mL). (Figure 5C-β-actin) The expression of β-actin expression in the PM2.5-treated HTR8/SVneo cells (PM2.5 concentration: 0. 50 μg/mL, 100 μg/mL, 200 μg/mL). (Figure 5K-Mito-Cyt-c) The expression of Cyt-c expression in mitochondria after treated with different concentration of PM2.5 (0, 50 μg/mL, 100 μg/mL, 200 μg/mL). (Figure 5K-Mito-VDAC-1) The expression of VDAC-1 expression in mitochondria after treated with different concentration of PM2.5 (0, 50 μg/mL, 100 μg/mL, 200 μg/mL). (Figure 5K-Cyto-Cyt-c) The expression of Cyt-c expression in cytoplasm after treated with different co [file elife-85944-fig5-data2.zip › Figure 5-source data 2/Figure5C-NQO-1.tif]

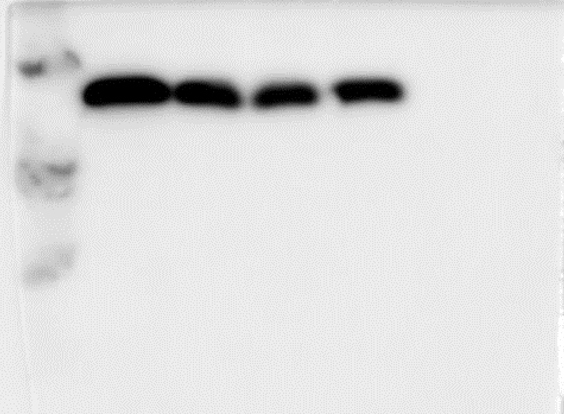

Supplement: Figure 5—source data 2. — (Figure 5B-HO-1) The expression of HO-1 expression in the placental tissue of Wild Type (The first three columns) and PM2.5-treated mice (the last three columns). (Figure 5B-NQO-1) The expression of NQO-1 expression in the placental tissue of Wild Type (The first three columns) and PM2.5-treated mice (the last three columns). (Figure 5B-GCLC) The expression of GCLC expression in the placental tissue of Wild Type (The first three columns) and PM2.5-treated mice (the last three columns). (Figure 5B-SOD-1) The expression of SOD-1 expression in the placental tissue of Wild Type (The first three columns) and PM2.5-treated mice (the last three columns). (Figure 5B-β-actin) The expression of β-actin expression in the placental tissue of Wild Type (The first three columns) and PM2.5-treated mice (the last three columns). (Figure 5C-HO-1) The expression of HO-1 expression in the PM2.5-treated HTR8/SVneo cells (PM2.5 concentration: 0. 50 μg/mL, 100 μg/mL, 200 μg/mL). (Figure 5C-NQO-1) The expression of NQO-1 expression in the PM2.5-treated HTR8/SVneo cells (PM2.5 concentration: 0. 50 μg/mL, 100 μg/mL, 200 μg/mL). (Figure 5C-GCLC) The expression of GCLC expression in the PM2.5-treated HTR8/SVneo cells (PM2.5 concentration: 0. 50 μg/mL, 100 μg/mL, 200 μg/mL). (Figure 5C-SOD-1) The expression of SOD-1 expression in the PM2.5-treated HTR8/SVneo cells (PM2.5 concentration: 0. 50 μg/mL, 100 μg/mL, 200 μg/mL). (Figure 5C-β-actin) The expression of β-actin expression in the PM2.5-treated HTR8/SVneo cells (PM2.5 concentration: 0. 50 μg/mL, 100 μg/mL, 200 μg/mL). (Figure 5K-Mito-Cyt-c) The expression of Cyt-c expression in mitochondria after treated with different concentration of PM2.5 (0, 50 μg/mL, 100 μg/mL, 200 μg/mL). (Figure 5K-Mito-VDAC-1) The expression of VDAC-1 expression in mitochondria after treated with different concentration of PM2.5 (0, 50 μg/mL, 100 μg/mL, 200 μg/mL). (Figure 5K-Cyto-Cyt-c) The expression of Cyt-c expression in cytoplasm after treated with different co [file elife-85944-fig5-data2.zip › Figure 5-source data 2/Figure5C-SOD-1.tif]

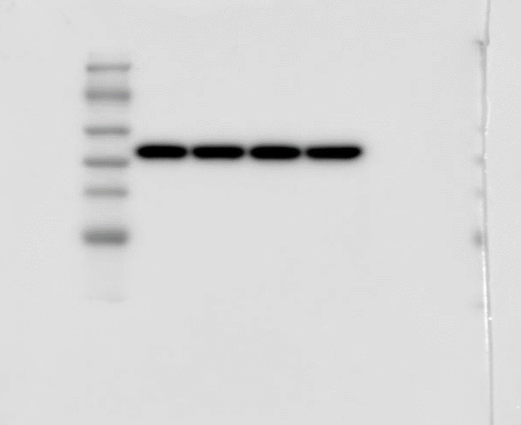

Supplement: Figure 5—source data 2. — (Figure 5B-HO-1) The expression of HO-1 expression in the placental tissue of Wild Type (The first three columns) and PM2.5-treated mice (the last three columns). (Figure 5B-NQO-1) The expression of NQO-1 expression in the placental tissue of Wild Type (The first three columns) and PM2.5-treated mice (the last three columns). (Figure 5B-GCLC) The expression of GCLC expression in the placental tissue of Wild Type (The first three columns) and PM2.5-treated mice (the last three columns). (Figure 5B-SOD-1) The expression of SOD-1 expression in the placental tissue of Wild Type (The first three columns) and PM2.5-treated mice (the last three columns). (Figure 5B-β-actin) The expression of β-actin expression in the placental tissue of Wild Type (The first three columns) and PM2.5-treated mice (the last three columns). (Figure 5C-HO-1) The expression of HO-1 expression in the PM2.5-treated HTR8/SVneo cells (PM2.5 concentration: 0. 50 μg/mL, 100 μg/mL, 200 μg/mL). (Figure 5C-NQO-1) The expression of NQO-1 expression in the PM2.5-treated HTR8/SVneo cells (PM2.5 concentration: 0. 50 μg/mL, 100 μg/mL, 200 μg/mL). (Figure 5C-GCLC) The expression of GCLC expression in the PM2.5-treated HTR8/SVneo cells (PM2.5 concentration: 0. 50 μg/mL, 100 μg/mL, 200 μg/mL). (Figure 5C-SOD-1) The expression of SOD-1 expression in the PM2.5-treated HTR8/SVneo cells (PM2.5 concentration: 0. 50 μg/mL, 100 μg/mL, 200 μg/mL). (Figure 5C-β-actin) The expression of β-actin expression in the PM2.5-treated HTR8/SVneo cells (PM2.5 concentration: 0. 50 μg/mL, 100 μg/mL, 200 μg/mL). (Figure 5K-Mito-Cyt-c) The expression of Cyt-c expression in mitochondria after treated with different concentration of PM2.5 (0, 50 μg/mL, 100 μg/mL, 200 μg/mL). (Figure 5K-Mito-VDAC-1) The expression of VDAC-1 expression in mitochondria after treated with different concentration of PM2.5 (0, 50 μg/mL, 100 μg/mL, 200 μg/mL). (Figure 5K-Cyto-Cyt-c) The expression of Cyt-c expression in cytoplasm after treated with different co [file elife-85944-fig5-data2.zip › Figure 5-source data 2/Figure5C-a┬-actin.tif]

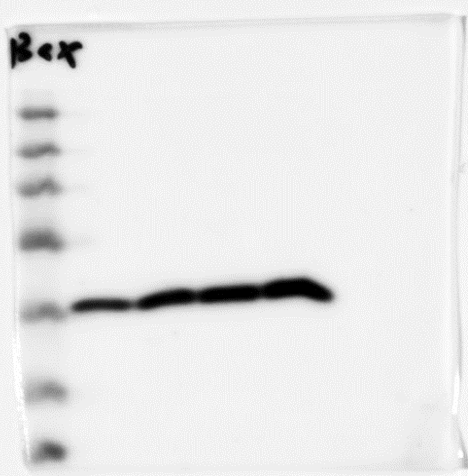

Supplement: Figure 5—source data 2. — (Figure 5B-HO-1) The expression of HO-1 expression in the placental tissue of Wild Type (The first three columns) and PM2.5-treated mice (the last three columns). (Figure 5B-NQO-1) The expression of NQO-1 expression in the placental tissue of Wild Type (The first three columns) and PM2.5-treated mice (the last three columns). (Figure 5B-GCLC) The expression of GCLC expression in the placental tissue of Wild Type (The first three columns) and PM2.5-treated mice (the last three columns). (Figure 5B-SOD-1) The expression of SOD-1 expression in the placental tissue of Wild Type (The first three columns) and PM2.5-treated mice (the last three columns). (Figure 5B-β-actin) The expression of β-actin expression in the placental tissue of Wild Type (The first three columns) and PM2.5-treated mice (the last three columns). (Figure 5C-HO-1) The expression of HO-1 expression in the PM2.5-treated HTR8/SVneo cells (PM2.5 concentration: 0. 50 μg/mL, 100 μg/mL, 200 μg/mL). (Figure 5C-NQO-1) The expression of NQO-1 expression in the PM2.5-treated HTR8/SVneo cells (PM2.5 concentration: 0. 50 μg/mL, 100 μg/mL, 200 μg/mL). (Figure 5C-GCLC) The expression of GCLC expression in the PM2.5-treated HTR8/SVneo cells (PM2.5 concentration: 0. 50 μg/mL, 100 μg/mL, 200 μg/mL). (Figure 5C-SOD-1) The expression of SOD-1 expression in the PM2.5-treated HTR8/SVneo cells (PM2.5 concentration: 0. 50 μg/mL, 100 μg/mL, 200 μg/mL). (Figure 5C-β-actin) The expression of β-actin expression in the PM2.5-treated HTR8/SVneo cells (PM2.5 concentration: 0. 50 μg/mL, 100 μg/mL, 200 μg/mL). (Figure 5K-Mito-Cyt-c) The expression of Cyt-c expression in mitochondria after treated with different concentration of PM2.5 (0, 50 μg/mL, 100 μg/mL, 200 μg/mL). (Figure 5K-Mito-VDAC-1) The expression of VDAC-1 expression in mitochondria after treated with different concentration of PM2.5 (0, 50 μg/mL, 100 μg/mL, 200 μg/mL). (Figure 5K-Cyto-Cyt-c) The expression of Cyt-c expression in cytoplasm after treated with different co [file elife-85944-fig5-data2.zip › Figure 5-source data 2/Figure5K-Cyto-BAX.tif]

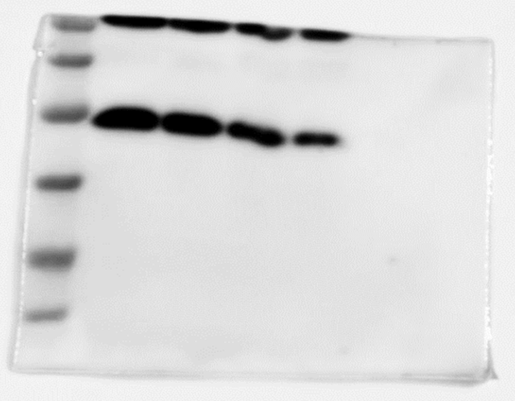

Supplement: Figure 5—source data 2. — (Figure 5B-HO-1) The expression of HO-1 expression in the placental tissue of Wild Type (The first three columns) and PM2.5-treated mice (the last three columns). (Figure 5B-NQO-1) The expression of NQO-1 expression in the placental tissue of Wild Type (The first three columns) and PM2.5-treated mice (the last three columns). (Figure 5B-GCLC) The expression of GCLC expression in the placental tissue of Wild Type (The first three columns) and PM2.5-treated mice (the last three columns). (Figure 5B-SOD-1) The expression of SOD-1 expression in the placental tissue of Wild Type (The first three columns) and PM2.5-treated mice (the last three columns). (Figure 5B-β-actin) The expression of β-actin expression in the placental tissue of Wild Type (The first three columns) and PM2.5-treated mice (the last three columns). (Figure 5C-HO-1) The expression of HO-1 expression in the PM2.5-treated HTR8/SVneo cells (PM2.5 concentration: 0. 50 μg/mL, 100 μg/mL, 200 μg/mL). (Figure 5C-NQO-1) The expression of NQO-1 expression in the PM2.5-treated HTR8/SVneo cells (PM2.5 concentration: 0. 50 μg/mL, 100 μg/mL, 200 μg/mL). (Figure 5C-GCLC) The expression of GCLC expression in the PM2.5-treated HTR8/SVneo cells (PM2.5 concentration: 0. 50 μg/mL, 100 μg/mL, 200 μg/mL). (Figure 5C-SOD-1) The expression of SOD-1 expression in the PM2.5-treated HTR8/SVneo cells (PM2.5 concentration: 0. 50 μg/mL, 100 μg/mL, 200 μg/mL). (Figure 5C-β-actin) The expression of β-actin expression in the PM2.5-treated HTR8/SVneo cells (PM2.5 concentration: 0. 50 μg/mL, 100 μg/mL, 200 μg/mL). (Figure 5K-Mito-Cyt-c) The expression of Cyt-c expression in mitochondria after treated with different concentration of PM2.5 (0, 50 μg/mL, 100 μg/mL, 200 μg/mL). (Figure 5K-Mito-VDAC-1) The expression of VDAC-1 expression in mitochondria after treated with different concentration of PM2.5 (0, 50 μg/mL, 100 μg/mL, 200 μg/mL). (Figure 5K-Cyto-Cyt-c) The expression of Cyt-c expression in cytoplasm after treated with different co [file elife-85944-fig5-data2.zip › Figure 5-source data 2/Figure5K-Cyto-BCL-2.tif]

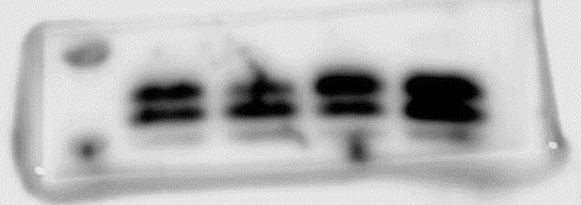

Supplement: Figure 5—source data 2. — (Figure 5B-HO-1) The expression of HO-1 expression in the placental tissue of Wild Type (The first three columns) and PM2.5-treated mice (the last three columns). (Figure 5B-NQO-1) The expression of NQO-1 expression in the placental tissue of Wild Type (The first three columns) and PM2.5-treated mice (the last three columns). (Figure 5B-GCLC) The expression of GCLC expression in the placental tissue of Wild Type (The first three columns) and PM2.5-treated mice (the last three columns). (Figure 5B-SOD-1) The expression of SOD-1 expression in the placental tissue of Wild Type (The first three columns) and PM2.5-treated mice (the last three columns). (Figure 5B-β-actin) The expression of β-actin expression in the placental tissue of Wild Type (The first three columns) and PM2.5-treated mice (the last three columns). (Figure 5C-HO-1) The expression of HO-1 expression in the PM2.5-treated HTR8/SVneo cells (PM2.5 concentration: 0. 50 μg/mL, 100 μg/mL, 200 μg/mL). (Figure 5C-NQO-1) The expression of NQO-1 expression in the PM2.5-treated HTR8/SVneo cells (PM2.5 concentration: 0. 50 μg/mL, 100 μg/mL, 200 μg/mL). (Figure 5C-GCLC) The expression of GCLC expression in the PM2.5-treated HTR8/SVneo cells (PM2.5 concentration: 0. 50 μg/mL, 100 μg/mL, 200 μg/mL). (Figure 5C-SOD-1) The expression of SOD-1 expression in the PM2.5-treated HTR8/SVneo cells (PM2.5 concentration: 0. 50 μg/mL, 100 μg/mL, 200 μg/mL). (Figure 5C-β-actin) The expression of β-actin expression in the PM2.5-treated HTR8/SVneo cells (PM2.5 concentration: 0. 50 μg/mL, 100 μg/mL, 200 μg/mL). (Figure 5K-Mito-Cyt-c) The expression of Cyt-c expression in mitochondria after treated with different concentration of PM2.5 (0, 50 μg/mL, 100 μg/mL, 200 μg/mL). (Figure 5K-Mito-VDAC-1) The expression of VDAC-1 expression in mitochondria after treated with different concentration of PM2.5 (0, 50 μg/mL, 100 μg/mL, 200 μg/mL). (Figure 5K-Cyto-Cyt-c) The expression of Cyt-c expression in cytoplasm after treated with different co [file elife-85944-fig5-data2.zip › Figure 5-source data 2/Figure5K-Cyto-CC3.tif]

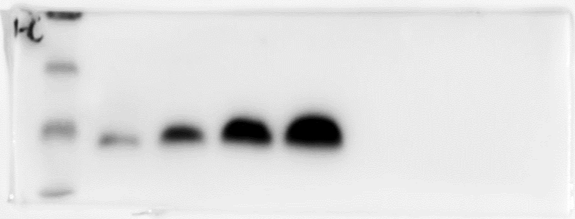

Supplement: Figure 5—source data 2. — (Figure 5B-HO-1) The expression of HO-1 expression in the placental tissue of Wild Type (The first three columns) and PM2.5-treated mice (the last three columns). (Figure 5B-NQO-1) The expression of NQO-1 expression in the placental tissue of Wild Type (The first three columns) and PM2.5-treated mice (the last three columns). (Figure 5B-GCLC) The expression of GCLC expression in the placental tissue of Wild Type (The first three columns) and PM2.5-treated mice (the last three columns). (Figure 5B-SOD-1) The expression of SOD-1 expression in the placental tissue of Wild Type (The first three columns) and PM2.5-treated mice (the last three columns). (Figure 5B-β-actin) The expression of β-actin expression in the placental tissue of Wild Type (The first three columns) and PM2.5-treated mice (the last three columns). (Figure 5C-HO-1) The expression of HO-1 expression in the PM2.5-treated HTR8/SVneo cells (PM2.5 concentration: 0. 50 μg/mL, 100 μg/mL, 200 μg/mL). (Figure 5C-NQO-1) The expression of NQO-1 expression in the PM2.5-treated HTR8/SVneo cells (PM2.5 concentration: 0. 50 μg/mL, 100 μg/mL, 200 μg/mL). (Figure 5C-GCLC) The expression of GCLC expression in the PM2.5-treated HTR8/SVneo cells (PM2.5 concentration: 0. 50 μg/mL, 100 μg/mL, 200 μg/mL). (Figure 5C-SOD-1) The expression of SOD-1 expression in the PM2.5-treated HTR8/SVneo cells (PM2.5 concentration: 0. 50 μg/mL, 100 μg/mL, 200 μg/mL). (Figure 5C-β-actin) The expression of β-actin expression in the PM2.5-treated HTR8/SVneo cells (PM2.5 concentration: 0. 50 μg/mL, 100 μg/mL, 200 μg/mL). (Figure 5K-Mito-Cyt-c) The expression of Cyt-c expression in mitochondria after treated with different concentration of PM2.5 (0, 50 μg/mL, 100 μg/mL, 200 μg/mL). (Figure 5K-Mito-VDAC-1) The expression of VDAC-1 expression in mitochondria after treated with different concentration of PM2.5 (0, 50 μg/mL, 100 μg/mL, 200 μg/mL). (Figure 5K-Cyto-Cyt-c) The expression of Cyt-c expression in cytoplasm after treated with different co [file elife-85944-fig5-data2.zip › Figure 5-source data 2/Figure5K-Cyto-CytC.tif]

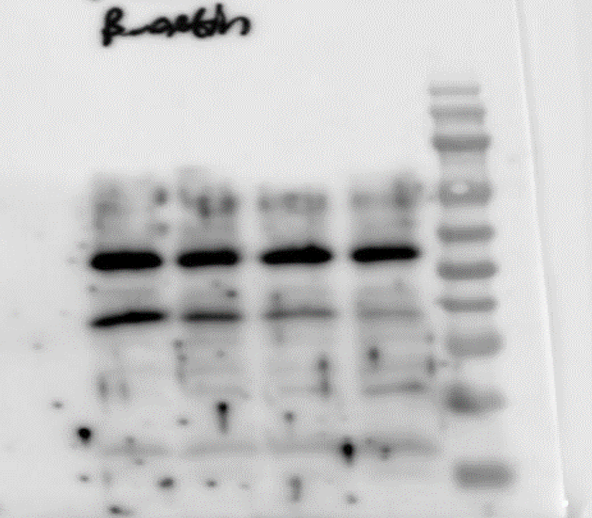

Supplement: Figure 5—source data 2. — (Figure 5B-HO-1) The expression of HO-1 expression in the placental tissue of Wild Type (The first three columns) and PM2.5-treated mice (the last three columns). (Figure 5B-NQO-1) The expression of NQO-1 expression in the placental tissue of Wild Type (The first three columns) and PM2.5-treated mice (the last three columns). (Figure 5B-GCLC) The expression of GCLC expression in the placental tissue of Wild Type (The first three columns) and PM2.5-treated mice (the last three columns). (Figure 5B-SOD-1) The expression of SOD-1 expression in the placental tissue of Wild Type (The first three columns) and PM2.5-treated mice (the last three columns). (Figure 5B-β-actin) The expression of β-actin expression in the placental tissue of Wild Type (The first three columns) and PM2.5-treated mice (the last three columns). (Figure 5C-HO-1) The expression of HO-1 expression in the PM2.5-treated HTR8/SVneo cells (PM2.5 concentration: 0. 50 μg/mL, 100 μg/mL, 200 μg/mL). (Figure 5C-NQO-1) The expression of NQO-1 expression in the PM2.5-treated HTR8/SVneo cells (PM2.5 concentration: 0. 50 μg/mL, 100 μg/mL, 200 μg/mL). (Figure 5C-GCLC) The expression of GCLC expression in the PM2.5-treated HTR8/SVneo cells (PM2.5 concentration: 0. 50 μg/mL, 100 μg/mL, 200 μg/mL). (Figure 5C-SOD-1) The expression of SOD-1 expression in the PM2.5-treated HTR8/SVneo cells (PM2.5 concentration: 0. 50 μg/mL, 100 μg/mL, 200 μg/mL). (Figure 5C-β-actin) The expression of β-actin expression in the PM2.5-treated HTR8/SVneo cells (PM2.5 concentration: 0. 50 μg/mL, 100 μg/mL, 200 μg/mL). (Figure 5K-Mito-Cyt-c) The expression of Cyt-c expression in mitochondria after treated with different concentration of PM2.5 (0, 50 μg/mL, 100 μg/mL, 200 μg/mL). (Figure 5K-Mito-VDAC-1) The expression of VDAC-1 expression in mitochondria after treated with different concentration of PM2.5 (0, 50 μg/mL, 100 μg/mL, 200 μg/mL). (Figure 5K-Cyto-Cyt-c) The expression of Cyt-c expression in cytoplasm after treated with different co [file elife-85944-fig5-data2.zip › Figure 5-source data 2/Figure5K-Cyto-a┬-actin .tif]

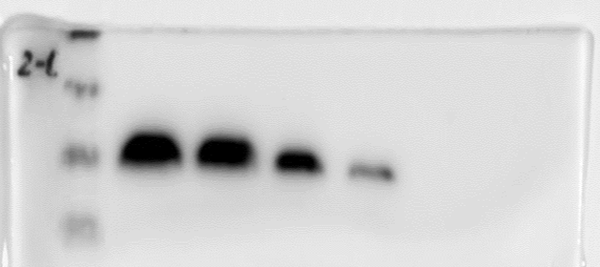

Supplement: Figure 5—source data 2. — (Figure 5B-HO-1) The expression of HO-1 expression in the placental tissue of Wild Type (The first three columns) and PM2.5-treated mice (the last three columns). (Figure 5B-NQO-1) The expression of NQO-1 expression in the placental tissue of Wild Type (The first three columns) and PM2.5-treated mice (the last three columns). (Figure 5B-GCLC) The expression of GCLC expression in the placental tissue of Wild Type (The first three columns) and PM2.5-treated mice (the last three columns). (Figure 5B-SOD-1) The expression of SOD-1 expression in the placental tissue of Wild Type (The first three columns) and PM2.5-treated mice (the last three columns). (Figure 5B-β-actin) The expression of β-actin expression in the placental tissue of Wild Type (The first three columns) and PM2.5-treated mice (the last three columns). (Figure 5C-HO-1) The expression of HO-1 expression in the PM2.5-treated HTR8/SVneo cells (PM2.5 concentration: 0. 50 μg/mL, 100 μg/mL, 200 μg/mL). (Figure 5C-NQO-1) The expression of NQO-1 expression in the PM2.5-treated HTR8/SVneo cells (PM2.5 concentration: 0. 50 μg/mL, 100 μg/mL, 200 μg/mL). (Figure 5C-GCLC) The expression of GCLC expression in the PM2.5-treated HTR8/SVneo cells (PM2.5 concentration: 0. 50 μg/mL, 100 μg/mL, 200 μg/mL). (Figure 5C-SOD-1) The expression of SOD-1 expression in the PM2.5-treated HTR8/SVneo cells (PM2.5 concentration: 0. 50 μg/mL, 100 μg/mL, 200 μg/mL). (Figure 5C-β-actin) The expression of β-actin expression in the PM2.5-treated HTR8/SVneo cells (PM2.5 concentration: 0. 50 μg/mL, 100 μg/mL, 200 μg/mL). (Figure 5K-Mito-Cyt-c) The expression of Cyt-c expression in mitochondria after treated with different concentration of PM2.5 (0, 50 μg/mL, 100 μg/mL, 200 μg/mL). (Figure 5K-Mito-VDAC-1) The expression of VDAC-1 expression in mitochondria after treated with different concentration of PM2.5 (0, 50 μg/mL, 100 μg/mL, 200 μg/mL). (Figure 5K-Cyto-Cyt-c) The expression of Cyt-c expression in cytoplasm after treated with different co [file elife-85944-fig5-data2.zip › Figure 5-source data 2/Figure5K-Mito-CytC.tif]

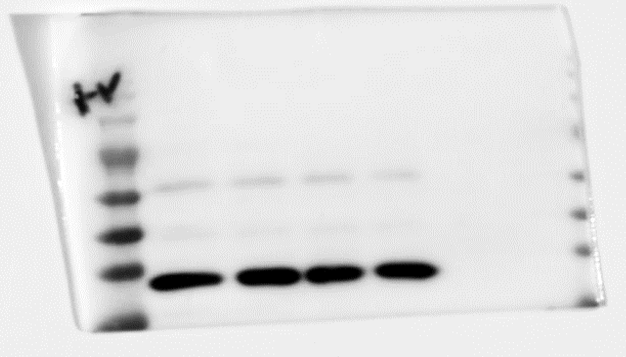

Supplement: Figure 5—source data 2. — (Figure 5B-HO-1) The expression of HO-1 expression in the placental tissue of Wild Type (The first three columns) and PM2.5-treated mice (the last three columns). (Figure 5B-NQO-1) The expression of NQO-1 expression in the placental tissue of Wild Type (The first three columns) and PM2.5-treated mice (the last three columns). (Figure 5B-GCLC) The expression of GCLC expression in the placental tissue of Wild Type (The first three columns) and PM2.5-treated mice (the last three columns). (Figure 5B-SOD-1) The expression of SOD-1 expression in the placental tissue of Wild Type (The first three columns) and PM2.5-treated mice (the last three columns). (Figure 5B-β-actin) The expression of β-actin expression in the placental tissue of Wild Type (The first three columns) and PM2.5-treated mice (the last three columns). (Figure 5C-HO-1) The expression of HO-1 expression in the PM2.5-treated HTR8/SVneo cells (PM2.5 concentration: 0. 50 μg/mL, 100 μg/mL, 200 μg/mL). (Figure 5C-NQO-1) The expression of NQO-1 expression in the PM2.5-treated HTR8/SVneo cells (PM2.5 concentration: 0. 50 μg/mL, 100 μg/mL, 200 μg/mL). (Figure 5C-GCLC) The expression of GCLC expression in the PM2.5-treated HTR8/SVneo cells (PM2.5 concentration: 0. 50 μg/mL, 100 μg/mL, 200 μg/mL). (Figure 5C-SOD-1) The expression of SOD-1 expression in the PM2.5-treated HTR8/SVneo cells (PM2.5 concentration: 0. 50 μg/mL, 100 μg/mL, 200 μg/mL). (Figure 5C-β-actin) The expression of β-actin expression in the PM2.5-treated HTR8/SVneo cells (PM2.5 concentration: 0. 50 μg/mL, 100 μg/mL, 200 μg/mL). (Figure 5K-Mito-Cyt-c) The expression of Cyt-c expression in mitochondria after treated with different concentration of PM2.5 (0, 50 μg/mL, 100 μg/mL, 200 μg/mL). (Figure 5K-Mito-VDAC-1) The expression of VDAC-1 expression in mitochondria after treated with different concentration of PM2.5 (0, 50 μg/mL, 100 μg/mL, 200 μg/mL). (Figure 5K-Cyto-Cyt-c) The expression of Cyt-c expression in cytoplasm after treated with different co [file elife-85944-fig5-data2.zip › Figure 5-source data 2/Figure5K-Mito-VDAC1.tif]

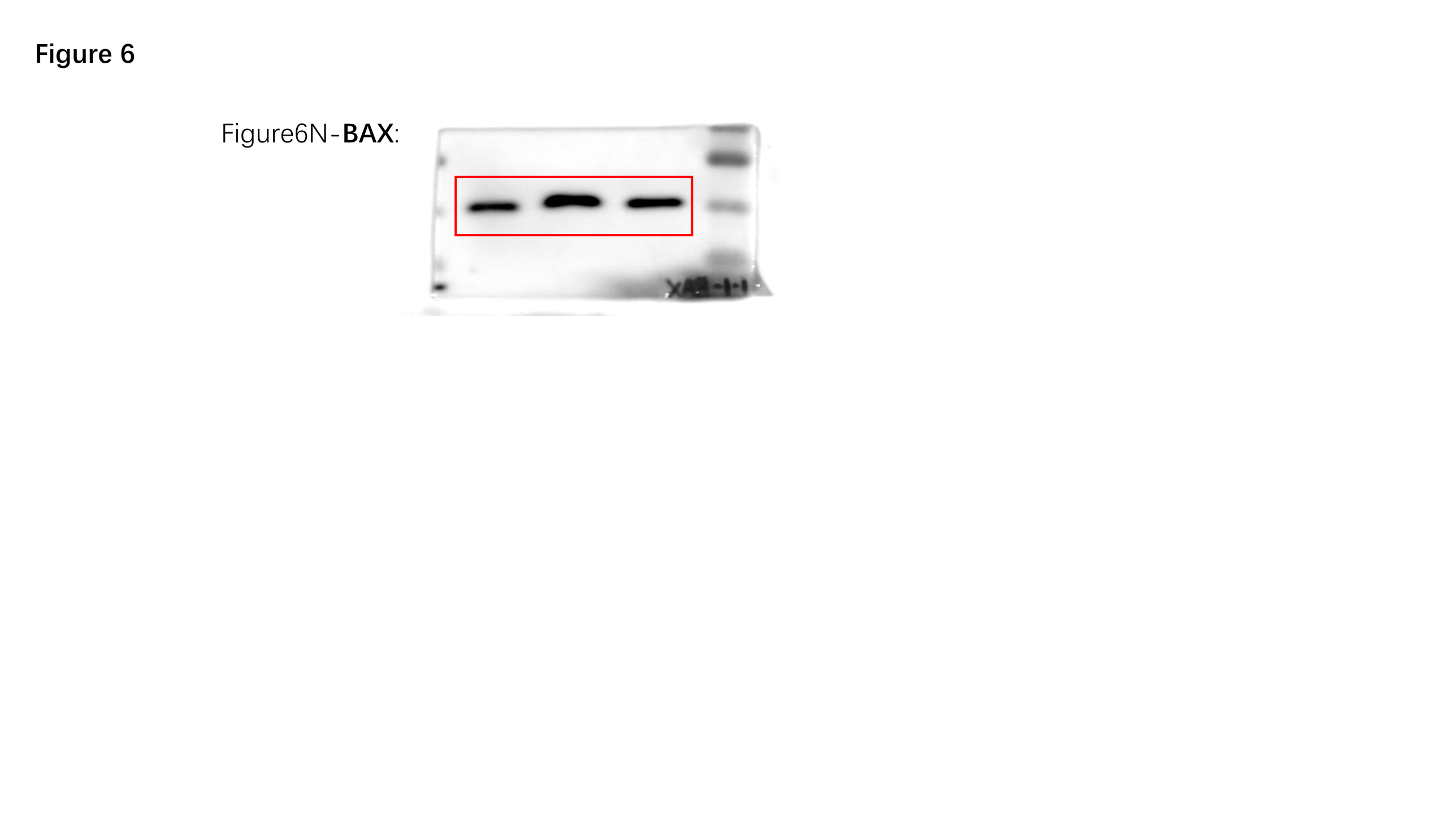

Supplement: Figure 6—source data 1. — (Figure 6N-BCL-2) The expression of BCL-2 expression in HTR8/SVneo (CON, PM2.5, PM2.5+NAC). (Figure 6N-BAX) The expression of BAX expression in HTR8/SVneo (CON, PM2.5, PM2.5+NAC). (Figure 6N-CC3) The expression of Cleaved-Caspase 3 expression in HTR8/SVneo (CON, PM2.5, PM2.5+NAC). (Figure 6N-β-actin) The expression of β-actin expression in HTR8/SVneo (CON, PM2.5, PM2.5+NAC). [file elife-85944-fig6-data1.zip › Figure 6-source data 1/Figure6N-BAX.TIF]

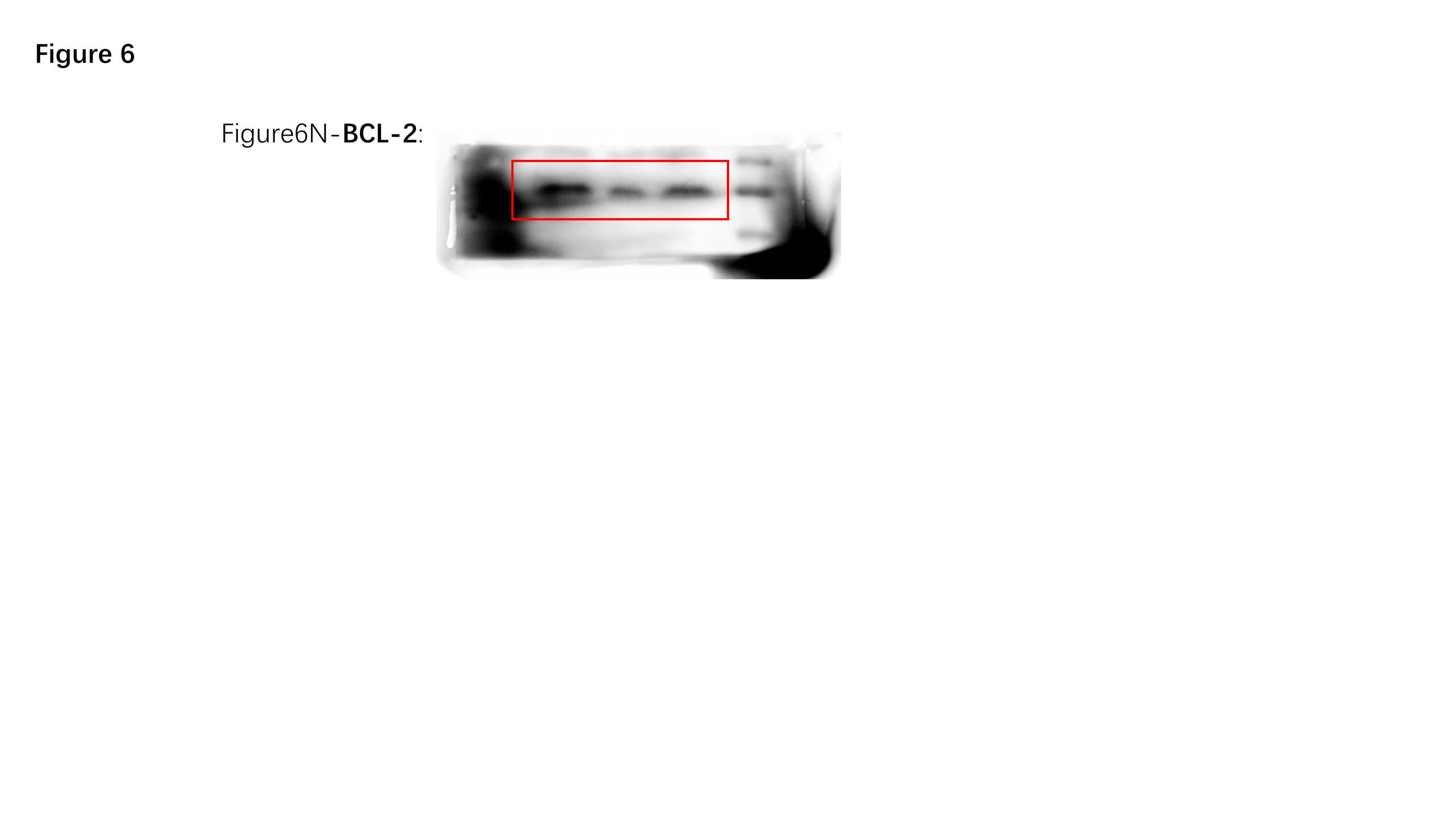

Supplement: Figure 6—source data 1. — (Figure 6N-BCL-2) The expression of BCL-2 expression in HTR8/SVneo (CON, PM2.5, PM2.5+NAC). (Figure 6N-BAX) The expression of BAX expression in HTR8/SVneo (CON, PM2.5, PM2.5+NAC). (Figure 6N-CC3) The expression of Cleaved-Caspase 3 expression in HTR8/SVneo (CON, PM2.5, PM2.5+NAC). (Figure 6N-β-actin) The expression of β-actin expression in HTR8/SVneo (CON, PM2.5, PM2.5+NAC). [file elife-85944-fig6-data1.zip › Figure 6-source data 1/Figure6N-BCL2.TIF]

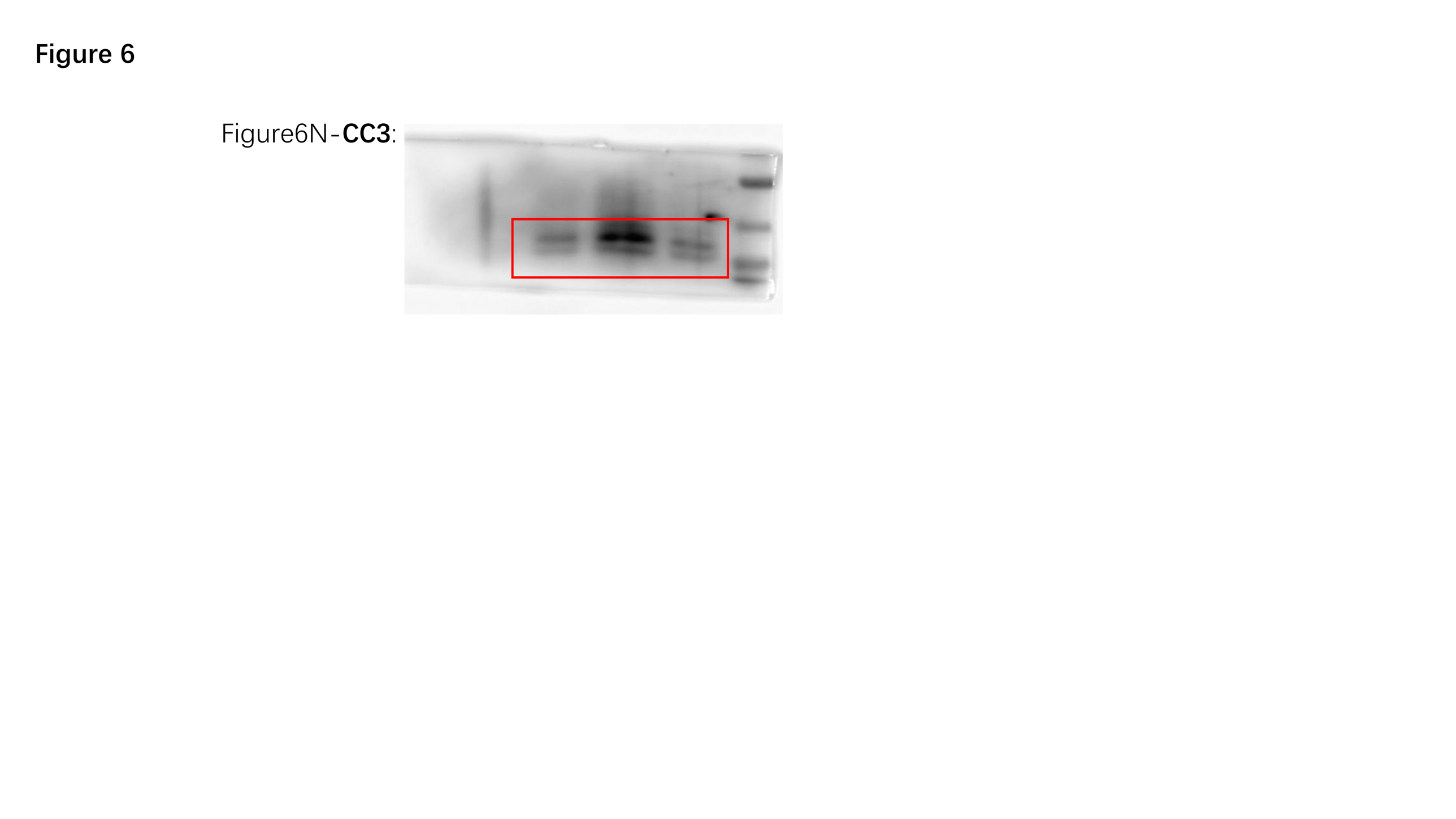

Supplement: Figure 6—source data 1. — (Figure 6N-BCL-2) The expression of BCL-2 expression in HTR8/SVneo (CON, PM2.5, PM2.5+NAC). (Figure 6N-BAX) The expression of BAX expression in HTR8/SVneo (CON, PM2.5, PM2.5+NAC). (Figure 6N-CC3) The expression of Cleaved-Caspase 3 expression in HTR8/SVneo (CON, PM2.5, PM2.5+NAC). (Figure 6N-β-actin) The expression of β-actin expression in HTR8/SVneo (CON, PM2.5, PM2.5+NAC). [file elife-85944-fig6-data1.zip › Figure 6-source data 1/Figure6N-CC3.TIF]

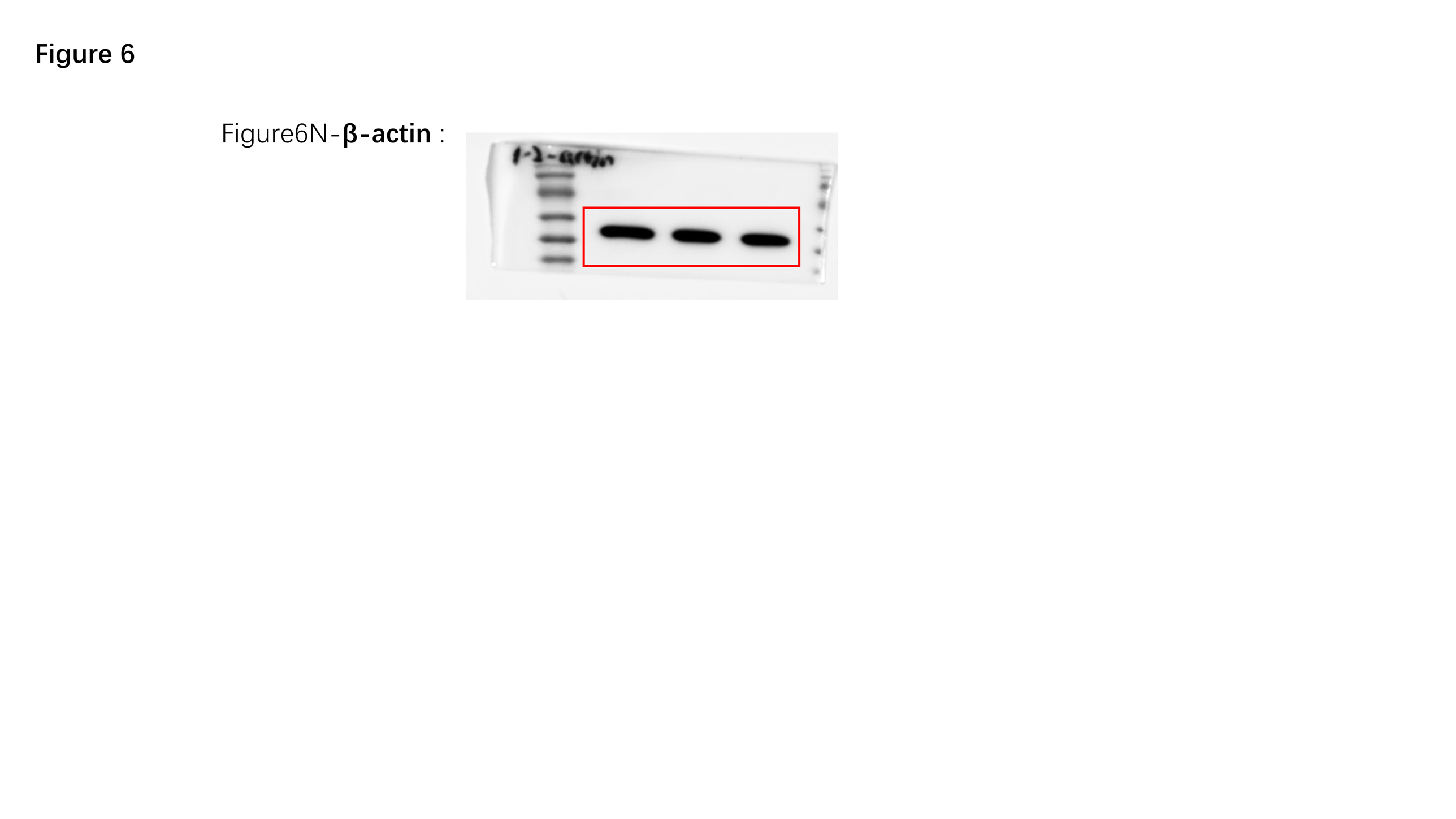

Supplement: Figure 6—source data 1. — (Figure 6N-BCL-2) The expression of BCL-2 expression in HTR8/SVneo (CON, PM2.5, PM2.5+NAC). (Figure 6N-BAX) The expression of BAX expression in HTR8/SVneo (CON, PM2.5, PM2.5+NAC). (Figure 6N-CC3) The expression of Cleaved-Caspase 3 expression in HTR8/SVneo (CON, PM2.5, PM2.5+NAC). (Figure 6N-β-actin) The expression of β-actin expression in HTR8/SVneo (CON, PM2.5, PM2.5+NAC). [file elife-85944-fig6-data1.zip › Figure 6-source data 1/Figure6N-a┬-actin.TIF]

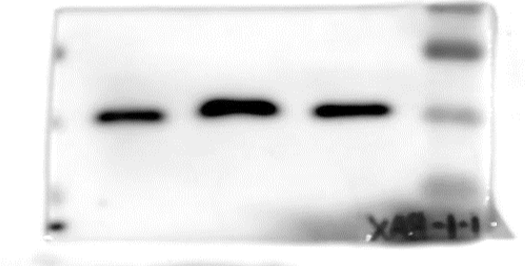

Supplement: Figure 6—source data 2. — (Figure 6N-BCL-2) The expression of BCL-2 expression in HTR8/SVneo (CON, PM2.5, PM2.5+NAC). (Figure 6N-BAX) The expression of BAX expression in HTR8/SVneo (CON, PM2.5, PM2.5+NAC). (Figure 6N-CC3) The expression of Cleaved-Caspase 3 expression in HTR8/SVneo (CON, PM2.5, PM2.5+NAC). (Figure 6N-β-actin) The expression of β-actin expression in HTR8/SVneo (CON, PM2.5, PM2.5+NAC). [file elife-85944-fig6-data2.zip › Figure 6-source data 2/Figure6N-BAX.tif]

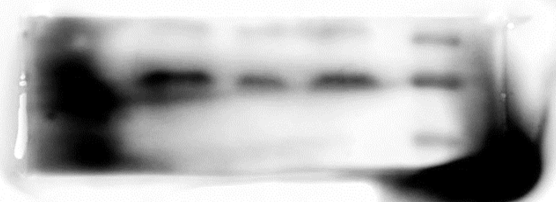

Supplement: Figure 6—source data 2. — (Figure 6N-BCL-2) The expression of BCL-2 expression in HTR8/SVneo (CON, PM2.5, PM2.5+NAC). (Figure 6N-BAX) The expression of BAX expression in HTR8/SVneo (CON, PM2.5, PM2.5+NAC). (Figure 6N-CC3) The expression of Cleaved-Caspase 3 expression in HTR8/SVneo (CON, PM2.5, PM2.5+NAC). (Figure 6N-β-actin) The expression of β-actin expression in HTR8/SVneo (CON, PM2.5, PM2.5+NAC). [file elife-85944-fig6-data2.zip › Figure 6-source data 2/Figure6N-BCL-2.tif]

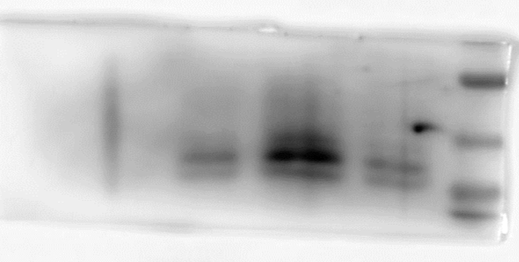

Supplement: Figure 6—source data 2. — (Figure 6N-BCL-2) The expression of BCL-2 expression in HTR8/SVneo (CON, PM2.5, PM2.5+NAC). (Figure 6N-BAX) The expression of BAX expression in HTR8/SVneo (CON, PM2.5, PM2.5+NAC). (Figure 6N-CC3) The expression of Cleaved-Caspase 3 expression in HTR8/SVneo (CON, PM2.5, PM2.5+NAC). (Figure 6N-β-actin) The expression of β-actin expression in HTR8/SVneo (CON, PM2.5, PM2.5+NAC). [file elife-85944-fig6-data2.zip › Figure 6-source data 2/Figure6N-CC3.tif]

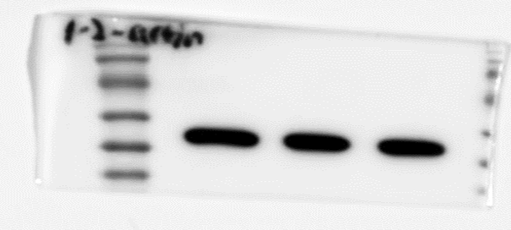

Supplement: Figure 6—source data 2. — (Figure 6N-BCL-2) The expression of BCL-2 expression in HTR8/SVneo (CON, PM2.5, PM2.5+NAC). (Figure 6N-BAX) The expression of BAX expression in HTR8/SVneo (CON, PM2.5, PM2.5+NAC). (Figure 6N-CC3) The expression of Cleaved-Caspase 3 expression in HTR8/SVneo (CON, PM2.5, PM2.5+NAC). (Figure 6N-β-actin) The expression of β-actin expression in HTR8/SVneo (CON, PM2.5, PM2.5+NAC). [file elife-85944-fig6-data2.zip › Figure 6-source data 2/Figure6N-a┬-actin.tif]

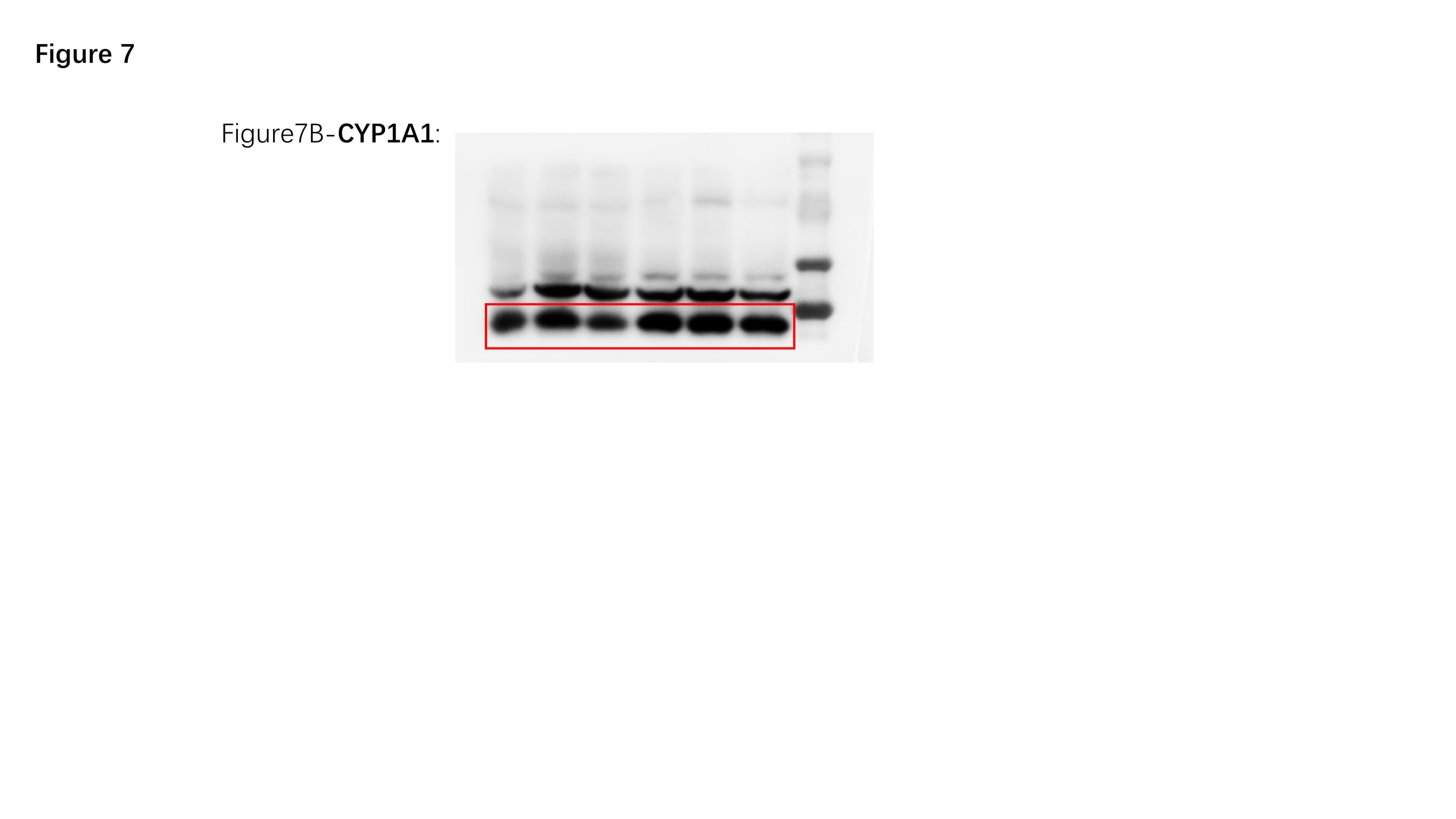

Supplement: Figure 7—source data 1. — (Figure 7B-CYP1A1) The expression of CYP1A1 expression in the placental tissue of Wild Type (The first three columns) and PM2.5-treated mice (the last three columns). (Figure 7B-β-actin) The expression of β-actin expression in the placental tissue of Wild Type (The first three columns) and PM2.5-treated mice (the last three columns). (Figure 7E-CYP1A1) The expression of CYP1A1 expression in the PM2.5-treated HTR8/SVneo cells (PM2.5 concentration: 0. 50 μg/mL, 100 μg/mL, 200 μg/mL). (Figure 7E-β-actin) The expression of β-actin expression in the PM2.5-treated HTR8/SVneo cells (PM2.5 concentration: 0. 50 μg/mL, 100 μg/mL, 200 μg/mL). (Figure 7F-CYP1A1) The expression of CYP1A1 expression in the HTR8/SVneo cells (si-NC, si-CYP1A1#1, si-CYP1A1#2). (Figure 7F-β-actin) The expression of β-actin expression in the HTR8/SVneo cells (si-NC, si-CYP1A1#1, si-CYP1A1#2). (Figure 7M-Mito-Cytc) The expression of Cytc expression in mitochondria of HTR8/SVneo (si-NC, PM2.5+si NC, PM2.5+si- CYP1A1#1, PM2.5+si- CYP1A1#2). (Figure 7M-Mito-VDAC-1) The expression of VDAC-1 expression in mitochondria of HTR8/SVneo (si-NC, PM2.5+si NC, PM2.5+si- CYP1A1#1, PM2.5+si- CYP1A1#2). (Figure 7M-Cyto-Cytc) The expression of Cytc expression in cytoplasm of HTR8/SVneo (si-NC, PM2.5+si NC, PM2.5+si- CYP1A1#1, PM2.5+si- CYP1A1#2). (Figure 7M-Cyto-BCL-2) The expression of BCL-2 expression in cytoplasm of HTR8/SVneo (si-NC, PM2.5+si NC, PM2.5+si- CYP1A1#1, PM2.5+si- CYP1A1#2). (Figure 7M-Cyto-BAX) The expression of BAX expression in cytoplasm of HTR8/SVneo (si-NC, PM2.5+si NC, PM2.5+si- CYP1A1#1, PM2.5+si- CYP1A1#2). (Figure 7M-Cyto-Cleaved-Caspase3) The expression of Cleaved-Caspase3 expression in cytoplasm of HTR8/SVneo (si-NC, PM2.5+si NC, PM2.5+si- CYP1A1#1, PM2.5+si- CYP1A1#2). (Figure 7M-Cyto-β-actin) The expression of β-actin expression in cytoplasm of HTR8/SVneo (si-NC, PM2.5+si NC, PM2.5+si- CYP1A1#1, PM2.5+si- CYP1A1#2). [file elife-85944-fig7-data1.zip › Figure 7-source data 1/Figure7B-CYP1A1.TIF]

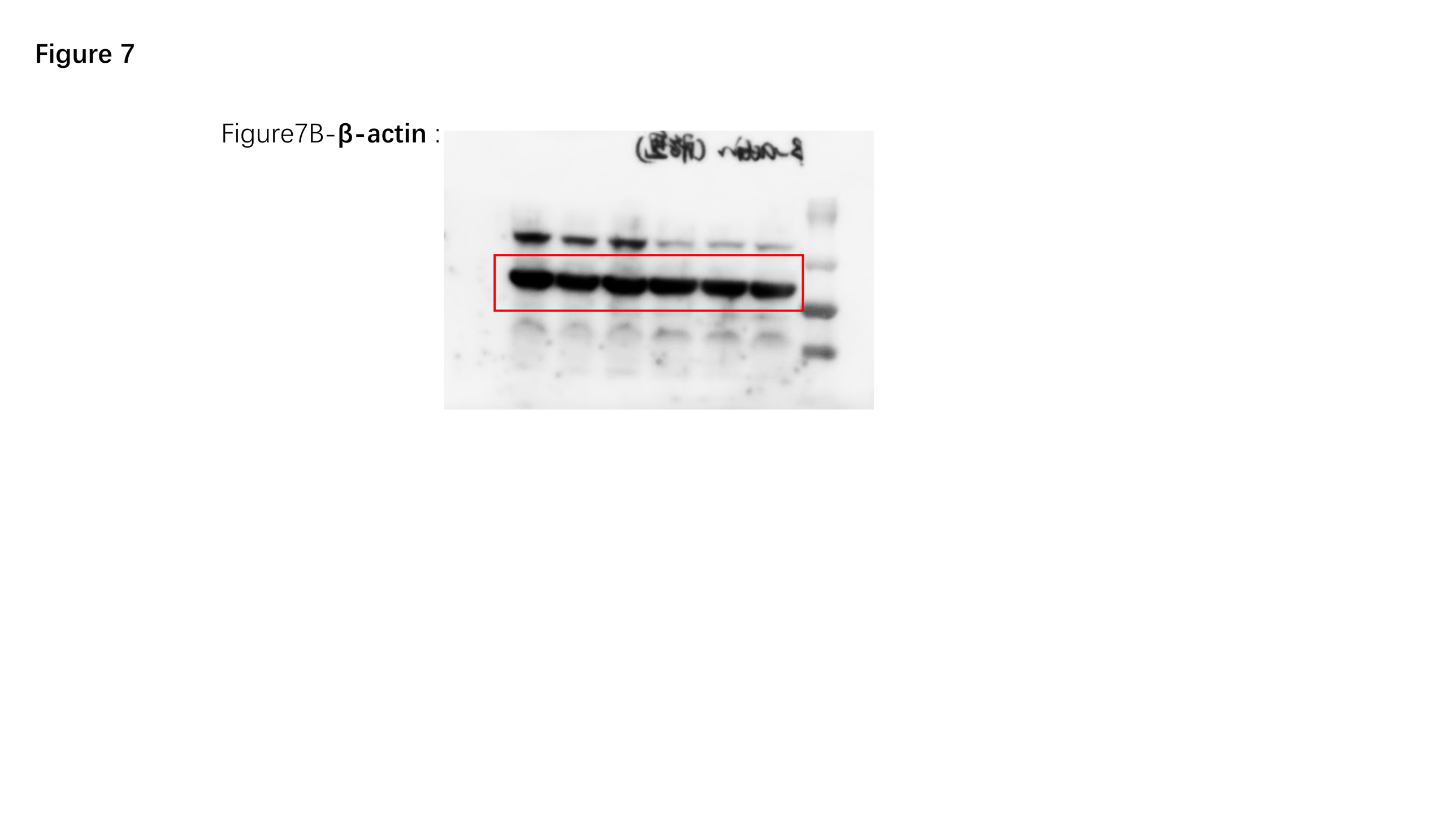

Supplement: Figure 7—source data 1. — (Figure 7B-CYP1A1) The expression of CYP1A1 expression in the placental tissue of Wild Type (The first three columns) and PM2.5-treated mice (the last three columns). (Figure 7B-β-actin) The expression of β-actin expression in the placental tissue of Wild Type (The first three columns) and PM2.5-treated mice (the last three columns). (Figure 7E-CYP1A1) The expression of CYP1A1 expression in the PM2.5-treated HTR8/SVneo cells (PM2.5 concentration: 0. 50 μg/mL, 100 μg/mL, 200 μg/mL). (Figure 7E-β-actin) The expression of β-actin expression in the PM2.5-treated HTR8/SVneo cells (PM2.5 concentration: 0. 50 μg/mL, 100 μg/mL, 200 μg/mL). (Figure 7F-CYP1A1) The expression of CYP1A1 expression in the HTR8/SVneo cells (si-NC, si-CYP1A1#1, si-CYP1A1#2). (Figure 7F-β-actin) The expression of β-actin expression in the HTR8/SVneo cells (si-NC, si-CYP1A1#1, si-CYP1A1#2). (Figure 7M-Mito-Cytc) The expression of Cytc expression in mitochondria of HTR8/SVneo (si-NC, PM2.5+si NC, PM2.5+si- CYP1A1#1, PM2.5+si- CYP1A1#2). (Figure 7M-Mito-VDAC-1) The expression of VDAC-1 expression in mitochondria of HTR8/SVneo (si-NC, PM2.5+si NC, PM2.5+si- CYP1A1#1, PM2.5+si- CYP1A1#2). (Figure 7M-Cyto-Cytc) The expression of Cytc expression in cytoplasm of HTR8/SVneo (si-NC, PM2.5+si NC, PM2.5+si- CYP1A1#1, PM2.5+si- CYP1A1#2). (Figure 7M-Cyto-BCL-2) The expression of BCL-2 expression in cytoplasm of HTR8/SVneo (si-NC, PM2.5+si NC, PM2.5+si- CYP1A1#1, PM2.5+si- CYP1A1#2). (Figure 7M-Cyto-BAX) The expression of BAX expression in cytoplasm of HTR8/SVneo (si-NC, PM2.5+si NC, PM2.5+si- CYP1A1#1, PM2.5+si- CYP1A1#2). (Figure 7M-Cyto-Cleaved-Caspase3) The expression of Cleaved-Caspase3 expression in cytoplasm of HTR8/SVneo (si-NC, PM2.5+si NC, PM2.5+si- CYP1A1#1, PM2.5+si- CYP1A1#2). (Figure 7M-Cyto-β-actin) The expression of β-actin expression in cytoplasm of HTR8/SVneo (si-NC, PM2.5+si NC, PM2.5+si- CYP1A1#1, PM2.5+si- CYP1A1#2). [file elife-85944-fig7-data1.zip › Figure 7-source data 1/Figure7B-a┬-actin.TIF]

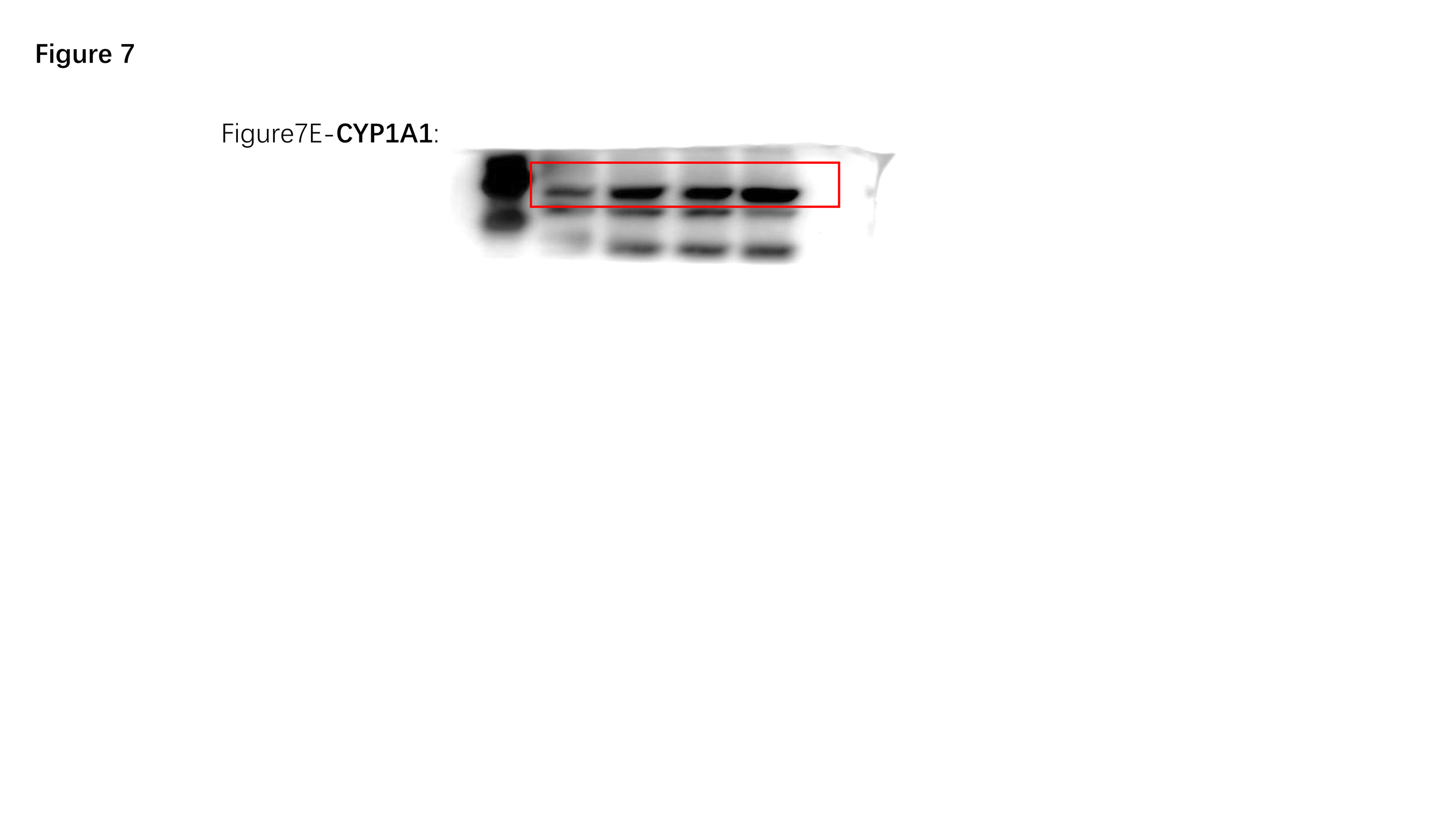

Supplement: Figure 7—source data 1. — (Figure 7B-CYP1A1) The expression of CYP1A1 expression in the placental tissue of Wild Type (The first three columns) and PM2.5-treated mice (the last three columns). (Figure 7B-β-actin) The expression of β-actin expression in the placental tissue of Wild Type (The first three columns) and PM2.5-treated mice (the last three columns). (Figure 7E-CYP1A1) The expression of CYP1A1 expression in the PM2.5-treated HTR8/SVneo cells (PM2.5 concentration: 0. 50 μg/mL, 100 μg/mL, 200 μg/mL). (Figure 7E-β-actin) The expression of β-actin expression in the PM2.5-treated HTR8/SVneo cells (PM2.5 concentration: 0. 50 μg/mL, 100 μg/mL, 200 μg/mL). (Figure 7F-CYP1A1) The expression of CYP1A1 expression in the HTR8/SVneo cells (si-NC, si-CYP1A1#1, si-CYP1A1#2). (Figure 7F-β-actin) The expression of β-actin expression in the HTR8/SVneo cells (si-NC, si-CYP1A1#1, si-CYP1A1#2). (Figure 7M-Mito-Cytc) The expression of Cytc expression in mitochondria of HTR8/SVneo (si-NC, PM2.5+si NC, PM2.5+si- CYP1A1#1, PM2.5+si- CYP1A1#2). (Figure 7M-Mito-VDAC-1) The expression of VDAC-1 expression in mitochondria of HTR8/SVneo (si-NC, PM2.5+si NC, PM2.5+si- CYP1A1#1, PM2.5+si- CYP1A1#2). (Figure 7M-Cyto-Cytc) The expression of Cytc expression in cytoplasm of HTR8/SVneo (si-NC, PM2.5+si NC, PM2.5+si- CYP1A1#1, PM2.5+si- CYP1A1#2). (Figure 7M-Cyto-BCL-2) The expression of BCL-2 expression in cytoplasm of HTR8/SVneo (si-NC, PM2.5+si NC, PM2.5+si- CYP1A1#1, PM2.5+si- CYP1A1#2). (Figure 7M-Cyto-BAX) The expression of BAX expression in cytoplasm of HTR8/SVneo (si-NC, PM2.5+si NC, PM2.5+si- CYP1A1#1, PM2.5+si- CYP1A1#2). (Figure 7M-Cyto-Cleaved-Caspase3) The expression of Cleaved-Caspase3 expression in cytoplasm of HTR8/SVneo (si-NC, PM2.5+si NC, PM2.5+si- CYP1A1#1, PM2.5+si- CYP1A1#2). (Figure 7M-Cyto-β-actin) The expression of β-actin expression in cytoplasm of HTR8/SVneo (si-NC, PM2.5+si NC, PM2.5+si- CYP1A1#1, PM2.5+si- CYP1A1#2). [file elife-85944-fig7-data1.zip › Figure 7-source data 1/Figure7E-CYP1A1.TIF]

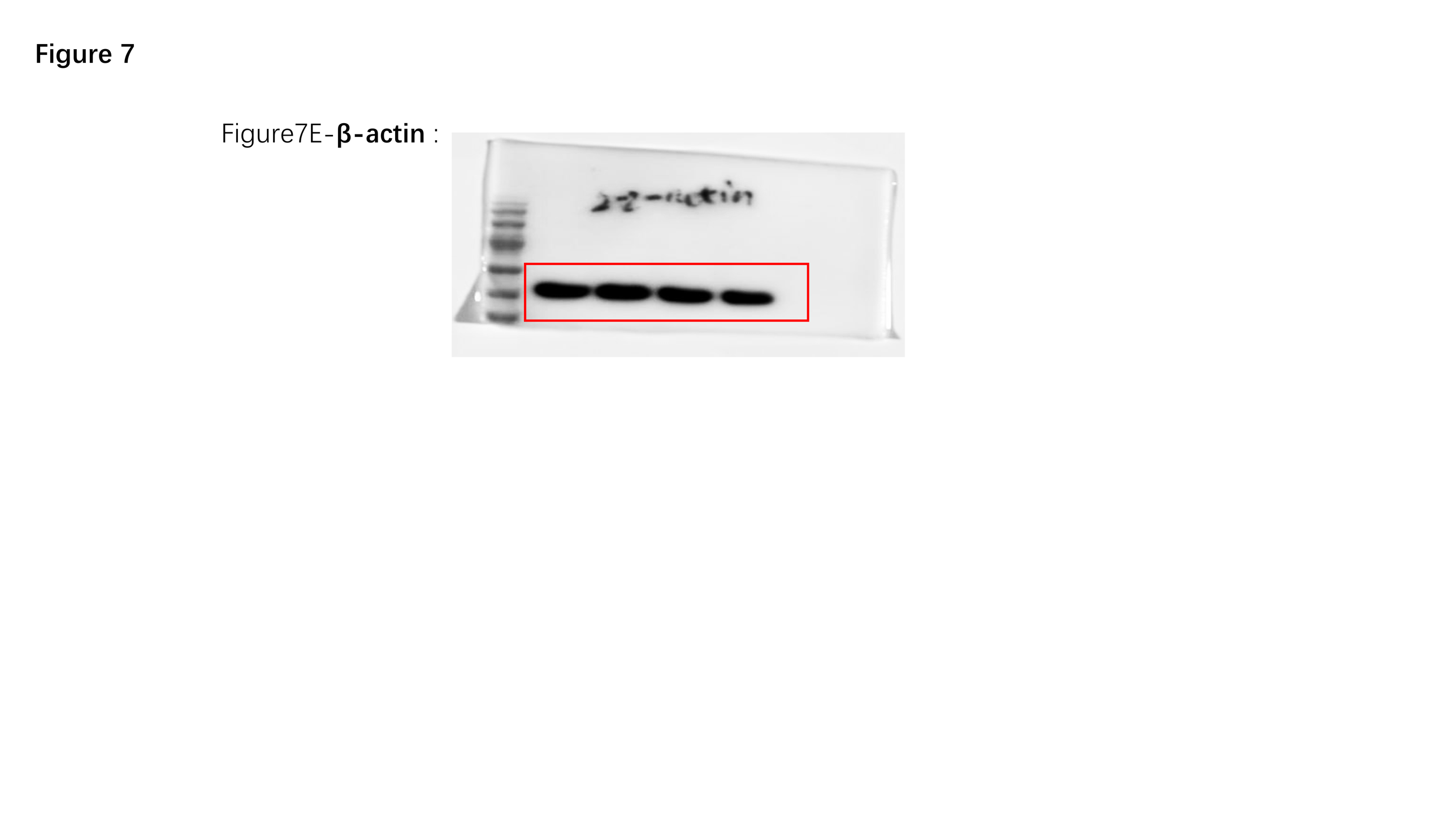

Supplement: Figure 7—source data 1. — (Figure 7B-CYP1A1) The expression of CYP1A1 expression in the placental tissue of Wild Type (The first three columns) and PM2.5-treated mice (the last three columns). (Figure 7B-β-actin) The expression of β-actin expression in the placental tissue of Wild Type (The first three columns) and PM2.5-treated mice (the last three columns). (Figure 7E-CYP1A1) The expression of CYP1A1 expression in the PM2.5-treated HTR8/SVneo cells (PM2.5 concentration: 0. 50 μg/mL, 100 μg/mL, 200 μg/mL). (Figure 7E-β-actin) The expression of β-actin expression in the PM2.5-treated HTR8/SVneo cells (PM2.5 concentration: 0. 50 μg/mL, 100 μg/mL, 200 μg/mL). (Figure 7F-CYP1A1) The expression of CYP1A1 expression in the HTR8/SVneo cells (si-NC, si-CYP1A1#1, si-CYP1A1#2). (Figure 7F-β-actin) The expression of β-actin expression in the HTR8/SVneo cells (si-NC, si-CYP1A1#1, si-CYP1A1#2). (Figure 7M-Mito-Cytc) The expression of Cytc expression in mitochondria of HTR8/SVneo (si-NC, PM2.5+si NC, PM2.5+si- CYP1A1#1, PM2.5+si- CYP1A1#2). (Figure 7M-Mito-VDAC-1) The expression of VDAC-1 expression in mitochondria of HTR8/SVneo (si-NC, PM2.5+si NC, PM2.5+si- CYP1A1#1, PM2.5+si- CYP1A1#2). (Figure 7M-Cyto-Cytc) The expression of Cytc expression in cytoplasm of HTR8/SVneo (si-NC, PM2.5+si NC, PM2.5+si- CYP1A1#1, PM2.5+si- CYP1A1#2). (Figure 7M-Cyto-BCL-2) The expression of BCL-2 expression in cytoplasm of HTR8/SVneo (si-NC, PM2.5+si NC, PM2.5+si- CYP1A1#1, PM2.5+si- CYP1A1#2). (Figure 7M-Cyto-BAX) The expression of BAX expression in cytoplasm of HTR8/SVneo (si-NC, PM2.5+si NC, PM2.5+si- CYP1A1#1, PM2.5+si- CYP1A1#2). (Figure 7M-Cyto-Cleaved-Caspase3) The expression of Cleaved-Caspase3 expression in cytoplasm of HTR8/SVneo (si-NC, PM2.5+si NC, PM2.5+si- CYP1A1#1, PM2.5+si- CYP1A1#2). (Figure 7M-Cyto-β-actin) The expression of β-actin expression in cytoplasm of HTR8/SVneo (si-NC, PM2.5+si NC, PM2.5+si- CYP1A1#1, PM2.5+si- CYP1A1#2). [file elife-85944-fig7-data1.zip › Figure 7-source data 1/Figure7E-a┬-actin.TIF]

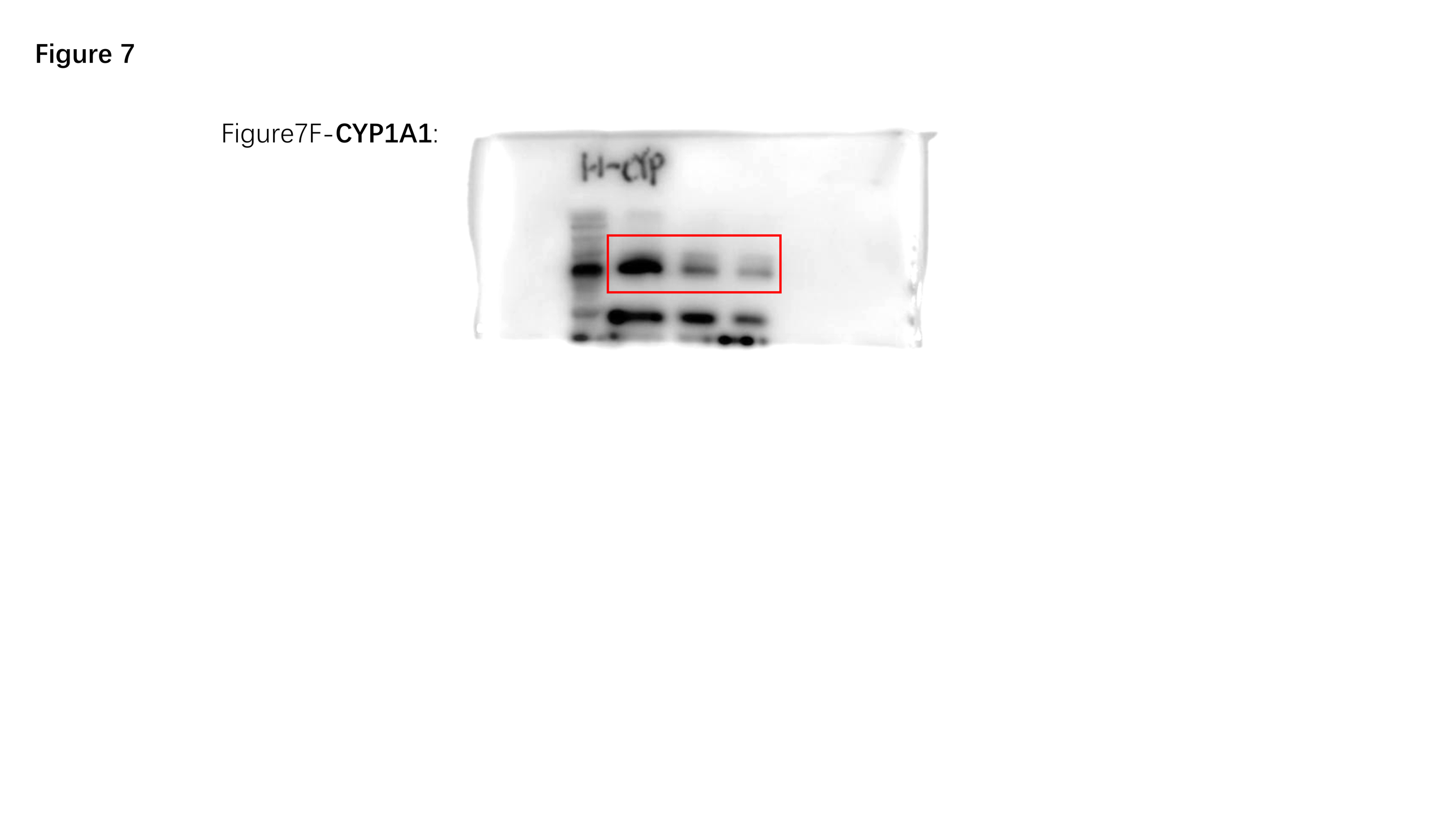

Supplement: Figure 7—source data 1. — (Figure 7B-CYP1A1) The expression of CYP1A1 expression in the placental tissue of Wild Type (The first three columns) and PM2.5-treated mice (the last three columns). (Figure 7B-β-actin) The expression of β-actin expression in the placental tissue of Wild Type (The first three columns) and PM2.5-treated mice (the last three columns). (Figure 7E-CYP1A1) The expression of CYP1A1 expression in the PM2.5-treated HTR8/SVneo cells (PM2.5 concentration: 0. 50 μg/mL, 100 μg/mL, 200 μg/mL). (Figure 7E-β-actin) The expression of β-actin expression in the PM2.5-treated HTR8/SVneo cells (PM2.5 concentration: 0. 50 μg/mL, 100 μg/mL, 200 μg/mL). (Figure 7F-CYP1A1) The expression of CYP1A1 expression in the HTR8/SVneo cells (si-NC, si-CYP1A1#1, si-CYP1A1#2). (Figure 7F-β-actin) The expression of β-actin expression in the HTR8/SVneo cells (si-NC, si-CYP1A1#1, si-CYP1A1#2). (Figure 7M-Mito-Cytc) The expression of Cytc expression in mitochondria of HTR8/SVneo (si-NC, PM2.5+si NC, PM2.5+si- CYP1A1#1, PM2.5+si- CYP1A1#2). (Figure 7M-Mito-VDAC-1) The expression of VDAC-1 expression in mitochondria of HTR8/SVneo (si-NC, PM2.5+si NC, PM2.5+si- CYP1A1#1, PM2.5+si- CYP1A1#2). (Figure 7M-Cyto-Cytc) The expression of Cytc expression in cytoplasm of HTR8/SVneo (si-NC, PM2.5+si NC, PM2.5+si- CYP1A1#1, PM2.5+si- CYP1A1#2). (Figure 7M-Cyto-BCL-2) The expression of BCL-2 expression in cytoplasm of HTR8/SVneo (si-NC, PM2.5+si NC, PM2.5+si- CYP1A1#1, PM2.5+si- CYP1A1#2). (Figure 7M-Cyto-BAX) The expression of BAX expression in cytoplasm of HTR8/SVneo (si-NC, PM2.5+si NC, PM2.5+si- CYP1A1#1, PM2.5+si- CYP1A1#2). (Figure 7M-Cyto-Cleaved-Caspase3) The expression of Cleaved-Caspase3 expression in cytoplasm of HTR8/SVneo (si-NC, PM2.5+si NC, PM2.5+si- CYP1A1#1, PM2.5+si- CYP1A1#2). (Figure 7M-Cyto-β-actin) The expression of β-actin expression in cytoplasm of HTR8/SVneo (si-NC, PM2.5+si NC, PM2.5+si- CYP1A1#1, PM2.5+si- CYP1A1#2). [file elife-85944-fig7-data1.zip › Figure 7-source data 1/Figure7F-CYP1A1.TIF]

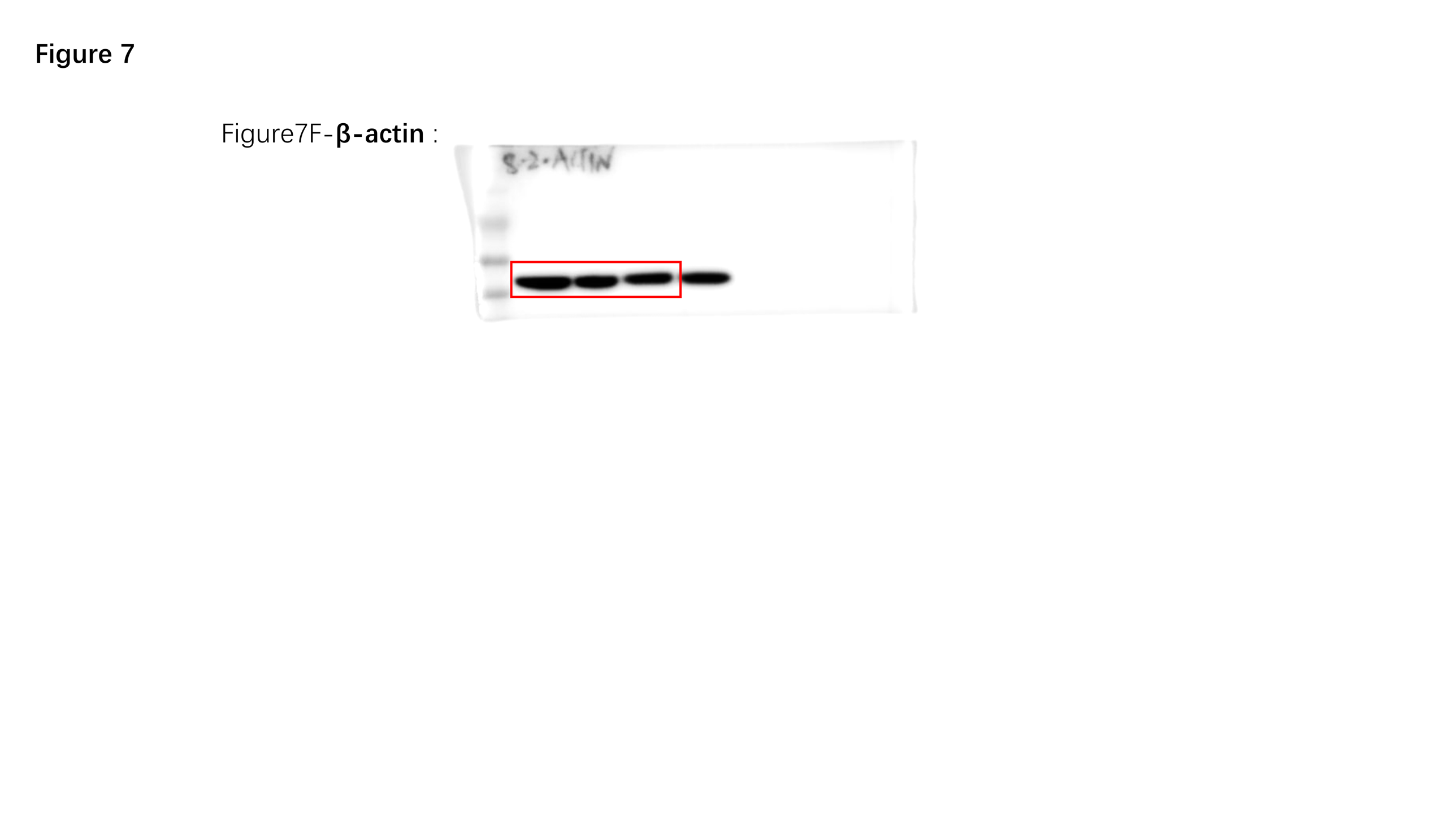

Supplement: Figure 7—source data 1. — (Figure 7B-CYP1A1) The expression of CYP1A1 expression in the placental tissue of Wild Type (The first three columns) and PM2.5-treated mice (the last three columns). (Figure 7B-β-actin) The expression of β-actin expression in the placental tissue of Wild Type (The first three columns) and PM2.5-treated mice (the last three columns). (Figure 7E-CYP1A1) The expression of CYP1A1 expression in the PM2.5-treated HTR8/SVneo cells (PM2.5 concentration: 0. 50 μg/mL, 100 μg/mL, 200 μg/mL). (Figure 7E-β-actin) The expression of β-actin expression in the PM2.5-treated HTR8/SVneo cells (PM2.5 concentration: 0. 50 μg/mL, 100 μg/mL, 200 μg/mL). (Figure 7F-CYP1A1) The expression of CYP1A1 expression in the HTR8/SVneo cells (si-NC, si-CYP1A1#1, si-CYP1A1#2). (Figure 7F-β-actin) The expression of β-actin expression in the HTR8/SVneo cells (si-NC, si-CYP1A1#1, si-CYP1A1#2). (Figure 7M-Mito-Cytc) The expression of Cytc expression in mitochondria of HTR8/SVneo (si-NC, PM2.5+si NC, PM2.5+si- CYP1A1#1, PM2.5+si- CYP1A1#2). (Figure 7M-Mito-VDAC-1) The expression of VDAC-1 expression in mitochondria of HTR8/SVneo (si-NC, PM2.5+si NC, PM2.5+si- CYP1A1#1, PM2.5+si- CYP1A1#2). (Figure 7M-Cyto-Cytc) The expression of Cytc expression in cytoplasm of HTR8/SVneo (si-NC, PM2.5+si NC, PM2.5+si- CYP1A1#1, PM2.5+si- CYP1A1#2). (Figure 7M-Cyto-BCL-2) The expression of BCL-2 expression in cytoplasm of HTR8/SVneo (si-NC, PM2.5+si NC, PM2.5+si- CYP1A1#1, PM2.5+si- CYP1A1#2). (Figure 7M-Cyto-BAX) The expression of BAX expression in cytoplasm of HTR8/SVneo (si-NC, PM2.5+si NC, PM2.5+si- CYP1A1#1, PM2.5+si- CYP1A1#2). (Figure 7M-Cyto-Cleaved-Caspase3) The expression of Cleaved-Caspase3 expression in cytoplasm of HTR8/SVneo (si-NC, PM2.5+si NC, PM2.5+si- CYP1A1#1, PM2.5+si- CYP1A1#2). (Figure 7M-Cyto-β-actin) The expression of β-actin expression in cytoplasm of HTR8/SVneo (si-NC, PM2.5+si NC, PM2.5+si- CYP1A1#1, PM2.5+si- CYP1A1#2). [file elife-85944-fig7-data1.zip › Figure 7-source data 1/Figure7F-a┬-actin.TIF]

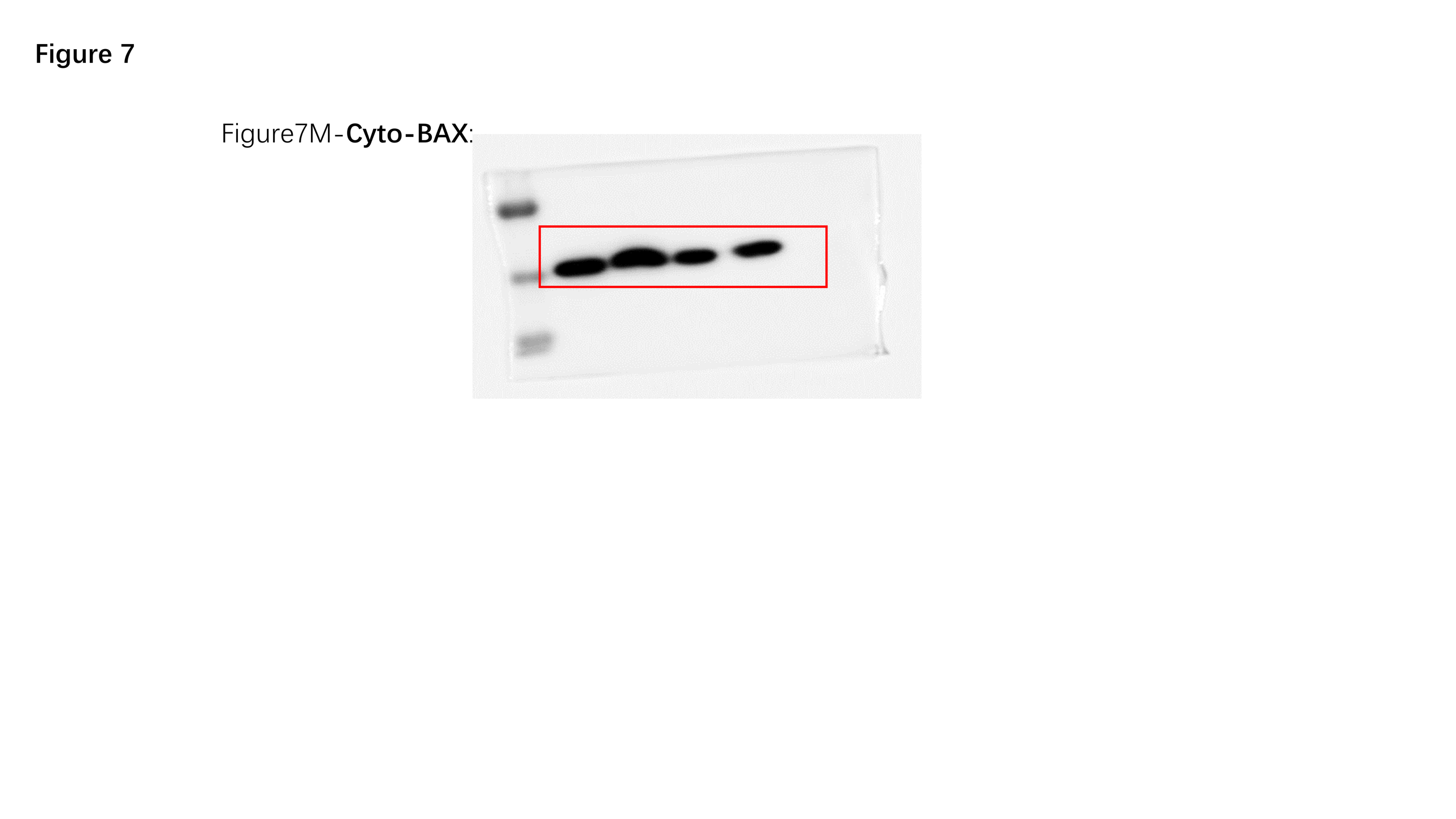

Supplement: Figure 7—source data 1. — (Figure 7B-CYP1A1) The expression of CYP1A1 expression in the placental tissue of Wild Type (The first three columns) and PM2.5-treated mice (the last three columns). (Figure 7B-β-actin) The expression of β-actin expression in the placental tissue of Wild Type (The first three columns) and PM2.5-treated mice (the last three columns). (Figure 7E-CYP1A1) The expression of CYP1A1 expression in the PM2.5-treated HTR8/SVneo cells (PM2.5 concentration: 0. 50 μg/mL, 100 μg/mL, 200 μg/mL). (Figure 7E-β-actin) The expression of β-actin expression in the PM2.5-treated HTR8/SVneo cells (PM2.5 concentration: 0. 50 μg/mL, 100 μg/mL, 200 μg/mL). (Figure 7F-CYP1A1) The expression of CYP1A1 expression in the HTR8/SVneo cells (si-NC, si-CYP1A1#1, si-CYP1A1#2). (Figure 7F-β-actin) The expression of β-actin expression in the HTR8/SVneo cells (si-NC, si-CYP1A1#1, si-CYP1A1#2). (Figure 7M-Mito-Cytc) The expression of Cytc expression in mitochondria of HTR8/SVneo (si-NC, PM2.5+si NC, PM2.5+si- CYP1A1#1, PM2.5+si- CYP1A1#2). (Figure 7M-Mito-VDAC-1) The expression of VDAC-1 expression in mitochondria of HTR8/SVneo (si-NC, PM2.5+si NC, PM2.5+si- CYP1A1#1, PM2.5+si- CYP1A1#2). (Figure 7M-Cyto-Cytc) The expression of Cytc expression in cytoplasm of HTR8/SVneo (si-NC, PM2.5+si NC, PM2.5+si- CYP1A1#1, PM2.5+si- CYP1A1#2). (Figure 7M-Cyto-BCL-2) The expression of BCL-2 expression in cytoplasm of HTR8/SVneo (si-NC, PM2.5+si NC, PM2.5+si- CYP1A1#1, PM2.5+si- CYP1A1#2). (Figure 7M-Cyto-BAX) The expression of BAX expression in cytoplasm of HTR8/SVneo (si-NC, PM2.5+si NC, PM2.5+si- CYP1A1#1, PM2.5+si- CYP1A1#2). (Figure 7M-Cyto-Cleaved-Caspase3) The expression of Cleaved-Caspase3 expression in cytoplasm of HTR8/SVneo (si-NC, PM2.5+si NC, PM2.5+si- CYP1A1#1, PM2.5+si- CYP1A1#2). (Figure 7M-Cyto-β-actin) The expression of β-actin expression in cytoplasm of HTR8/SVneo (si-NC, PM2.5+si NC, PM2.5+si- CYP1A1#1, PM2.5+si- CYP1A1#2). [file elife-85944-fig7-data1.zip › Figure 7-source data 1/Figure7M-Cyto-BAX.TIF]

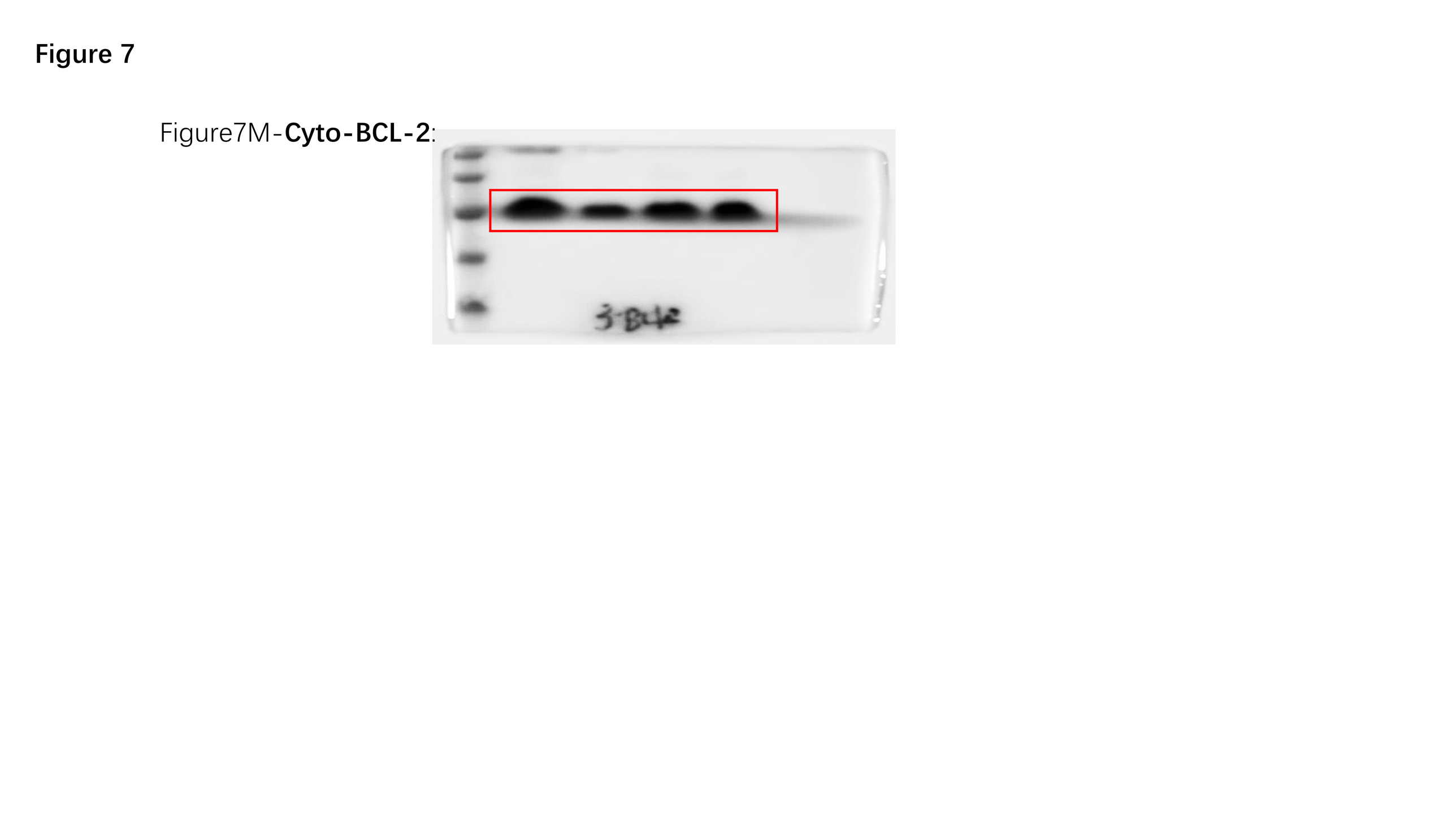

Supplement: Figure 7—source data 1. — (Figure 7B-CYP1A1) The expression of CYP1A1 expression in the placental tissue of Wild Type (The first three columns) and PM2.5-treated mice (the last three columns). (Figure 7B-β-actin) The expression of β-actin expression in the placental tissue of Wild Type (The first three columns) and PM2.5-treated mice (the last three columns). (Figure 7E-CYP1A1) The expression of CYP1A1 expression in the PM2.5-treated HTR8/SVneo cells (PM2.5 concentration: 0. 50 μg/mL, 100 μg/mL, 200 μg/mL). (Figure 7E-β-actin) The expression of β-actin expression in the PM2.5-treated HTR8/SVneo cells (PM2.5 concentration: 0. 50 μg/mL, 100 μg/mL, 200 μg/mL). (Figure 7F-CYP1A1) The expression of CYP1A1 expression in the HTR8/SVneo cells (si-NC, si-CYP1A1#1, si-CYP1A1#2). (Figure 7F-β-actin) The expression of β-actin expression in the HTR8/SVneo cells (si-NC, si-CYP1A1#1, si-CYP1A1#2). (Figure 7M-Mito-Cytc) The expression of Cytc expression in mitochondria of HTR8/SVneo (si-NC, PM2.5+si NC, PM2.5+si- CYP1A1#1, PM2.5+si- CYP1A1#2). (Figure 7M-Mito-VDAC-1) The expression of VDAC-1 expression in mitochondria of HTR8/SVneo (si-NC, PM2.5+si NC, PM2.5+si- CYP1A1#1, PM2.5+si- CYP1A1#2). (Figure 7M-Cyto-Cytc) The expression of Cytc expression in cytoplasm of HTR8/SVneo (si-NC, PM2.5+si NC, PM2.5+si- CYP1A1#1, PM2.5+si- CYP1A1#2). (Figure 7M-Cyto-BCL-2) The expression of BCL-2 expression in cytoplasm of HTR8/SVneo (si-NC, PM2.5+si NC, PM2.5+si- CYP1A1#1, PM2.5+si- CYP1A1#2). (Figure 7M-Cyto-BAX) The expression of BAX expression in cytoplasm of HTR8/SVneo (si-NC, PM2.5+si NC, PM2.5+si- CYP1A1#1, PM2.5+si- CYP1A1#2). (Figure 7M-Cyto-Cleaved-Caspase3) The expression of Cleaved-Caspase3 expression in cytoplasm of HTR8/SVneo (si-NC, PM2.5+si NC, PM2.5+si- CYP1A1#1, PM2.5+si- CYP1A1#2). (Figure 7M-Cyto-β-actin) The expression of β-actin expression in cytoplasm of HTR8/SVneo (si-NC, PM2.5+si NC, PM2.5+si- CYP1A1#1, PM2.5+si- CYP1A1#2). [file elife-85944-fig7-data1.zip › Figure 7-source data 1/Figure7M-Cyto-BCL-2.TIF]

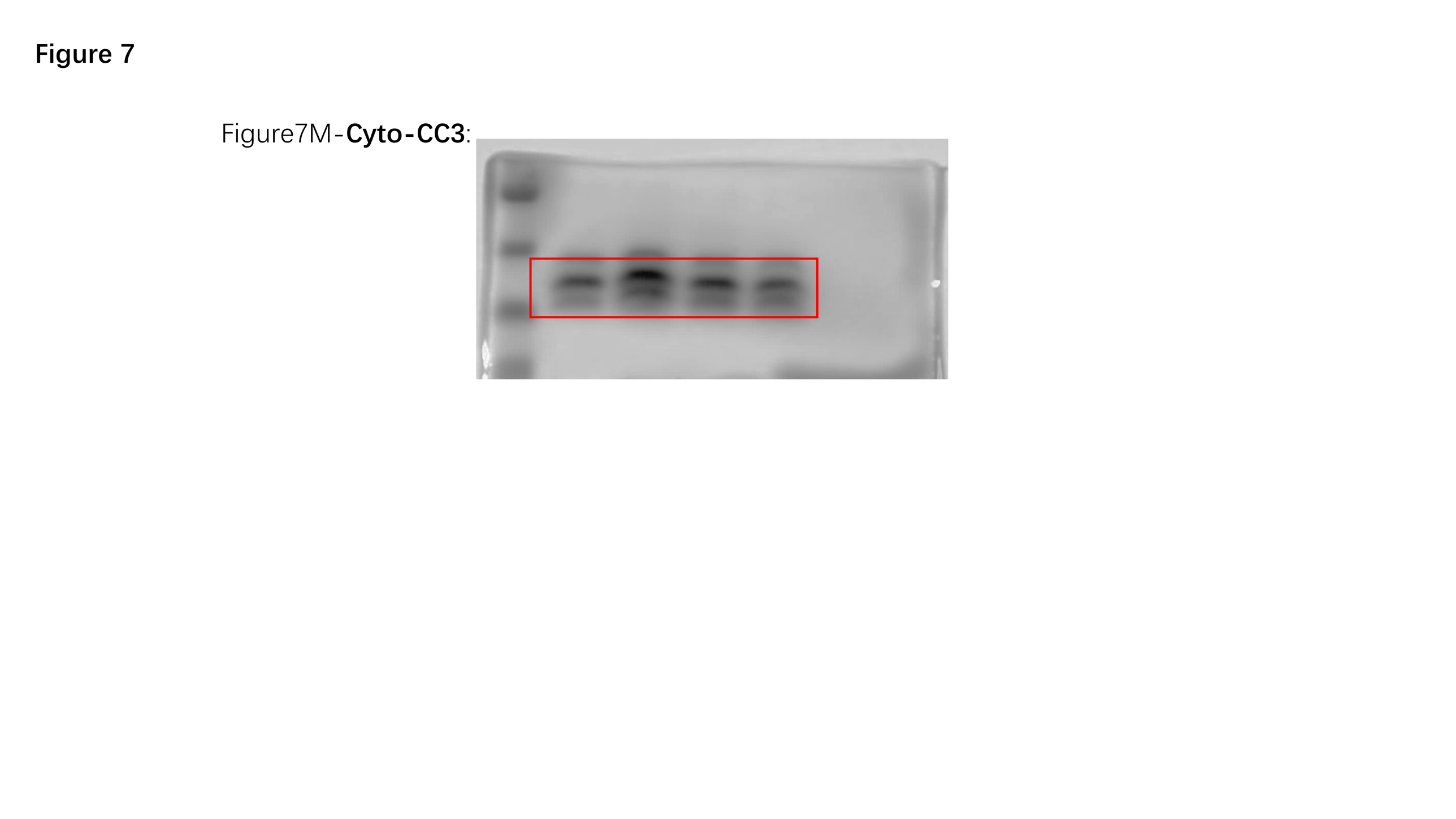

Supplement: Figure 7—source data 1. — (Figure 7B-CYP1A1) The expression of CYP1A1 expression in the placental tissue of Wild Type (The first three columns) and PM2.5-treated mice (the last three columns). (Figure 7B-β-actin) The expression of β-actin expression in the placental tissue of Wild Type (The first three columns) and PM2.5-treated mice (the last three columns). (Figure 7E-CYP1A1) The expression of CYP1A1 expression in the PM2.5-treated HTR8/SVneo cells (PM2.5 concentration: 0. 50 μg/mL, 100 μg/mL, 200 μg/mL). (Figure 7E-β-actin) The expression of β-actin expression in the PM2.5-treated HTR8/SVneo cells (PM2.5 concentration: 0. 50 μg/mL, 100 μg/mL, 200 μg/mL). (Figure 7F-CYP1A1) The expression of CYP1A1 expression in the HTR8/SVneo cells (si-NC, si-CYP1A1#1, si-CYP1A1#2). (Figure 7F-β-actin) The expression of β-actin expression in the HTR8/SVneo cells (si-NC, si-CYP1A1#1, si-CYP1A1#2). (Figure 7M-Mito-Cytc) The expression of Cytc expression in mitochondria of HTR8/SVneo (si-NC, PM2.5+si NC, PM2.5+si- CYP1A1#1, PM2.5+si- CYP1A1#2). (Figure 7M-Mito-VDAC-1) The expression of VDAC-1 expression in mitochondria of HTR8/SVneo (si-NC, PM2.5+si NC, PM2.5+si- CYP1A1#1, PM2.5+si- CYP1A1#2). (Figure 7M-Cyto-Cytc) The expression of Cytc expression in cytoplasm of HTR8/SVneo (si-NC, PM2.5+si NC, PM2.5+si- CYP1A1#1, PM2.5+si- CYP1A1#2). (Figure 7M-Cyto-BCL-2) The expression of BCL-2 expression in cytoplasm of HTR8/SVneo (si-NC, PM2.5+si NC, PM2.5+si- CYP1A1#1, PM2.5+si- CYP1A1#2). (Figure 7M-Cyto-BAX) The expression of BAX expression in cytoplasm of HTR8/SVneo (si-NC, PM2.5+si NC, PM2.5+si- CYP1A1#1, PM2.5+si- CYP1A1#2). (Figure 7M-Cyto-Cleaved-Caspase3) The expression of Cleaved-Caspase3 expression in cytoplasm of HTR8/SVneo (si-NC, PM2.5+si NC, PM2.5+si- CYP1A1#1, PM2.5+si- CYP1A1#2). (Figure 7M-Cyto-β-actin) The expression of β-actin expression in cytoplasm of HTR8/SVneo (si-NC, PM2.5+si NC, PM2.5+si- CYP1A1#1, PM2.5+si- CYP1A1#2). [file elife-85944-fig7-data1.zip › Figure 7-source data 1/Figure7M-Cyto-CC3.TIF]

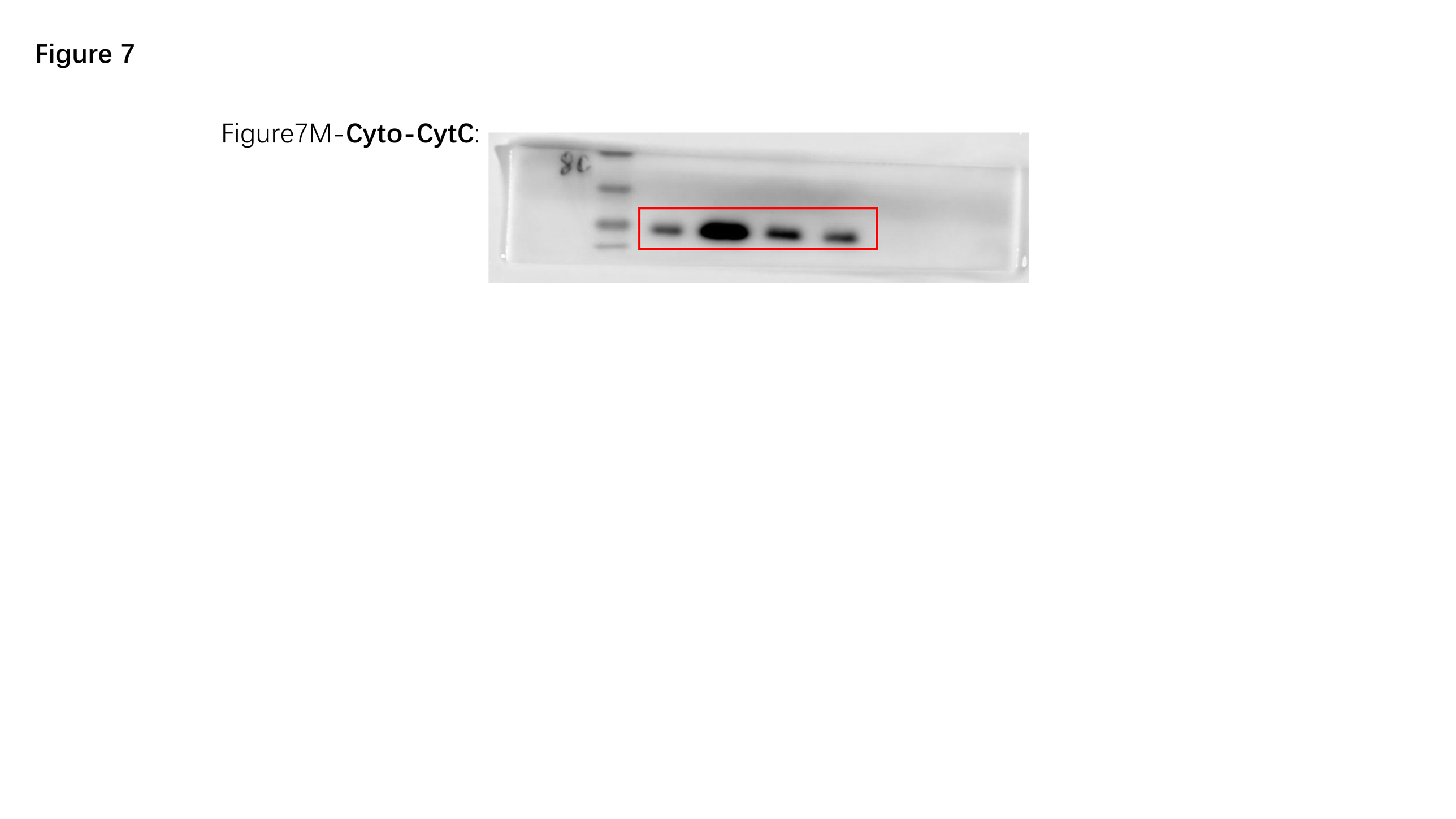

Supplement: Figure 7—source data 1. — (Figure 7B-CYP1A1) The expression of CYP1A1 expression in the placental tissue of Wild Type (The first three columns) and PM2.5-treated mice (the last three columns). (Figure 7B-β-actin) The expression of β-actin expression in the placental tissue of Wild Type (The first three columns) and PM2.5-treated mice (the last three columns). (Figure 7E-CYP1A1) The expression of CYP1A1 expression in the PM2.5-treated HTR8/SVneo cells (PM2.5 concentration: 0. 50 μg/mL, 100 μg/mL, 200 μg/mL). (Figure 7E-β-actin) The expression of β-actin expression in the PM2.5-treated HTR8/SVneo cells (PM2.5 concentration: 0. 50 μg/mL, 100 μg/mL, 200 μg/mL). (Figure 7F-CYP1A1) The expression of CYP1A1 expression in the HTR8/SVneo cells (si-NC, si-CYP1A1#1, si-CYP1A1#2). (Figure 7F-β-actin) The expression of β-actin expression in the HTR8/SVneo cells (si-NC, si-CYP1A1#1, si-CYP1A1#2). (Figure 7M-Mito-Cytc) The expression of Cytc expression in mitochondria of HTR8/SVneo (si-NC, PM2.5+si NC, PM2.5+si- CYP1A1#1, PM2.5+si- CYP1A1#2). (Figure 7M-Mito-VDAC-1) The expression of VDAC-1 expression in mitochondria of HTR8/SVneo (si-NC, PM2.5+si NC, PM2.5+si- CYP1A1#1, PM2.5+si- CYP1A1#2). (Figure 7M-Cyto-Cytc) The expression of Cytc expression in cytoplasm of HTR8/SVneo (si-NC, PM2.5+si NC, PM2.5+si- CYP1A1#1, PM2.5+si- CYP1A1#2). (Figure 7M-Cyto-BCL-2) The expression of BCL-2 expression in cytoplasm of HTR8/SVneo (si-NC, PM2.5+si NC, PM2.5+si- CYP1A1#1, PM2.5+si- CYP1A1#2). (Figure 7M-Cyto-BAX) The expression of BAX expression in cytoplasm of HTR8/SVneo (si-NC, PM2.5+si NC, PM2.5+si- CYP1A1#1, PM2.5+si- CYP1A1#2). (Figure 7M-Cyto-Cleaved-Caspase3) The expression of Cleaved-Caspase3 expression in cytoplasm of HTR8/SVneo (si-NC, PM2.5+si NC, PM2.5+si- CYP1A1#1, PM2.5+si- CYP1A1#2). (Figure 7M-Cyto-β-actin) The expression of β-actin expression in cytoplasm of HTR8/SVneo (si-NC, PM2.5+si NC, PM2.5+si- CYP1A1#1, PM2.5+si- CYP1A1#2). [file elife-85944-fig7-data1.zip › Figure 7-source data 1/Figure7M-Cyto-CytC.TIF]

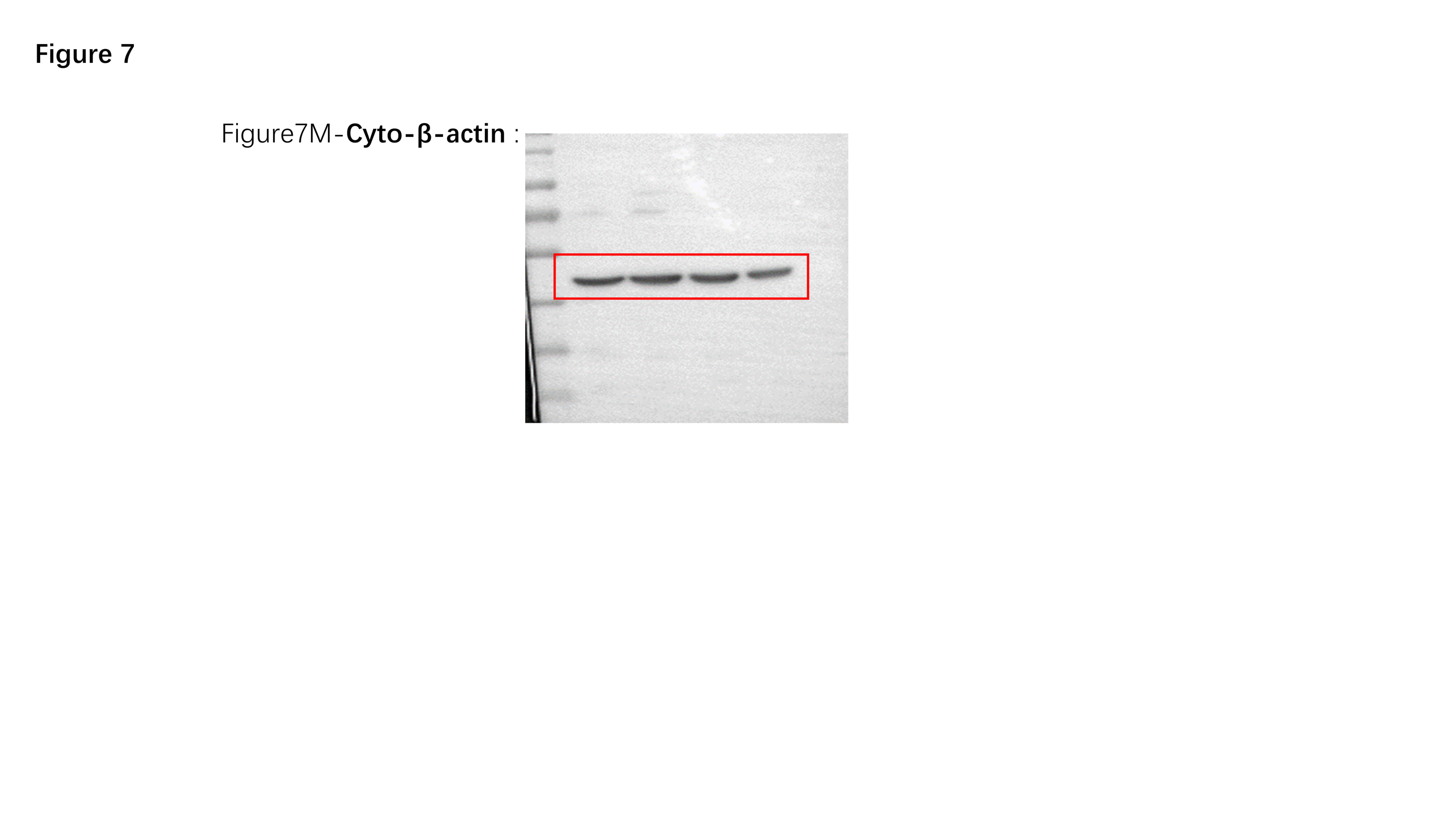

Supplement: Figure 7—source data 1. — (Figure 7B-CYP1A1) The expression of CYP1A1 expression in the placental tissue of Wild Type (The first three columns) and PM2.5-treated mice (the last three columns). (Figure 7B-β-actin) The expression of β-actin expression in the placental tissue of Wild Type (The first three columns) and PM2.5-treated mice (the last three columns). (Figure 7E-CYP1A1) The expression of CYP1A1 expression in the PM2.5-treated HTR8/SVneo cells (PM2.5 concentration: 0. 50 μg/mL, 100 μg/mL, 200 μg/mL). (Figure 7E-β-actin) The expression of β-actin expression in the PM2.5-treated HTR8/SVneo cells (PM2.5 concentration: 0. 50 μg/mL, 100 μg/mL, 200 μg/mL). (Figure 7F-CYP1A1) The expression of CYP1A1 expression in the HTR8/SVneo cells (si-NC, si-CYP1A1#1, si-CYP1A1#2). (Figure 7F-β-actin) The expression of β-actin expression in the HTR8/SVneo cells (si-NC, si-CYP1A1#1, si-CYP1A1#2). (Figure 7M-Mito-Cytc) The expression of Cytc expression in mitochondria of HTR8/SVneo (si-NC, PM2.5+si NC, PM2.5+si- CYP1A1#1, PM2.5+si- CYP1A1#2). (Figure 7M-Mito-VDAC-1) The expression of VDAC-1 expression in mitochondria of HTR8/SVneo (si-NC, PM2.5+si NC, PM2.5+si- CYP1A1#1, PM2.5+si- CYP1A1#2). (Figure 7M-Cyto-Cytc) The expression of Cytc expression in cytoplasm of HTR8/SVneo (si-NC, PM2.5+si NC, PM2.5+si- CYP1A1#1, PM2.5+si- CYP1A1#2). (Figure 7M-Cyto-BCL-2) The expression of BCL-2 expression in cytoplasm of HTR8/SVneo (si-NC, PM2.5+si NC, PM2.5+si- CYP1A1#1, PM2.5+si- CYP1A1#2). (Figure 7M-Cyto-BAX) The expression of BAX expression in cytoplasm of HTR8/SVneo (si-NC, PM2.5+si NC, PM2.5+si- CYP1A1#1, PM2.5+si- CYP1A1#2). (Figure 7M-Cyto-Cleaved-Caspase3) The expression of Cleaved-Caspase3 expression in cytoplasm of HTR8/SVneo (si-NC, PM2.5+si NC, PM2.5+si- CYP1A1#1, PM2.5+si- CYP1A1#2). (Figure 7M-Cyto-β-actin) The expression of β-actin expression in cytoplasm of HTR8/SVneo (si-NC, PM2.5+si NC, PM2.5+si- CYP1A1#1, PM2.5+si- CYP1A1#2). [file elife-85944-fig7-data1.zip › Figure 7-source data 1/Figure7M-Cyto-a┬-actin.TIF]

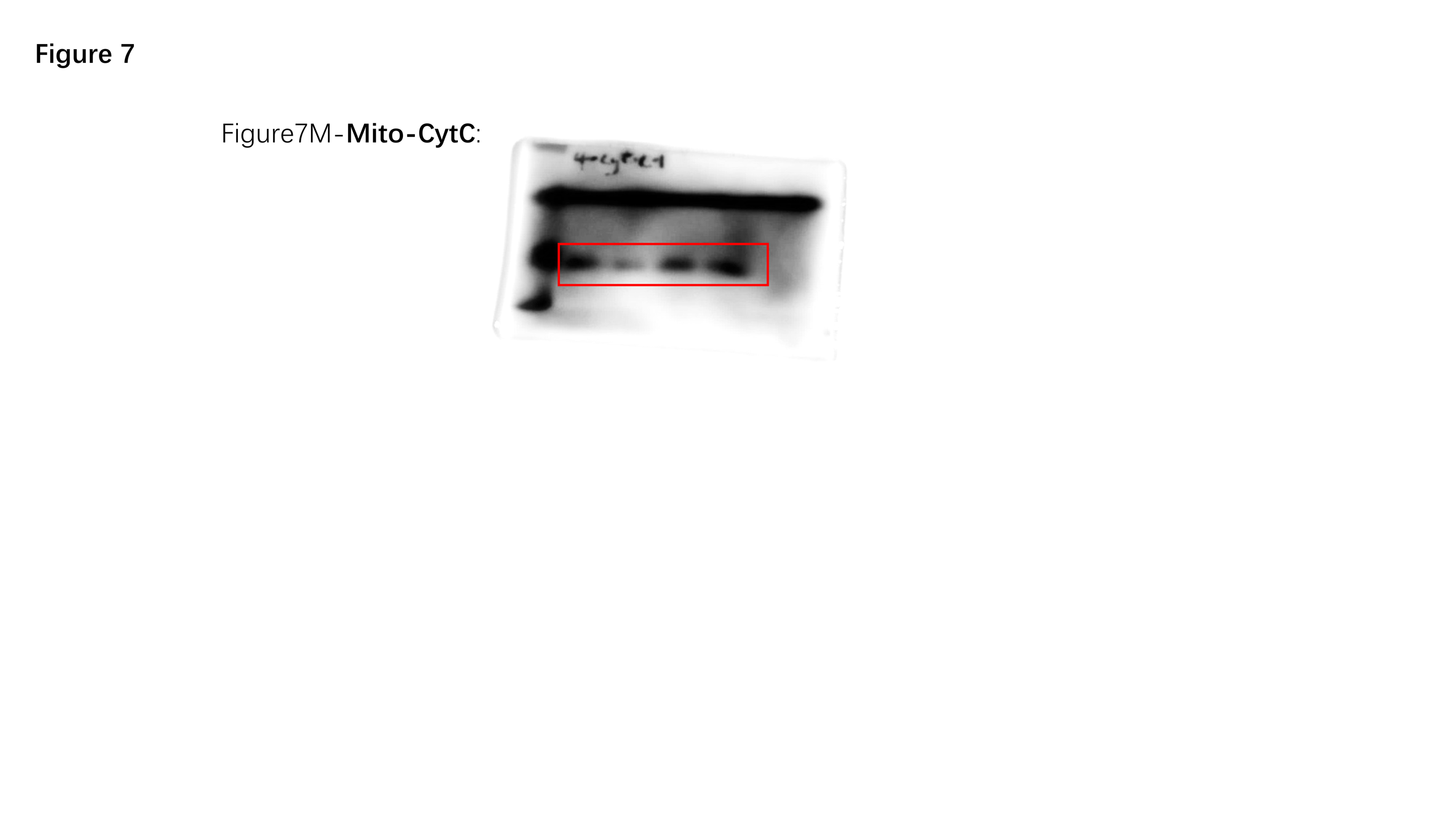

Supplement: Figure 7—source data 1. — (Figure 7B-CYP1A1) The expression of CYP1A1 expression in the placental tissue of Wild Type (The first three columns) and PM2.5-treated mice (the last three columns). (Figure 7B-β-actin) The expression of β-actin expression in the placental tissue of Wild Type (The first three columns) and PM2.5-treated mice (the last three columns). (Figure 7E-CYP1A1) The expression of CYP1A1 expression in the PM2.5-treated HTR8/SVneo cells (PM2.5 concentration: 0. 50 μg/mL, 100 μg/mL, 200 μg/mL). (Figure 7E-β-actin) The expression of β-actin expression in the PM2.5-treated HTR8/SVneo cells (PM2.5 concentration: 0. 50 μg/mL, 100 μg/mL, 200 μg/mL). (Figure 7F-CYP1A1) The expression of CYP1A1 expression in the HTR8/SVneo cells (si-NC, si-CYP1A1#1, si-CYP1A1#2). (Figure 7F-β-actin) The expression of β-actin expression in the HTR8/SVneo cells (si-NC, si-CYP1A1#1, si-CYP1A1#2). (Figure 7M-Mito-Cytc) The expression of Cytc expression in mitochondria of HTR8/SVneo (si-NC, PM2.5+si NC, PM2.5+si- CYP1A1#1, PM2.5+si- CYP1A1#2). (Figure 7M-Mito-VDAC-1) The expression of VDAC-1 expression in mitochondria of HTR8/SVneo (si-NC, PM2.5+si NC, PM2.5+si- CYP1A1#1, PM2.5+si- CYP1A1#2). (Figure 7M-Cyto-Cytc) The expression of Cytc expression in cytoplasm of HTR8/SVneo (si-NC, PM2.5+si NC, PM2.5+si- CYP1A1#1, PM2.5+si- CYP1A1#2). (Figure 7M-Cyto-BCL-2) The expression of BCL-2 expression in cytoplasm of HTR8/SVneo (si-NC, PM2.5+si NC, PM2.5+si- CYP1A1#1, PM2.5+si- CYP1A1#2). (Figure 7M-Cyto-BAX) The expression of BAX expression in cytoplasm of HTR8/SVneo (si-NC, PM2.5+si NC, PM2.5+si- CYP1A1#1, PM2.5+si- CYP1A1#2). (Figure 7M-Cyto-Cleaved-Caspase3) The expression of Cleaved-Caspase3 expression in cytoplasm of HTR8/SVneo (si-NC, PM2.5+si NC, PM2.5+si- CYP1A1#1, PM2.5+si- CYP1A1#2). (Figure 7M-Cyto-β-actin) The expression of β-actin expression in cytoplasm of HTR8/SVneo (si-NC, PM2.5+si NC, PM2.5+si- CYP1A1#1, PM2.5+si- CYP1A1#2). [file elife-85944-fig7-data1.zip › Figure 7-source data 1/Figure7M-Mito-CytC.TIF]

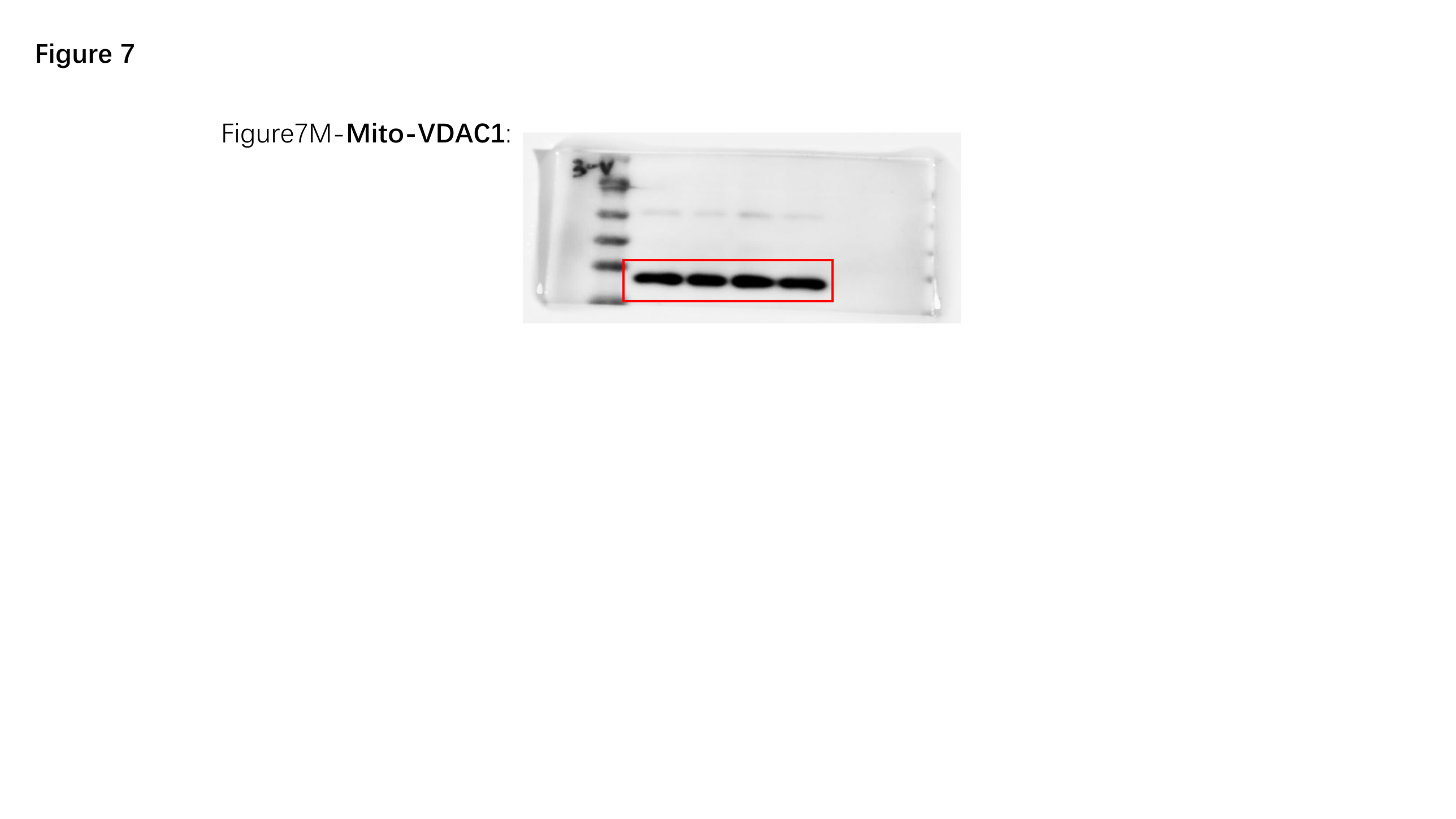

Supplement: Figure 7—source data 1. — (Figure 7B-CYP1A1) The expression of CYP1A1 expression in the placental tissue of Wild Type (The first three columns) and PM2.5-treated mice (the last three columns). (Figure 7B-β-actin) The expression of β-actin expression in the placental tissue of Wild Type (The first three columns) and PM2.5-treated mice (the last three columns). (Figure 7E-CYP1A1) The expression of CYP1A1 expression in the PM2.5-treated HTR8/SVneo cells (PM2.5 concentration: 0. 50 μg/mL, 100 μg/mL, 200 μg/mL). (Figure 7E-β-actin) The expression of β-actin expression in the PM2.5-treated HTR8/SVneo cells (PM2.5 concentration: 0. 50 μg/mL, 100 μg/mL, 200 μg/mL). (Figure 7F-CYP1A1) The expression of CYP1A1 expression in the HTR8/SVneo cells (si-NC, si-CYP1A1#1, si-CYP1A1#2). (Figure 7F-β-actin) The expression of β-actin expression in the HTR8/SVneo cells (si-NC, si-CYP1A1#1, si-CYP1A1#2). (Figure 7M-Mito-Cytc) The expression of Cytc expression in mitochondria of HTR8/SVneo (si-NC, PM2.5+si NC, PM2.5+si- CYP1A1#1, PM2.5+si- CYP1A1#2). (Figure 7M-Mito-VDAC-1) The expression of VDAC-1 expression in mitochondria of HTR8/SVneo (si-NC, PM2.5+si NC, PM2.5+si- CYP1A1#1, PM2.5+si- CYP1A1#2). (Figure 7M-Cyto-Cytc) The expression of Cytc expression in cytoplasm of HTR8/SVneo (si-NC, PM2.5+si NC, PM2.5+si- CYP1A1#1, PM2.5+si- CYP1A1#2). (Figure 7M-Cyto-BCL-2) The expression of BCL-2 expression in cytoplasm of HTR8/SVneo (si-NC, PM2.5+si NC, PM2.5+si- CYP1A1#1, PM2.5+si- CYP1A1#2). (Figure 7M-Cyto-BAX) The expression of BAX expression in cytoplasm of HTR8/SVneo (si-NC, PM2.5+si NC, PM2.5+si- CYP1A1#1, PM2.5+si- CYP1A1#2). (Figure 7M-Cyto-Cleaved-Caspase3) The expression of Cleaved-Caspase3 expression in cytoplasm of HTR8/SVneo (si-NC, PM2.5+si NC, PM2.5+si- CYP1A1#1, PM2.5+si- CYP1A1#2). (Figure 7M-Cyto-β-actin) The expression of β-actin expression in cytoplasm of HTR8/SVneo (si-NC, PM2.5+si NC, PM2.5+si- CYP1A1#1, PM2.5+si- CYP1A1#2). [file elife-85944-fig7-data1.zip › Figure 7-source data 1/Figure7M-Mito-VDAC1.TIF]

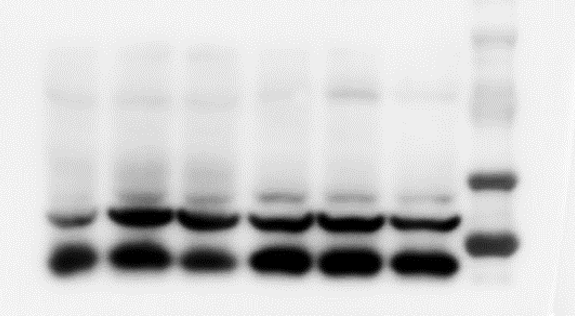

Supplement: Figure 7—source data 2. — (Figure 7B-CYP1A1) The expression of CYP1A1 expression in the placental tissue of Wild Type (The first three columns) and PM2.5-treated mice (the last three columns). (Figure 7B-β-actin) The expression of β-actin expression in the placental tissue of Wild Type (The first three columns) and PM2.5-treated mice (the last three columns). (Figure 7E-CYP1A1) The expression of CYP1A1 expression in the PM2.5-treated HTR8/SVneo cells (PM2.5 concentration: 0. 50 μg/mL, 100 μg/mL, 200 μg/mL). (Figure 7E-β-actin) The expression of β-actin expression in the PM2.5-treated HTR8/SVneo cells (PM2.5 concentration: 0. 50 μg/mL, 100 μg/mL, 200 μg/mL). (Figure 7F-CYP1A1) The expression of CYP1A1 expression in the HTR8/SVneo cells (si-NC, si-CYP1A1#1, si-CYP1A1#2). (Figure 7F-β-actin) The expression of β-actin expression in the HTR8/SVneo cells (si-NC, si-CYP1A1#1, si-CYP1A1#2). (Figure 7M-Mito-Cytc) The expression of Cytc expression in mitochondria of HTR8/SVneo (si-NC, PM2.5+si NC, PM2.5+si- CYP1A1#1, PM2.5+si- CYP1A1#2). (Figure 7M-Mito-VDAC-1) The expression of VDAC-1 expression in mitochondria of HTR8/SVneo (si-NC, PM2.5+si NC, PM2.5+si- CYP1A1#1, PM2.5+si- CYP1A1#2). (Figure 7M-Cyto-Cytc) The expression of Cytc expression in cytoplasm of HTR8/SVneo (si-NC, PM2.5+si NC, PM2.5+si- CYP1A1#1, PM2.5+si- CYP1A1#2). (Figure 7M-Cyto-BCL-2) The expression of BCL-2 expression in cytoplasm of HTR8/SVneo (si-NC, PM2.5+si NC, PM2.5+si- CYP1A1#1, PM2.5+si- CYP1A1#2). (Figure 7M-Cyto-BAX) The expression of BAX expression in cytoplasm of HTR8/SVneo (si-NC, PM2.5+si NC, PM2.5+si- CYP1A1#1, PM2.5+si- CYP1A1#2). (Figure 7M-Cyto-Cleaved-Caspase3) The expression of Cleaved-Caspase3 expression in cytoplasm of HTR8/SVneo (si-NC, PM2.5+si NC, PM2.5+si- CYP1A1#1, PM2.5+si- CYP1A1#2). (Figure 7M-Cyto-β-actin) The expression of β-actin expression in cytoplasm of HTR8/SVneo (si-NC, PM2.5+si NC, PM2.5+si- CYP1A1#1, PM2.5+si- CYP1A1#2). [file elife-85944-fig7-data2.zip › Figure 7-source data 2/Figure7B-CYP1A1.tif]

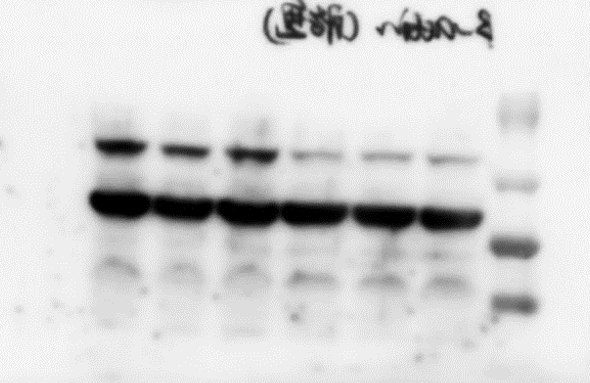

Supplement: Figure 7—source data 2. — (Figure 7B-CYP1A1) The expression of CYP1A1 expression in the placental tissue of Wild Type (The first three columns) and PM2.5-treated mice (the last three columns). (Figure 7B-β-actin) The expression of β-actin expression in the placental tissue of Wild Type (The first three columns) and PM2.5-treated mice (the last three columns). (Figure 7E-CYP1A1) The expression of CYP1A1 expression in the PM2.5-treated HTR8/SVneo cells (PM2.5 concentration: 0. 50 μg/mL, 100 μg/mL, 200 μg/mL). (Figure 7E-β-actin) The expression of β-actin expression in the PM2.5-treated HTR8/SVneo cells (PM2.5 concentration: 0. 50 μg/mL, 100 μg/mL, 200 μg/mL). (Figure 7F-CYP1A1) The expression of CYP1A1 expression in the HTR8/SVneo cells (si-NC, si-CYP1A1#1, si-CYP1A1#2). (Figure 7F-β-actin) The expression of β-actin expression in the HTR8/SVneo cells (si-NC, si-CYP1A1#1, si-CYP1A1#2). (Figure 7M-Mito-Cytc) The expression of Cytc expression in mitochondria of HTR8/SVneo (si-NC, PM2.5+si NC, PM2.5+si- CYP1A1#1, PM2.5+si- CYP1A1#2). (Figure 7M-Mito-VDAC-1) The expression of VDAC-1 expression in mitochondria of HTR8/SVneo (si-NC, PM2.5+si NC, PM2.5+si- CYP1A1#1, PM2.5+si- CYP1A1#2). (Figure 7M-Cyto-Cytc) The expression of Cytc expression in cytoplasm of HTR8/SVneo (si-NC, PM2.5+si NC, PM2.5+si- CYP1A1#1, PM2.5+si- CYP1A1#2). (Figure 7M-Cyto-BCL-2) The expression of BCL-2 expression in cytoplasm of HTR8/SVneo (si-NC, PM2.5+si NC, PM2.5+si- CYP1A1#1, PM2.5+si- CYP1A1#2). (Figure 7M-Cyto-BAX) The expression of BAX expression in cytoplasm of HTR8/SVneo (si-NC, PM2.5+si NC, PM2.5+si- CYP1A1#1, PM2.5+si- CYP1A1#2). (Figure 7M-Cyto-Cleaved-Caspase3) The expression of Cleaved-Caspase3 expression in cytoplasm of HTR8/SVneo (si-NC, PM2.5+si NC, PM2.5+si- CYP1A1#1, PM2.5+si- CYP1A1#2). (Figure 7M-Cyto-β-actin) The expression of β-actin expression in cytoplasm of HTR8/SVneo (si-NC, PM2.5+si NC, PM2.5+si- CYP1A1#1, PM2.5+si- CYP1A1#2). [file elife-85944-fig7-data2.zip › Figure 7-source data 2/Figure7B-a┬-actin.tif]

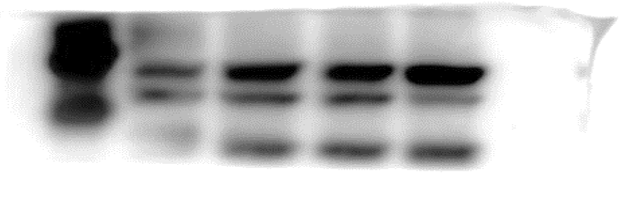

Supplement: Figure 7—source data 2. — (Figure 7B-CYP1A1) The expression of CYP1A1 expression in the placental tissue of Wild Type (The first three columns) and PM2.5-treated mice (the last three columns). (Figure 7B-β-actin) The expression of β-actin expression in the placental tissue of Wild Type (The first three columns) and PM2.5-treated mice (the last three columns). (Figure 7E-CYP1A1) The expression of CYP1A1 expression in the PM2.5-treated HTR8/SVneo cells (PM2.5 concentration: 0. 50 μg/mL, 100 μg/mL, 200 μg/mL). (Figure 7E-β-actin) The expression of β-actin expression in the PM2.5-treated HTR8/SVneo cells (PM2.5 concentration: 0. 50 μg/mL, 100 μg/mL, 200 μg/mL). (Figure 7F-CYP1A1) The expression of CYP1A1 expression in the HTR8/SVneo cells (si-NC, si-CYP1A1#1, si-CYP1A1#2). (Figure 7F-β-actin) The expression of β-actin expression in the HTR8/SVneo cells (si-NC, si-CYP1A1#1, si-CYP1A1#2). (Figure 7M-Mito-Cytc) The expression of Cytc expression in mitochondria of HTR8/SVneo (si-NC, PM2.5+si NC, PM2.5+si- CYP1A1#1, PM2.5+si- CYP1A1#2). (Figure 7M-Mito-VDAC-1) The expression of VDAC-1 expression in mitochondria of HTR8/SVneo (si-NC, PM2.5+si NC, PM2.5+si- CYP1A1#1, PM2.5+si- CYP1A1#2). (Figure 7M-Cyto-Cytc) The expression of Cytc expression in cytoplasm of HTR8/SVneo (si-NC, PM2.5+si NC, PM2.5+si- CYP1A1#1, PM2.5+si- CYP1A1#2). (Figure 7M-Cyto-BCL-2) The expression of BCL-2 expression in cytoplasm of HTR8/SVneo (si-NC, PM2.5+si NC, PM2.5+si- CYP1A1#1, PM2.5+si- CYP1A1#2). (Figure 7M-Cyto-BAX) The expression of BAX expression in cytoplasm of HTR8/SVneo (si-NC, PM2.5+si NC, PM2.5+si- CYP1A1#1, PM2.5+si- CYP1A1#2). (Figure 7M-Cyto-Cleaved-Caspase3) The expression of Cleaved-Caspase3 expression in cytoplasm of HTR8/SVneo (si-NC, PM2.5+si NC, PM2.5+si- CYP1A1#1, PM2.5+si- CYP1A1#2). (Figure 7M-Cyto-β-actin) The expression of β-actin expression in cytoplasm of HTR8/SVneo (si-NC, PM2.5+si NC, PM2.5+si- CYP1A1#1, PM2.5+si- CYP1A1#2). [file elife-85944-fig7-data2.zip › Figure 7-source data 2/Figure7E-CYP1A1.tif]

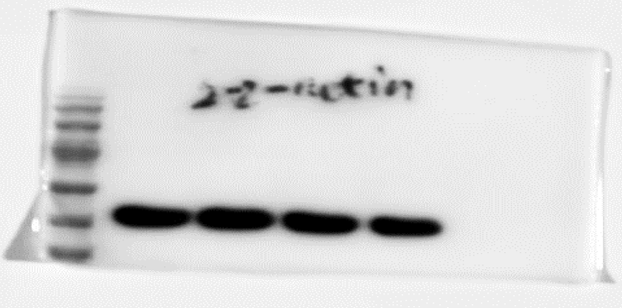

Supplement: Figure 7—source data 2. — (Figure 7B-CYP1A1) The expression of CYP1A1 expression in the placental tissue of Wild Type (The first three columns) and PM2.5-treated mice (the last three columns). (Figure 7B-β-actin) The expression of β-actin expression in the placental tissue of Wild Type (The first three columns) and PM2.5-treated mice (the last three columns). (Figure 7E-CYP1A1) The expression of CYP1A1 expression in the PM2.5-treated HTR8/SVneo cells (PM2.5 concentration: 0. 50 μg/mL, 100 μg/mL, 200 μg/mL). (Figure 7E-β-actin) The expression of β-actin expression in the PM2.5-treated HTR8/SVneo cells (PM2.5 concentration: 0. 50 μg/mL, 100 μg/mL, 200 μg/mL). (Figure 7F-CYP1A1) The expression of CYP1A1 expression in the HTR8/SVneo cells (si-NC, si-CYP1A1#1, si-CYP1A1#2). (Figure 7F-β-actin) The expression of β-actin expression in the HTR8/SVneo cells (si-NC, si-CYP1A1#1, si-CYP1A1#2). (Figure 7M-Mito-Cytc) The expression of Cytc expression in mitochondria of HTR8/SVneo (si-NC, PM2.5+si NC, PM2.5+si- CYP1A1#1, PM2.5+si- CYP1A1#2). (Figure 7M-Mito-VDAC-1) The expression of VDAC-1 expression in mitochondria of HTR8/SVneo (si-NC, PM2.5+si NC, PM2.5+si- CYP1A1#1, PM2.5+si- CYP1A1#2). (Figure 7M-Cyto-Cytc) The expression of Cytc expression in cytoplasm of HTR8/SVneo (si-NC, PM2.5+si NC, PM2.5+si- CYP1A1#1, PM2.5+si- CYP1A1#2). (Figure 7M-Cyto-BCL-2) The expression of BCL-2 expression in cytoplasm of HTR8/SVneo (si-NC, PM2.5+si NC, PM2.5+si- CYP1A1#1, PM2.5+si- CYP1A1#2). (Figure 7M-Cyto-BAX) The expression of BAX expression in cytoplasm of HTR8/SVneo (si-NC, PM2.5+si NC, PM2.5+si- CYP1A1#1, PM2.5+si- CYP1A1#2). (Figure 7M-Cyto-Cleaved-Caspase3) The expression of Cleaved-Caspase3 expression in cytoplasm of HTR8/SVneo (si-NC, PM2.5+si NC, PM2.5+si- CYP1A1#1, PM2.5+si- CYP1A1#2). (Figure 7M-Cyto-β-actin) The expression of β-actin expression in cytoplasm of HTR8/SVneo (si-NC, PM2.5+si NC, PM2.5+si- CYP1A1#1, PM2.5+si- CYP1A1#2). [file elife-85944-fig7-data2.zip › Figure 7-source data 2/Figure7E-a┬-actin.tif]

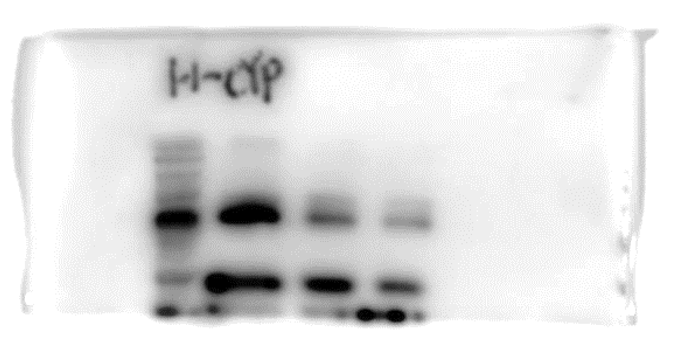

Supplement: Figure 7—source data 2. — (Figure 7B-CYP1A1) The expression of CYP1A1 expression in the placental tissue of Wild Type (The first three columns) and PM2.5-treated mice (the last three columns). (Figure 7B-β-actin) The expression of β-actin expression in the placental tissue of Wild Type (The first three columns) and PM2.5-treated mice (the last three columns). (Figure 7E-CYP1A1) The expression of CYP1A1 expression in the PM2.5-treated HTR8/SVneo cells (PM2.5 concentration: 0. 50 μg/mL, 100 μg/mL, 200 μg/mL). (Figure 7E-β-actin) The expression of β-actin expression in the PM2.5-treated HTR8/SVneo cells (PM2.5 concentration: 0. 50 μg/mL, 100 μg/mL, 200 μg/mL). (Figure 7F-CYP1A1) The expression of CYP1A1 expression in the HTR8/SVneo cells (si-NC, si-CYP1A1#1, si-CYP1A1#2). (Figure 7F-β-actin) The expression of β-actin expression in the HTR8/SVneo cells (si-NC, si-CYP1A1#1, si-CYP1A1#2). (Figure 7M-Mito-Cytc) The expression of Cytc expression in mitochondria of HTR8/SVneo (si-NC, PM2.5+si NC, PM2.5+si- CYP1A1#1, PM2.5+si- CYP1A1#2). (Figure 7M-Mito-VDAC-1) The expression of VDAC-1 expression in mitochondria of HTR8/SVneo (si-NC, PM2.5+si NC, PM2.5+si- CYP1A1#1, PM2.5+si- CYP1A1#2). (Figure 7M-Cyto-Cytc) The expression of Cytc expression in cytoplasm of HTR8/SVneo (si-NC, PM2.5+si NC, PM2.5+si- CYP1A1#1, PM2.5+si- CYP1A1#2). (Figure 7M-Cyto-BCL-2) The expression of BCL-2 expression in cytoplasm of HTR8/SVneo (si-NC, PM2.5+si NC, PM2.5+si- CYP1A1#1, PM2.5+si- CYP1A1#2). (Figure 7M-Cyto-BAX) The expression of BAX expression in cytoplasm of HTR8/SVneo (si-NC, PM2.5+si NC, PM2.5+si- CYP1A1#1, PM2.5+si- CYP1A1#2). (Figure 7M-Cyto-Cleaved-Caspase3) The expression of Cleaved-Caspase3 expression in cytoplasm of HTR8/SVneo (si-NC, PM2.5+si NC, PM2.5+si- CYP1A1#1, PM2.5+si- CYP1A1#2). (Figure 7M-Cyto-β-actin) The expression of β-actin expression in cytoplasm of HTR8/SVneo (si-NC, PM2.5+si NC, PM2.5+si- CYP1A1#1, PM2.5+si- CYP1A1#2). [file elife-85944-fig7-data2.zip › Figure 7-source data 2/Figure7F-CYP1A1.tif]

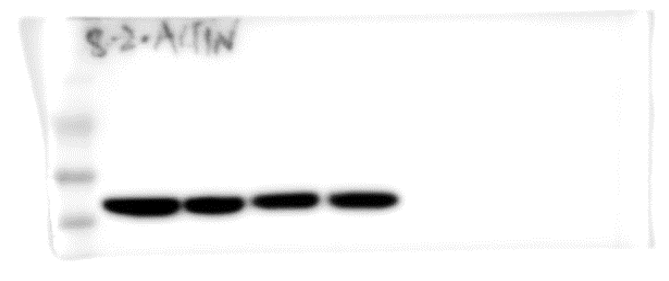

Supplement: Figure 7—source data 2. — (Figure 7B-CYP1A1) The expression of CYP1A1 expression in the placental tissue of Wild Type (The first three columns) and PM2.5-treated mice (the last three columns). (Figure 7B-β-actin) The expression of β-actin expression in the placental tissue of Wild Type (The first three columns) and PM2.5-treated mice (the last three columns). (Figure 7E-CYP1A1) The expression of CYP1A1 expression in the PM2.5-treated HTR8/SVneo cells (PM2.5 concentration: 0. 50 μg/mL, 100 μg/mL, 200 μg/mL). (Figure 7E-β-actin) The expression of β-actin expression in the PM2.5-treated HTR8/SVneo cells (PM2.5 concentration: 0. 50 μg/mL, 100 μg/mL, 200 μg/mL). (Figure 7F-CYP1A1) The expression of CYP1A1 expression in the HTR8/SVneo cells (si-NC, si-CYP1A1#1, si-CYP1A1#2). (Figure 7F-β-actin) The expression of β-actin expression in the HTR8/SVneo cells (si-NC, si-CYP1A1#1, si-CYP1A1#2). (Figure 7M-Mito-Cytc) The expression of Cytc expression in mitochondria of HTR8/SVneo (si-NC, PM2.5+si NC, PM2.5+si- CYP1A1#1, PM2.5+si- CYP1A1#2). (Figure 7M-Mito-VDAC-1) The expression of VDAC-1 expression in mitochondria of HTR8/SVneo (si-NC, PM2.5+si NC, PM2.5+si- CYP1A1#1, PM2.5+si- CYP1A1#2). (Figure 7M-Cyto-Cytc) The expression of Cytc expression in cytoplasm of HTR8/SVneo (si-NC, PM2.5+si NC, PM2.5+si- CYP1A1#1, PM2.5+si- CYP1A1#2). (Figure 7M-Cyto-BCL-2) The expression of BCL-2 expression in cytoplasm of HTR8/SVneo (si-NC, PM2.5+si NC, PM2.5+si- CYP1A1#1, PM2.5+si- CYP1A1#2). (Figure 7M-Cyto-BAX) The expression of BAX expression in cytoplasm of HTR8/SVneo (si-NC, PM2.5+si NC, PM2.5+si- CYP1A1#1, PM2.5+si- CYP1A1#2). (Figure 7M-Cyto-Cleaved-Caspase3) The expression of Cleaved-Caspase3 expression in cytoplasm of HTR8/SVneo (si-NC, PM2.5+si NC, PM2.5+si- CYP1A1#1, PM2.5+si- CYP1A1#2). (Figure 7M-Cyto-β-actin) The expression of β-actin expression in cytoplasm of HTR8/SVneo (si-NC, PM2.5+si NC, PM2.5+si- CYP1A1#1, PM2.5+si- CYP1A1#2). [file elife-85944-fig7-data2.zip › Figure 7-source data 2/Figure7F-a┬-actin.tif]

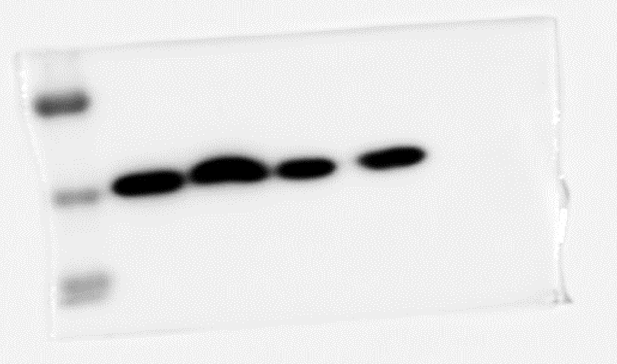

Supplement: Figure 7—source data 2. — (Figure 7B-CYP1A1) The expression of CYP1A1 expression in the placental tissue of Wild Type (The first three columns) and PM2.5-treated mice (the last three columns). (Figure 7B-β-actin) The expression of β-actin expression in the placental tissue of Wild Type (The first three columns) and PM2.5-treated mice (the last three columns). (Figure 7E-CYP1A1) The expression of CYP1A1 expression in the PM2.5-treated HTR8/SVneo cells (PM2.5 concentration: 0. 50 μg/mL, 100 μg/mL, 200 μg/mL). (Figure 7E-β-actin) The expression of β-actin expression in the PM2.5-treated HTR8/SVneo cells (PM2.5 concentration: 0. 50 μg/mL, 100 μg/mL, 200 μg/mL). (Figure 7F-CYP1A1) The expression of CYP1A1 expression in the HTR8/SVneo cells (si-NC, si-CYP1A1#1, si-CYP1A1#2). (Figure 7F-β-actin) The expression of β-actin expression in the HTR8/SVneo cells (si-NC, si-CYP1A1#1, si-CYP1A1#2). (Figure 7M-Mito-Cytc) The expression of Cytc expression in mitochondria of HTR8/SVneo (si-NC, PM2.5+si NC, PM2.5+si- CYP1A1#1, PM2.5+si- CYP1A1#2). (Figure 7M-Mito-VDAC-1) The expression of VDAC-1 expression in mitochondria of HTR8/SVneo (si-NC, PM2.5+si NC, PM2.5+si- CYP1A1#1, PM2.5+si- CYP1A1#2). (Figure 7M-Cyto-Cytc) The expression of Cytc expression in cytoplasm of HTR8/SVneo (si-NC, PM2.5+si NC, PM2.5+si- CYP1A1#1, PM2.5+si- CYP1A1#2). (Figure 7M-Cyto-BCL-2) The expression of BCL-2 expression in cytoplasm of HTR8/SVneo (si-NC, PM2.5+si NC, PM2.5+si- CYP1A1#1, PM2.5+si- CYP1A1#2). (Figure 7M-Cyto-BAX) The expression of BAX expression in cytoplasm of HTR8/SVneo (si-NC, PM2.5+si NC, PM2.5+si- CYP1A1#1, PM2.5+si- CYP1A1#2). (Figure 7M-Cyto-Cleaved-Caspase3) The expression of Cleaved-Caspase3 expression in cytoplasm of HTR8/SVneo (si-NC, PM2.5+si NC, PM2.5+si- CYP1A1#1, PM2.5+si- CYP1A1#2). (Figure 7M-Cyto-β-actin) The expression of β-actin expression in cytoplasm of HTR8/SVneo (si-NC, PM2.5+si NC, PM2.5+si- CYP1A1#1, PM2.5+si- CYP1A1#2). [file elife-85944-fig7-data2.zip › Figure 7-source data 2/Figure7M-Cyto-BAX.tif]

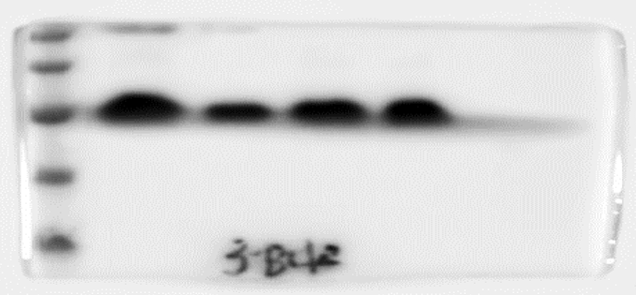

Supplement: Figure 7—source data 2. — (Figure 7B-CYP1A1) The expression of CYP1A1 expression in the placental tissue of Wild Type (The first three columns) and PM2.5-treated mice (the last three columns). (Figure 7B-β-actin) The expression of β-actin expression in the placental tissue of Wild Type (The first three columns) and PM2.5-treated mice (the last three columns). (Figure 7E-CYP1A1) The expression of CYP1A1 expression in the PM2.5-treated HTR8/SVneo cells (PM2.5 concentration: 0. 50 μg/mL, 100 μg/mL, 200 μg/mL). (Figure 7E-β-actin) The expression of β-actin expression in the PM2.5-treated HTR8/SVneo cells (PM2.5 concentration: 0. 50 μg/mL, 100 μg/mL, 200 μg/mL). (Figure 7F-CYP1A1) The expression of CYP1A1 expression in the HTR8/SVneo cells (si-NC, si-CYP1A1#1, si-CYP1A1#2). (Figure 7F-β-actin) The expression of β-actin expression in the HTR8/SVneo cells (si-NC, si-CYP1A1#1, si-CYP1A1#2). (Figure 7M-Mito-Cytc) The expression of Cytc expression in mitochondria of HTR8/SVneo (si-NC, PM2.5+si NC, PM2.5+si- CYP1A1#1, PM2.5+si- CYP1A1#2). (Figure 7M-Mito-VDAC-1) The expression of VDAC-1 expression in mitochondria of HTR8/SVneo (si-NC, PM2.5+si NC, PM2.5+si- CYP1A1#1, PM2.5+si- CYP1A1#2). (Figure 7M-Cyto-Cytc) The expression of Cytc expression in cytoplasm of HTR8/SVneo (si-NC, PM2.5+si NC, PM2.5+si- CYP1A1#1, PM2.5+si- CYP1A1#2). (Figure 7M-Cyto-BCL-2) The expression of BCL-2 expression in cytoplasm of HTR8/SVneo (si-NC, PM2.5+si NC, PM2.5+si- CYP1A1#1, PM2.5+si- CYP1A1#2). (Figure 7M-Cyto-BAX) The expression of BAX expression in cytoplasm of HTR8/SVneo (si-NC, PM2.5+si NC, PM2.5+si- CYP1A1#1, PM2.5+si- CYP1A1#2). (Figure 7M-Cyto-Cleaved-Caspase3) The expression of Cleaved-Caspase3 expression in cytoplasm of HTR8/SVneo (si-NC, PM2.5+si NC, PM2.5+si- CYP1A1#1, PM2.5+si- CYP1A1#2). (Figure 7M-Cyto-β-actin) The expression of β-actin expression in cytoplasm of HTR8/SVneo (si-NC, PM2.5+si NC, PM2.5+si- CYP1A1#1, PM2.5+si- CYP1A1#2). [file elife-85944-fig7-data2.zip › Figure 7-source data 2/Figure7M-Cyto-BCL-2.tif]

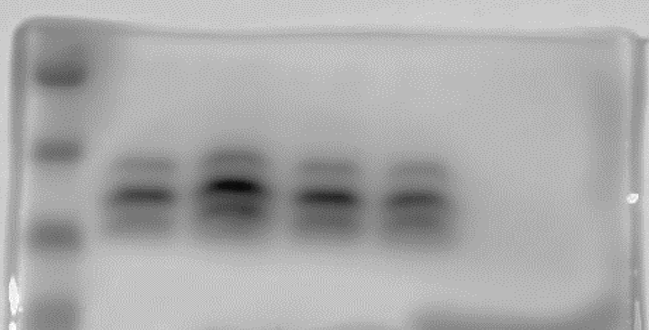

Supplement: Figure 7—source data 2. — (Figure 7B-CYP1A1) The expression of CYP1A1 expression in the placental tissue of Wild Type (The first three columns) and PM2.5-treated mice (the last three columns). (Figure 7B-β-actin) The expression of β-actin expression in the placental tissue of Wild Type (The first three columns) and PM2.5-treated mice (the last three columns). (Figure 7E-CYP1A1) The expression of CYP1A1 expression in the PM2.5-treated HTR8/SVneo cells (PM2.5 concentration: 0. 50 μg/mL, 100 μg/mL, 200 μg/mL). (Figure 7E-β-actin) The expression of β-actin expression in the PM2.5-treated HTR8/SVneo cells (PM2.5 concentration: 0. 50 μg/mL, 100 μg/mL, 200 μg/mL). (Figure 7F-CYP1A1) The expression of CYP1A1 expression in the HTR8/SVneo cells (si-NC, si-CYP1A1#1, si-CYP1A1#2). (Figure 7F-β-actin) The expression of β-actin expression in the HTR8/SVneo cells (si-NC, si-CYP1A1#1, si-CYP1A1#2). (Figure 7M-Mito-Cytc) The expression of Cytc expression in mitochondria of HTR8/SVneo (si-NC, PM2.5+si NC, PM2.5+si- CYP1A1#1, PM2.5+si- CYP1A1#2). (Figure 7M-Mito-VDAC-1) The expression of VDAC-1 expression in mitochondria of HTR8/SVneo (si-NC, PM2.5+si NC, PM2.5+si- CYP1A1#1, PM2.5+si- CYP1A1#2). (Figure 7M-Cyto-Cytc) The expression of Cytc expression in cytoplasm of HTR8/SVneo (si-NC, PM2.5+si NC, PM2.5+si- CYP1A1#1, PM2.5+si- CYP1A1#2). (Figure 7M-Cyto-BCL-2) The expression of BCL-2 expression in cytoplasm of HTR8/SVneo (si-NC, PM2.5+si NC, PM2.5+si- CYP1A1#1, PM2.5+si- CYP1A1#2). (Figure 7M-Cyto-BAX) The expression of BAX expression in cytoplasm of HTR8/SVneo (si-NC, PM2.5+si NC, PM2.5+si- CYP1A1#1, PM2.5+si- CYP1A1#2). (Figure 7M-Cyto-Cleaved-Caspase3) The expression of Cleaved-Caspase3 expression in cytoplasm of HTR8/SVneo (si-NC, PM2.5+si NC, PM2.5+si- CYP1A1#1, PM2.5+si- CYP1A1#2). (Figure 7M-Cyto-β-actin) The expression of β-actin expression in cytoplasm of HTR8/SVneo (si-NC, PM2.5+si NC, PM2.5+si- CYP1A1#1, PM2.5+si- CYP1A1#2). [file elife-85944-fig7-data2.zip › Figure 7-source data 2/Figure7M-Cyto-CC3.tif]

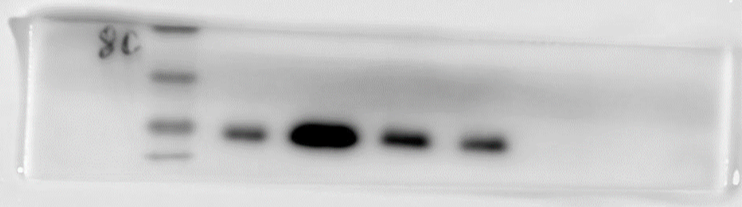

Supplement: Figure 7—source data 2. — (Figure 7B-CYP1A1) The expression of CYP1A1 expression in the placental tissue of Wild Type (The first three columns) and PM2.5-treated mice (the last three columns). (Figure 7B-β-actin) The expression of β-actin expression in the placental tissue of Wild Type (The first three columns) and PM2.5-treated mice (the last three columns). (Figure 7E-CYP1A1) The expression of CYP1A1 expression in the PM2.5-treated HTR8/SVneo cells (PM2.5 concentration: 0. 50 μg/mL, 100 μg/mL, 200 μg/mL). (Figure 7E-β-actin) The expression of β-actin expression in the PM2.5-treated HTR8/SVneo cells (PM2.5 concentration: 0. 50 μg/mL, 100 μg/mL, 200 μg/mL). (Figure 7F-CYP1A1) The expression of CYP1A1 expression in the HTR8/SVneo cells (si-NC, si-CYP1A1#1, si-CYP1A1#2). (Figure 7F-β-actin) The expression of β-actin expression in the HTR8/SVneo cells (si-NC, si-CYP1A1#1, si-CYP1A1#2). (Figure 7M-Mito-Cytc) The expression of Cytc expression in mitochondria of HTR8/SVneo (si-NC, PM2.5+si NC, PM2.5+si- CYP1A1#1, PM2.5+si- CYP1A1#2). (Figure 7M-Mito-VDAC-1) The expression of VDAC-1 expression in mitochondria of HTR8/SVneo (si-NC, PM2.5+si NC, PM2.5+si- CYP1A1#1, PM2.5+si- CYP1A1#2). (Figure 7M-Cyto-Cytc) The expression of Cytc expression in cytoplasm of HTR8/SVneo (si-NC, PM2.5+si NC, PM2.5+si- CYP1A1#1, PM2.5+si- CYP1A1#2). (Figure 7M-Cyto-BCL-2) The expression of BCL-2 expression in cytoplasm of HTR8/SVneo (si-NC, PM2.5+si NC, PM2.5+si- CYP1A1#1, PM2.5+si- CYP1A1#2). (Figure 7M-Cyto-BAX) The expression of BAX expression in cytoplasm of HTR8/SVneo (si-NC, PM2.5+si NC, PM2.5+si- CYP1A1#1, PM2.5+si- CYP1A1#2). (Figure 7M-Cyto-Cleaved-Caspase3) The expression of Cleaved-Caspase3 expression in cytoplasm of HTR8/SVneo (si-NC, PM2.5+si NC, PM2.5+si- CYP1A1#1, PM2.5+si- CYP1A1#2). (Figure 7M-Cyto-β-actin) The expression of β-actin expression in cytoplasm of HTR8/SVneo (si-NC, PM2.5+si NC, PM2.5+si- CYP1A1#1, PM2.5+si- CYP1A1#2). [file elife-85944-fig7-data2.zip › Figure 7-source data 2/Figure7M-Cyto-CytC.tif]

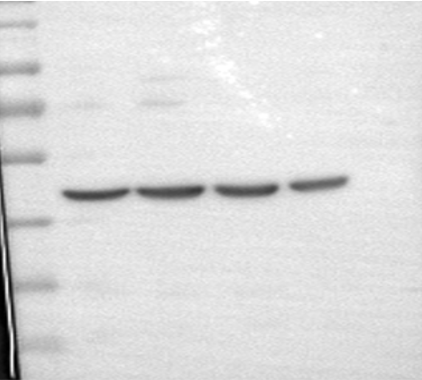

Supplement: Figure 7—source data 2. — (Figure 7B-CYP1A1) The expression of CYP1A1 expression in the placental tissue of Wild Type (The first three columns) and PM2.5-treated mice (the last three columns). (Figure 7B-β-actin) The expression of β-actin expression in the placental tissue of Wild Type (The first three columns) and PM2.5-treated mice (the last three columns). (Figure 7E-CYP1A1) The expression of CYP1A1 expression in the PM2.5-treated HTR8/SVneo cells (PM2.5 concentration: 0. 50 μg/mL, 100 μg/mL, 200 μg/mL). (Figure 7E-β-actin) The expression of β-actin expression in the PM2.5-treated HTR8/SVneo cells (PM2.5 concentration: 0. 50 μg/mL, 100 μg/mL, 200 μg/mL). (Figure 7F-CYP1A1) The expression of CYP1A1 expression in the HTR8/SVneo cells (si-NC, si-CYP1A1#1, si-CYP1A1#2). (Figure 7F-β-actin) The expression of β-actin expression in the HTR8/SVneo cells (si-NC, si-CYP1A1#1, si-CYP1A1#2). (Figure 7M-Mito-Cytc) The expression of Cytc expression in mitochondria of HTR8/SVneo (si-NC, PM2.5+si NC, PM2.5+si- CYP1A1#1, PM2.5+si- CYP1A1#2). (Figure 7M-Mito-VDAC-1) The expression of VDAC-1 expression in mitochondria of HTR8/SVneo (si-NC, PM2.5+si NC, PM2.5+si- CYP1A1#1, PM2.5+si- CYP1A1#2). (Figure 7M-Cyto-Cytc) The expression of Cytc expression in cytoplasm of HTR8/SVneo (si-NC, PM2.5+si NC, PM2.5+si- CYP1A1#1, PM2.5+si- CYP1A1#2). (Figure 7M-Cyto-BCL-2) The expression of BCL-2 expression in cytoplasm of HTR8/SVneo (si-NC, PM2.5+si NC, PM2.5+si- CYP1A1#1, PM2.5+si- CYP1A1#2). (Figure 7M-Cyto-BAX) The expression of BAX expression in cytoplasm of HTR8/SVneo (si-NC, PM2.5+si NC, PM2.5+si- CYP1A1#1, PM2.5+si- CYP1A1#2). (Figure 7M-Cyto-Cleaved-Caspase3) The expression of Cleaved-Caspase3 expression in cytoplasm of HTR8/SVneo (si-NC, PM2.5+si NC, PM2.5+si- CYP1A1#1, PM2.5+si- CYP1A1#2). (Figure 7M-Cyto-β-actin) The expression of β-actin expression in cytoplasm of HTR8/SVneo (si-NC, PM2.5+si NC, PM2.5+si- CYP1A1#1, PM2.5+si- CYP1A1#2). [file elife-85944-fig7-data2.zip › Figure 7-source data 2/Figure7M-Cyto-a┬-actin.tif]

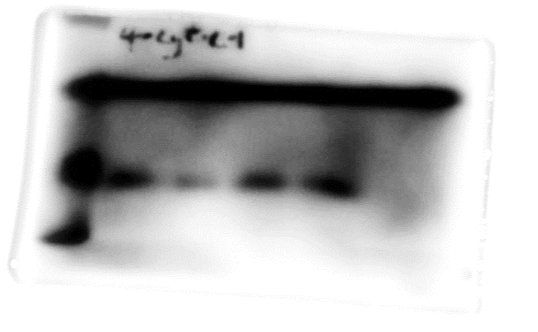

Supplement: Figure 7—source data 2. — (Figure 7B-CYP1A1) The expression of CYP1A1 expression in the placental tissue of Wild Type (The first three columns) and PM2.5-treated mice (the last three columns). (Figure 7B-β-actin) The expression of β-actin expression in the placental tissue of Wild Type (The first three columns) and PM2.5-treated mice (the last three columns). (Figure 7E-CYP1A1) The expression of CYP1A1 expression in the PM2.5-treated HTR8/SVneo cells (PM2.5 concentration: 0. 50 μg/mL, 100 μg/mL, 200 μg/mL). (Figure 7E-β-actin) The expression of β-actin expression in the PM2.5-treated HTR8/SVneo cells (PM2.5 concentration: 0. 50 μg/mL, 100 μg/mL, 200 μg/mL). (Figure 7F-CYP1A1) The expression of CYP1A1 expression in the HTR8/SVneo cells (si-NC, si-CYP1A1#1, si-CYP1A1#2). (Figure 7F-β-actin) The expression of β-actin expression in the HTR8/SVneo cells (si-NC, si-CYP1A1#1, si-CYP1A1#2). (Figure 7M-Mito-Cytc) The expression of Cytc expression in mitochondria of HTR8/SVneo (si-NC, PM2.5+si NC, PM2.5+si- CYP1A1#1, PM2.5+si- CYP1A1#2). (Figure 7M-Mito-VDAC-1) The expression of VDAC-1 expression in mitochondria of HTR8/SVneo (si-NC, PM2.5+si NC, PM2.5+si- CYP1A1#1, PM2.5+si- CYP1A1#2). (Figure 7M-Cyto-Cytc) The expression of Cytc expression in cytoplasm of HTR8/SVneo (si-NC, PM2.5+si NC, PM2.5+si- CYP1A1#1, PM2.5+si- CYP1A1#2). (Figure 7M-Cyto-BCL-2) The expression of BCL-2 expression in cytoplasm of HTR8/SVneo (si-NC, PM2.5+si NC, PM2.5+si- CYP1A1#1, PM2.5+si- CYP1A1#2). (Figure 7M-Cyto-BAX) The expression of BAX expression in cytoplasm of HTR8/SVneo (si-NC, PM2.5+si NC, PM2.5+si- CYP1A1#1, PM2.5+si- CYP1A1#2). (Figure 7M-Cyto-Cleaved-Caspase3) The expression of Cleaved-Caspase3 expression in cytoplasm of HTR8/SVneo (si-NC, PM2.5+si NC, PM2.5+si- CYP1A1#1, PM2.5+si- CYP1A1#2). (Figure 7M-Cyto-β-actin) The expression of β-actin expression in cytoplasm of HTR8/SVneo (si-NC, PM2.5+si NC, PM2.5+si- CYP1A1#1, PM2.5+si- CYP1A1#2). [file elife-85944-fig7-data2.zip › Figure 7-source data 2/Figure7M-Mito-CytC.tif]

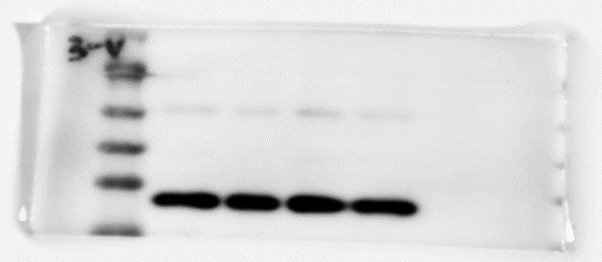

Supplement: Figure 7—source data 2. — (Figure 7B-CYP1A1) The expression of CYP1A1 expression in the placental tissue of Wild Type (The first three columns) and PM2.5-treated mice (the last three columns). (Figure 7B-β-actin) The expression of β-actin expression in the placental tissue of Wild Type (The first three columns) and PM2.5-treated mice (the last three columns). (Figure 7E-CYP1A1) The expression of CYP1A1 expression in the PM2.5-treated HTR8/SVneo cells (PM2.5 concentration: 0. 50 μg/mL, 100 μg/mL, 200 μg/mL). (Figure 7E-β-actin) The expression of β-actin expression in the PM2.5-treated HTR8/SVneo cells (PM2.5 concentration: 0. 50 μg/mL, 100 μg/mL, 200 μg/mL). (Figure 7F-CYP1A1) The expression of CYP1A1 expression in the HTR8/SVneo cells (si-NC, si-CYP1A1#1, si-CYP1A1#2). (Figure 7F-β-actin) The expression of β-actin expression in the HTR8/SVneo cells (si-NC, si-CYP1A1#1, si-CYP1A1#2). (Figure 7M-Mito-Cytc) The expression of Cytc expression in mitochondria of HTR8/SVneo (si-NC, PM2.5+si NC, PM2.5+si- CYP1A1#1, PM2.5+si- CYP1A1#2). (Figure 7M-Mito-VDAC-1) The expression of VDAC-1 expression in mitochondria of HTR8/SVneo (si-NC, PM2.5+si NC, PM2.5+si- CYP1A1#1, PM2.5+si- CYP1A1#2). (Figure 7M-Cyto-Cytc) The expression of Cytc expression in cytoplasm of HTR8/SVneo (si-NC, PM2.5+si NC, PM2.5+si- CYP1A1#1, PM2.5+si- CYP1A1#2). (Figure 7M-Cyto-BCL-2) The expression of BCL-2 expression in cytoplasm of HTR8/SVneo (si-NC, PM2.5+si NC, PM2.5+si- CYP1A1#1, PM2.5+si- CYP1A1#2). (Figure 7M-Cyto-BAX) The expression of BAX expression in cytoplasm of HTR8/SVneo (si-NC, PM2.5+si NC, PM2.5+si- CYP1A1#1, PM2.5+si- CYP1A1#2). (Figure 7M-Cyto-Cleaved-Caspase3) The expression of Cleaved-Caspase3 expression in cytoplasm of HTR8/SVneo (si-NC, PM2.5+si NC, PM2.5+si- CYP1A1#1, PM2.5+si- CYP1A1#2). (Figure 7M-Cyto-β-actin) The expression of β-actin expression in cytoplasm of HTR8/SVneo (si-NC, PM2.5+si NC, PM2.5+si- CYP1A1#1, PM2.5+si- CYP1A1#2). [file elife-85944-fig7-data2.zip › Figure 7-source data 2/Figure7M-Mito-VDAC1.tif]

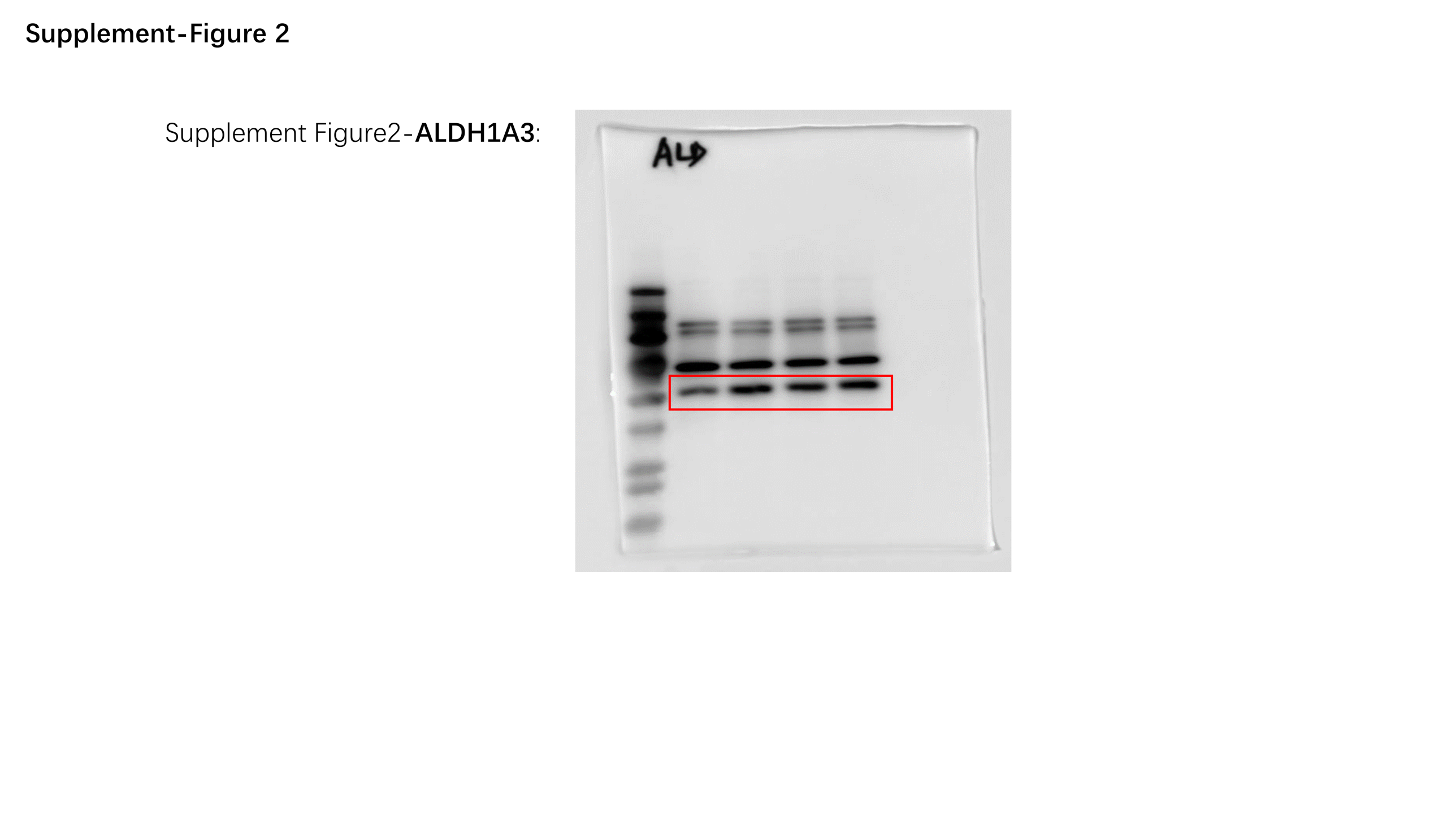

Supplement: Figure 7—figure supplement 1—source data 1. — (Figure 7—figure supplement 1B–CYP1A1) The expression of CYP1A1 expression in the PM2.5-treated HTR8/SVneo cells (PM2.5 concentration: 0. 50 μg/mL, 100 μg/mL, 200 μg/mL). (Figure 7—figure supplement 1B–CYP1B1) The expression of CYP1B1 expression in the PM2.5-treated HTR8/SVneo cells (PM2.5 concentration: 0. 50 μg/mL, 100 μg/mL, 200 μg/mL). (Figure 7—figure supplement 1B–ALDH1A3) The expression of ALDH1A3 expression in the PM2.5-treated HTR8/SVneo cells (PM2.5 concentration: 0. 50 μg/mL, 100 μg/mL, 200 μg/mL). (Figure 7—figure supplement 1B-β-actin) The expression of β-actin expression in the PM2.5-treated HTR8/SVneo cells (PM2.5 concentration: 0. 50 μg/mL, 100 μg/mL, 200 μg/mL). [file elife-85944-fig7-figsupp1-data1.zip › Figure 7-figure supplement 1-source data 1/Figure 7-figure supplement 1B-ALDH1A3.TIF]

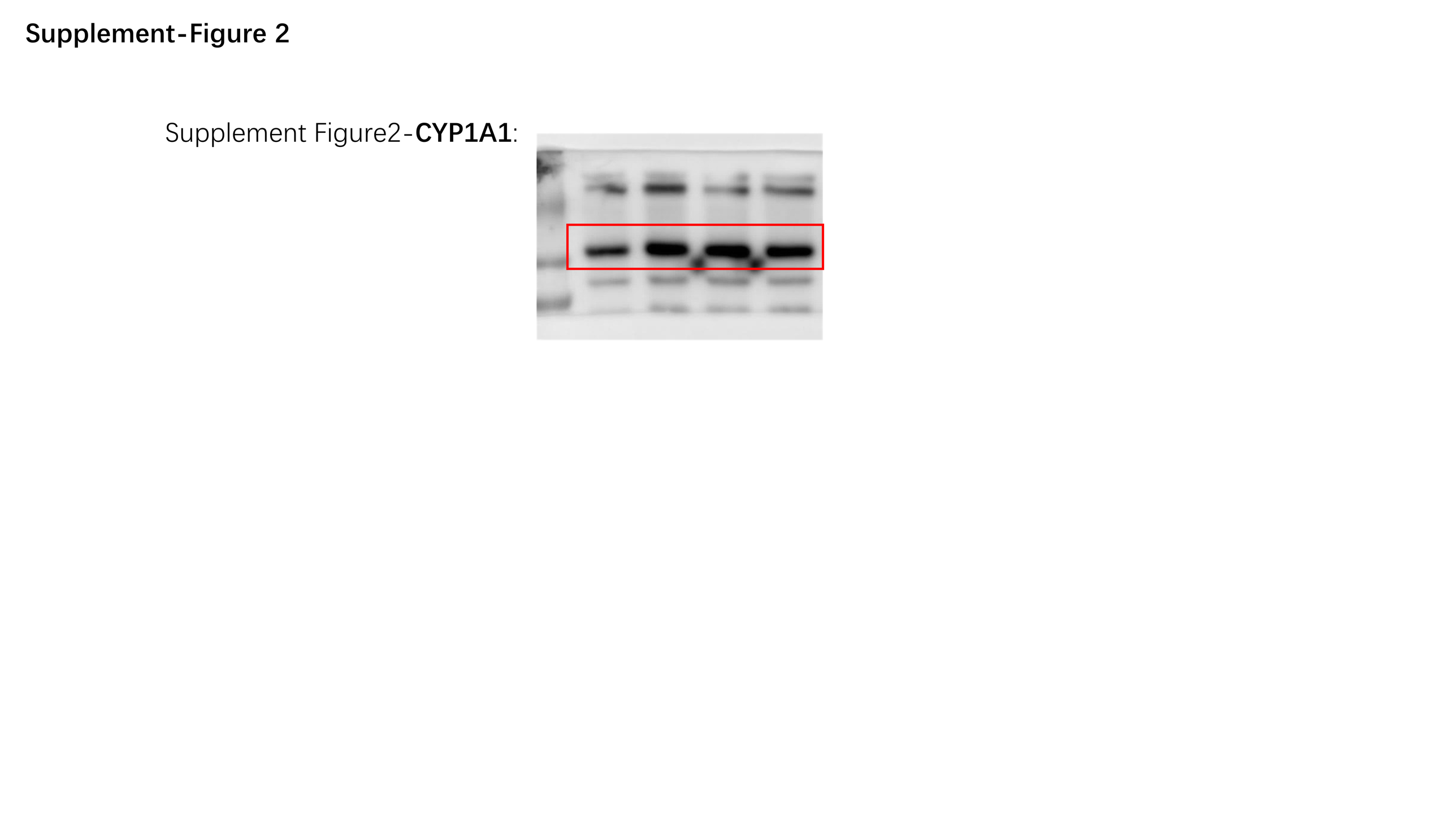

Supplement: Figure 7—figure supplement 1—source data 1. — (Figure 7—figure supplement 1B–CYP1A1) The expression of CYP1A1 expression in the PM2.5-treated HTR8/SVneo cells (PM2.5 concentration: 0. 50 μg/mL, 100 μg/mL, 200 μg/mL). (Figure 7—figure supplement 1B–CYP1B1) The expression of CYP1B1 expression in the PM2.5-treated HTR8/SVneo cells (PM2.5 concentration: 0. 50 μg/mL, 100 μg/mL, 200 μg/mL). (Figure 7—figure supplement 1B–ALDH1A3) The expression of ALDH1A3 expression in the PM2.5-treated HTR8/SVneo cells (PM2.5 concentration: 0. 50 μg/mL, 100 μg/mL, 200 μg/mL). (Figure 7—figure supplement 1B-β-actin) The expression of β-actin expression in the PM2.5-treated HTR8/SVneo cells (PM2.5 concentration: 0. 50 μg/mL, 100 μg/mL, 200 μg/mL). [file elife-85944-fig7-figsupp1-data1.zip › Figure 7-figure supplement 1-source data 1/Figure 7-figure supplement 1B-CYP1A1.TIF]

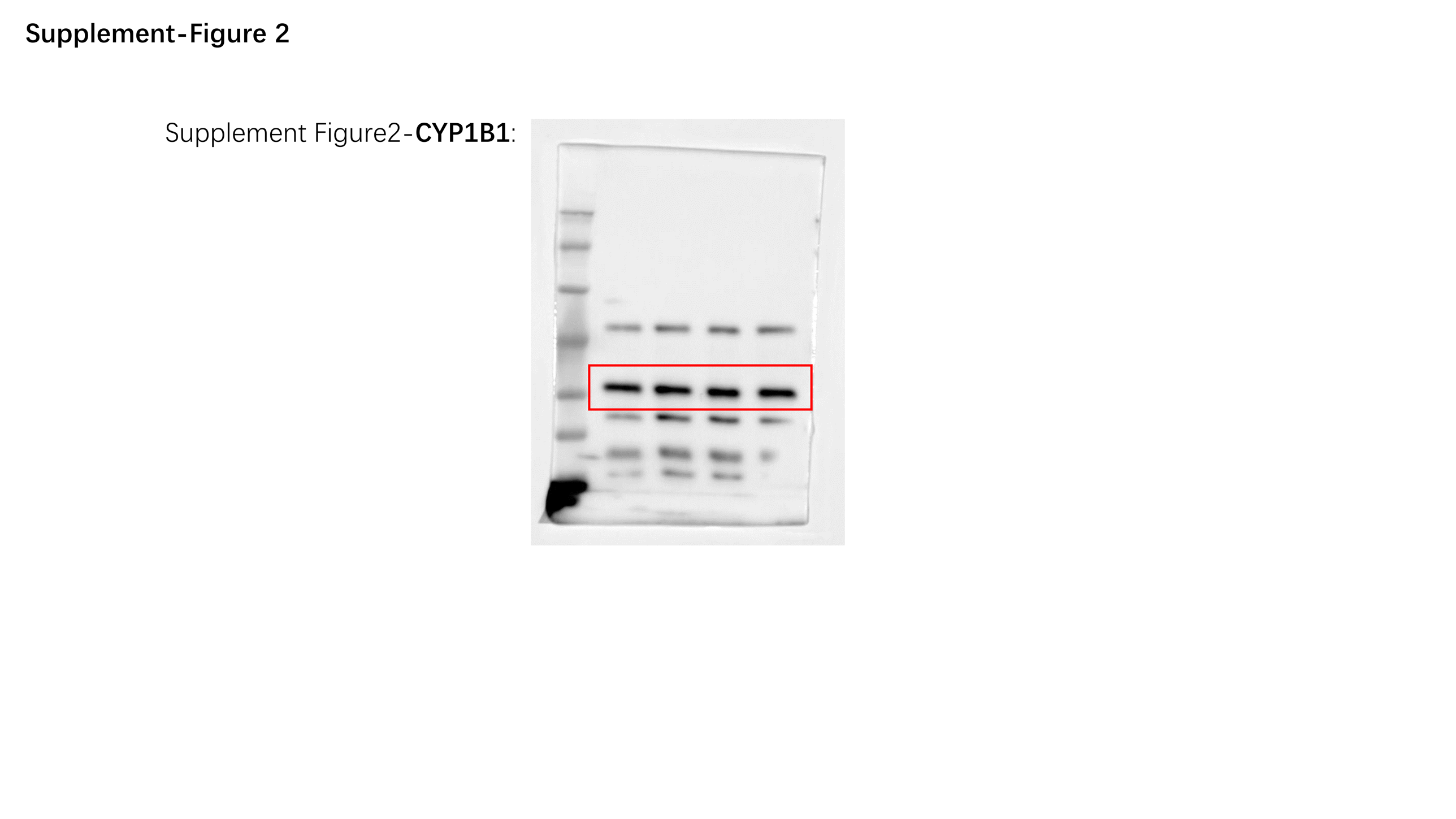

Supplement: Figure 7—figure supplement 1—source data 1. — (Figure 7—figure supplement 1B–CYP1A1) The expression of CYP1A1 expression in the PM2.5-treated HTR8/SVneo cells (PM2.5 concentration: 0. 50 μg/mL, 100 μg/mL, 200 μg/mL). (Figure 7—figure supplement 1B–CYP1B1) The expression of CYP1B1 expression in the PM2.5-treated HTR8/SVneo cells (PM2.5 concentration: 0. 50 μg/mL, 100 μg/mL, 200 μg/mL). (Figure 7—figure supplement 1B–ALDH1A3) The expression of ALDH1A3 expression in the PM2.5-treated HTR8/SVneo cells (PM2.5 concentration: 0. 50 μg/mL, 100 μg/mL, 200 μg/mL). (Figure 7—figure supplement 1B-β-actin) The expression of β-actin expression in the PM2.5-treated HTR8/SVneo cells (PM2.5 concentration: 0. 50 μg/mL, 100 μg/mL, 200 μg/mL). [file elife-85944-fig7-figsupp1-data1.zip › Figure 7-figure supplement 1-source data 1/Figure 7-figure supplement 1B-CYP1B1.TIF]

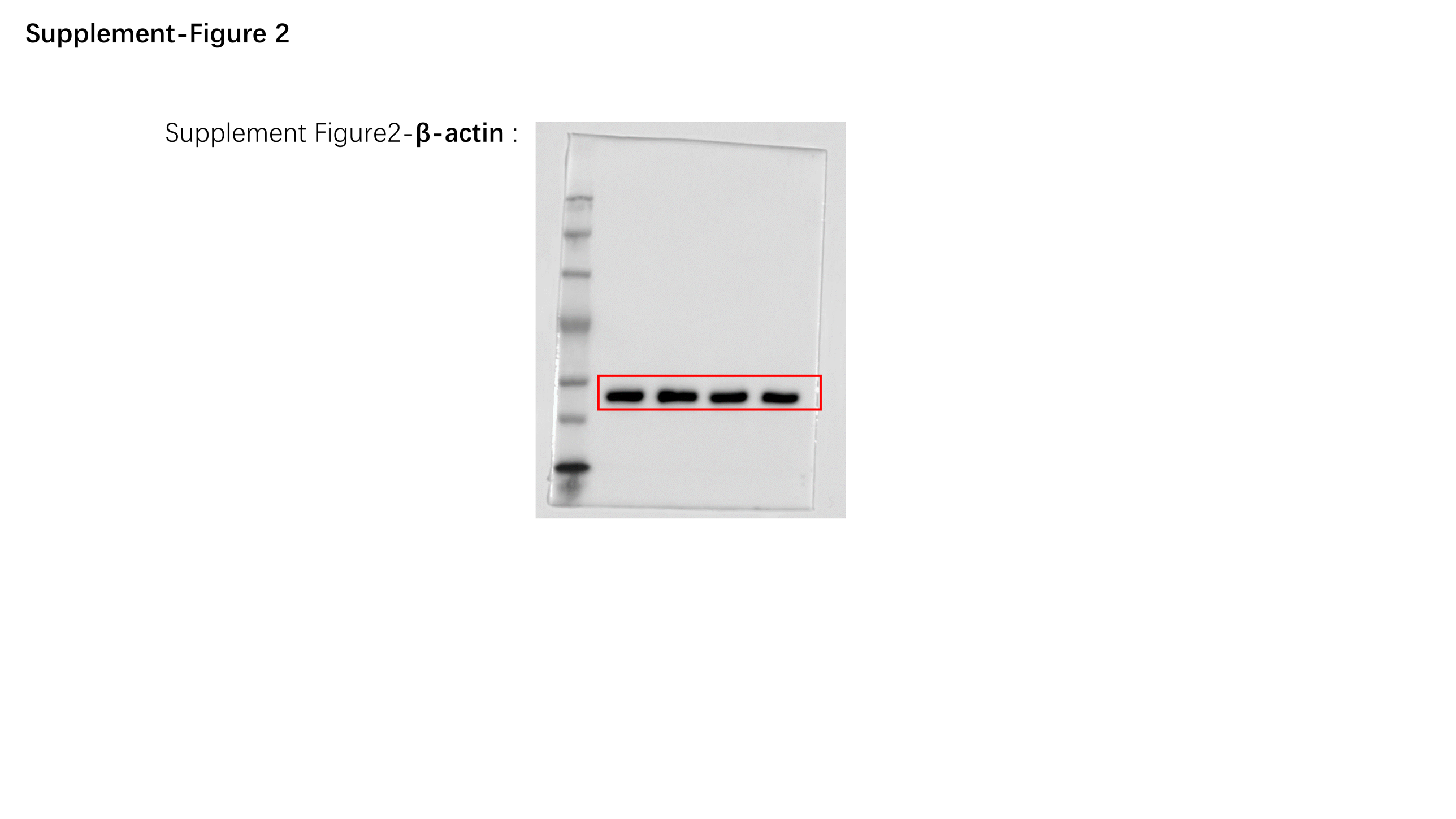

Supplement: Figure 7—figure supplement 1—source data 1. — (Figure 7—figure supplement 1B–CYP1A1) The expression of CYP1A1 expression in the PM2.5-treated HTR8/SVneo cells (PM2.5 concentration: 0. 50 μg/mL, 100 μg/mL, 200 μg/mL). (Figure 7—figure supplement 1B–CYP1B1) The expression of CYP1B1 expression in the PM2.5-treated HTR8/SVneo cells (PM2.5 concentration: 0. 50 μg/mL, 100 μg/mL, 200 μg/mL). (Figure 7—figure supplement 1B–ALDH1A3) The expression of ALDH1A3 expression in the PM2.5-treated HTR8/SVneo cells (PM2.5 concentration: 0. 50 μg/mL, 100 μg/mL, 200 μg/mL). (Figure 7—figure supplement 1B-β-actin) The expression of β-actin expression in the PM2.5-treated HTR8/SVneo cells (PM2.5 concentration: 0. 50 μg/mL, 100 μg/mL, 200 μg/mL). [file elife-85944-fig7-figsupp1-data1.zip › Figure 7-figure supplement 1-source data 1/Figure 7-figure supplement 1B-a┬-actin.TIF]

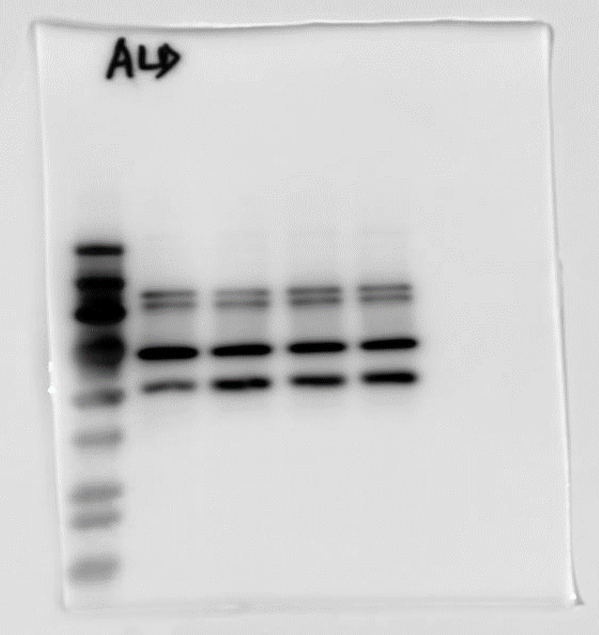

Supplement: Figure 7—figure supplement 1—source data 2. — (Figure 7—figure supplement 1B–CYP1A1) The expression of CYP1A1 expression in the PM2.5-treated HTR8/SVneo cells (PM2.5 concentration: 0. 50 μg/mL, 100 μg/mL, 200 μg/mL). (Figure 7—figure supplement 1B–CYP1B1) The expression of CYP1B1 expression in the PM2.5-treated HTR8/SVneo cells (PM2.5 concentration: 0. 50 μg/mL, 100 μg/mL, 200 μg/mL). (Figure 7—figure supplement 1B–ALDH1A3) The expression of ALDH1A3 expression in the PM2.5-treated HTR8/SVneo cells (PM2.5 concentration: 0. 50 μg/mL, 100 μg/mL, 200 μg/mL). (Figure 7—figure supplement 1B-β-actin) The expression of β-actin expression in the PM2.5-treated HTR8/SVneo cells (PM2.5 concentration: 0. 50 μg/mL, 100 μg/mL, 200 μg/mL). [file elife-85944-fig7-figsupp1-data2.zip › Figure7-figure supplement 1-source data 2/Figure7-figure supplement 1B-ALDH1A3.tif]

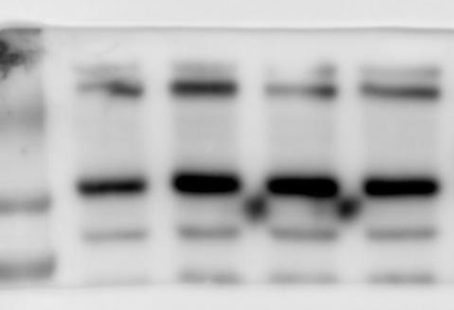

Supplement: Figure 7—figure supplement 1—source data 2. — (Figure 7—figure supplement 1B–CYP1A1) The expression of CYP1A1 expression in the PM2.5-treated HTR8/SVneo cells (PM2.5 concentration: 0. 50 μg/mL, 100 μg/mL, 200 μg/mL). (Figure 7—figure supplement 1B–CYP1B1) The expression of CYP1B1 expression in the PM2.5-treated HTR8/SVneo cells (PM2.5 concentration: 0. 50 μg/mL, 100 μg/mL, 200 μg/mL). (Figure 7—figure supplement 1B–ALDH1A3) The expression of ALDH1A3 expression in the PM2.5-treated HTR8/SVneo cells (PM2.5 concentration: 0. 50 μg/mL, 100 μg/mL, 200 μg/mL). (Figure 7—figure supplement 1B-β-actin) The expression of β-actin expression in the PM2.5-treated HTR8/SVneo cells (PM2.5 concentration: 0. 50 μg/mL, 100 μg/mL, 200 μg/mL). [file elife-85944-fig7-figsupp1-data2.zip › Figure7-figure supplement 1-source data 2/Figure7-figure supplement 1B-CYP1A1.tif]

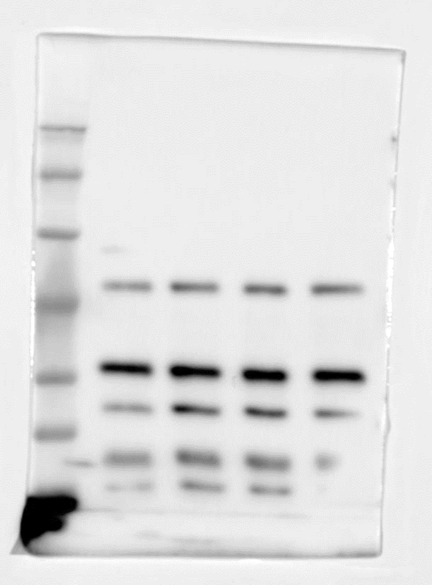

Supplement: Figure 7—figure supplement 1—source data 2. — (Figure 7—figure supplement 1B–CYP1A1) The expression of CYP1A1 expression in the PM2.5-treated HTR8/SVneo cells (PM2.5 concentration: 0. 50 μg/mL, 100 μg/mL, 200 μg/mL). (Figure 7—figure supplement 1B–CYP1B1) The expression of CYP1B1 expression in the PM2.5-treated HTR8/SVneo cells (PM2.5 concentration: 0. 50 μg/mL, 100 μg/mL, 200 μg/mL). (Figure 7—figure supplement 1B–ALDH1A3) The expression of ALDH1A3 expression in the PM2.5-treated HTR8/SVneo cells (PM2.5 concentration: 0. 50 μg/mL, 100 μg/mL, 200 μg/mL). (Figure 7—figure supplement 1B-β-actin) The expression of β-actin expression in the PM2.5-treated HTR8/SVneo cells (PM2.5 concentration: 0. 50 μg/mL, 100 μg/mL, 200 μg/mL). [file elife-85944-fig7-figsupp1-data2.zip › Figure7-figure supplement 1-source data 2/Figure7-figure supplement 1B-CYP1B1.tif]

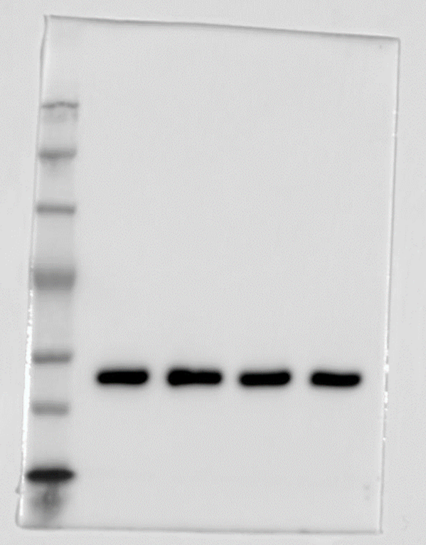

Supplement: Figure 7—figure supplement 1—source data 2. — (Figure 7—figure supplement 1B–CYP1A1) The expression of CYP1A1 expression in the PM2.5-treated HTR8/SVneo cells (PM2.5 concentration: 0. 50 μg/mL, 100 μg/mL, 200 μg/mL). (Figure 7—figure supplement 1B–CYP1B1) The expression of CYP1B1 expression in the PM2.5-treated HTR8/SVneo cells (PM2.5 concentration: 0. 50 μg/mL, 100 μg/mL, 200 μg/mL). (Figure 7—figure supplement 1B–ALDH1A3) The expression of ALDH1A3 expression in the PM2.5-treated HTR8/SVneo cells (PM2.5 concentration: 0. 50 μg/mL, 100 μg/mL, 200 μg/mL). (Figure 7—figure supplement 1B-β-actin) The expression of β-actin expression in the PM2.5-treated HTR8/SVneo cells (PM2.5 concentration: 0. 50 μg/mL, 100 μg/mL, 200 μg/mL). [file elife-85944-fig7-figsupp1-data2.zip › Figure7-figure supplement 1-source data 2/Figure7-figure supplement 1B-a┬-actin.tif]

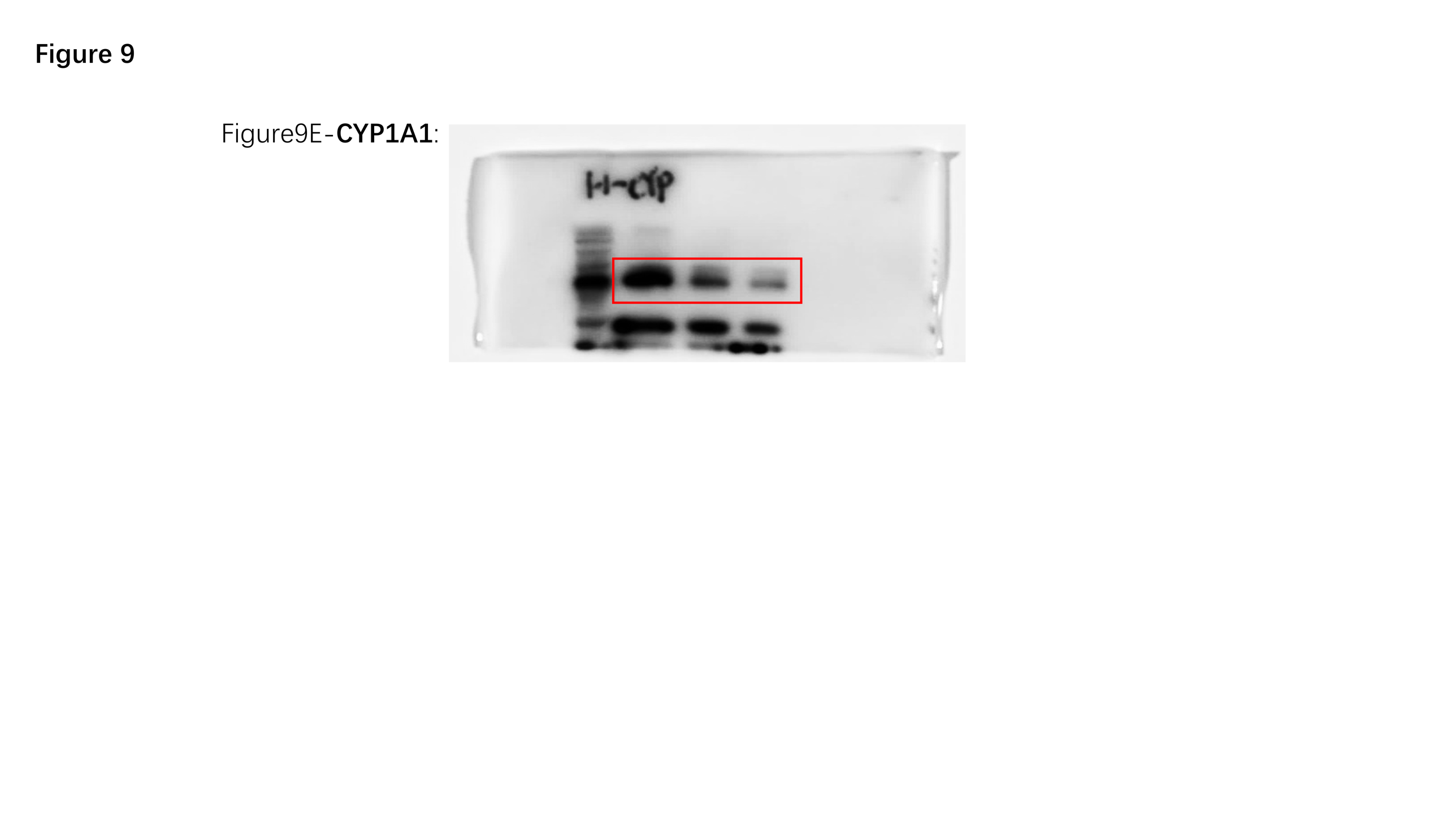

Supplement: Figure 9—source data 1. — (Figure 9E-KLF9) The expression of KLF9 expression in the HTR8/SVneo (si-NC, si- KLF9#1, si- KLF9#2). (Figure 9E-CYP1A1) The expression of CYP1A1 expression in the HTR8/SVneo (si-NC, si- KLF9#1, si- KLF9#2). (Figure 9E-β-actin) The expression of β-actin expression in the HTR8/SVneo (si-NC, si- KLF9#1, si- KLF9#2). (Figure 9E-Vector-KLF9-KLF9) The expression of KLF9 expression in the HTR8/SVneo (CON, KLF9 over expression). (Figure 9E-Vector-KLF9-CYP1A1) The expression of CYP1A1 expression in the HTR8/SVneo (CON, KLF9 over expression). (Figure 9E-Vector-KLF9-β-actin) The expression of β-actin expression in the HTR8/SVneo (CON, KLF9 over expression). (Figure 9G-ChIP) Chromatin from KLF9 over-expression HTR8/SVneo cells was subjected to ChIP assay using KLF9 antibody or control IgG. PCR amplification with primers spanning the –96/+16 bp region of the CYP1A1 promoter was performed. A 2% agarose gel electrophoresis was performed on PCR products. [file elife-85944-fig9-data1.zip › Figure 9-source data 1/Figure9E-CYP1A1.TIF]

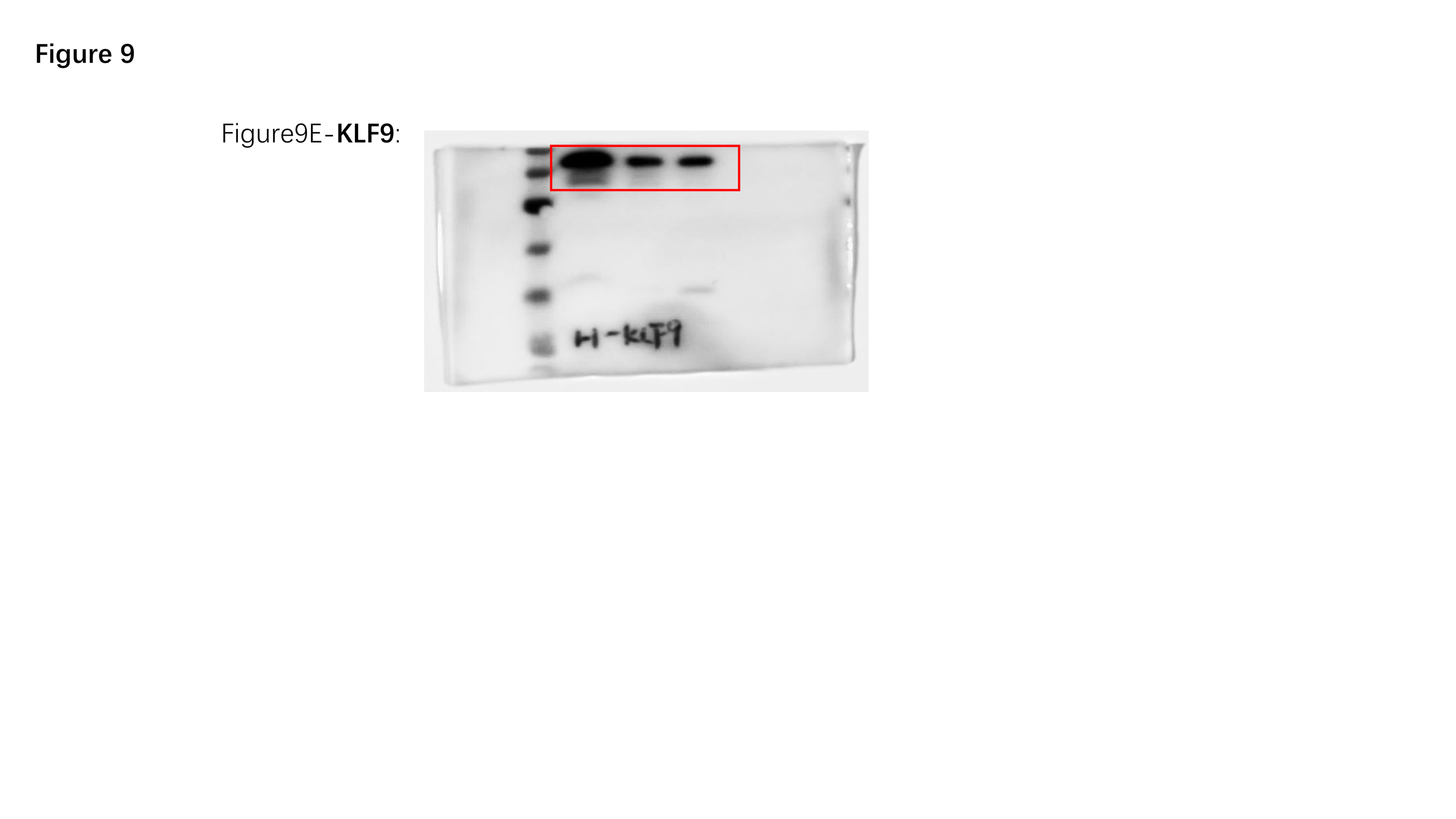

Supplement: Figure 9—source data 1. — (Figure 9E-KLF9) The expression of KLF9 expression in the HTR8/SVneo (si-NC, si- KLF9#1, si- KLF9#2). (Figure 9E-CYP1A1) The expression of CYP1A1 expression in the HTR8/SVneo (si-NC, si- KLF9#1, si- KLF9#2). (Figure 9E-β-actin) The expression of β-actin expression in the HTR8/SVneo (si-NC, si- KLF9#1, si- KLF9#2). (Figure 9E-Vector-KLF9-KLF9) The expression of KLF9 expression in the HTR8/SVneo (CON, KLF9 over expression). (Figure 9E-Vector-KLF9-CYP1A1) The expression of CYP1A1 expression in the HTR8/SVneo (CON, KLF9 over expression). (Figure 9E-Vector-KLF9-β-actin) The expression of β-actin expression in the HTR8/SVneo (CON, KLF9 over expression). (Figure 9G-ChIP) Chromatin from KLF9 over-expression HTR8/SVneo cells was subjected to ChIP assay using KLF9 antibody or control IgG. PCR amplification with primers spanning the –96/+16 bp region of the CYP1A1 promoter was performed. A 2% agarose gel electrophoresis was performed on PCR products. [file elife-85944-fig9-data1.zip › Figure 9-source data 1/Figure9E-KLF9.TIF]

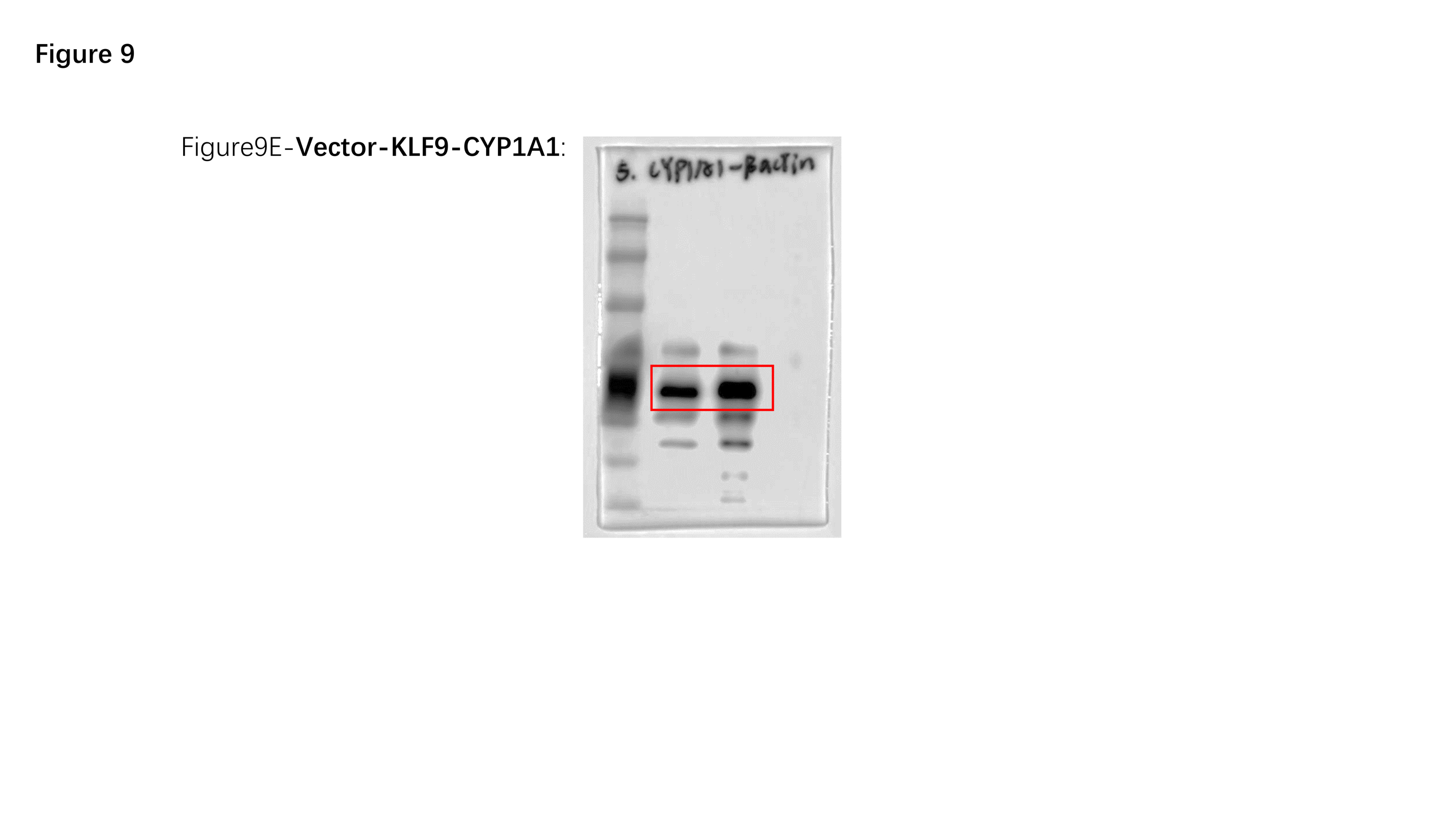

Supplement: Figure 9—source data 1. — (Figure 9E-KLF9) The expression of KLF9 expression in the HTR8/SVneo (si-NC, si- KLF9#1, si- KLF9#2). (Figure 9E-CYP1A1) The expression of CYP1A1 expression in the HTR8/SVneo (si-NC, si- KLF9#1, si- KLF9#2). (Figure 9E-β-actin) The expression of β-actin expression in the HTR8/SVneo (si-NC, si- KLF9#1, si- KLF9#2). (Figure 9E-Vector-KLF9-KLF9) The expression of KLF9 expression in the HTR8/SVneo (CON, KLF9 over expression). (Figure 9E-Vector-KLF9-CYP1A1) The expression of CYP1A1 expression in the HTR8/SVneo (CON, KLF9 over expression). (Figure 9E-Vector-KLF9-β-actin) The expression of β-actin expression in the HTR8/SVneo (CON, KLF9 over expression). (Figure 9G-ChIP) Chromatin from KLF9 over-expression HTR8/SVneo cells was subjected to ChIP assay using KLF9 antibody or control IgG. PCR amplification with primers spanning the –96/+16 bp region of the CYP1A1 promoter was performed. A 2% agarose gel electrophoresis was performed on PCR products. [file elife-85944-fig9-data1.zip › Figure 9-source data 1/Figure9E-Vector-KLF9-CYP1A1.TIF]

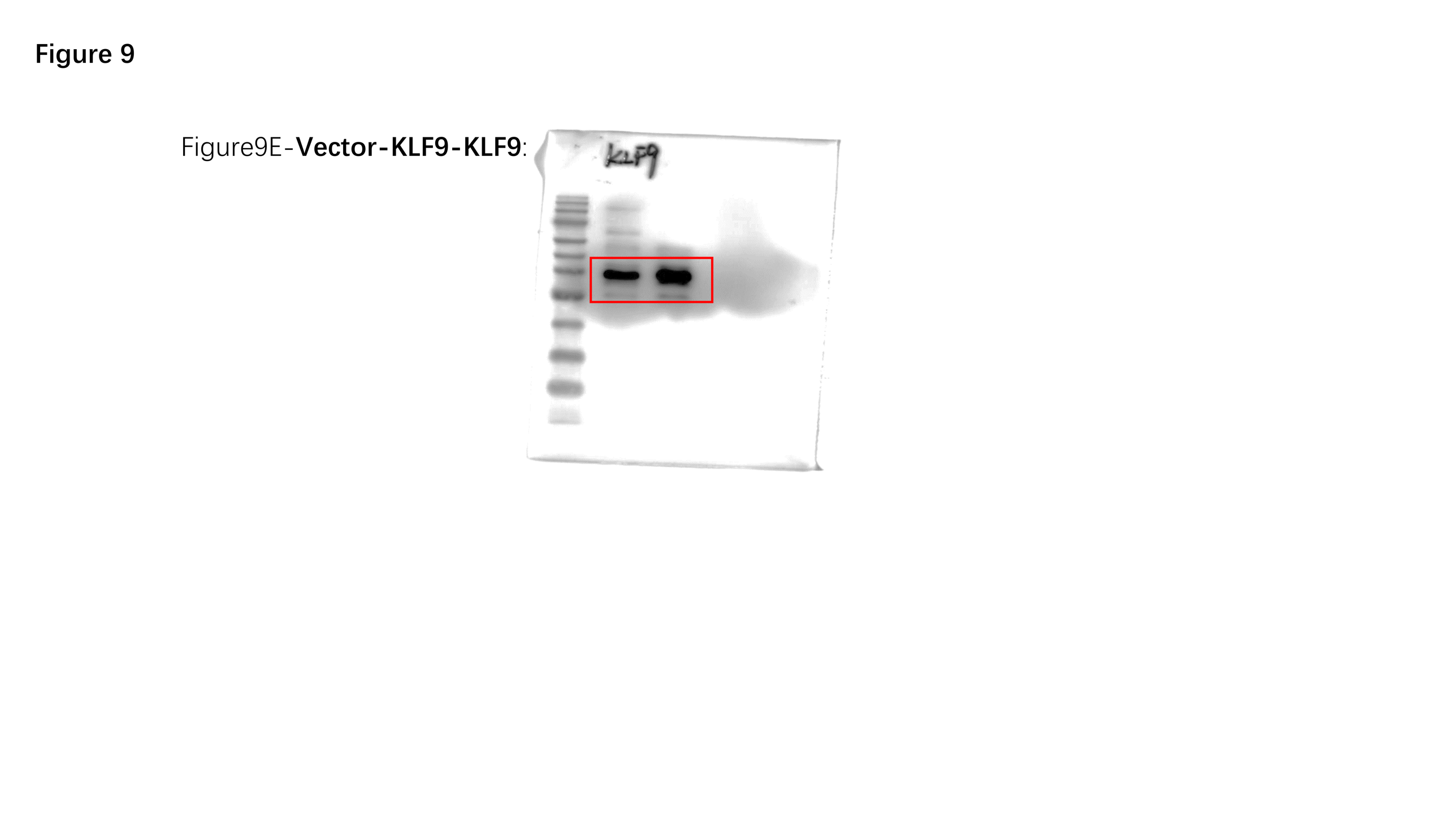

Supplement: Figure 9—source data 1. — (Figure 9E-KLF9) The expression of KLF9 expression in the HTR8/SVneo (si-NC, si- KLF9#1, si- KLF9#2). (Figure 9E-CYP1A1) The expression of CYP1A1 expression in the HTR8/SVneo (si-NC, si- KLF9#1, si- KLF9#2). (Figure 9E-β-actin) The expression of β-actin expression in the HTR8/SVneo (si-NC, si- KLF9#1, si- KLF9#2). (Figure 9E-Vector-KLF9-KLF9) The expression of KLF9 expression in the HTR8/SVneo (CON, KLF9 over expression). (Figure 9E-Vector-KLF9-CYP1A1) The expression of CYP1A1 expression in the HTR8/SVneo (CON, KLF9 over expression). (Figure 9E-Vector-KLF9-β-actin) The expression of β-actin expression in the HTR8/SVneo (CON, KLF9 over expression). (Figure 9G-ChIP) Chromatin from KLF9 over-expression HTR8/SVneo cells was subjected to ChIP assay using KLF9 antibody or control IgG. PCR amplification with primers spanning the –96/+16 bp region of the CYP1A1 promoter was performed. A 2% agarose gel electrophoresis was performed on PCR products. [file elife-85944-fig9-data1.zip › Figure 9-source data 1/Figure9E-Vector-KLF9-KLF9.TIF]

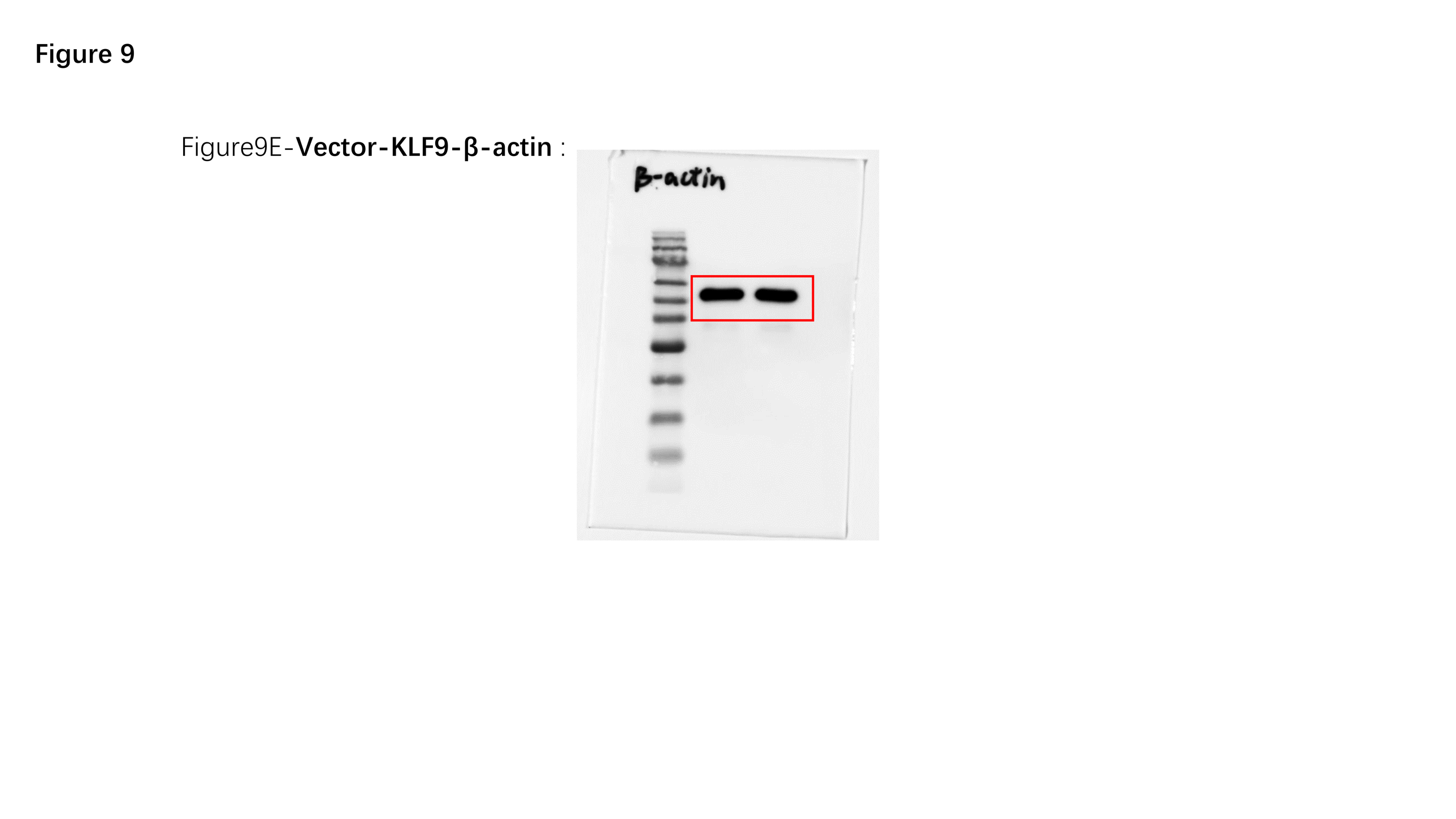

Supplement: Figure 9—source data 1. — (Figure 9E-KLF9) The expression of KLF9 expression in the HTR8/SVneo (si-NC, si- KLF9#1, si- KLF9#2). (Figure 9E-CYP1A1) The expression of CYP1A1 expression in the HTR8/SVneo (si-NC, si- KLF9#1, si- KLF9#2). (Figure 9E-β-actin) The expression of β-actin expression in the HTR8/SVneo (si-NC, si- KLF9#1, si- KLF9#2). (Figure 9E-Vector-KLF9-KLF9) The expression of KLF9 expression in the HTR8/SVneo (CON, KLF9 over expression). (Figure 9E-Vector-KLF9-CYP1A1) The expression of CYP1A1 expression in the HTR8/SVneo (CON, KLF9 over expression). (Figure 9E-Vector-KLF9-β-actin) The expression of β-actin expression in the HTR8/SVneo (CON, KLF9 over expression). (Figure 9G-ChIP) Chromatin from KLF9 over-expression HTR8/SVneo cells was subjected to ChIP assay using KLF9 antibody or control IgG. PCR amplification with primers spanning the –96/+16 bp region of the CYP1A1 promoter was performed. A 2% agarose gel electrophoresis was performed on PCR products. [file elife-85944-fig9-data1.zip › Figure 9-source data 1/Figure9E-Vector-KLF9-a┬-actin .TIF]

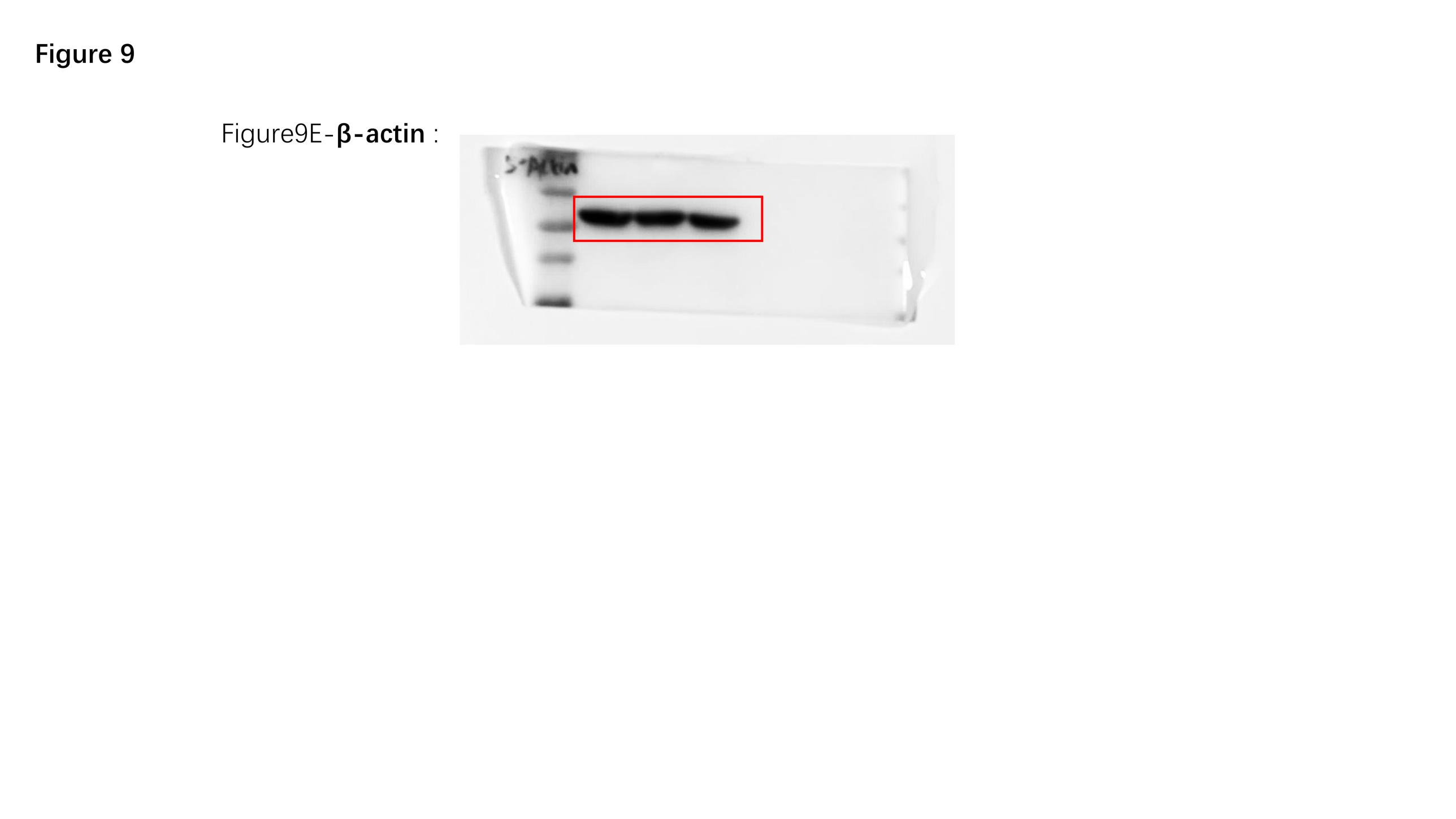

Supplement: Figure 9—source data 1. — (Figure 9E-KLF9) The expression of KLF9 expression in the HTR8/SVneo (si-NC, si- KLF9#1, si- KLF9#2). (Figure 9E-CYP1A1) The expression of CYP1A1 expression in the HTR8/SVneo (si-NC, si- KLF9#1, si- KLF9#2). (Figure 9E-β-actin) The expression of β-actin expression in the HTR8/SVneo (si-NC, si- KLF9#1, si- KLF9#2). (Figure 9E-Vector-KLF9-KLF9) The expression of KLF9 expression in the HTR8/SVneo (CON, KLF9 over expression). (Figure 9E-Vector-KLF9-CYP1A1) The expression of CYP1A1 expression in the HTR8/SVneo (CON, KLF9 over expression). (Figure 9E-Vector-KLF9-β-actin) The expression of β-actin expression in the HTR8/SVneo (CON, KLF9 over expression). (Figure 9G-ChIP) Chromatin from KLF9 over-expression HTR8/SVneo cells was subjected to ChIP assay using KLF9 antibody or control IgG. PCR amplification with primers spanning the –96/+16 bp region of the CYP1A1 promoter was performed. A 2% agarose gel electrophoresis was performed on PCR products. [file elife-85944-fig9-data1.zip › Figure 9-source data 1/Figure9E-a┬-actin .TIF]

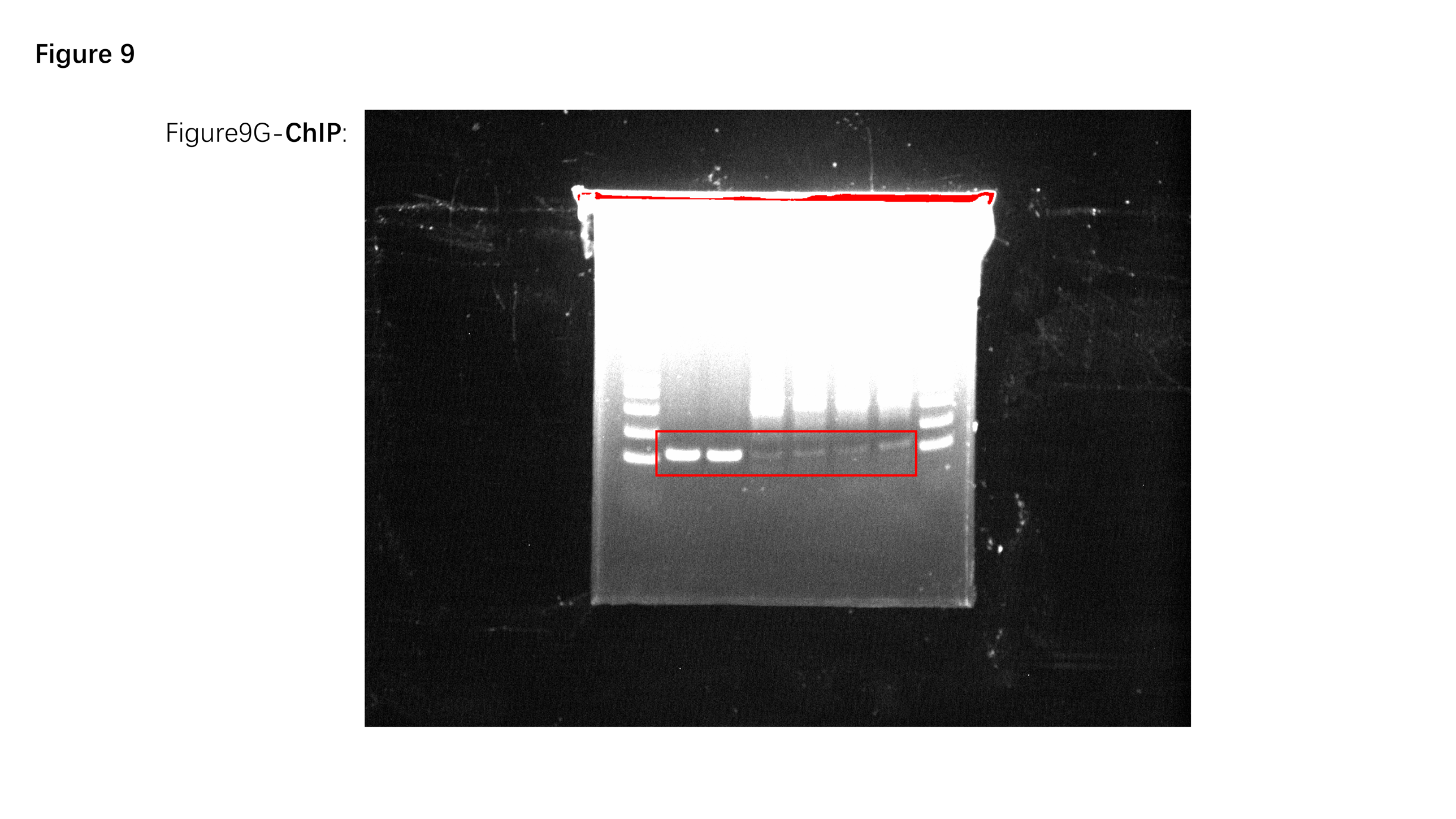

Supplement: Figure 9—source data 1. — (Figure 9E-KLF9) The expression of KLF9 expression in the HTR8/SVneo (si-NC, si- KLF9#1, si- KLF9#2). (Figure 9E-CYP1A1) The expression of CYP1A1 expression in the HTR8/SVneo (si-NC, si- KLF9#1, si- KLF9#2). (Figure 9E-β-actin) The expression of β-actin expression in the HTR8/SVneo (si-NC, si- KLF9#1, si- KLF9#2). (Figure 9E-Vector-KLF9-KLF9) The expression of KLF9 expression in the HTR8/SVneo (CON, KLF9 over expression). (Figure 9E-Vector-KLF9-CYP1A1) The expression of CYP1A1 expression in the HTR8/SVneo (CON, KLF9 over expression). (Figure 9E-Vector-KLF9-β-actin) The expression of β-actin expression in the HTR8/SVneo (CON, KLF9 over expression). (Figure 9G-ChIP) Chromatin from KLF9 over-expression HTR8/SVneo cells was subjected to ChIP assay using KLF9 antibody or control IgG. PCR amplification with primers spanning the –96/+16 bp region of the CYP1A1 promoter was performed. A 2% agarose gel electrophoresis was performed on PCR products. [file elife-85944-fig9-data1.zip › Figure 9-source data 1/Figure9G-ChIP.TIF]

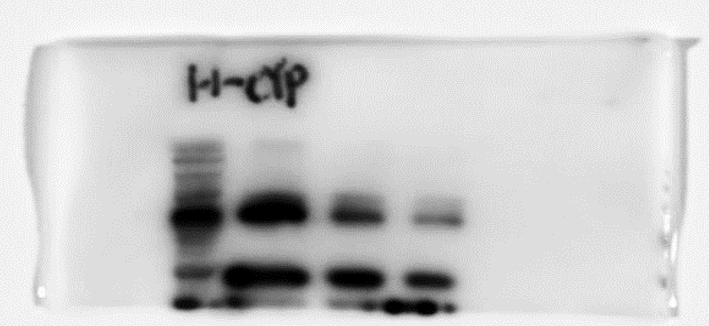

Supplement: Figure 9—source data 2. — (Figure 9E-KLF9) The expression of KLF9 expression in the HTR8/SVneo (si-NC, si- KLF9#1, si- KLF9#2). (Figure 9E-CYP1A1) The expression of CYP1A1 expression in the HTR8/SVneo (si-NC, si- KLF9#1, si- KLF9#2). (Figure 9E-β-actin) The expression of β-actin expression in the HTR8/SVneo (si-NC, si- KLF9#1, si- KLF9#2). (Figure 9E-Vector-KLF9-KLF9) The expression of KLF9 expression in the HTR8/SVneo (CON, KLF9 over expression). (Figure 9E-Vector-KLF9-CYP1A1) The expression of CYP1A1 expression in the HTR8/SVneo (CON, KLF9 over expression). (Figure 9E-Vector-KLF9-β-actin) The expression of β-actin expression in the HTR8/SVneo (CON, KLF9 over expression). (Figure 9G-ChIP) Chromatin from KLF9 over-expression HTR8/SVneo cells was subjected to ChIP assay using KLF9 antibody or control IgG. PCR amplification with primers spanning the –96/+16 bp region of the CYP1A1 promoter was performed. A 2% agarose gel electrophoresis was performed on PCR products. [file elife-85944-fig9-data2.zip › Figure 9-source data 2/Figure9E-CYP1A1.tif]

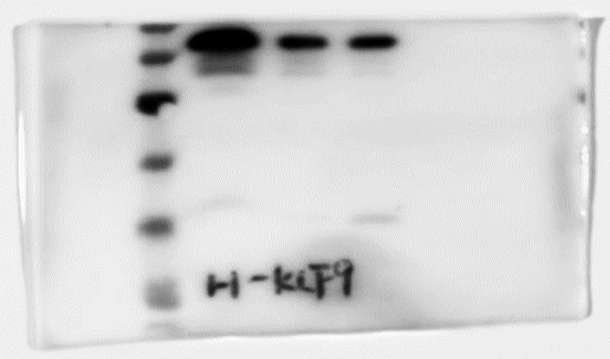

Supplement: Figure 9—source data 2. — (Figure 9E-KLF9) The expression of KLF9 expression in the HTR8/SVneo (si-NC, si- KLF9#1, si- KLF9#2). (Figure 9E-CYP1A1) The expression of CYP1A1 expression in the HTR8/SVneo (si-NC, si- KLF9#1, si- KLF9#2). (Figure 9E-β-actin) The expression of β-actin expression in the HTR8/SVneo (si-NC, si- KLF9#1, si- KLF9#2). (Figure 9E-Vector-KLF9-KLF9) The expression of KLF9 expression in the HTR8/SVneo (CON, KLF9 over expression). (Figure 9E-Vector-KLF9-CYP1A1) The expression of CYP1A1 expression in the HTR8/SVneo (CON, KLF9 over expression). (Figure 9E-Vector-KLF9-β-actin) The expression of β-actin expression in the HTR8/SVneo (CON, KLF9 over expression). (Figure 9G-ChIP) Chromatin from KLF9 over-expression HTR8/SVneo cells was subjected to ChIP assay using KLF9 antibody or control IgG. PCR amplification with primers spanning the –96/+16 bp region of the CYP1A1 promoter was performed. A 2% agarose gel electrophoresis was performed on PCR products. [file elife-85944-fig9-data2.zip › Figure 9-source data 2/Figure9E-KLF9.tif]

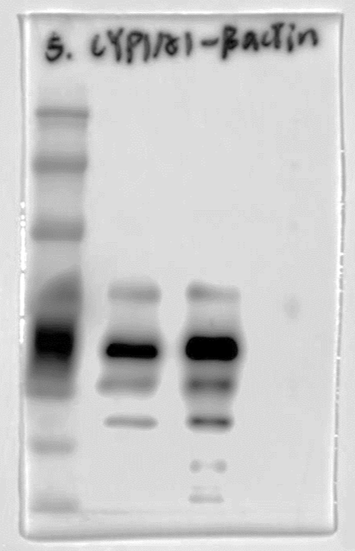

Supplement: Figure 9—source data 2. — (Figure 9E-KLF9) The expression of KLF9 expression in the HTR8/SVneo (si-NC, si- KLF9#1, si- KLF9#2). (Figure 9E-CYP1A1) The expression of CYP1A1 expression in the HTR8/SVneo (si-NC, si- KLF9#1, si- KLF9#2). (Figure 9E-β-actin) The expression of β-actin expression in the HTR8/SVneo (si-NC, si- KLF9#1, si- KLF9#2). (Figure 9E-Vector-KLF9-KLF9) The expression of KLF9 expression in the HTR8/SVneo (CON, KLF9 over expression). (Figure 9E-Vector-KLF9-CYP1A1) The expression of CYP1A1 expression in the HTR8/SVneo (CON, KLF9 over expression). (Figure 9E-Vector-KLF9-β-actin) The expression of β-actin expression in the HTR8/SVneo (CON, KLF9 over expression). (Figure 9G-ChIP) Chromatin from KLF9 over-expression HTR8/SVneo cells was subjected to ChIP assay using KLF9 antibody or control IgG. PCR amplification with primers spanning the –96/+16 bp region of the CYP1A1 promoter was performed. A 2% agarose gel electrophoresis was performed on PCR products. [file elife-85944-fig9-data2.zip › Figure 9-source data 2/Figure9E-Vector-KLF9-CYP1A1.tif]

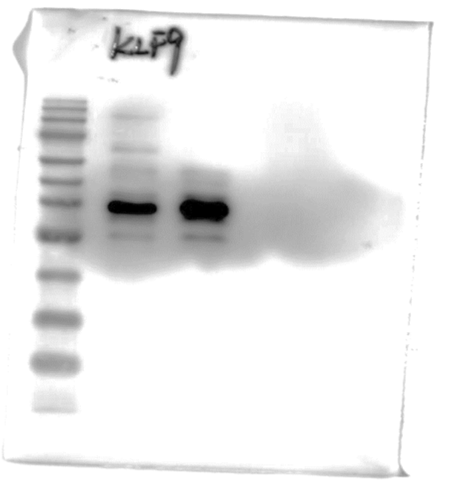

Supplement: Figure 9—source data 2. — (Figure 9E-KLF9) The expression of KLF9 expression in the HTR8/SVneo (si-NC, si- KLF9#1, si- KLF9#2). (Figure 9E-CYP1A1) The expression of CYP1A1 expression in the HTR8/SVneo (si-NC, si- KLF9#1, si- KLF9#2). (Figure 9E-β-actin) The expression of β-actin expression in the HTR8/SVneo (si-NC, si- KLF9#1, si- KLF9#2). (Figure 9E-Vector-KLF9-KLF9) The expression of KLF9 expression in the HTR8/SVneo (CON, KLF9 over expression). (Figure 9E-Vector-KLF9-CYP1A1) The expression of CYP1A1 expression in the HTR8/SVneo (CON, KLF9 over expression). (Figure 9E-Vector-KLF9-β-actin) The expression of β-actin expression in the HTR8/SVneo (CON, KLF9 over expression). (Figure 9G-ChIP) Chromatin from KLF9 over-expression HTR8/SVneo cells was subjected to ChIP assay using KLF9 antibody or control IgG. PCR amplification with primers spanning the –96/+16 bp region of the CYP1A1 promoter was performed. A 2% agarose gel electrophoresis was performed on PCR products. [file elife-85944-fig9-data2.zip › Figure 9-source data 2/Figure9E-Vector-KLF9-KLF9.tif]

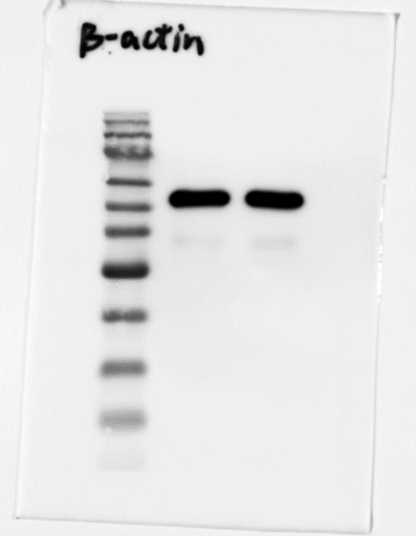

Supplement: Figure 9—source data 2. — (Figure 9E-KLF9) The expression of KLF9 expression in the HTR8/SVneo (si-NC, si- KLF9#1, si- KLF9#2). (Figure 9E-CYP1A1) The expression of CYP1A1 expression in the HTR8/SVneo (si-NC, si- KLF9#1, si- KLF9#2). (Figure 9E-β-actin) The expression of β-actin expression in the HTR8/SVneo (si-NC, si- KLF9#1, si- KLF9#2). (Figure 9E-Vector-KLF9-KLF9) The expression of KLF9 expression in the HTR8/SVneo (CON, KLF9 over expression). (Figure 9E-Vector-KLF9-CYP1A1) The expression of CYP1A1 expression in the HTR8/SVneo (CON, KLF9 over expression). (Figure 9E-Vector-KLF9-β-actin) The expression of β-actin expression in the HTR8/SVneo (CON, KLF9 over expression). (Figure 9G-ChIP) Chromatin from KLF9 over-expression HTR8/SVneo cells was subjected to ChIP assay using KLF9 antibody or control IgG. PCR amplification with primers spanning the –96/+16 bp region of the CYP1A1 promoter was performed. A 2% agarose gel electrophoresis was performed on PCR products. [file elife-85944-fig9-data2.zip › Figure 9-source data 2/Figure9E-Vector-KLF9-a┬-actin.tif]

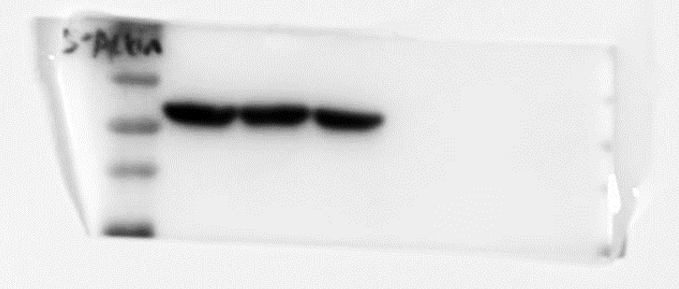

Supplement: Figure 9—source data 2. — (Figure 9E-KLF9) The expression of KLF9 expression in the HTR8/SVneo (si-NC, si- KLF9#1, si- KLF9#2). (Figure 9E-CYP1A1) The expression of CYP1A1 expression in the HTR8/SVneo (si-NC, si- KLF9#1, si- KLF9#2). (Figure 9E-β-actin) The expression of β-actin expression in the HTR8/SVneo (si-NC, si- KLF9#1, si- KLF9#2). (Figure 9E-Vector-KLF9-KLF9) The expression of KLF9 expression in the HTR8/SVneo (CON, KLF9 over expression). (Figure 9E-Vector-KLF9-CYP1A1) The expression of CYP1A1 expression in the HTR8/SVneo (CON, KLF9 over expression). (Figure 9E-Vector-KLF9-β-actin) The expression of β-actin expression in the HTR8/SVneo (CON, KLF9 over expression). (Figure 9G-ChIP) Chromatin from KLF9 over-expression HTR8/SVneo cells was subjected to ChIP assay using KLF9 antibody or control IgG. PCR amplification with primers spanning the –96/+16 bp region of the CYP1A1 promoter was performed. A 2% agarose gel electrophoresis was performed on PCR products. [file elife-85944-fig9-data2.zip › Figure 9-source data 2/Figure9E-a┬-actin.tif]

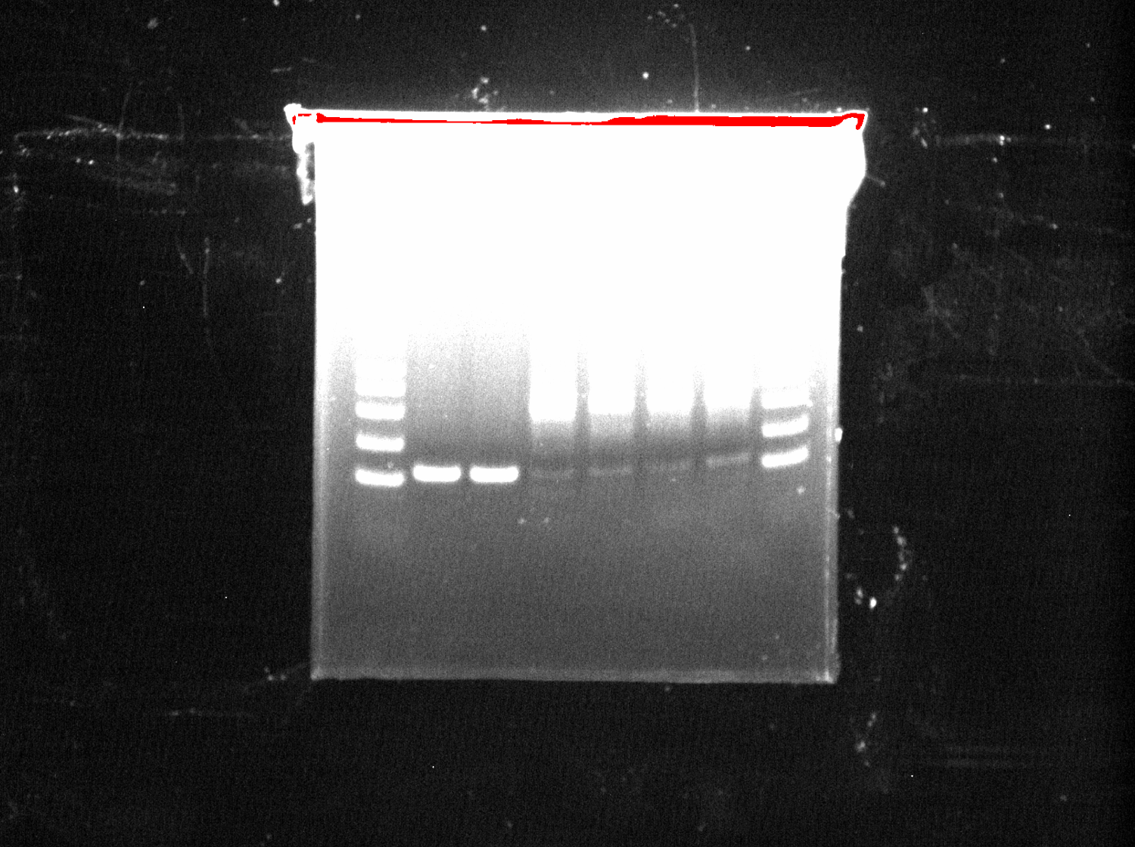

Supplement: Figure 9—source data 2. — (Figure 9E-KLF9) The expression of KLF9 expression in the HTR8/SVneo (si-NC, si- KLF9#1, si- KLF9#2). (Figure 9E-CYP1A1) The expression of CYP1A1 expression in the HTR8/SVneo (si-NC, si- KLF9#1, si- KLF9#2). (Figure 9E-β-actin) The expression of β-actin expression in the HTR8/SVneo (si-NC, si- KLF9#1, si- KLF9#2). (Figure 9E-Vector-KLF9-KLF9) The expression of KLF9 expression in the HTR8/SVneo (CON, KLF9 over expression). (Figure 9E-Vector-KLF9-CYP1A1) The expression of CYP1A1 expression in the HTR8/SVneo (CON, KLF9 over expression). (Figure 9E-Vector-KLF9-β-actin) The expression of β-actin expression in the HTR8/SVneo (CON, KLF9 over expression). (Figure 9G-ChIP) Chromatin from KLF9 over-expression HTR8/SVneo cells was subjected to ChIP assay using KLF9 antibody or control IgG. PCR amplification with primers spanning the –96/+16 bp region of the CYP1A1 promoter was performed. A 2% agarose gel electrophoresis was performed on PCR products. [file elife-85944-fig9-data2.zip › Figure 9-source data 2/Figure9G-ChIP.tif]

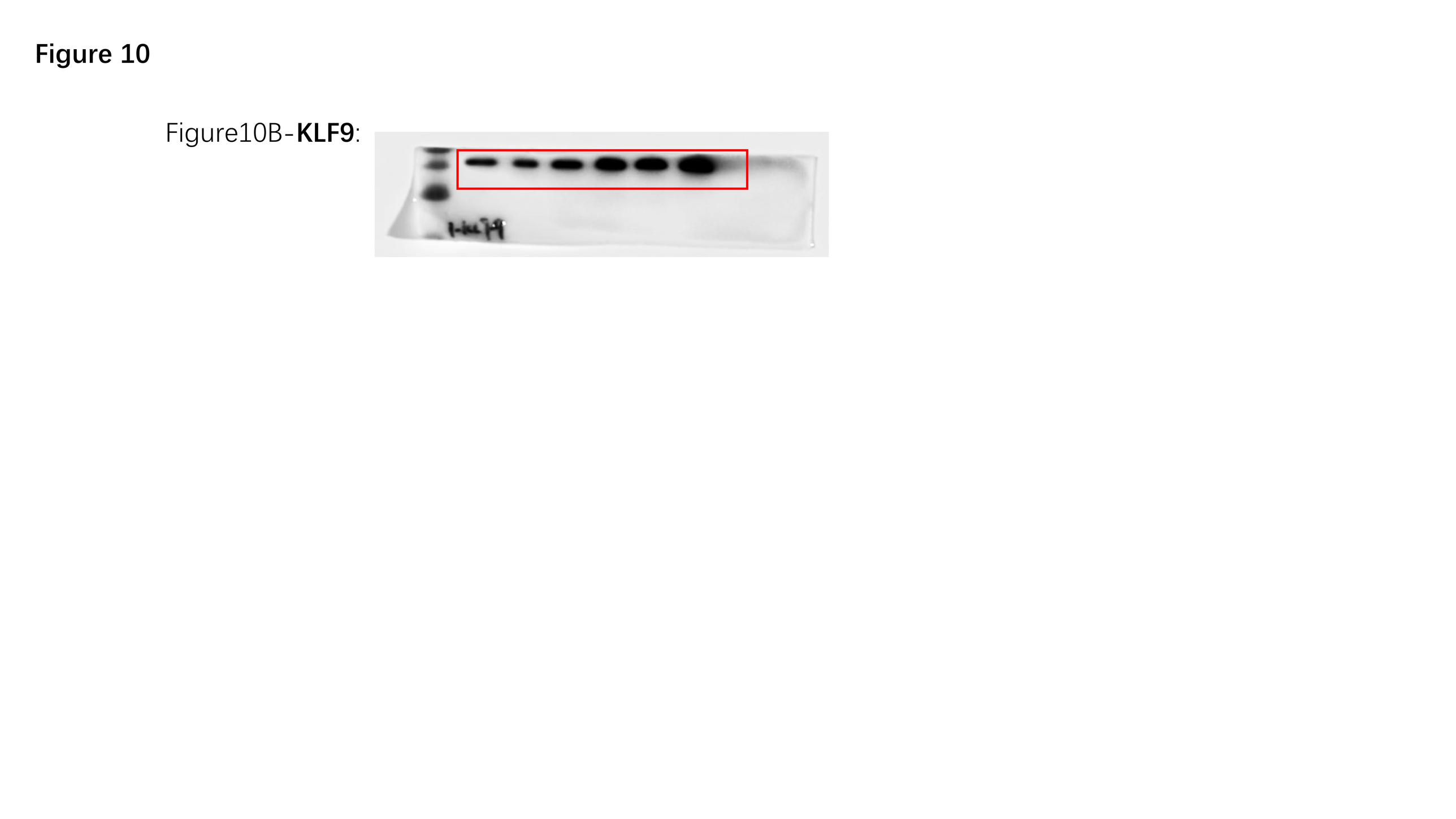

Supplement: Figure 10—source data 1. — (Figure 10B-KLF9) The expression of KLF9 expression in the placental tissue of Wild Type (The first three columns) and PM2.5-treated mice (the last three columns). (Figure 10B-β-actin) The expression of β-actin expression in the placental tissue of Wild Type (The first three columns) and PM2.5-treated mice (the last three columns). (Figure 10C-KLF9) The expression of KLF9 expression in the PM2.5-treated HTR8/SVneo cells (PM2.5 concentration: 0. 50 μg/mL, 100 μg/mL, 200 μg/mL). (Figure 10C-β-actin) The expression of β-actin expression in the PM2.5-treated HTR8/SVneo cells (PM2.5 concentration: 0. 50 μg/mL, 100 μg/mL, 200 μg/mL). (Figure 10K-Mito-Cytc) The expression of Cytc expression in mitochondria of HTR8/SVneo (si-NC, PM2.5+si NC, PM2.5+si-KLF9#1, PM2.5+si-KLF9#2). (Figure 10K-Mito-VDAC-1) The expression of VDAC-1 expression in mitochondria of HTR8/SVneo (si-NC, PM2.5+si NC, PM2.5+si-KLF9#1, PM2.5+si-KLF9#2). (Figure 10K-Cyto-Cytc) The expression of Cytc expression in cytoplasm of HTR8/SVneo (si-NC, PM2.5+si NC, PM2.5+si-KLF9#1, PM2.5+si-KLF9#2). (Figure 10K-Cyto-BCL-2) The expression of BCL-2 expression in cytoplasm of HTR8/SVneo (si-NC, PM2.5+si NC, PM2.5+si- KLF9#1, PM2.5+si- KLF9#2). (Figure 10K-Cyto-BAX) The expression of BAX expression in cytoplasm of HTR8/SVneo (si-NC, PM2.5+si NC, PM2.5+si- KLF9#1, PM2.5+si- KLF9#2). (Figure 10K-Cyto-Cleaved-Caspase3) The expression of Cleaved-Caspase3 expression in cytoplasm of HTR8/SVneo (si-NC, PM2.5+si NC, PM2.5+si- KLF9#1, PM2.5+si- KLF9#2). (Figure 10K-Cyto-β-actin) The expression of β-actin expression in cytoplasm of HTR8/SVneo (si-NC, PM2.5+si NC, PM2.5+si- KLF9#1, PM2.5+si- KLF9#2) (Figure 10L-Mito-Cytc) The expression of Cytc expression in mitochondria of HTR8/SVneo (si-NC, PM2.5+si NC, PM2.5+si-KLF9#1, PM2.5+si-KLF9#2, PM2.5+CYP1 A1 overexpression, PM2.5+si-KLF9#1+CYP1 A1 overexpression, PM2.5+si-KLF9#2+CYP1 A1 overexpression). (Figure 10L-Mito-VDAC-1) The expression of VDAC-1 expression in mitochondria of HTR8 [file elife-85944-fig10-data1.zip › Figure 10-source data 1/Figure10B-KLF9.TIF]

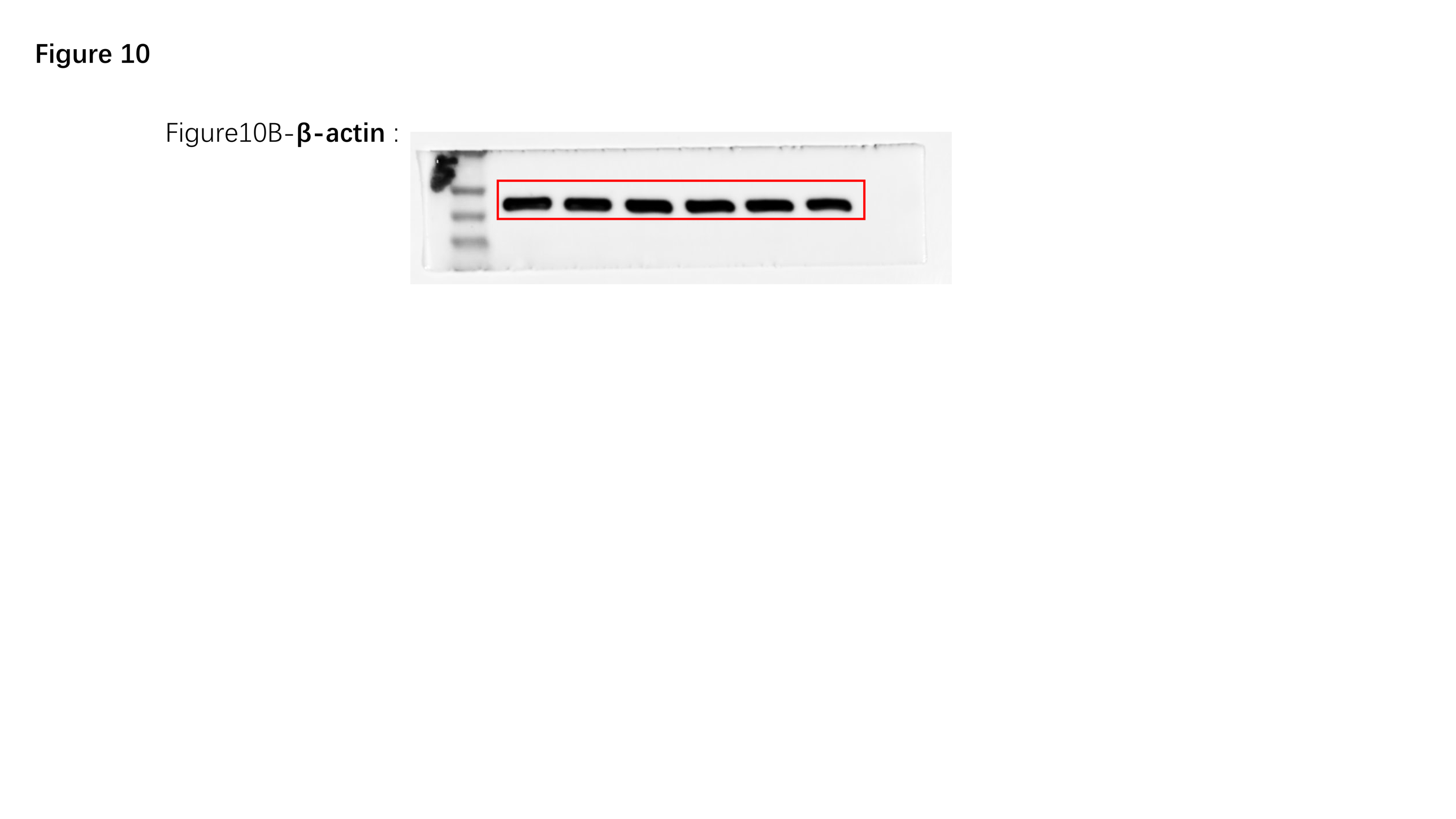

Supplement: Figure 10—source data 1. — (Figure 10B-KLF9) The expression of KLF9 expression in the placental tissue of Wild Type (The first three columns) and PM2.5-treated mice (the last three columns). (Figure 10B-β-actin) The expression of β-actin expression in the placental tissue of Wild Type (The first three columns) and PM2.5-treated mice (the last three columns). (Figure 10C-KLF9) The expression of KLF9 expression in the PM2.5-treated HTR8/SVneo cells (PM2.5 concentration: 0. 50 μg/mL, 100 μg/mL, 200 μg/mL). (Figure 10C-β-actin) The expression of β-actin expression in the PM2.5-treated HTR8/SVneo cells (PM2.5 concentration: 0. 50 μg/mL, 100 μg/mL, 200 μg/mL). (Figure 10K-Mito-Cytc) The expression of Cytc expression in mitochondria of HTR8/SVneo (si-NC, PM2.5+si NC, PM2.5+si-KLF9#1, PM2.5+si-KLF9#2). (Figure 10K-Mito-VDAC-1) The expression of VDAC-1 expression in mitochondria of HTR8/SVneo (si-NC, PM2.5+si NC, PM2.5+si-KLF9#1, PM2.5+si-KLF9#2). (Figure 10K-Cyto-Cytc) The expression of Cytc expression in cytoplasm of HTR8/SVneo (si-NC, PM2.5+si NC, PM2.5+si-KLF9#1, PM2.5+si-KLF9#2). (Figure 10K-Cyto-BCL-2) The expression of BCL-2 expression in cytoplasm of HTR8/SVneo (si-NC, PM2.5+si NC, PM2.5+si- KLF9#1, PM2.5+si- KLF9#2). (Figure 10K-Cyto-BAX) The expression of BAX expression in cytoplasm of HTR8/SVneo (si-NC, PM2.5+si NC, PM2.5+si- KLF9#1, PM2.5+si- KLF9#2). (Figure 10K-Cyto-Cleaved-Caspase3) The expression of Cleaved-Caspase3 expression in cytoplasm of HTR8/SVneo (si-NC, PM2.5+si NC, PM2.5+si- KLF9#1, PM2.5+si- KLF9#2). (Figure 10K-Cyto-β-actin) The expression of β-actin expression in cytoplasm of HTR8/SVneo (si-NC, PM2.5+si NC, PM2.5+si- KLF9#1, PM2.5+si- KLF9#2) (Figure 10L-Mito-Cytc) The expression of Cytc expression in mitochondria of HTR8/SVneo (si-NC, PM2.5+si NC, PM2.5+si-KLF9#1, PM2.5+si-KLF9#2, PM2.5+CYP1 A1 overexpression, PM2.5+si-KLF9#1+CYP1 A1 overexpression, PM2.5+si-KLF9#2+CYP1 A1 overexpression). (Figure 10L-Mito-VDAC-1) The expression of VDAC-1 expression in mitochondria of HTR8 [file elife-85944-fig10-data1.zip › Figure 10-source data 1/Figure10B-a┬-actin.TIF]
