# Supplementary material for: PM2.5 leads to adverse pregnancy outcomes by inducing trophoblast oxidative stress and mitochondrial apoptosis via KLF9/CYP1A1 transcriptional axis
Source: eLife. 2023 Sep 22;12:e85944. doi: 10.7554/eLife.85944 (PMC10584374; doi:10.7554/eLife.85944)
Supplement: Figure 5—source data 1. — (Figure 5B-HO-1) The expression of HO-1 expression in the placental tissue of Wild Type (The first three columns) and PM2.5-treated mice (the last three columns) (Figure 5B-NQO-1). The expression of NQO-1 expression in the placental tissue of Wild Type (The first three columns) and PM2.5-treated mice (the last three columns) (Figure 5B-GCLC). The expression of GCLC expression in the placental tissue of Wild Type (The first three columns) and PM2.5-treated mice (the last three columns) (Figure 5B-SOD-1). The expression of SOD-1 expression in the placental tissue of Wild Type (The first three columns) and PM2.5-treated mice (the last three columns) (Figure 5B-β-actin). The expression of β-actin expression in the placental tissue of Wild Type (The first three columns) and PM2.5-treated mice (the last three columns) (Figure 5C-HO-1). The expression of HO-1 expression in the PM2.5-treated HTR8/SVneo cells (PM2.5 concentration: 0. 50 μg/mL, 100 μg/mL, 200 μg/mL) (Figure 5C-NQO-1). The expression of NQO-1 expression in the PM2.5-treated HTR8/SVneo cells (PM2.5 concentration: 0. 50 μg/mL, 100 μg/mL, 200 μg/mL) (Figure 5C-GCLC). The expression of GCLC expression in the PM2.5-treated HTR8/SVneo cells (PM2.5 concentration: 0. 50 μg/mL, 100 μg/mL, 200 μg/mL) (Figure 5C-SOD-1). The expression of SOD-1 expression in the PM2.5-treated HTR8/SVneo cells (PM2.5 concentration: 0. 50 μg/mL, 100 μg/mL, 200 μg/mL) (Figure 5C-β-actin). The expression of β-actin expression in the PM2.5-treated HTR8/SVneo cells (PM2.5 concentration: 0. 50 μg/mL, 100 μg/mL, 200 μg/mL) (Figure 5K-Mito-Cyt-c). The expression of Cyt-c expression in mitochondria after treated with different concentration of PM2.5 (0, 50 μg/mL, 100 μg/mL, 200 μg/mL) (Figure 5K-Mito-VDAC-1). The expression of VDAC-1 expression in mitochondria after treated with different concentration of PM2.5 (0, 50 μg/mL, 100 μg/mL, 200 μg/mL) (Figure 5K-Cyto-Cyt-c). The expression of Cyt-c expression in cytoplasm after treated with different co [file elife-85944-fig5-data1.zip › Figure 5-source data 1/Figure5-source data1-Figure legends.docx]

**Figure5B-HO-1** The expression of HO-1 expression in the placental tissue of Wild Type (The first three columns) and PM2.5-treated mice (the last three columns).

**Figure5B-NQO-1** The expression of NQO-1 expression in the placental tissue of Wild Type (The first three columns) and PM2.5-treated mice (the last three columns).

**Figure5B-GCLC** The expression of GCLC expression in the placental tissue of Wild Type (The first three columns) and PM2.5-treated mice (the last three columns).

**Figure5B-SOD-1** The expression of SOD-1 expression in the placental tissue of Wild Type (The first three columns) and PM2.5-treated mice (the last three columns).

**Figure5B-β-actin** The expression of β-actin expression in the placental tissue of Wild Type (The first three columns) and PM2.5-treated mice (the last three columns).

**Figure5C-HO-1** The expression of HO-1 expression in the PM2.5-treated HTR8/SVneo cells (PM2.5 concentration: 0. 50μg/mL, 100μg/mL, 200μg/mL)

**Figure5C-NQO-1** The expression of NQO-1 expression in the PM2.5-treated HTR8/SVneo cells (PM2.5 concentration: 0. 50μg/mL, 100μg/mL, 200μg/mL)

**Figure5C-GCLC** The expression of GCLC expression in the PM2.5-treated HTR8/SVneo cells (PM2.5 concentration: 0. 50μg/mL, 100μg/mL, 200μg/mL)

**Figure5C-SOD-1** The expression of SOD-1 expression in the PM2.5-treated HTR8/SVneo cells (PM2.5 concentration: 0. 50μg/mL, 100μg/mL, 200μg/mL)

**Figure5C-β-actin** The expression of β-actin expression in the PM2.5-treated HTR8/SVneo cells (PM2.5 concentration: 0. 50μg/mL, 100μg/mL, 200μg/mL)

**Figure5K-Mito-Cyt-c** The expression of Cyt-c expression in mitochondria after treated with different concentration of PM2.5 (0, 50μg/mL, 100μg/mL, 200μg/mL)

**Figure5K-Mito-VDAC-1** The expression of VDAC-1 expression in mitochondria after treated with different concentration of PM2.5 (0, 50μg/mL, 100μg/mL, 200μg/mL)

**Figure5K-Cyto-Cyt-c** The expression of Cyt-c expression in cytoplasm after treated with different concentration of PM2.5 (0, 50μg/mL, 100μg/mL, 200μg/mL)

**Figure5K-Cyto-BCL-2** The expression of BCL-2 expression in cytoplasm after treated with different concentration of PM2.5 (0, 50μg/mL, 100μg/mL, 200μg/mL)

**Figure5K-Cyto-BAX** The expression of BAX expression in cytoplasm after treated with different concentration of PM2.5 (0, 50μg/mL, 100μg/mL, 200μg/mL)

**Figure5K-Cyto-CC3** The expression of Cleaved-Caspase 3 expression in cytoplasm after treated with different concentration of PM2.5 (0, 50μg/mL, 100μg/mL, 200μg/mL)

**Figure5K-Cyto-****β-actin** The expression of β-actin expression in cytoplasm after treated with different concentration of PM2.5 (0, 50μg/mL, 100μg/mL, 200μg/mL)
