# Supplementary material for: PM2.5 leads to adverse pregnancy outcomes by inducing trophoblast oxidative stress and mitochondrial apoptosis via KLF9/CYP1A1 transcriptional axis
Source: eLife. 2023 Sep 22;12:e85944. doi: 10.7554/eLife.85944 (PMC10584374; doi:10.7554/eLife.85944)
Supplement: Figure 6—source data 1. — (Figure 6N-BCL-2) The expression of BCL-2 expression in HTR8/SVneo (CON, PM2.5, PM2.5+NAC). (Figure 6N-BAX) The expression of BAX expression in HTR8/SVneo (CON, PM2.5, PM2.5+NAC). (Figure 6N-CC3) The expression of Cleaved-Caspase 3 expression in HTR8/SVneo (CON, PM2.5, PM2.5+NAC). (Figure 6N-β-actin) The expression of β-actin expression in HTR8/SVneo (CON, PM2.5, PM2.5+NAC). [file elife-85944-fig6-data1.zip › Figure 6-source data 1/Figure6-source data1-Figure legends.docx]

**Figure6N-BCL-2** The expression of BCL-2 expression in HTR8/SVneo (CON, PM2.5, PM2.5+NAC).

**Figure6N-BAX** The expression of BAX expression in HTR8/SVneo (CON, PM2.5, PM2.5+NAC).

**Figure6N-CC3** The expression of Cleaved-Caspase 3 expression in HTR8/SVneo (CON, PM2.5, PM2.5+NAC).

**Figure6N-β-actin** The expression of β-actin expression in HTR8/SVneo (CON, PM2.5, PM2.5+NAC).
