# Supplementary material for: PM2.5 leads to adverse pregnancy outcomes by inducing trophoblast oxidative stress and mitochondrial apoptosis via KLF9/CYP1A1 transcriptional axis
Source: eLife. 2023 Sep 22;12:e85944. doi: 10.7554/eLife.85944 (PMC10584374; doi:10.7554/eLife.85944)
Supplement: Figure 7—source data 1. — (Figure 7B-CYP1A1) The expression of CYP1A1 expression in the placental tissue of Wild Type (The first three columns) and PM2.5-treated mice (the last three columns). (Figure 7B-β-actin) The expression of β-actin expression in the placental tissue of Wild Type (The first three columns) and PM2.5-treated mice (the last three columns). (Figure 7E-CYP1A1) The expression of CYP1A1 expression in the PM2.5-treated HTR8/SVneo cells (PM2.5 concentration: 0. 50 μg/mL, 100 μg/mL, 200 μg/mL). (Figure 7E-β-actin) The expression of β-actin expression in the PM2.5-treated HTR8/SVneo cells (PM2.5 concentration: 0. 50 μg/mL, 100 μg/mL, 200 μg/mL). (Figure 7F-CYP1A1) The expression of CYP1A1 expression in the HTR8/SVneo cells (si-NC, si-CYP1A1#1, si-CYP1A1#2). (Figure 7F-β-actin) The expression of β-actin expression in the HTR8/SVneo cells (si-NC, si-CYP1A1#1, si-CYP1A1#2). (Figure 7M-Mito-Cytc) The expression of Cytc expression in mitochondria of HTR8/SVneo (si-NC, PM2.5+si NC, PM2.5+si- CYP1A1#1, PM2.5+si- CYP1A1#2). (Figure 7M-Mito-VDAC-1) The expression of VDAC-1 expression in mitochondria of HTR8/SVneo (si-NC, PM2.5+si NC, PM2.5+si- CYP1A1#1, PM2.5+si- CYP1A1#2). (Figure 7M-Cyto-Cytc) The expression of Cytc expression in cytoplasm of HTR8/SVneo (si-NC, PM2.5+si NC, PM2.5+si- CYP1A1#1, PM2.5+si- CYP1A1#2). (Figure 7M-Cyto-BCL-2) The expression of BCL-2 expression in cytoplasm of HTR8/SVneo (si-NC, PM2.5+si NC, PM2.5+si- CYP1A1#1, PM2.5+si- CYP1A1#2). (Figure 7M-Cyto-BAX) The expression of BAX expression in cytoplasm of HTR8/SVneo (si-NC, PM2.5+si NC, PM2.5+si- CYP1A1#1, PM2.5+si- CYP1A1#2). (Figure 7M-Cyto-Cleaved-Caspase3) The expression of Cleaved-Caspase3 expression in cytoplasm of HTR8/SVneo (si-NC, PM2.5+si NC, PM2.5+si- CYP1A1#1, PM2.5+si- CYP1A1#2). (Figure 7M-Cyto-β-actin) The expression of β-actin expression in cytoplasm of HTR8/SVneo (si-NC, PM2.5+si NC, PM2.5+si- CYP1A1#1, PM2.5+si- CYP1A1#2). [file elife-85944-fig7-data1.zip › Figure 7-source data 1/Figure7-source data1-Figure legends.docx]

**Figure7B-CYP1A1** The expression of CYP1A1 expression in the placental tissue of Wild Type (The first three columns) and PM2.5-treated mice (the last three columns).

**Figure7B-****β-actin** The expression of β-actin expression in the placental tissue of Wild Type (The first three columns) and PM2.5-treated mice (the last three columns).

**Figure7E-CYP1A1** The expression of CYP1A1 expression in the PM2.5-treated HTR8/SVneo cells (PM2.5 concentration: 0. 50μg/mL, 100μg/mL, 200μg/mL)

**Figure7E-****β-actin** The expression of β-actin expression in the PM2.5-treated HTR8/SVneo cells (PM2.5 concentration: 0. 50μg/mL, 100μg/mL, 200μg/mL)

**Figure7F-CYP1A1** The expression of CYP1A1 expression in the HTR8/SVneo cells (si-NC, si-CYP1A1#1, si-CYP1A1#2)

**Figure7F-β-actin** The expression of β-actin expression in the HTR8/SVneo cells (si-NC, si-CYP1A1#1, si-CYP1A1#2)

**Figure7M-Mito-Cytc** The expression of Cytc expression in mitochondria of HTR8/SVneo (si-NC, PM2.5+si-NC, PM2.5+si- CYP1A1#1, PM2.5+si- CYP1A1#2)

**Figure7M-Mito-VDAC-1** The expression of VDAC-1 expression in mitochondria of HTR8/SVneo (si-NC, PM2.5+si-NC, PM2.5+si- CYP1A1#1, PM2.5+si- CYP1A1#2)

**Figure7M-Cyto-Cytc** The expression of Cytc expression in cytoplasm of HTR8/SVneo (si-NC, PM2.5+si-NC, PM2.5+si- CYP1A1#1, PM2.5+si- CYP1A1#2)

**Figure7M-Cyto-BCL-2** The expression of BCL-2 expression in cytoplasm of HTR8/SVneo (si-NC, PM2.5+si-NC, PM2.5+si- CYP1A1#1, PM2.5+si- CYP1A1#2)

**Figure7M-Cyto-BAX** The expression of BAX expression in cytoplasm of HTR8/SVneo (si-NC, PM2.5+si-NC, PM2.5+si- CYP1A1#1, PM2.5+si- CYP1A1#2)

**Figure7M-Cyto-****Cleaved-Caspase3** The expression of Cleaved-Caspase3 expression in cytoplasm of HTR8/SVneo (si-NC, PM2.5+si-NC, PM2.5+si- CYP1A1#1, PM2.5+si- CYP1A1#2)

**Figure7M-Cyto-β-actin** The expression of β-actin expression in cytoplasm of HTR8/SVneo (si-NC, PM2.5+si-NC, PM2.5+si- CYP1A1#1, PM2.5+si- CYP1A1#2)
