# Supplementary material for: PM2.5 leads to adverse pregnancy outcomes by inducing trophoblast oxidative stress and mitochondrial apoptosis via KLF9/CYP1A1 transcriptional axis
Source: eLife. 2023 Sep 22;12:e85944. doi: 10.7554/eLife.85944 (PMC10584374; doi:10.7554/eLife.85944)
Supplement: Figure 7—figure supplement 1—source data 1. — (Figure 7—figure supplement 1B–CYP1A1) The expression of CYP1A1 expression in the PM2.5-treated HTR8/SVneo cells (PM2.5 concentration: 0. 50 μg/mL, 100 μg/mL, 200 μg/mL). (Figure 7—figure supplement 1B–CYP1B1) The expression of CYP1B1 expression in the PM2.5-treated HTR8/SVneo cells (PM2.5 concentration: 0. 50 μg/mL, 100 μg/mL, 200 μg/mL). (Figure 7—figure supplement 1B–ALDH1A3) The expression of ALDH1A3 expression in the PM2.5-treated HTR8/SVneo cells (PM2.5 concentration: 0. 50 μg/mL, 100 μg/mL, 200 μg/mL). (Figure 7—figure supplement 1B-β-actin) The expression of β-actin expression in the PM2.5-treated HTR8/SVneo cells (PM2.5 concentration: 0. 50 μg/mL, 100 μg/mL, 200 μg/mL). [file elife-85944-fig7-figsupp1-data1.zip › Figure 7-figure supplement 1-source data 1/Figure7-figure supplement 1-source data1-Figure legends.docx]

**Figure7-figure supplement 1B-CYP1A1** The expression of CYP1A1 expression in the PM2.5-treated HTR8/SVneo cells (PM2.5 concentration: 0. 50μg/mL, 100μg/mL, 200μg/mL)

**Figure7-figure supplement 1B-CYP1B1** The expression of CYP1B1 expression in the PM2.5-treated HTR8/SVneo cells (PM2.5 concentration: 0. 50μg/mL, 100μg/mL, 200μg/mL)

**Figure7-figure supplement 1B-ALDH1A3** The expression of ALDH1A3 expression in the PM2.5-treated HTR8/SVneo cells (PM2.5 concentration: 0. 50μg/mL, 100μg/mL, 200μg/mL)

**Figure7-figure supplement 1B-β-actin** The expression of β-actin expression in the PM2.5-treated HTR8/SVneo cells (PM2.5 concentration: 0. 50μg/mL, 100μg/mL, 200μg/mL)
