# Supplementary material for: PM2.5 leads to adverse pregnancy outcomes by inducing trophoblast oxidative stress and mitochondrial apoptosis via KLF9/CYP1A1 transcriptional axis
Source: eLife. 2023 Sep 22;12:e85944. doi: 10.7554/eLife.85944 (PMC10584374; doi:10.7554/eLife.85944)
Supplement: Figure 9—source data 1. — (Figure 9E-KLF9) The expression of KLF9 expression in the HTR8/SVneo (si-NC, si- KLF9#1, si- KLF9#2). (Figure 9E-CYP1A1) The expression of CYP1A1 expression in the HTR8/SVneo (si-NC, si- KLF9#1, si- KLF9#2). (Figure 9E-β-actin) The expression of β-actin expression in the HTR8/SVneo (si-NC, si- KLF9#1, si- KLF9#2). (Figure 9E-Vector-KLF9-KLF9) The expression of KLF9 expression in the HTR8/SVneo (CON, KLF9 over expression). (Figure 9E-Vector-KLF9-CYP1A1) The expression of CYP1A1 expression in the HTR8/SVneo (CON, KLF9 over expression). (Figure 9E-Vector-KLF9-β-actin) The expression of β-actin expression in the HTR8/SVneo (CON, KLF9 over expression). (Figure 9G-ChIP) Chromatin from KLF9 over-expression HTR8/SVneo cells was subjected to ChIP assay using KLF9 antibody or control IgG. PCR amplification with primers spanning the –96/+16 bp region of the CYP1A1 promoter was performed. A 2% agarose gel electrophoresis was performed on PCR products. [file elife-85944-fig9-data1.zip › Figure 9-source data 1/Figure9-source data1-Figure legends.docx]

**Figure9E-KLF9** The expression of KLF9 expression in the HTR8/SVneo (si-NC, si- KLF9#1, si- KLF9#2)

**Figure9E-CYP1A1** The expression of CYP1A1 expression in the HTR8/SVneo (si-NC, si- KLF9#1, si- KLF9#2)

**Figure9E-β-actin** The expression of β-actin expression in the HTR8/SVneo (si-NC, si- KLF9#1, si- KLF9#2)

**Figure9E- Vector-KLF9-KLF9** The expression of KLF9 expression in the HTR8/SVneo (CON, KLF9 over expression)

**Figure9E-Vector-KLF9-CYP1A1** The expression of CYP1A1 expression in the HTR8/SVneo (CON, KLF9 over expression)

**Figure9E-Vector-KLF9-β-actin** The expression of β-actin expression in the HTR8/SVneo (CON, KLF9 over expression)

**Figure9G-ChIP** Chromatin from KLF9 over-expression HTR8/SVneo cells was subjected to ChIP assay using KLF9 antibody or control IgG. PCR amplification with primers spanning the -96/+16 bp region of the CYP1A1 promoter was performed. A 2% agarose gel electrophoresis was performed on PCR products.
