# Supplementary material for: PM2.5 leads to adverse pregnancy outcomes by inducing trophoblast oxidative stress and mitochondrial apoptosis via KLF9/CYP1A1 transcriptional axis
Source: eLife. 2023 Sep 22;12:e85944. doi: 10.7554/eLife.85944 (PMC10584374; doi:10.7554/eLife.85944)
Supplement: Figure 10—source data 1. — (Figure 10B-KLF9) The expression of KLF9 expression in the placental tissue of Wild Type (The first three columns) and PM2.5-treated mice (the last three columns). (Figure 10B-β-actin) The expression of β-actin expression in the placental tissue of Wild Type (The first three columns) and PM2.5-treated mice (the last three columns). (Figure 10C-KLF9) The expression of KLF9 expression in the PM2.5-treated HTR8/SVneo cells (PM2.5 concentration: 0. 50 μg/mL, 100 μg/mL, 200 μg/mL). (Figure 10C-β-actin) The expression of β-actin expression in the PM2.5-treated HTR8/SVneo cells (PM2.5 concentration: 0. 50 μg/mL, 100 μg/mL, 200 μg/mL). (Figure 10K-Mito-Cytc) The expression of Cytc expression in mitochondria of HTR8/SVneo (si-NC, PM2.5+si NC, PM2.5+si-KLF9#1, PM2.5+si-KLF9#2). (Figure 10K-Mito-VDAC-1) The expression of VDAC-1 expression in mitochondria of HTR8/SVneo (si-NC, PM2.5+si NC, PM2.5+si-KLF9#1, PM2.5+si-KLF9#2). (Figure 10K-Cyto-Cytc) The expression of Cytc expression in cytoplasm of HTR8/SVneo (si-NC, PM2.5+si NC, PM2.5+si-KLF9#1, PM2.5+si-KLF9#2). (Figure 10K-Cyto-BCL-2) The expression of BCL-2 expression in cytoplasm of HTR8/SVneo (si-NC, PM2.5+si NC, PM2.5+si- KLF9#1, PM2.5+si- KLF9#2). (Figure 10K-Cyto-BAX) The expression of BAX expression in cytoplasm of HTR8/SVneo (si-NC, PM2.5+si NC, PM2.5+si- KLF9#1, PM2.5+si- KLF9#2). (Figure 10K-Cyto-Cleaved-Caspase3) The expression of Cleaved-Caspase3 expression in cytoplasm of HTR8/SVneo (si-NC, PM2.5+si NC, PM2.5+si- KLF9#1, PM2.5+si- KLF9#2). (Figure 10K-Cyto-β-actin) The expression of β-actin expression in cytoplasm of HTR8/SVneo (si-NC, PM2.5+si NC, PM2.5+si- KLF9#1, PM2.5+si- KLF9#2) (Figure 10L-Mito-Cytc) The expression of Cytc expression in mitochondria of HTR8/SVneo (si-NC, PM2.5+si NC, PM2.5+si-KLF9#1, PM2.5+si-KLF9#2, PM2.5+CYP1 A1 overexpression, PM2.5+si-KLF9#1+CYP1 A1 overexpression, PM2.5+si-KLF9#2+CYP1 A1 overexpression). (Figure 10L-Mito-VDAC-1) The expression of VDAC-1 expression in mitochondria of HTR8 [file elife-85944-fig10-data1.zip › Figure 10-source data 1/Figure10-source data1-Figure legends.docx]

**Figure10B-KLF9** The expression of KLF9 expression in the placental tissue of Wild Type (The first three columns) and PM2.5-treated mice (the last three columns).

**Figure10B-β-actin** The expression of β-actin expression in the placental tissue of Wild Type (The first three columns) and PM2.5-treated mice (the last three columns).

**Figure10C-KLF9** The expression of KLF9 expression in the PM2.5-treated HTR8/SVneo cells (PM2.5 concentration: 0. 50μg/mL, 100μg/mL, 200μg/mL)

**Figure10C-β-actin** The expression of β-actin expression in the PM2.5-treated HTR8/SVneo cells (PM2.5 concentration: 0. 50μg/mL, 100μg/mL, 200μg/mL)

**Figure10K-Mito-Cytc** The expression of Cytc expression in mitochondria of HTR8/SVneo (si-NC, PM2.5+si-NC, PM2.5+si-KLF9#1, PM2.5+si-KLF9#2)

**Figure10K-Mito-VDAC-1** The expression of VDAC-1 expression in mitochondria of HTR8/SVneo (si-NC, PM2.5+si-NC, PM2.5+si-KLF9#1, PM2.5+si-KLF9#2)

**Figure10K-Cyto-Cytc** The expression of Cytc expression in cytoplasm of HTR8/SVneo (si-NC, PM2.5+si-NC, PM2.5+si-KLF9#1, PM2.5+si-KLF9#2)

**Figure10K-Cyto-BCL-2** The expression of BCL-2 expression in cytoplasm of HTR8/SVneo (si-NC, PM2.5+si-NC, PM2.5+si- KLF9#1, PM2.5+si- KLF9#2)

**Figure10K-Cyto-BAX** The expression of BAX expression in cytoplasm of HTR8/SVneo (si-NC, PM2.5+si-NC, PM2.5+si- KLF9#1, PM2.5+si- KLF9#2)

**Figure10K-Cyto-Cleaved-Caspase3** The expression of Cleaved-Caspase3 expression in cytoplasm of HTR8/SVneo (si-NC, PM2.5+si-NC, PM2.5+si- KLF9#1, PM2.5+si- KLF9#2)

**Figure10K-Cyto-β-actin** The expression of β-actin expression in cytoplasm of HTR8/SVneo (si-NC, PM2.5+si-NC, PM2.5+si- KLF9#1, PM2.5+si- KLF9#2)

**Figure10L-Mito-Cytc** The expression of Cytc expression in mitochondria of HTR8/SVneo (si-NC, PM2.5+si-NC, PM2.5+si-KLF9#1, PM2.5+si-KLF9#2, PM2.5+CYP1A1 overexpression, PM2.5+si-KLF9#1+ CYP1A1 overexpression, PM2.5+si-KLF9#2+ CYP1A1 overexpression)

**Figure10L-Mito-VDAC-1** The expression of VDAC-1 expression in mitochondria of HTR8/SVneo (si-NC, PM2.5+si-NC, PM2.5+si-KLF9#1, PM2.5+si-KLF9#2, PM2.5+CYP1A1 overexpression, PM2.5+si-KLF9#1+ CYP1A1 overexpression, PM2.5+si-KLF9#2+ CYP1A1 overexpression)

**Figure10L-Cyto-Cytc** The expression of Cytc expression in cytoplasm of HTR8/SVneo (si-NC, PM2.5+si-NC, PM2.5+si-KLF9#1, PM2.5+si-KLF9#2, PM2.5+CYP1A1 overexpression, PM2.5+si-KLF9#1+ CYP1A1 overexpression, PM2.5+si-KLF9#2+ CYP1A1 overexpression)

**Figure10L-Cyto-BCL-2** The expression of BCL-2 expression in cytoplasm of HTR8/SVneo (si-NC, PM2.5+si-NC, PM2.5+si-KLF9#1, PM2.5+si-KLF9#2, PM2.5+CYP1A1 overexpression, PM2.5+si-KLF9#1+ CYP1A1 overexpression, PM2.5+si-KLF9#2+ CYP1A1 overexpression)

**Figure10L-Cyto-BAX** The expression of BAX expression in cytoplasm of HTR8/SVneo (si-NC, PM2.5+si-NC, PM2.5+si-KLF9#1, PM2.5+si-KLF9#2, PM2.5+CYP1A1 overexpression, PM2.5+si-KLF9#1+ CYP1A1 overexpression, PM2.5+si-KLF9#2+ CYP1A1 overexpression)

**Figure10L-Cyto-Cleaved-Caspase3** The expression of Cleaved-Caspase3 expression in cytoplasm of HTR8/SVneo (si-NC, PM2.5+si-NC, PM2.5+si-KLF9#1, PM2.5+si-KLF9#2, PM2.5+CYP1A1 overexpression, PM2.5+si-KLF9#1+ CYP1A1 overexpression, PM2.5+si-KLF9#2+ CYP1A1 overexpression)

**Figure10L-Cyto-β-actin** The expression of β-actin expression in cytoplasm of HTR8/SVneo (si-NC, PM2.5+si-NC, PM2.5+si-KLF9#1, PM2.5+si-KLF9#2, PM2.5+CYP1A1 overexpression, PM2.5+si-KLF9#1+ CYP1A1 overexpression, PM2.5+si-KLF9#2+ CYP1A1 overexpression)
