# Supplementary material for: PM2.5 leads to adverse pregnancy outcomes by inducing trophoblast oxidative stress and mitochondrial apoptosis via KLF9/CYP1A1 transcriptional axis
Source: eLife. 2023 Sep 22;12:e85944. doi: 10.7554/eLife.85944 (PMC10584374; doi:10.7554/eLife.85944)
Supplement: Figure 10—figure supplement 1—source data 1. — (Figure 10—figure supplement 1A–KLF9) The expression of KLF9 expression in the HTR8/SVneo cell nucleus after treated with PM2.5 (PM2.5 concentration: 0. 50 μg/mL, 100 μg/mL, 200 μg/mL). (Figure 10—figure supplement 1B–Lamin B) The expression of Lamin B expression in the HTR8/SVneo cell nucleus after treated with PM2.5 (PM2.5 concentration: 0. 50 μg/mL, 100 μg/mL, 200 μg/mL). [file elife-85944-fig10-figsupp1-data1.zip › Figure10-figure supplement 1-source data 1/Figure10-figure supplement 1-source data1-Figure legends.docx]

**Figure10-figure supplement 1A-KLF9** The expression of KLF9 expression in the HTR8/SVneo cell nucleus after treated with PM2.5 (PM2.5 concentration: 0. 50μg/mL, 100μg/mL, 200μg/mL)

**Figure10-figure supplement 1B-Lamin B** The expression of Lamin B expression in the HTR8/SVneo cell nucleus after treated with PM2.5 (PM2.5 concentration: 0. 50μg/mL, 100μg/mL, 200μg/mL)
