# Supplementary material for: PM2.5 leads to adverse pregnancy outcomes by inducing trophoblast oxidative stress and mitochondrial apoptosis via KLF9/CYP1A1 transcriptional axis
Source: eLife. 2023 Sep 22;12:e85944. doi: 10.7554/eLife.85944 (PMC10584374; doi:10.7554/eLife.85944)
Supplement: Supplementary file 1. [file elife-85944-supp1.docx]

**Supplementary File 1.** The gestation and characteristics of the pregnant women (n=31).

| **Characteristics** | **Pregnant women**  **(n=31)** |
| --- | --- |
| Age (year) | 28.581±2.592 |
| Gestational age (week) | 39.368±0.412 |
| Body mass index | 26.897±2.205 |
| Proteinuria (mg/day) | 0.030±0.035 |
| Systolic blood pressure (mm/Hg) | 120.581±6.402 |
| Diastolic blood pressure (mm/Hg) | 82.387±4.470 |
| Birth weight (g) | 3289.852±288.949 |
| Smoking status | 4 |
| Abnormal fetus | No |
| Fetal growth restriction | No |
