# Supplementary material for: PM2.5 leads to adverse pregnancy outcomes by inducing trophoblast oxidative stress and mitochondrial apoptosis via KLF9/CYP1A1 transcriptional axis
Source: eLife. 2023 Sep 22;12:e85944. doi: 10.7554/eLife.85944 (PMC10584374; doi:10.7554/eLife.85944)
Supplement: Supplementary file 2. [file elife-85944-supp2.docx]

**Supplementary File 2.** The sequence of siRNAs used in this study.

| **siRNA** | **Sequence (5'to3')** | **Purpose** |
| --- | --- | --- |
| si*CYP1A1*#1 | Forward: GGUAUGUGGUGGUAUCAGUTT | Knockdown |
|  | Reverse: ACUGAUACCACCACAUACCTT |  |
| si*CYP1A1*#2 | Forward: CCUUCAAGGACCUGAAUGATT | Knockdown |
|  | Reverse: UCAUUCAGGUCCUUGAAGGTT |  |
| si*KLF9*#1 | Forward: GCCCAUUACAGAGUGCAUATT | Knockdown |
|  | Reverse: UAUGCACUCUGUAAUGGGCTT |  |
| si*KLF9*#2 | Forward: GGAGUGACCACCUCACAAATT | Knockdown |
|  | Reverse: UUUGUGAGGUGGUCACUCCTT |  |
