# Supplementary material for: PM2.5 leads to adverse pregnancy outcomes by inducing trophoblast oxidative stress and mitochondrial apoptosis via KLF9/CYP1A1 transcriptional axis
Source: eLife. 2023 Sep 22;12:e85944. doi: 10.7554/eLife.85944 (PMC10584374; doi:10.7554/eLife.85944)
Supplement: Supplementary file 3. [file elife-85944-supp3.docx]

**Supplementary File 3.** The sequence of primers used in this study.

| **Primer** | **Sequence (5'to3')** | **Purpose** |
| --- | --- | --- |
| CYP1A1 | Forward: TGGCATCCTCTACAGACTCCTG | qPCR |
|  | Reverse: CTTCAGGTTGCGTGCCATCTCA |  |
| KLF9 | Forward: CTACAGTGGCTGTGGGAAAGTC | qPCR |
|  | Reverse: CTCGTCTGAGCGGGAGAACTTT |  |
| GAPDH | Forward: GTCTCCTCTGACTTCAACAGCG | qPCR |
|  | Reverse: ACCACCCTGTTGCTGTAGCCAA |  |
| CYP1A1-promoter-primer | Forward: CTGCTTCTCCCTCCATCT | ChIP-qPCR |
|  | Reverse: GGAACTGTCACCTTCAGG |  |
| CYP1B1 | Forward: GTGTGGAAGGAGCACTTTGAGG | qPCR |
|  | Reverse: GATGCTGGTGTACTGTTGAGGG |  |
| ALDH1A3 | Forward: CTGCTACAACGCCCTCTATGCA | qPCR |
|  | Reverse: GTCGCCAAGTTTGATGGTGACAG |  |
