# Supplementary material for: PM2.5 leads to adverse pregnancy outcomes by inducing trophoblast oxidative stress and mitochondrial apoptosis via KLF9/CYP1A1 transcriptional axis
Source: eLife. 2023 Sep 22;12:e85944. doi: 10.7554/eLife.85944 (PMC10584374; doi:10.7554/eLife.85944)
Supplement: Supplementary file 4. [file elife-85944-supp4.docx]

**Supplementary File 4.** The information of antibodies used in this study.

| **Antibodies** | **Identifier** | **Purpose** |
| --- | --- | --- |
| CYP1A1 | Proteintech Cat#13241-1-AP | WB IHC |
| KLF9 | Abcam Cat#ab227920 | WB IHC ChIP |
| HO-1 | Cohesion Biosciences Cat#CQA2561 | WB |
| NQO-1 | Cohesion Biosciences Cat#CPA1342 | WB |
| GCLC | Cohesion Biosciences Cat#CPA2092 | WB |
| SOD-1 | Cohesion Biosciences Cat#CPA1476 | WB |
| CK-7 | Abcam Cat#ab68459 | IHC |
| β-actin | Proteintech Cat#66009 | WB |
| Lamin B1 | Proteintech Cat# 12987-1-AP | WB |
| VDAC1 | Proteintech Cat#55259-1-AP | WB |
| BAX | Cell Signalling technology Cat#2772 | WB |
| BCL-2 | Cell Signalling technology Cat#15071 | WB |
| Cleaved-caspase 3 | Cell Signalling technology Cat#9661 | WB |
| Cytochrome-C | Cell Signalling technology Cat#4280 | WB |
| CYP1B1 | Cell Signalling technologyCat#9661 | WB |
| ALDH1A3 | Cell Signalling technologyCat#4280 | WB |
